# Palladium-catalysed Tsuji–Trost-type vinyl epoxide cross-coupling with umpolung hydrazones†

**DOI:** 10.1039/d4sc05411c

**Published:** 2024-10-16

**Authors:** Evan F. W. Chen, Chao-Jun Li

**Affiliations:** a Department of Chemistry, FRQNT Centre for Green Chemistry and Catalysis, McGill University 801 Sherbrooke Street West Montreal Quebec H3A 0B8 Canada cj.li@mcgill.ca

## Abstract

Selective functionalisation of synthetically useful vinyl epoxides *via* carbon–carbon (C–C) bond formation has been a major challenge for many years due to its unique inherent chemical reactivity. Non-stabilised carbanions in the form of organometallic reagents have been shown to be robust and versatile reagents in C–C bond formation; however, they are employed in superstoichiometric quantities, require the protection of active functional groups, and generate copious amounts of metallic waste. Therefore, the development of mild carbanion sources as simple alternatives is highly desired. In this work, we report a highly chemo- and regioselective palladium-catalysed vinyl epoxide cross-coupling utilising hydrazones as organometallic equivalents (HOME). Hydrazones, generated from carbonyl-containing renewable feedstocks, enable a more sustainable reaction, and provide an alternative to highly reactive and sensitive unstabilized organometallic reagents. A broad substrate scope, with high functional group tolerance, is demonstrated along with the late-stage functionalisation of natural product derivatives.

## Abstract 2

A sustainable palladium-catalyzed vinyl epoxide cross coupling with umpolung hydrazones has been developed. This platform provides access to branched homoallylic alcohols in a regioselective manner.

## Graphical abstract

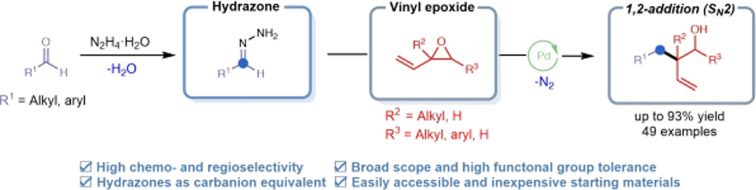

## Introduction

Characterised by a C–C double bond adjacent to an oxirane ring, vinyl epoxides have been recognised as a crucial building block in a diverse array of reactions.^1^ Vinyl epoxides exhibit unique reactivity, often acting as potent electrophiles^2–4^ and radical acceptors,^5^ and serve as substrates in sigmatropic rearrangements,^6^ showcasing their versatility in various synthetic transformations. With three different electrophilic sites, these substrates can be readily transformed into synthetically useful linear allylic or branched homoallylic alcohols *via* S_N_2′ (1,4-addition) and S_N_2-type (1,2-addition) ring openings. Over the past few decades, various studies have employed numerous unstabilized carbon nucleophiles for vinyl epoxide functionalisation.^1,7–10^ Despite these efforts, many alkylation methodologies frequently yielded mixtures of S_N_2 and S_N_2′ products,^7,10,11^ and site-selective alkylation of vinyl epoxides has proven to be difficult. Owing to the inherent steric interactions between the alkyl carbanion and the vinyl epoxide, S_N_2′ alkylation onto the least hindered alkenyl terminus is typically observed, while exclusive S_N_2 alkylation is uncommon.

**Fig. 1:**
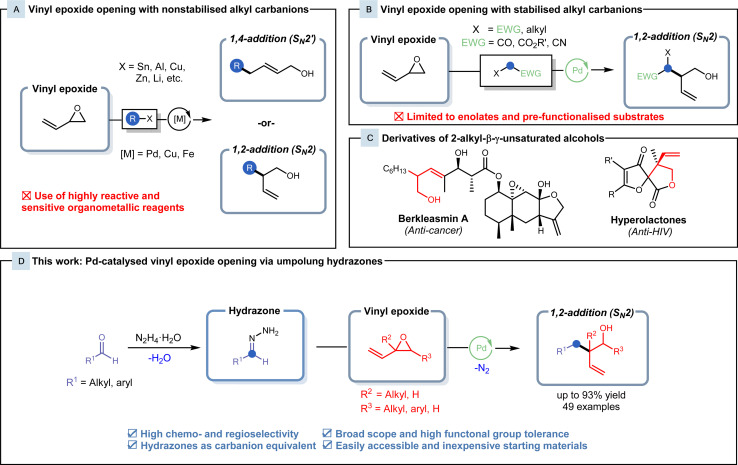
(A) Known methods in metal-catalysed vinyl epoxide opening with non-stabilised alkyl carbanions. (B) Recent reports on Pd-catalysed S_N_2-type vinyl epoxide opening with stabilised alkyl carbanions. (C) Bio-active natural products containing 2-alkyl-β-γ-unsaturated alcohol motifs. (D) This work: regioselective synthesis of 2-alkyl-β-γ-unsaturated alcohols *via* Pd-catalysed Tsuji–Trost type vinyl epoxide opening with HOME chemistry.

The development of transition metal catalysis provides a venue for the regioselective alkylation of vinyl epoxides. Since its discovery in 1973, the palladium-catalysed Tsuji–Trost allylation has been a cornerstone for allylic alkylations.^12,13^ Enhanced with the introduction of chiral ligands,^14^ this reaction has been demonstrated to be a powerful tool in crafting intricate, chiral molecules.^15–17^ With structural similarities shared between vinyl epoxides and allyl acetates, Trost and his colleagues broadened the scope to encompass the alkylation of vinyl epoxides in 1981.^18^ Non-stabilised alkyl carbanions, such as Grignard, organolithium, organozincate and organostannane reagents, have been shown to be robust and versatile nucleophiles in the generation of new C–C bonds with vinyl epoxides (Fig. 1A).^19–22^ Nevertheless, limitations are presented in these methodologies, as they employ highly reactive and air/moisture-sensitive reagents with low chemoselectivity, often requiring handling under inert conditions during preparation and use. Organometallic reagents also generate stoichiometric amounts of toxic metallic waste and show a high dependence on alkyl halide precursors from non-renewable petroleum feedstocks. The use of non-renewable carbon resources does not provide a route for sustainable chemistry, and therefore, the development of alternatives to organometallic reagents is highly desired.

Carbonyl-derived pronucleophiles in conjunction with palladium catalysis have recently been reported to display selectivity towards S_N_2-type vinyl epoxide openings (Fig. 1B). Conventionally, malonate substrates are utilised as stabilised nucleophiles,^18^ but recently, this field has expanded to include enolates derived from pre-functionalised esters.^23,24^ In 2021, Lundgren and his coworkers reported a Pd-catalysed benzylation of isoprene with 2,2,2-trifluoroethyl (hetero)arylacetate ester enolates to generate lactone intermediates that were subjected to subsequent hydrolysis and decarboxylation to form the branched homoallylic alcohol.^25^ These 2-alkyl-β-γ-unsaturated alcohol products also possess high synthetic value, as their derivatives appear as core motifs of various natural products and bio-active compounds, such as berkleasmins and hyperolactones (Fig. 1C).^26,27^ Although these methods provide highly efficient routes for the synthesis of these branched homoallylic alcohols, pre-functionalised and commercially unavailable starting materials hinder the applicability of these methods.

Hydrazones generated from inexpensive, naturally occurring carbonyls can serve as alkyl carbanion equivalents and undergo various reactions with carbon electrophiles.^28,29^ In our research group, we have been extensively developing this methodology and exploring the use of hydrazones as organometallic equivalents (HOME) in classical nucleophilic addition and cross-coupling reactions.^28^ This Umpolung strategy allows for efficient transformations under mild conditions comparable to classical organometallic reagents, with the benefit of generating nitrogen gas and water as inert byproducts. Hydrazones also exhibit lower basicity and reactivity along with air/moisture stability in comparison to organometallic reagents, allowing for safer handling. Herein, we report the palladium-catalysed cross-coupling of vinyl epoxides with hydrazones as alkyl organometallic reagent surrogates to generate branched homoallylic alcohols with high regio- and chemoselectivity (Fig. 1D).

## Results and discussion

We began our investigation with the cross-coupling of benzaldehyde hydrazone 1aa (1.25 equiv) with vinyl epoxide (0.23 mmol) 2a using a Pd(0)/triphenylphosphine catalyst system (Table 1) with lithium *tert*-butoxide (2 equiv) as the base. Under these conditions, we primarily observed a C-alkylated product, and *N*-alkylated products were not detected. Only racemic branched homoallylic alcohol 3aa was observed in 29% yield and the linear allylic alcohol product 6 was not detected (entry 2). We hypothesised that only one equivalent of the base was required for the transformation, as the alkoxy-intermediate generated from the vinyl epoxide ring opening potentially acts as an equivalent of base to facilitate the deprotonation of the hydrazone (see ESI Fig. S1†). Vinyl epoxides are also known to undergo reactions and rearrangements in the presence of Lewis acids.^6,30^ While palladium is the catalyst for our system, it could also potentially act as a Lewis acid. This theory was validated through a control experiment with 1 equivalent of PEPPSI-IPr, where 3aa was not detected and we primarily observed decomposition of 2a. With these results, a lower Pd catalyst loading of 5% and 1 equivalent of base gave higher yields. Any further decrease of the Pd loading and base diminished the yield. *N*-heterocyclic carbene (NHC) ligands were previously reported to be an efficient ligand for the Pd-catalysed C-allylations of hydrazones.^31,32^ Electron-rich, bulky NHC ligands such as 1,3-bis(2,4,6-trimethylphenyl)imidazolium chloride (IMes HCl) and 1,3-bis(2,6-diisopropylphenyl)imidazolium chloride (IPr HCl) were tested in combination with Pd_2_(dba)_3_ and afforded 3aa with yields of 19% (entry 3) and 36% (entry 4). A pre-ligated IPr (PEPPSI-IPr) was very efficient in this transformation and gave 3aa in a 90% yield (entry 1). Other *tert*-butoxide salts, organic bases (1,8-diazabicyclo(5.4.0)undec-7-ene (DBU), 1,5,7-triazabicyclo(4.4.0)dec-5-ene (TBD)), and lithium salt bases were screened but all gave reduced yields (entries 8 and 9, see ESI Table S2†). Cs_2_CO_3_ was the only other base that gave 3aa with a high yield of 82% (entry 7). Finally, various solvents such as 2-MeTHF, dioxane, toluene, and DMF were screened, but THF remained as the best solvent for this reaction (see ESI Table S3†). Control experiments show that no homoallylic alcohol 3aa was produced in the absence of a Pd catalyst and base.

**Table 1:** Reaction optimisation for the Pd-catalysed vinyl epoxide opening with hydrazones

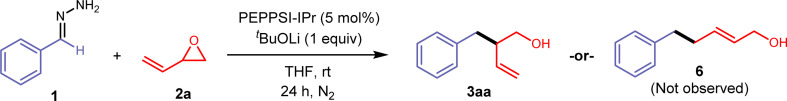		
Entry	Deviation from standard conditions^a^	3aa (yield%)
1	None	90
2	Pd_2_(dba)_3_ (5 mol%), PPh_3_ (10 mol%), 2 equiv ^*t*^BuOLi, 45 °C	29
3	Pd_2_(dba)_3_ (2.5 mol%), IMes HCl (5 mol%)	19
4	Pd_2_(dba)_3_ (2.5 mol%), IPr HCl (5 mol%)	36
5	[Pd(allyl)Cl]_2_ (2.5 mol%), IPr HCl (5 mol%)	48
6	SPhos Pd G4 (5 mol%)	58
7	Cs_2_CO_3_ instead of ^*t*^BuOLi	82
8	K_2_CO_3_ instead of ^*t*^BuOLi	24
9	DBU instead of ^*t*^BuOLi	n.d.
10	45 °C	64
11	Dioxane instead of THF	84
12	Toluene instead of THF	78
13	No base	n.d.
14	No catalyst	n.d.
15	Under air	69
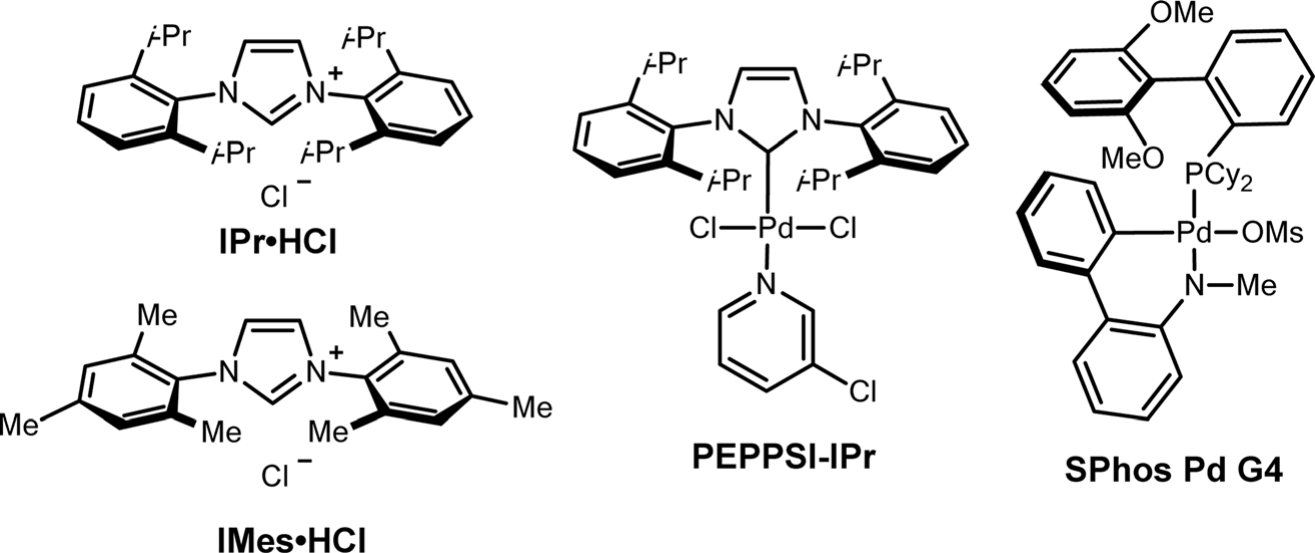		

a Standard conditions: benzaldehyde hydrazone, 1a (0.28 mmol, 1.25 equiv), vinyl epoxide, 2a (0.23 mmol), PEPPSI-IPr (5 mol%), and ^*t*^BuOLi (0.23 mmol, 1 equiv) in 1.5 mL THF for 24 h at room temperature under N_2_. Yields were determined by ^1^H NMR with 1,3,5-trimethoxybenzene as an internal standard.

### Substrate scope

**Fig. 2:**
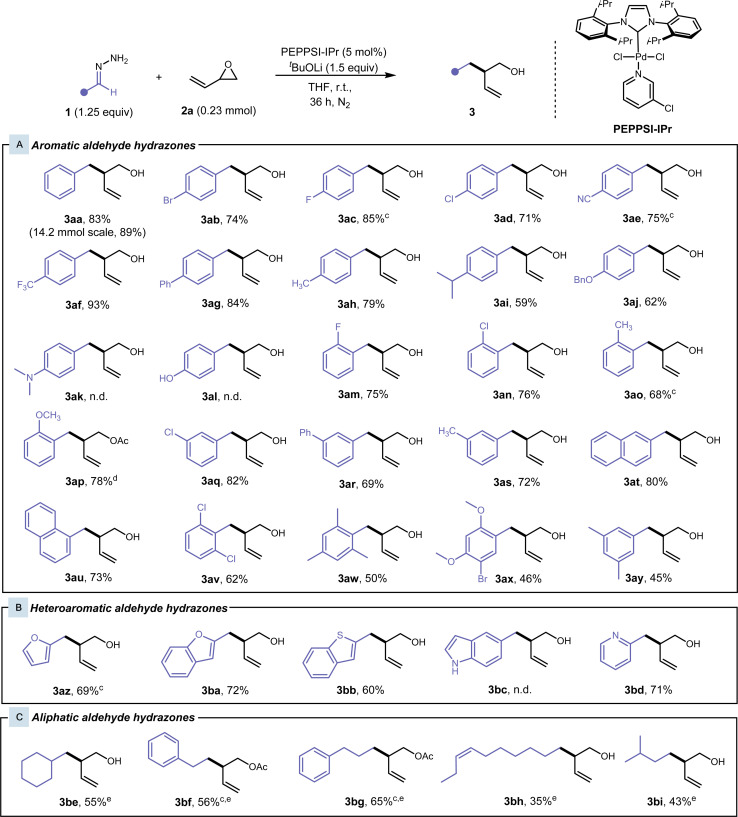
Aldehyde hydrazone substrate scope.^a,b^ (a) Reaction conditions: Hydrazone 1 (0.28 mmol, 1.25 equiv), vinyl epoxide 2a (0.23 mmol), PEPPSI-IPr (5 mol%), and ^*t*^BuOLi (0.34 mmol, 1.5 equiv) in 1.5 mL THF at room temperature for 36 h under N_2_. (b) Yields of isolated products. (c) Hydrazone 1 (0.28 mmol, 1.25 equiv), vinyl epoxide 2a (0.23 mmol), PEPPSI-IPr (5 mol%), and Cs_2_CO_3_ (0.28 mmol, 1.25 equiv) in 1.5 mL THF at room temperature for 36 h under N_2_. (d) Acetylation conditions: crude compound (purified with a silica plug), Ac_2_O (500 μL), and pyridine (500 μL) at room temperature overnight. (e) Hydrazone 1 (0.45 mmol, 2 equiv), vinyl epoxide 2a (0.23 mmol), PEPPSI-IPr (5 mol%), FeCl_3_ (10 mol%) and ^*t*^BuOLi (0.34 mmol, 1.5 equiv), in 1.5 mL THF at 45 °C for 24 h under N_2_.

With our optimised conditions for the cross-coupling of hydrazone 1aa and vinyl epoxide 2a, we proceeded to our substrate scope. Various substituted benzaldehyde hydrazones were explored (Fig. 2A). Initially, we observed complete conversion with benzaldehyde hydrazone 1aa and 2a within 24 hours, but incomplete conversion for *para*-substituted benzaldehyde hydrazones and polyaromatic hydrazones was observed. Lithium *tert*-butoxide was increased to 1.5 equivalents and a reaction time of 36 h was employed to ensure complete conversion and to improve reproducibility of the procedure (See ESI Table S4†). In some cases, the product was unable to be separated from the *tert*-butanol side-product, therefore cesium carbonate (1.25 equiv) was used instead of lithium *tert*-butoxide. In general, hydrazones with various substituents and substitution patterns gave moderate to excellent yields. Aryl hydrazones containing halogens, heteroatoms, and polyaromatic systems worked well under our conditions and this reaction exhibited excellent functional group tolerance. *Para*-substituted benzaldehyde hydrazones were first investigated. Hydrazones bearing electron-withdrawing groups, such as fluoro-, bromo-, chloro-, trifluoromethyl-, cyano- and phenyl-, at this position gave desired products 3ab–3ag at high yields of 71–93%. With electron-donating groups in the *para*-position, in the case of methyl-, isopropyl-, and benzyloxy-benzaldehyde hydrazones, the yield of the desired product 3ah–3aj had slightly diminished (59–79%). This is due to an electronic effect, where the electron-donating group destabilises the carbanion formed at the benzylic position. In the case of 3ak, 3al, and 3bc, the strong electron-donating group did not favour the formation of the carbanion, and we did not observe any desired product. Only the aryl hydrazone starting material was observed and recovered. Electron-donating and electron-withdrawing *ortho*-substituted benzaldehyde hydrazones also performed well in these conditions, giving desired products 3am–3ap in 68–78% yield. Regarding 3ap, the pre-coordination of the methoxy-group onto the Pd catalyst could direct the hydrazone coordination to the Pd catalyst after deprotonation, leading to an increased yield compared to the other cases with electron-donating groups in the *ortho*-position. *Meta*-substituted benzaldehyde hydrazones were also investigated and gave desired products 3aq–3as in moderately high yields of 69–82%. 1- and 2-Naphthaldehyde hydrazones proved to be effective substrates, giving the desired products 3au and 3at in high yields of 80% and 73%, respectively. Multiply-substituted benzaldehyde hydrazones worked well to yield alcohols 3av–3ay in moderate yields of 45–62%, where the steric hindrance and electron-donating properties of the *ortho*-substituents hinder the yield. Heteroaryl aldehyde hydrazones were also well tolerated (Fig. 2B), and moderately high yields of 60–71% were achieved for branched homoallylic alcohols 3az–3bd.

We then shifted our focus to aliphatic aldehyde hydrazones (Fig. 2C), since generating carbanions from aliphatic aldehyde hydrazones presents a greater challenge. Without a neighbouring aromatic group, resonance stability of the carbanion is absent, and these hydrazones tend to react with themselves to form their inactive azine counterpart at an increased rate. Our initial optimised conditions showed low yields, and the reaction was reoptimized (See ESI Table S5† for more details). A Lewis acid catalyst (10 mol% FeCl_3_) was added to increase the reactivity of the vinyl epoxide, while the reaction was heated at 50 °C for 24 h to accelerate the reaction and to out-compete the azine side reaction. We were able to achieve the desired products 3be–3bi from cyclohexanecarboxaldehyde, 3-phenylpropionaldehyde, phenylacetaldehyde, *cis*-dec-7-enal, and isovaleraldehyde hydrazones in low to moderate yields of 35–65%, where the stability of the hydrazone mainly dictated the yield. Acetophenone-derived hydrazones were also subjected to the standard conditions and these modified conditions. However, only trace amounts of product were able to be detected by GCMS. This could be attributed to the added steric bulk about the benzylic position of the generated carbanion.

**Fig. 3:**
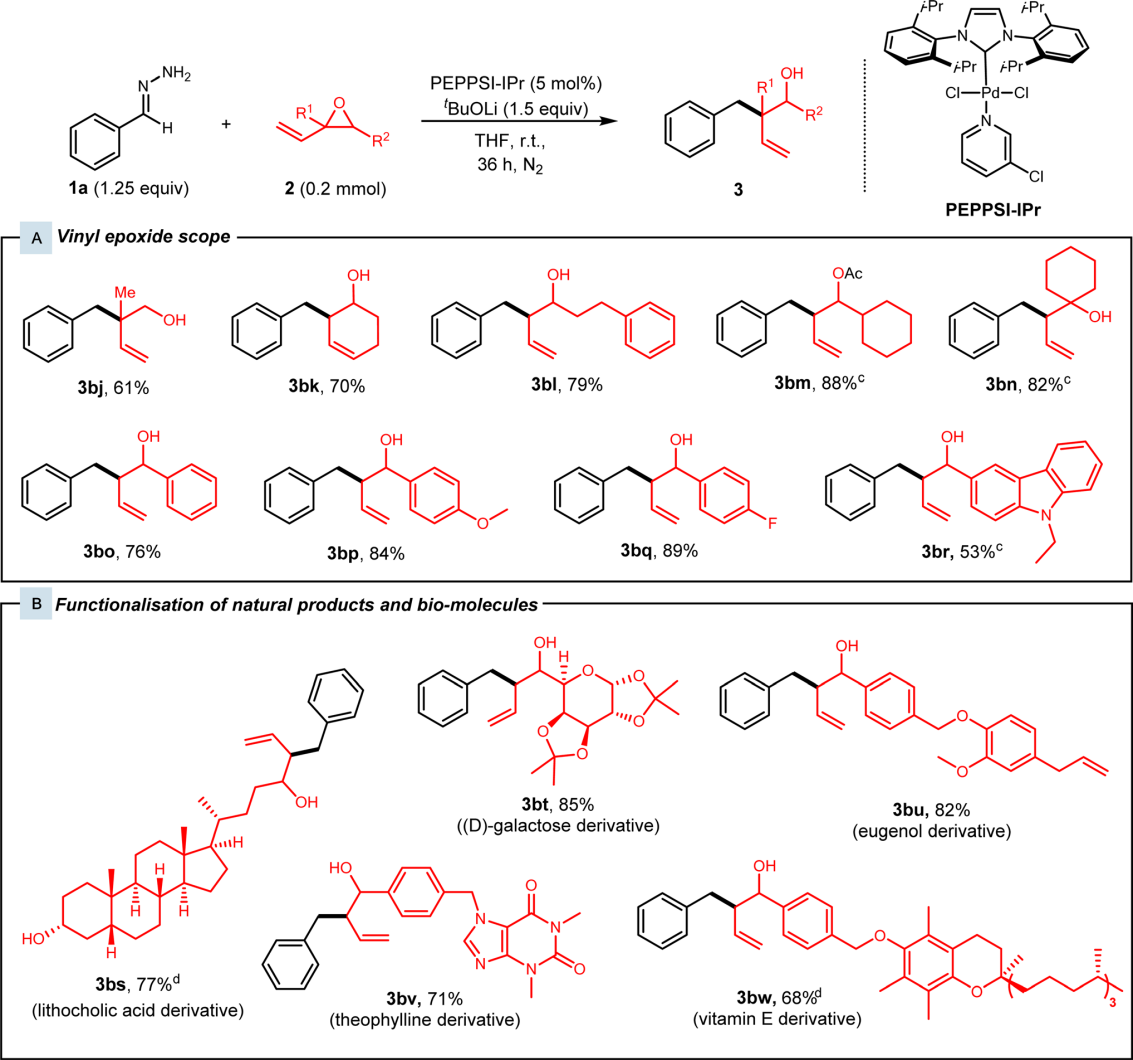
Vinyl epoxide substrate scope.^a,b^ (a) Reaction conditions: Hydrazone 1 (0.25 mmol, 1.25 equiv), vinyl epoxide 2 (0.2 mmol), PEPPSI-IPr (5 mol%), and ^*t*^BuOLi (0.38 mmol, 1.5 equiv) in 1.5 mL THF at room temperature for 36 h under N_2_. (b) Yields of isolated products. (c) Reaction conditions: Hydrazone 1 (0.20 mmol), vinyl epoxide 2 (0.25 mmol, 1.25 mmol), PEPPSI-IPr (5 mol%), and ^*t*^BuOLi (0.38 mmol, 1.5 equiv) in 1.5 mL THF at room temperature for 36 h under N_2_. (d) 0.15 mmol vinyl epoxide.

Subsequently, various substituted vinyl epoxides were then investigated (Fig. 3). Alkyl-substituted vinyl epoxides afforded the desired products 3bj–3bn in high yields of 61–88%. Next, benzaldehyde-derived vinyl epoxides present as a mixture of diastereomers were subjected to our protocol, and high yields of 76–89% were achieved for racemic alcohols 3bo–3bq. We also tested our conditions with a heteroaryl-substituted vinyl epoxide, which gave 3br in 53% yield.

With the high functional group tolerance and efficiency of this protocol, we then turned our attention to the late-stage derivatisation of natural products (Fig. 3B). Vinyl epoxides synthesised from various natural product derivatives were subjected to standard conditions. Lithocholic acid and (d)-galactose-derived vinyl epoxides were superior substrates as they afforded the branched homoallylic alcohols 3bs and 3bt in yields of 77% and 85% respectively. Alkaloids such as theophylline-derived vinyl epoxide 2m were tested with our conditions and alcohol 3bv was achieved with 71% yield. Lastly, eugenol and vitamin E-derived vinyl epoxides 2n and 2o reacted smoothly under our catalytic system and gave alcohols 3bu and 3bw in 82% and 68% yield.

### Mechanistic investigation

**Fig. 4:**
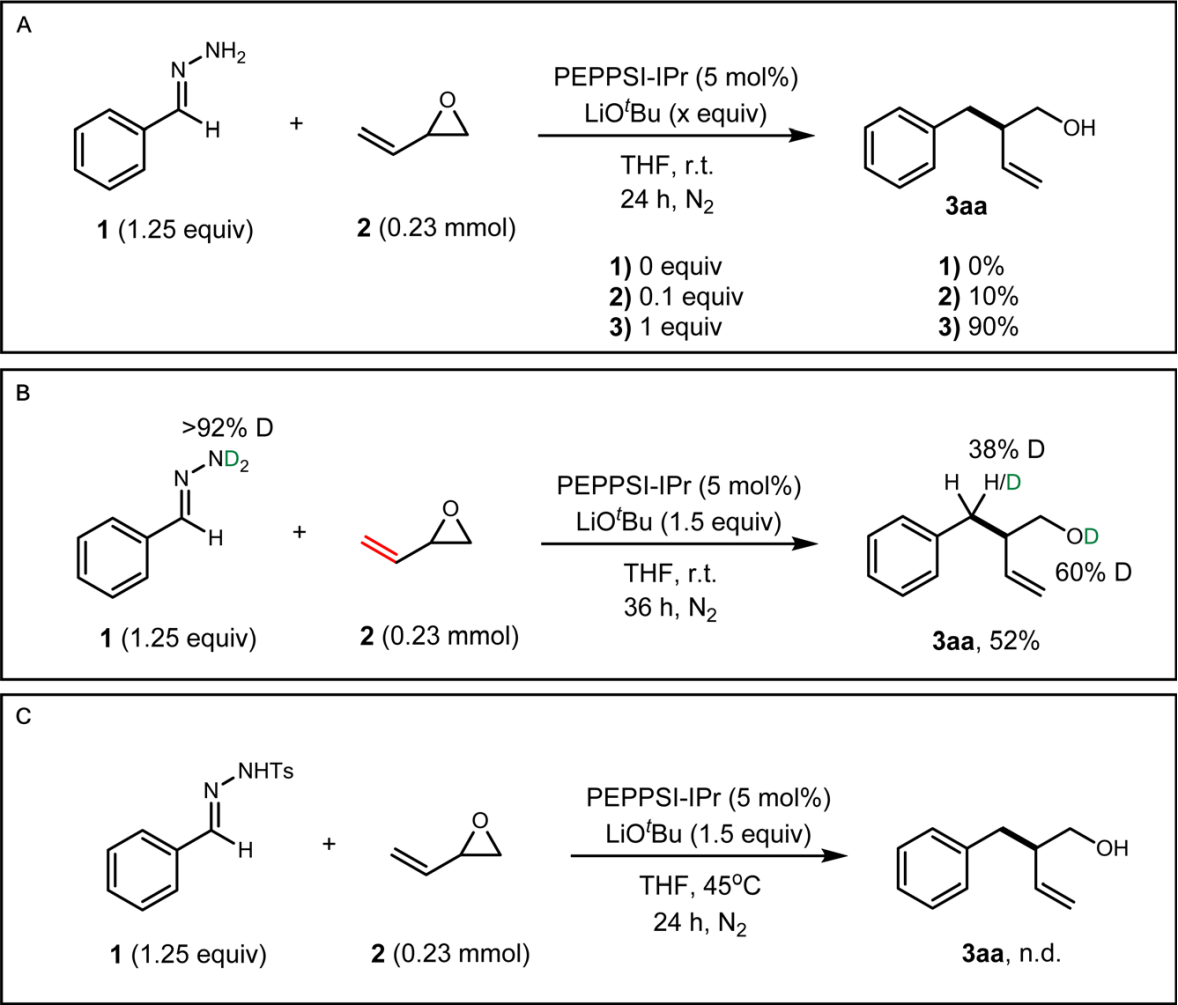
Mechanistic studies. (A) Base control studies. (B) Deuterium labelling studies. (C) Carbene mechanism probe experiment.

Several control experiments were performed to gain preliminary insight into the reaction mechanism (see the ESI† for more details). We first investigated the participation of the alkoxy-intermediate in our reaction. In previous literature, it was theorised that the alkoxy-intermediate deprotonates the nucleophile which directs the 1,2-addition onto the vinyl epoxide.^33^ In the absence of base, no reaction occurred between 1 and 2a (Fig. 4A). When 10 mol% base was employed, 3aa was only observed in 10% yield. These results suggest that the added base performs the initial deprotonation step to form a 2,3-diazaallyl anion. Otherwise, we would expect an adduct of the hydrazone and the vinyl epoxide if the alkoxy-intermediate performs the first deprotonation of the hydrazone. Subsequently, we performed deuterium isotope experiments. Deuterated benzaldehyde hydrazone 1 was subjected to our protocol and we observed deuteration of both the benzylic position (38% D) and the alcohol (60% D) of 3aa (Fig. 4B). Deuteration of the alcohol is evidence that the alkoxy-intermediate participates in one deprotonation step. We then subjected vinyl epoxide 2a with *N*-tosyl benzaldehyde hydrazone, a common carbene precursor. No reaction occurred, which deemed a carbene mechanism to be implausible and the reaction likely proceeds through a two-electron pathway (Fig. 4C). The high regioselectivity could be attributed to a 3,3′-elimination step analogous to diallylpalladium complexes in the work of Morken and coworkers (See ESI Fig. S2†).^34,35^

**Fig. 5:**
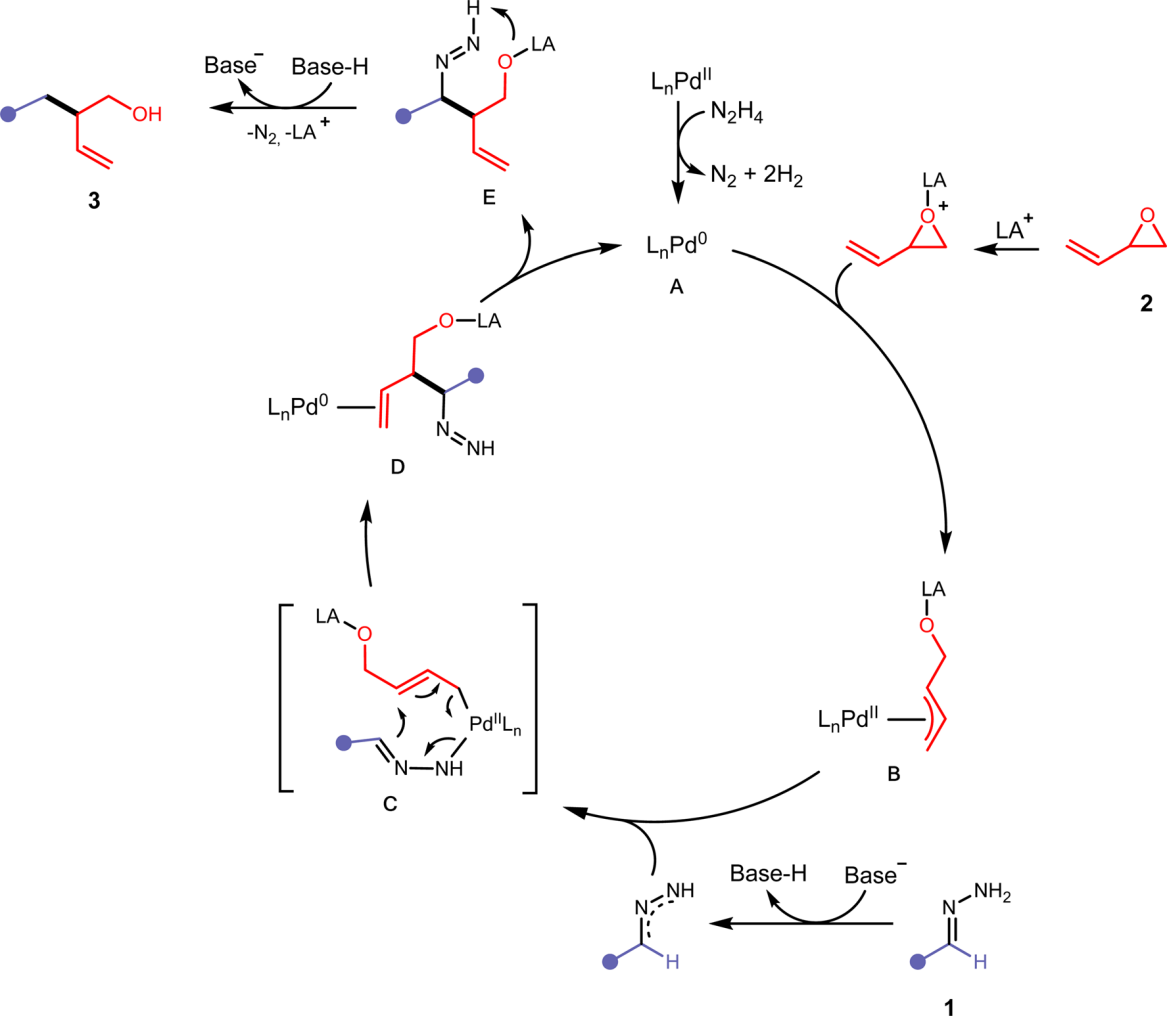
Proposed mechanism for the Pd-catalysed cross-coupling of hydrazones and vinyl epoxides.

Based on the previously described experimental observations, and preceding literature in similarly operating catalytic systems,^31,34–36^ we have proposed the following mechanism (Fig. 5). Initially, the Pd(ii) precatalyst is activated with residual hydrazine in the reaction mixture leading to the IPr-Pd(0) active catalyst A. Hydrazine has been shown to reduce Pd(ii) to Pd(0) efficiently.^37^A then undergoes oxidative addition with an activated vinyl epoxide 2 to form the π-allylpalladium complex B. Deprotonation of the hydrazone 1 with ^*t*^BuOLi would allow the formation of a 2,3-diazaallyl anion, which then coordinates to B and forms C. A 3,3′-elimination would occur, leading to D in a regioselective manner. After ligand dissociation to reform active catalyst A, intermediate E undergoes intramolecular deprotonation and releases nitrogen with the assistance of a protonated base to form compound 3.

## Conclusions

In summary, we have developed a regioselective and high-yielding Pd-catalysed method for the synthesis of 2-alkyl-β-γ-unsaturated alcohols utilising vinyl epoxides and Umpolung hydrazones. Highlights of this methodology include the efficient transformation of various substituted aryl/alkyl aldehyde-derived hydrazones and substituted vinyl epoxides. A broad substrate scope, high functional group tolerance, and late-stage modification of complex, diverse molecules demonstrate its synthetic utility. HOME chemistry provides a sustainable route for the functionalisation of vinyl epoxides with the deployment of carbonyl precursors as mild carbanion sources, and the absence of alkyl halides or external organometallic reagents. Our laboratory is currently conducting further research on the mechanism and exploring an asymmetric variant of this reaction.

## Author contributions

EC and CJL conceptualised the idea of this work. EC designed and conducted experiments for the optimisation and scope, and wrote the manuscript. CJL provided general guidance, project direction and manuscript revision.

## Conflicts of interest

The authors declare no conflict of interest.

## Supplementary Material

SC-015-D4SC05411C-s001

## Data Availability

All experimental procedures, mechanistic investigations, and characterisation data of all synthesised compounds are available in the ESI.†

## References

[cit1] He J., Ling J., Chiu P. (2014). Vinyl Epoxides in Organic Synthesis. Chem. Rev..

[cit2] Murakami T., Shimizu T., Taguchi K. (2000). Synthesis of Sphingadienine-type Glucocerebrosides. Tetrahedron.

[cit3] Trost B. M., Dong G., Vance J. A. (2010). Cyclic 1,2-Diketones as Core Building Blocks: A Strategy for the Total Synthesis of (−)-Terpestacin. Chem.–Eur. J..

[cit4] Mangion I., Strotman N., Drahl M., Imbriglio J., Guidry E. (2009). Dynamic Kinetic Asymmetric Allylation of Hydrazines and Hydroxylamines. Org. Lett..

[cit5] Charrier N., Gravestock D., Zard S. Z. (2006). Radical Additions of Xanthates to Vinyl Epoxides and Related Derivatives: A Powerful Tool for the Modular Creation of Quaternary Centers. Angew. Chem., Int. Ed..

[cit6] Deng X.-M., Sun X.-L., Tang Y. (2005). Highly Regioselective Rearrangement of 2-Substituted Vinylepoxides Catalyzed by Gallium(III) Triflate. J. Org. Chem..

[cit7] Herr R. W., Johnson C. R. (1970). Comparison of the Reactions of Methylmagnesium, Methyllithium, and Methylcopper Reagents with 1,2-Epoxybutane and 3,4-Epoxy-1-butene. J. Am. Chem. Soc..

[cit8] Equey O., Vrancken E., Alexakis A. (2004). Regioselective SN2 Opening of Vinylic Epoxides with Trialkylzincates and Trialkylaluminates. Eur. J. Org. Chem..

[cit9] Smith A. B., Pitram S. M., Gaunt M. J., Kozmin S. A. (2002). Dithiane Additions to Vinyl Epoxides: Steric Control over the SN2 and SN2‘ Manifolds. J. Am. Chem. Soc..

[cit10] Restorp P., Somfai P. (2005). Regioselective and Divergent Opening of Vinyl Epoxides with Alkyne Nucleophiles. Eur. J. Org. Chem..

[cit11] Hübscher T., Helmchen G. (2006). Enantioselective Formal Synthesis of Brefeldin A and Analogues *via* Anionic Cyclization of an Alkenyl Epoxide. Synlett.

[cit12] Tsuji J., Takahashi H., Morikawa M. (1965). Organic Syntheses by Means of Noble Metal Compounds XVII. Reaction of π-Allylpalladium Chloride with Nucleophiles. Tetrahedron Lett..

[cit13] Trost B. M., Fullerton T. J. (1973). New Synthetic Reactions. Allylic Alkylation. J. Am. Chem. Soc..

[cit14] Trost B. M., Van Vranken D. L., Bingel C. (1992). A Modular Approach for Ligand Design for Asymmetric Allylic Alkylations *via* Enantioselective Palladium-Catalyzed Ionizations. J. Am. Chem. Soc..

[cit15] Trost B. M., Crawley M. L. (2003). Asymmetric Transition-Metal-Catalyzed Allylic Alkylations: Applications in Total Synthesis. Chem. Rev..

[cit16] Trost B. M., Van Vranken D. L. (1996). Asymmetric Transition Metal-Catalyzed Allylic Alkylations. Chem. Rev..

[cit17] Pàmies O., Margalef J., Cañellas S., James J., Judge E., Guiry P. J., Moberg C., Bäckvall J.-E., Pfaltz A., Pericàs M. A., Diéguez M. (2021). Recent Advances in Enantioselective Pd-Catalyzed Allylic Substitution: From Design to Applications. Chem. Rev..

[cit18] Trost B. M., Molander G. A. (1981). Neutral Alkylations *via* Palladium(0) Catalysis. J. Am. Chem. Soc..

[cit19] Echavarren A. M., Tueting D. R., Stille J. K. (1988). Palladium-Catalyzed Coupling of Vinyl Epoxides with Organostannanes. J. Am. Chem. Soc..

[cit20] Pineschi M., Del Moro F., Crotti P., Di Bussolo V., Macchia F. (2004). Catalytic Regiodivergent Kinetic Resolution of Allylic Epoxides: A New Entry to Allylic and Homoallylic Alcohols with High Optical Purity. J. Org. Chem..

[cit21] Hata T., Bannai R., Otsuki M., Urabe H. (2010). Iron-Catalyzed Regio- and Stereoselective Substitution of γ,δ-Epoxy-α,β-unsaturated Esters and Amides with Grignard Reagents. Org. Lett..

[cit22] Mori T., Nakamura T., Kimura M. (2011). Stereoselective Coupling Reaction of Dimethylzinc and Alkyne toward Nickelacycles. Org. Lett..

[cit23] Fox M. E., Lennona I. C., Farina V. (2007). Catalytic Asymmetric Synthesis of Ethyl (1R,2S)-Dehydrocoronamate. Tetrahedron Lett..

[cit24] Wu Y., Du C., Hu C., Li Y., Xie Z. (2011). Biomimetic Synthesis of Hyperolactones. J. Org. Chem..

[cit25] Doyle M. G. J., Gabbey A. L., McNutt W., Lundgren R. J. (2021). Enantioselective Tertiary Electrophile (Hetero)Benzylation: Pd-Catalyzed Substitution of Isoprene Monoxide with Arylacetates. Angew. Chem., Int. Ed..

[cit26] Nicolaou K. C., Wu T. R., Sarlah D., Shaw D. M., Rowcliffe E., Burton D. R. (2008). Total Synthesis, Revised Structure, and Biological Evaluation of Biyouyanagin A and Analogues Thereof. J. Am. Chem. Soc..

[cit27] Isaka M., Srisanoh U., Veeranondha S., Choowong W., Lumyong S. (2009). Cytotoxic Eremophilane Sesquiterpenoids From the Saprobic Fungus Berkleasmium Nigroapicale BCC 8220. Tetrahedron.

[cit28] Dai X.-J., Li C.-C., Li C.-J. (2021). Carbonyl Umpolung as an Organometallic Reagent Surrogate. Chem. Soc. Rev..

[cit29] Li C.-J. (2023). HOME-Chemistry: Hydrazone as Organo-Metallic Equivalent. Pure Appl. Chem..

[cit30] Alexakis A., Vrancken E., Mangeney P. (2000). Effect of BF_3_·Et_2_O Reagent on the Base-Promoted Rearrangements of Epoxides Attached to Eight-Membered Rings. J. Chem. Soc., Perkin Trans..

[cit31] Zhu D., Lv L., Li C.-C., Ung S., Gao J., Li C.-J. (2018). Umpolung of Carbonyl Groups as Alkyl Organometallic Reagent Surrogates for Palladium-Catalyzed Allylic Alkylation. Angew. Chem., Int. Ed..

[cit32] Kan J., Chen Z., Qiu Z., Lv L., Li C., Li C.-J. (2022). Umpolung Carbonyls Enable Direct Allylation and Olefination of Carbohydrates. Sci. Adv..

[cit33] Trost B. M., Bunt R. C., Lemoine R. C., Calkins T. L. (2000). Dynamic Kinetic Asymmetric Transformation of Diene Monoepoxides: A Practical Asymmetric Synthesis of Vinylglycinol, Vigabatrin, and Ethambutol. J. Am. Chem. Soc..

[cit34] Zhang P., Brozek L. A., Morken J. P. (2010). Pd-Catalyzed Enantioselective Allyl–Allyl Cross-Coupling. J. Am. Chem. Soc..

[cit35] Brozek L. A., Ardolino M. J., Morken J. P. (2011). Diastereocontrol in Asymmetric Allyl–Allyl Cross-Coupling: Stereocontrolled Reaction of Prochiral Allylboronates with Prochiral Allyl Chlorides. J. Am. Chem. Soc..

[cit36] Lv L., Li C.-J. (2021). Palladium-Catalyzed Defluorinative Alkylation of gem-Difluorocyclopropanes: Switching Regioselectivity *via* Simple Hydrazones. Angew. Chem., Int. Ed..

[cit37] Wang Y., Shi Y.-F., Chen Y.-B., Wu L.-M. (2012). Hydrazine Reduction of Metal Ions to Porous Submicro-Structures of Ag, Pd, Cu, Ni, and Bi. J. Solid State Chem..

