# Supplementary material for: Palladium-catalysed Tsuji–Trost-type vinyl epoxide cross-coupling with umpolung hydrazones
Source: Chem Sci. 2024 Oct 16;15(45):18943–51. doi: 10.1039/d4sc05411c (PMC11500674; doi:10.1039/d4sc05411c)

Supplementary Information (SI) for Chemical Science.
This journal is © The Royal Society of Chemistry 2024

Supporting Information

Palladium-Catalysed Tsuji-Trost-Type Vinyl Epoxide Cross-Coupling with Umpolung Hydrazones

Evan F. W. Chen[†] and Chao-Jun Li[†]*

[†]Department of Chemistry, and FRQNT Centre for Green Chemistry and Catalysis, McGill University, 801 Sherbrooke Street West, Montreal, Quebec H3A 0B8, Canada.

Corresponding Author

*Chao-Jun Li (email: cj.li@mcgill.ca)

Table of Contents

General Procedures 3
General procedures for the synthesis of hydrazone or vinyl epoxide starting materials 3
Aromatic aldehyde hydrazone synthesis procedure 3
General procedure for Corey-Chaykovski epoxidation for synthesis of vinyl epoxides 4
Preparation of aldehyde natural product derivatives 5
General procedure for the synthesis of branched homoallylic alcohols: 6
Optimisation of reaction conditions 7
Preliminary mechanism studies for the Pd-catalysed cross-coupling of vinyl epoxides and hydrazones 10
Characterisation data for compounds 12
Characterisation data for compounds 2d-2o 12
Characterisation data for compounds 3aa-3br 17
References 35
NMR spectra collection for compounds 36

General Procedures

All reagents and solvents were purchased from commercial sources (Alfa, MilliporeSigma, TCI, Combi-Blocks and Ambeed) and used without further purification unless otherwise stated. ^{1}H, ^{13}C and ^{19}F NMR spectra were taken on a Bruker AV500 500 MHz spectrometer. Chemical shifts of ^{1}H NMR spectra were reported using residual solvent signal of $CDCl_3$ (δ = 7.26 ppm). Chemical shifts of ^{13}C NMR spectra were reported using residual solvent signal of $CDCl_3$ (δ = 77.16 ppm) as internal standard. The peak patterns are indicated as follows: s, singlet; d, doublet; dd, doublet of doublet; t, triplet; q, quartet; h, heptet; m, multiplet. The coupling constants, J, are reported in Hertz (Hz). Column chromatography was performed on silica gel (200-300 mesh) or with SepaFlash™ Column HP Ruby spherical silica pre-packed columns and visualised with ultraviolet light. ESI-MS was obtained from the maXis Impact™ QTOF mass spectrometer. All solvents were purified and dried by standard techniques or used directly from a solvent purification system.

All reactions were carried out in oven-dried Biotage or Chemglass 2.0–5.0 mL microwave reaction vials, covered by aluminium caps with PTFE-faced silicone septa, under an atmosphere of nitrogen or argon unless otherwise stated. All air and moisture sensitive catalysts, ligands, and reagents were stored and charged in MBRAUN UNI lab Pro Glove Box Workstation.

General procedures for the synthesis of hydrazone or vinyl epoxide starting materials

Aromatic aldehyde hydrazone synthesis procedure

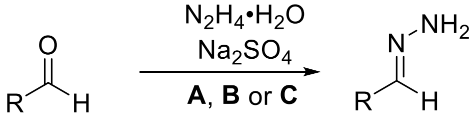

Hydrazones synthesized from their corresponding aldehydes were >95% pure by NMR and were used without further purification.

Method A:

To a 25 mL round-bottom flask, 0.5 mL of THF and hydrazine hydrate (64% aq. solution, 3.5 mmol, 1.3 equiv) was charged and stirred vigorously. In a pressure-equalising addition funnel, the aldehyde (2.4 mmol, 1 equiv) was dissolved in 10 mL of THF. The aldehyde was then added dropwise over 10 minutes to the hydrazine solution. Sodium sulphate was added once the addition of the aldehyde was complete, and the reaction stirred vigorously at room temperature for 2 hours. Afterwards, the mixture was transferred into a separatory funnel and diluted with H_2O (15 mL) and CH_2Cl_2 (15 mL). The organic layer was extracted and washed with brine (15 mL) and then dried with Na_2SO_4. The solvent was then removed under reduced pressure at room temperature and the hydrazone was isolated.

Method B (for polar aldehydes):

To a 25 mL round-bottom flask, 0.5 mL of MeOH and hydrazine hydrate (64% aq. solution) was charged and stirred vigorously. In a pressure-equalising addition funnel, the aldehyde was dissolved in 10 mL of MeOH. The aldehyde was then added dropwise over 10 minutes to the hydrazine solution. Sodium

sulphate was added once the addition of the aldehyde was complete, and the reaction stirred vigorously for 2 hours at room temperature. Afterwards, CH_2Cl_2 (5 mL) was added, and the reaction was filtered into a 50 mL round-bottom flask. The solvent was then removed under reduced pressure at room temperature and the hydrazone was isolated.

Method C (for aliphatic aldehydes):

To a 25 mL round-bottom flask, 0.5 mL of THF and hydrazine hydrate (64% aq. solution, 3.5 mmol, 1.3 equiv) was charged and stirred vigorously. In a pressure-equalising addition funnel, the aldehyde (2.4 mmol, 1 equiv) was dissolved in 10 mL of THF. The aldehyde was then added dropwise over 10 minutes to the hydrazine solution. Sodium sulphate was added once the addition of the aldehyde was complete, and the reaction stirred vigorously at room temperature for 1 hour. Afterwards, the solution was filtered, and the solvent was removed under reduced pressure at room temperature to yield the hydrazone.

General procedure for Corey-Chaykovsky epoxidation for synthesis of vinyl epoxides

This modified procedure was based off of previous literature.[1]

1-allyltetrahydrothiophenium bromide synthesis

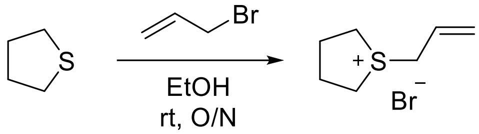

To a dry 100 mL round bottom flask, dry ethanol (30 mL) was added under argon. Tetrahydrothiophene (10.6 mL, 1.1 equiv) and allyl bromide (8.6 mL, 1 equiv) were then added in one addition. The reaction mixture was allowed to stir overnight, and the solvent was removed under reduced pressure to yield 1-allyltetrahydrothiophenium bromide as an off-white solid.

Caution – tetrahydrothiophene and the product have a strong unpleasant odour. The product is very hygroscopic, store under inert gas or in a desiccator.

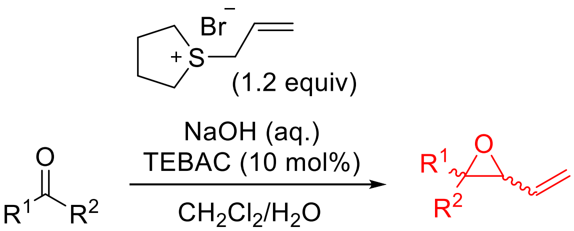

2

To a round bottom flask, aldehyde/ketone (4 mmol), benzyltriethylammonium chloride (0.4 mmol, 0.1 equiv), and 1-allyltetrahydrothiophenium bromide (4.8 mmol, 1.2 equiv) was added. CH_2Cl_2 (8 mL) was added, and the round bottom flask was cooled to 0 °C. The vessel was evacuated and purged with argon three times, being careful not to evaporate the CH_2Cl_2. Afterwards, 6.8 mL of 10 M NaOH which was chilled at 0°C, was added slowly via syringe. The reaction stirred at 0 °C for 1.5 h and was monitored by

TLC. Upon completion, the reaction was diluted with 10 mL of CH_2Cl_2 and 10 mL of H_2O. The organic layer was extracted, washed with brine, and dried over Na_2SO_4. The solvent was removed under reduced pressure to yield a crude oil, which was subjected to a silica gel plug immediately. The plug was packed with EtOAc/Et_3N (99:1), and then washed with hexanes/Et_3N (99:1). Afterwards, the crude oil was loaded directly onto the plug and flushed with hexanes/Et_3N (99:1, 50 mL) and then hexanes/EtOAc/Et_3N (ratio is compound specific, see compounds below). The second flush was collected, and the solvent was removed under reduced pressure to yield the pure vinyl epoxide.

Preparation of aldehyde natural product derivatives

Lithocholic acid derivative S3

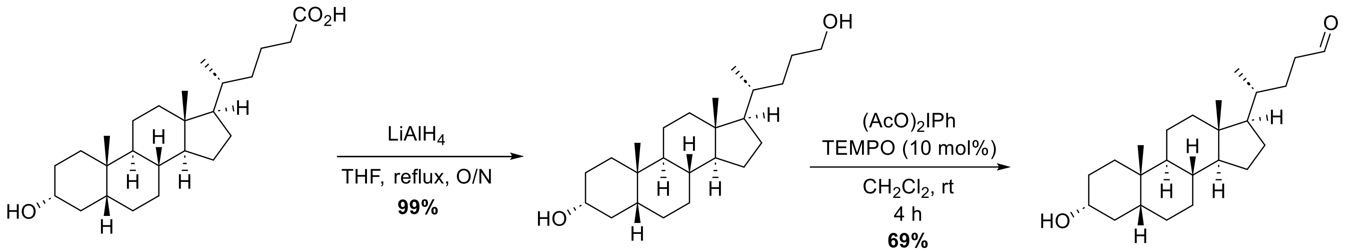

S1 Lithocholic acid

S2 was prepared from **S1** according to previous literature and all spectra matched with previous literature.[2]

The solution of lithocholic acid **S1** (1 g, 2.7 mmol) in dry THF (30 mL) was added dropwise to a suspension of powdered $LiAlH_4$ (304 mg, 8 mmol,) in dry THF (15 mL) at room temperature. The reaction mixture was refluxed and stirred overnight. To this mixture was added 60 mL water and 25 mL H_2SO_4 (1 M), stirring for 1 h. The mixture was extracted with ethyl acetate (3 × 50 mL). After drying with anhydrous Na_2SO_4 and evaporating the solvent, **S2** was obtained as a white solid in quantitative yield.

S3 was prepared from **S2** according to previous literature and all spectra matched with previous literature.[3]

To a solution of **S2** (212 mg, 0.6 mmol) in CH_2Cl_2, TEMPO (9 mg, 0.06 mmol, 0.1 equiv) was added followed by bis(acetoxy)iodobenzene (208 mg, 0.65 mmol, 1.1 equiv). The reaction was stirred at room temperature for 4 h and monitored by TLC (3:1 hexanes/ethyl acetate). Afterwards, saturated aqueous $Na_2S_2O_3$ (6 mL) was added to the mixture and the solution was extracted with CH_2Cl_2 (3 x 25 mL). The organic extract was then dried over Na_2SO_4 and then the solvent was evaporated under reduced pressure. The residue was purified by column chromatography on silica (3:1 petroleum ether/ethyl acetate) to afford **S3** as a white solid (69% yield).

(D)-galactose derivative

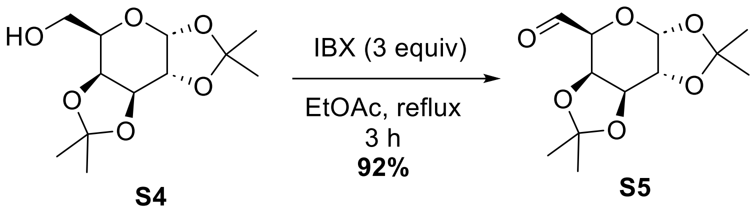

S5 was prepared from **S4** with method from previous literature [4] and all spectra matched with previous literature.[5]

To a solution of **S4** (500 mg, 1.9 mmol) in ethyl acetate, IBX (3 equiv) was added, and the solution was refluxed for 3 h. Afterwards, the precipitate was filtered and **S5** was obtained as a colourless oil (92% yield). Flush crude compound with silica plug with CH_2Cl_2 if compound is not pure after filtration.

General procedure for the synthesis of branched homoallylic alcohols:

General procedure A:

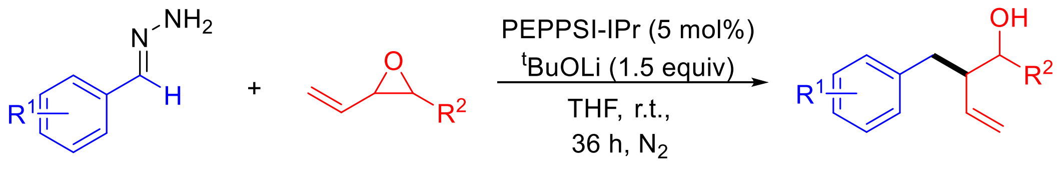

To a 2-5 mL oven-dried microwave vial with a stir bar, PEPPSI-IPr (7.7 mg, 0.011 mmol, 0.05 equiv), LiO^{t}Bu (27.0 mg, 0.34 mmol, 1.5 equiv) and vinyl epoxide (0.23 mmol, 1 equiv) was added inside a glovebox. 0.5 mL of dry THF was then added and the mixture was stirred for 10 min. Then, the hydrazone (0.28 mmol, 1.25 equiv) was added and an additional 1 mL of dry THF was added. The microwave vial was capped with an aluminium cap with a septum. Then, the vial was brought outside the glovebox and stirred for 36 hours at room temperature. Afterwards, the reaction mixture was filtered through a neutral alumina plug and washed with CH_2Cl_2, then ethyl acetate. The solvent was evaporated under reduced pressure and the remaining residue was purified with preparatory thin layer chromatography (5:1 petroleum ether/ethyl acetate) or flash chromatography (0-25% ethyl acetate/hexanes, gradient).

General procedure B:

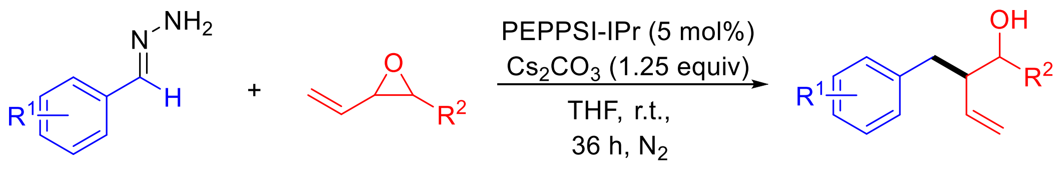

To a 2-5 mL oven-dried microwave vial with a stir bar, PEPPSI-IPr 7.7 (7.7 mg, 0.011 mmol, 0.05 equiv), Cs_2CO_3 (92 mg, 0.28 mmol, 1.25 equiv) and vinyl epoxide (0.23 mmol, 1 equiv) was added inside a

glovebox. 1 mL of dry THF was then added and the mixture was stirred for 10 min. Then, the hydrazone (0.28 mmol, 1.25 equiv) was added and an additional 1 mL of dry THF was added. The microwave vial was capped, and the vial was brought outside the glovebox and stirred for 36 hours at room temperature. Afterwards, the reaction mixture was separated into two 2 mL Eppendorf tubes and centrifuged using a micro-centrifuge. The supernatant was collected, and the salt pellet was washed with an additional 1.5 mL of CH_2Cl_2 and centrifuged again. The supernatant was collected once again and afterwards, the solvent was evaporated under reduced pressure. The residue was purified with preparatory thin layer chromatography (5:1 petroleum ether/ethyl acetate) or flash chromatography (0-25% ethyl acetate/hexanes, gradient).

General procedure C (for aliphatic aldehyde hydrazones)**:**

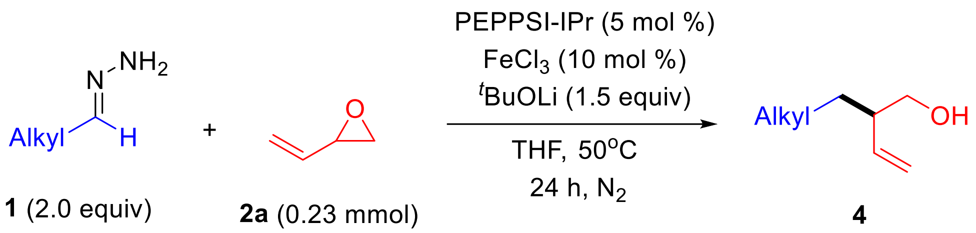

1 (2.0 equiv) **2a** (0.23 mmol) **4**

To a 2-5 mL oven-dried microwave vial with a stir bar, PEPPSI-IPr (7.7 mg, 0.011 mmol, 0.05 equiv), LiO^{t}Bu (27.0 mg, 0.34 mmol, 1.5 equiv) and vinyl epoxide (18 µL, 0.23 mmol, 1 equiv) was added inside a glovebox. 0.5 mL of dry THF was then added and the mixture was stirred for 10 min. Then, $FeCl_3$ (3.7 mg, 0.023 mmol, 10 mol%) and aliphatic hydrazone (0.46 mmol, 2.0 equiv) was added with an additional 1 mL of dry THF. The microwave vial was capped with an aluminium cap with a septum. Then, the vial was brought outside the glovebox and stirred for 24 hours at 50 °C. Afterwards, the reaction mixture was filtered through a neutral alumina plug and washed with CH_2Cl_2, then ethyl acetate. The solvent was evaporated under reduced pressure and the remaining residue was purified with flash chromatography (0-25% ethyl acetate/hexanes, gradient).

Optimisation of reaction conditions

Table S1. Optimization of pre-catalyst and ligand for Pd-catalysed cross-coupling of aryl aldehyde hydrazone and vinyl epoxide[a]

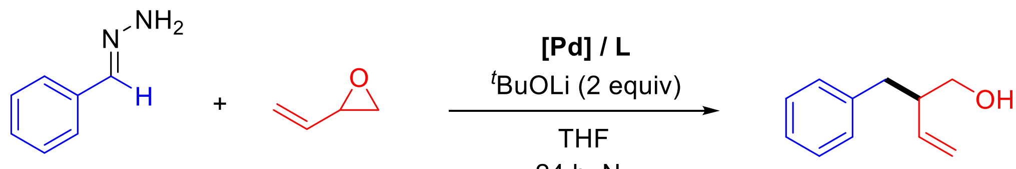

1a (1.25 equiv) **2a** (0.23 mmol) **3aa**

Entry	Pd catalyst, **[Pd]**	Ligand, **L**	Temperature (°C)	Yield (%)[b]
1	$Pd_2(dba)_3$ (5 mol%)	PPh_3 (10 mol%)	45	29
2	$Pd_2(dba)_3$ (5 mol%)	PPh_3 (10 mol%)	rt	0
3	$Pd_2(dba)_3$ (5 mol%)	^{i}Pr-PNP (5 mol%)	45	13
4	$Pd_2(dba)_3$ (5 mol%)	PCy_3 (10 mol%)	45	31

5	$Pd_2(dba)_3$ (5 mol%)	IMes•HCl (10 mol%)	45	32
6[b]	$Pd_2(dba)_3$ (2.5 mol%)	IMes•HCl (5 mol%)	rt	19
7[b]	$Pd_2(dba)_3$ (2.5 mol%)	IPr•HCl (5 mol%)	rt	36
8[b]	Pd(allyl)Cl_2 (2.5 mol%)	IPr•HCl (5 mol%)	rt	49
9[b]	$Pd(OAc)_2$ (5 mol%)	IPr•HCl (5 mol%)	rt	48
10[b]	SPhos Pd G4 (5 mol%)	-	rt	58
11	PEPPSI-IPr (10 mol%)	-	45	49
12[b]	PEPPSI-IPr (5 mol%)	-	rt	75
13	PEPPSI-SIPr	-	45	45

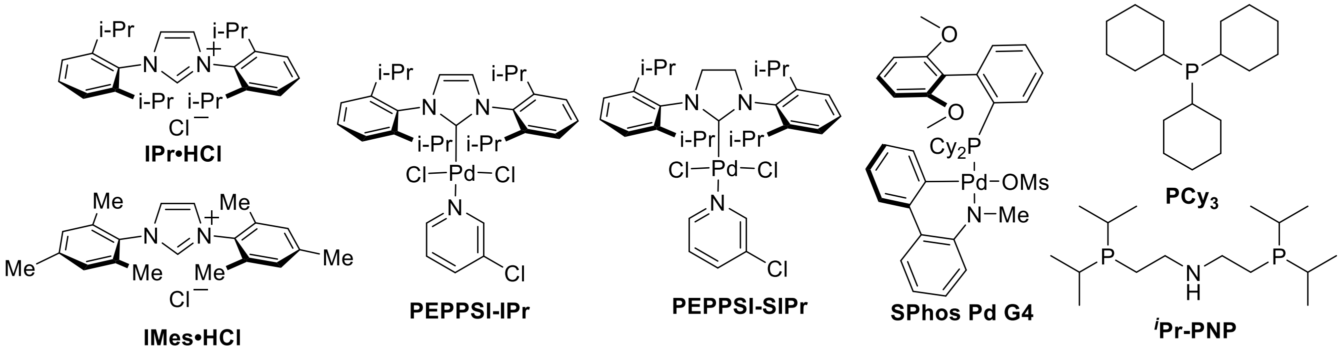

a) Reaction conditions: Hydrazone **1a** (0.28 mmol, 1.25 equiv), vinyl epoxide **2a** (0.23 mmol), Pd catalyst (10 mol%), ligand (monodentate, 20 mol%, ambidentate and NHC, 10 mol%) and ^{t}BuOLi (0.46 mmol, 2 equiv) in 1.5 mL THF at 45 °C for 24 h under N_2. b) 1 equiv LiO^{t}Bu.

Table S2. Optimization of base and equivalents of base.[a]

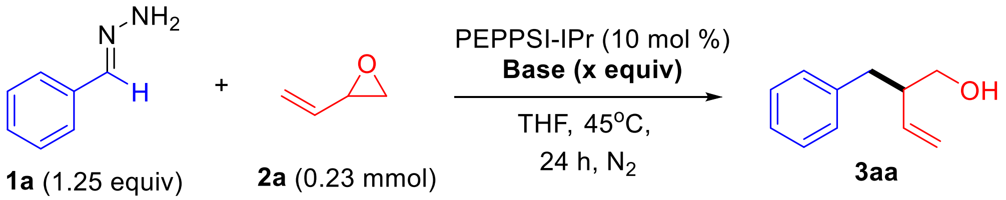

Entry	Base	Equivalent(s)	Yield (%)[b]
1	LiO^{t}Bu	2.0	49
2	LiO^{t}Bu	1.0	64
3[b]	LiO^{t}Bu	1.0	90
4	KO^{t}Bu	1.0	30
5	NaO^{t}Bu	1.0	19
6	Cs_2CO_3	1.0	54
7[b]	Cs_2CO_3	1.0	82
8	DBU	1.0	n.d.
9	TBD	1.0	n.d.
10	Li_2CO_3	1.0	n.d.
11	Li_3PO_4	1.0	n.d.
12	LiOH	1.0	n.d.
13[b]	K_2CO_3	1.0	24
14[b]	K_3PO_4	1.0	n.d.
15	$CaCO_3$	1.0	n.d.

a) Reaction conditions: Hydrazone **1a** (0.28 mmol, 1.25 equiv), vinyl epoxide **2a** (0.23 mmol), PEPPSI-IPr (10 mol%) and base in 1.5 mL THF at 45 °C for 24 h under N_2. b) Room temperature, 5 mol% PEPPSI-IPr.

Table S3. Optimisation of solvents.[a]

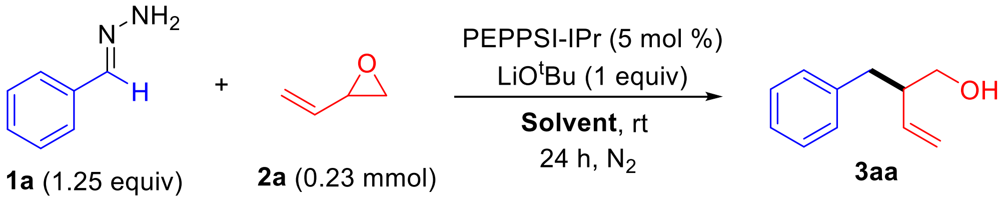

Entry	Variation from standard conditions	Yield (%)[b]
1	THF	90
2	1,4-Dioxane	84
3	DMF	71
4	Toluene	78
5	2-methyl-THF	62

a) Standard reaction conditions: Hydrazone **1a** (0.28 mmol, 1.25 equiv), vinyl epoxide **2a** (0.23 mmol), PEPPSI-IPr (5 mol%) ^{t}BuOLi (0.23 mmol, 1 equiv) in 1.5 mL solvent for 24 h under N_2.

Table S4. Optimisation of reaction conditions for electron deficient aromatic aldehyde hydrazones.

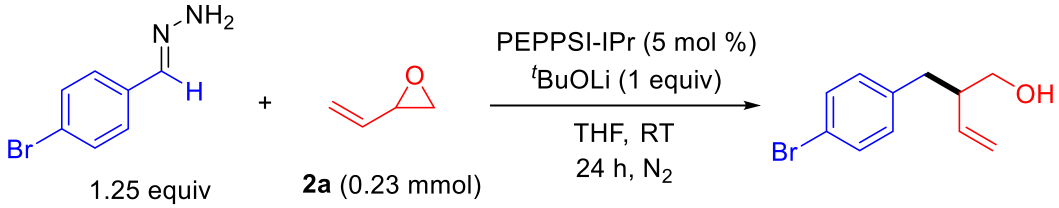

Entry	Variation from standard conditions	Yield (%)[b]
1	None	57
2	1.5 equiv LiO^{t}Bu	64
3	36 h reaction time	74
4	1.25 equiv Cs_2CO_3, 36 h reaction time	72

a) Standard reaction conditions: Hydrazone (0.28 mmol, 1.25 equiv), vinyl epoxide **2a** (0.23 mmol), PEPPSI-IPr (5 mol%), base in 1.5 mL solvent at room temperature for 36 h under N_2.

Table S5. Optimisation of reaction conditions for aliphatic aldehyde hydrazones.

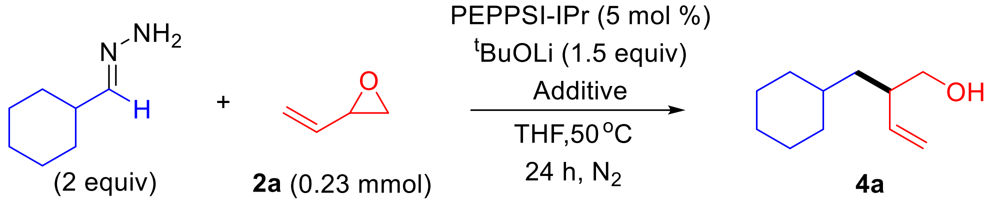

Entry	Variation from standard conditions	Yield (%)[b]
1	1.25 equiv hydrazone	21
2	Room temperature, 1.25 equiv hydrazone	12
3	None	33
4	10 mol% $Bi(OTf)_3$ additive	26
5	10 mol% $AlCl_3$ additive	14
6	10 mol% $In(OTf)_3$ additive	34
7	10 mol% $Sn(Me)_4$ additive	52
8	10 mol% $Sc(OTf)_3$ additive	54
9	10 mol% $FeCl_3$ additive	57

a) Standard reaction conditions: cyclohexanecarboxaldehyde hydrazone (0.46 mmol, 2.0 equiv), vinyl epoxide **2a** (0.23 mmol), PEPPSI-IPr (5 mol%), base in 1.5 mL solvent at 50 °C for 24 h under N_2.

Preliminary mechanism studies for the Pd-catalysed cross-coupling of vinyl epoxides and hydrazones

Table S6. Control experiments[a]

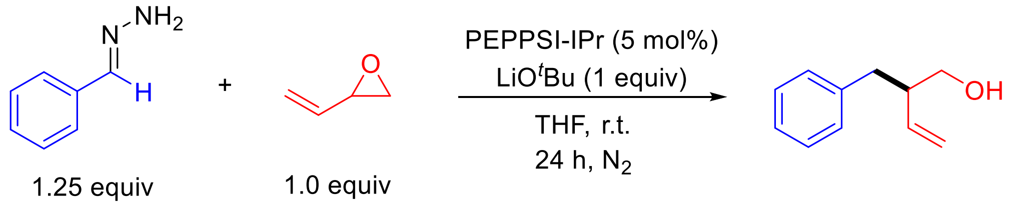

Entry	Variation from standard conditions	Yield (%)[b]
1	None	90
2	No PEPPSI-IPr	n.d.
3	IPr•HCl and no Pd	n.d.
4	$Pd_2(dba)_3$ no ligand added	n.d.
5	No base added	n.d.
6	No hydrazone added	n.d.
7	No vinyl epoxide added	n.d.
8	Under air	69
9	$FeCl_3$ and IPr•HCl, No Pd	n.d.

a) Standard reaction conditions: Hydrazone **1a** (0.28 mmol, 1.25 equiv), vinyl epoxide **2a** (0.23 mmol), PEPPSI-IPr (5 mol%) ^{t}BuOLi (0.23 mmol, 1 equiv) in 1.5 mL solvent for 24 h under N_2.

Initially, it was theorised that the alkoxide deprotonation of the nucleophile directs the nucleophilic attack. This leads to substitution of the more hindered branched position (Figure S1).[6] This theory has been disproven due to the requirement of external added base. If the alkoxy-allylpalladium intermediate performed the deprotonation of the hydrazone, the nucleophilic attack of the hydrazone onto the vinyl epoxide would be observed. However, we only observe unreacted starting materials, suggesting that the external base performs the first deprotonation of the hydrazone.

Figure S1. Alkoxy-directed nucleophilic attack pathway

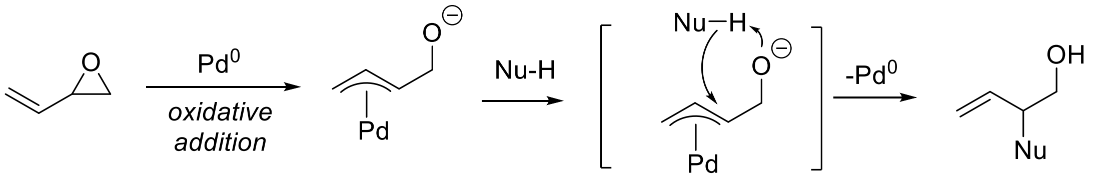

We propose that the branched selectivity arises from a 3,3'-elimination step of the diallylpalladium species. Analogous to the work by Morken and coworkers, [7, 8] and our past research on hydrazones and allylpalladium complexes,[9, 10] the origin of regioselectivity from this elimination step arises from steric interactions between the vinyl epoxide and the ligand. A linear orientation of the vinyl epoxide leads to a less sterically hindered transition state, which favours the formation of the branched homoallylic alcohol over the linear allylic alcohol product (Figure S2).

Figure S2. Regioselective vinyl epoxide opening with hydrazones.

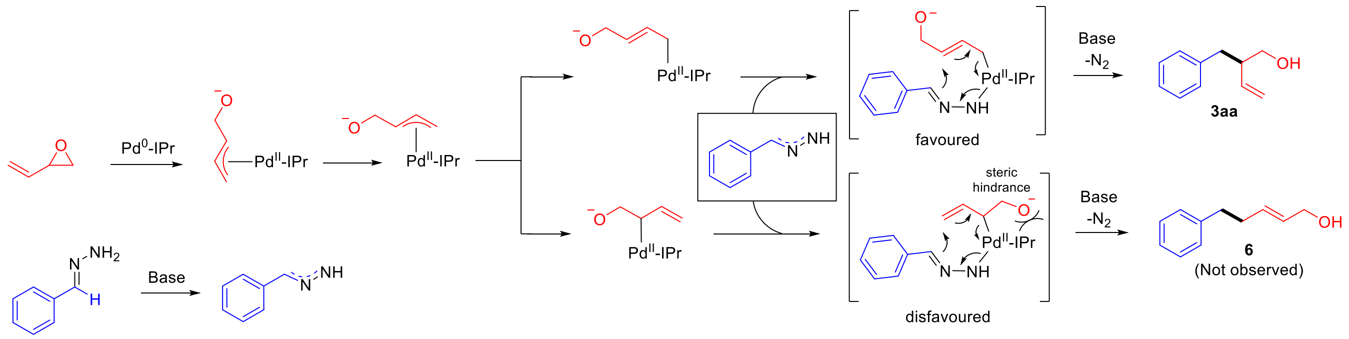

Characterisation data for compounds

Characterisation data for compounds 2d-2o

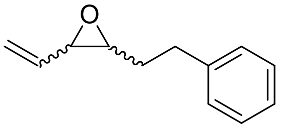

2-phenethyl-3-vinyloxirane (**2d**) was prepared according to the general procedure for Corey-Chaykovsky epoxidation of carbonyls. Silica plug was flushed with hexanes/EtOAc/Et_3N (90:9:1, 250 mL). Yield 79% (1:1.2 d.r. (cis/trans)), colourless oil.

^{1}H NMR (500 MHz, $CDCl_3$) δ 7.32 – 7.27 (m, 2H, cis/trans isomers), 7.22 – 7.18 (m, 3H, cis/trans isomers), 5.68 (ddd, J = 17.4, 10.6, 7.1 Hz, 0.5H, cis isomer), 5.55 (ddd, J = 17.6, 10.3, 7.5 Hz, 0.5H, trans isomer), 5.49 – 5.38 (m, 1H, cis/trans isomers), 5.36 – 5.30 (m, 0.5H, cis isomer), 5.27 – 5.21 (m, 0.5H, trans isomer), 3.42 (dd, J = 7.2, 4.3 Hz, 0.5H, cis isomer), 3.13 (ddd, J = 6.9, 5.8, 4.3 Hz, 0.5H, cis isomer), 3.06 (dd, J = 7.6, 2.2 Hz, 0.5H, trans isomer), 2.87 (dd, J = 5.7, 2.2 Hz, 0.5H, trans isomer), 2.85 – 2.79 (m, 1H, cis/trans isomers), 2.79 – 2.69 (m, 1H, cis/trans isomers), 1.98 – 1.76 (m, 2H, cis/trans isomers) (mixture of diastereomers).

^{13}C{^{1}H} NMR (126 MHz, $CDCl_3$) δ 141.3, 135.8, 132.4, 128.6, 128.6, 128.6, 128.5, 126.2, 120.5, 119.2, 59.9, 59.0, 58.2, 57.4, 34.0, 32.7, 32.3, 29.7 (mixture of diastereomers).

Spectra matched those previously reported.[1]

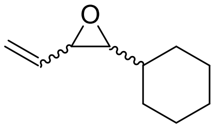

2-cyclohexyl-3-vinyloxirane (**2e**) was prepared according to the general procedure for Corey-Chaykovsky epoxidation of carbonyls. Silica plug was flushed with hexanes/EtOAc/Et_3N (90:9:1, 250 mL). Crude yield 42% (1:2 d.r.), colourless oil.

Compound **2e** was not able to be fully characterised due to its high volatility and mixture with hexanes. ^{1}H NMR showed evidence of vinyl epoxide.

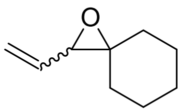

2-vinyl-1-oxaspiro[2.5]octane (**2f**) was prepared according to the general procedure for Corey-Chaykovsky epoxidation of carbonyls, reaction was allowed to stir at room temperature overnight for complete conversion. Silica plug was flushed with hexanes/EtOAc/Et_3N (90:9:1, 250 mL) Yield 44%, colourless oil.

^{1}H NMR (500 MHz, $CDCl_3$) δ 5.79 (ddd, J = 17.5, 10.6, 7.3 Hz, 1H), 5.51 – 5.43 (m, 1H), 5.38 – 5.30 (m, 1H), 3.21 (d, J = 7.2 Hz, 1H), 1.78 – 1.66 (m, 2H), 1.62 – 1.42 (m, 8H).

^{13}C{^{1}H} NMR (126 MHz, $CDCl_3$) δ 133.4, 120.0, 64.9, 64.5, 35.6, 29.5, 25.7, 25.2, 24.9.

Spectra matched those previously reported.[11]

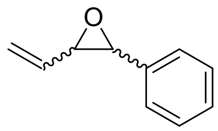

2-phenyl-3-vinyloxirane (**2g**) was prepared according to the general procedure for Corey-Chaykovsky epoxidation of carbonyls. Silica plug was flushed with hexanes/EtOAc/Et_3N (75:24:1, 250 mL) Yield 76% (1:1 d.r. (cis/trans)), colourless oil.

^{1}H NMR (500 MHz, $CDCl_3$) δ 7.40 – 7.31 (m, 5H, cis/trans isomers), 5.74 (ddd, J = 17.6, 10.4, 7.5 Hz, 0.5H, trans isomer), 5.58 – 5.50 (m, 1H, cis/trans isomers), 5.40 (ddd, J = 17.1, 10.4, 8.3 Hz, 0.5H, cis isomer), 5.35 (dd, J = 10.3, 1.2 Hz, 0.5H, trans isomer), 5.32 – 5.25 (m, 0.5H, cis isomer), 4.25 (d, J = 4.2 Hz, 0.5H, cis isomer), 3.77 (d, J = 2.0 Hz, 0.5H, trans isomer), 3.67 (dd, J = 8.3, 4.3 Hz, 0.5H, cis isomer), 3.37 (dd, J = 7.5, 2.0 Hz, 0.5H, trans isomer) (mixture of diastereomers).

^{13}C{^{1}H} NMR (126 MHz, $CDCl_3$) δ 137.2, 135.3, 135.2, 132.2, 128.7, 128.4, 128.3, 127.9, 126.6, 125.6, 122.1, 119.7, 63.1, 60.4, 59.9, 59.0 (mixture of diastereomers).

Spectra matched those previously reported.[12]

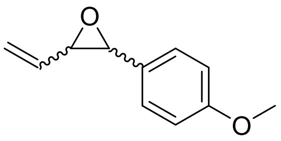

2-(4-methoxyphenyl)-3-vinyloxirane (**2h**) was prepared according to the general procedure for Corey-Chaykovsky epoxidation of carbonyls. Silica plug was flushed with hexanes/EtOAc/Et_3N (75:24:1, 250 mL) Yield 82% (1:1.2 d.r. (cis/trans)), colourless oil.

^{1}H NMR (500 MHz, $CDCl_3$) δ 7.26 (d, J = 8.8 Hz, 0.9H, cis isomer), 7.21 (d, J = 8.7 Hz, 1.1H, trans isomer), 6.89 (d, J = 8.7 Hz, 2H, cis/trans isomers), 5.73 (ddd, J = 17.6, 10.4, 7.4 Hz, 0.5H, trans isomer), 5.58 – 5.49 (m, 1H, cis/trans isomers), 5.41 (ddd, J = 17.2, 10.3, 8.2 Hz, 0.5H, cis isomer), 5.33 (dd, J = 10.4, 1.3 Hz, 0.5H, trans isomer), 5.28 (dd, J = 10.3, 1.7 Hz, 0.5H, cis isomer), 4.20 (d, J = 4.2 Hz, 0.5H, cis isomer), 3.81 (s, 3H, cis/trans isomers), 3.72 (d, J = 2.0 Hz, 0.5H, trans isomer), 3.63 (dd, J = 8.2, 4.2 Hz, 0.5H, cis isomer), 3.36 (dd, J = 7.4, 2.0 Hz, 0.5H, trans isomer) (mixture of diastereomers).

^{13}C{^{1}H} NMR (126 MHz, $CDCl_3$) 159.9, 159.3, 135.4, 132.4, 129.1, 127.8, 127.2, 126.9, 121.9, 119.5, 114.1, 113.8, 62.9, 60.3, 60.0, 58.8, 55.5, 55.4 (mixture of diastereomers).

Spectra matched those previously reported.[12]

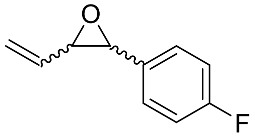

2-(4-methoxyphenyl)-3-vinyloxirane (2i) was prepared according to the general procedure for Corey-Chaykovsky epoxidation of carbonyls. Silica plug was flushed with hexanes/EtOAc/Et_3N (75:24:1, 250 mL) Yield 75% (1:1.2 d.r. (cis/trans)), colourless oil.

^{1}H NMR (500 MHz, $CDCl_3$) δ 7.34 – 7.28 (m, 0.9H, cis isomer), 7.27 – 7.23 (m, 1.1H, trans isomer), 7.04 (td, $^3J_{CF}$ = 8.7 Hz, J = 1.7 Hz, 2H, cis/trans isomers) 5.72 (ddd, J = 17.1, 10.5, 7.4 Hz, 0.5H, trans isomer), 5.58 – 5.49 (m, 1H, cis/trans isomers), 5.41 – 5.25 (m, 1.5H, cis/trans isomers), 4.21 (d, J = 4.2 Hz, 0.5H, cis isomer), 3.75 (d, J = 1.9 Hz, 0.5H, trans isomer), 3.65 (dd, J = 7.9, 4.2 Hz, 0.5H, cis isomer), 3.32 (dd, J = 7.4, 2.0 Hz, 0.5H, trans isomer) (mixture of diastereomers)

^{13}C{^{1}H} NMR (126 MHz, $CDCl_3$) δ 163.83 (d, $^1J_{CF}$ = 245.9 Hz), 162.49 (d, $^1J_{CF}$ = 246.0 Hz), 134.98, 132.90 (d, $^4J_{CF}$ = 3.0 Hz), 131.89, 130.98 (d, $^4J_{CF}$ = 3.2 Hz), 128.21 (d, $^3J_{CF}$ = 8.2 Hz), 127.29 (d, $^3J_{CF}$ = 8.4 Hz), 122.21, 119.89, 115.63 (d, $^2J_{CF}$ = 21.9 Hz), 115.28 (d, $^2J_{CF}$ = 21.7 Hz), 63.02, 59.86, 59.78, 58.42. (mixture of diastereomers).

Spectra matched those previously reported.[13]

^{19}F NMR (471 MHz, $CDCl_3$) δ -113.77, -114.46 (mixture of diastereomers)

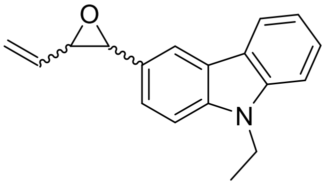

9-ethyl-3-(3-vinyloxiran-2-yl)-9H-carbazole (2j) was prepared according to the general procedure for Corey-Chaykovsky epoxidation of carbonyls. Silica plug was flushed with hexanes/EtOAc/Et_3N (50:49:1, 250 mL), crude was a mixture of epoxide and unreacted aldehyde. Compound is not stable in room temperature (colour changes yellow to dark orange) and was used immediately after silica plug.

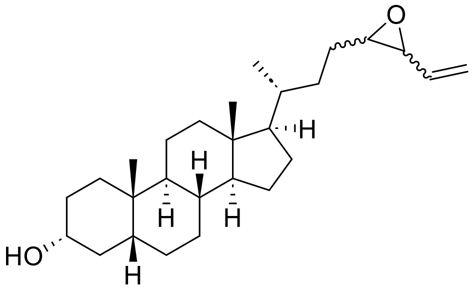

(3R,5R,8R,9S,10S,13R,14S,17R)-10,13-dimethyl-17-((2R)-4-(3-vinyloxiran-2-yl)butan-2-yl)hexadecahydro-1H-cyclopenta[a]phenanthren-3-ol (2k) was prepared according to the general

procedure for Corey-Chaykovsky epoxidation of carbonyls. Silica plug was flushed with hexanes/EtOAc/Et_3N (75:24:1 250 mL) Yield 96% (1:1.2 d.r. (cis/trans)), Yellow oil.

^{1}H NMR (500 MHz, $CDCl_3$) δ 5.71 (ddd, J = 17.5, 10.5, 7.3 Hz, 0.5H, cis isomer), 5.57 (ddd, J = 17.5, 10.2, 7.5 Hz, 0.5H, trans isomer), 5.51 – 5.40 (m, 1H, cis/trans isomers), 5.35 (dd, J = 10.5, 1.6 Hz, 0.5H, cis isomer), 5.24 (dd, J = 10.2, 1.5 Hz, 0.5H, trans isomer), 3.61 (tt, J = 10.7, 4.7 Hz, 1H, cis/trans isomers), 3.39 (td, J = 7.0, 4.2 Hz, 0.5H, cis isomer), 3.12 – 3.00 (m, 1H, cis/trans isomers), 2.79 (ddd, J = 6.3, 4.8, 2.1 Hz, 0.5H, trans isomer), 1.95 (dt, J = 12.2, 3.1 Hz, 1H, cis/trans isomer), 1.91 – 1.69 (m, 4H, cis/trans isomers), 1.69 – 0.84 (m, 30H, cis/trans isomers), 0.64 (s, 3H, cis/trans isomers) (mixture of diastereomers).

^{13}C{^{1}H} NMR (126 MHz, $CDCl_3$) δ 136.1, 132.8, 132.8, 120.5, 120.4, 119.0, 72.0, 61.1, 60.9, 59.3, 59.2, 59.0, 58.8, 57.5, 57.3, 56.6, 56.6, 56.3, 56.2, 56.1, 42.8, 42.2, 40.6, 40.3, 40.3, 36.6, 36.0, 35.8, 35.7, 35.6, 35.5, 35.5, 34.7, 32.4, 32.2, 32.1, 31.8, 30.7, 28.9, 28.7, 28.4, 27.3, 26.6, 24.7, 24.4, 24.3, 23.5, 21.0, 18.8, 18.6, 12.2 (mixture of diastereomers)

HRMS (ESI) m/z calculated for $C_{27}H_{44}O_2$([M+Na]$^+$): 423.3234, found: 423.3227.

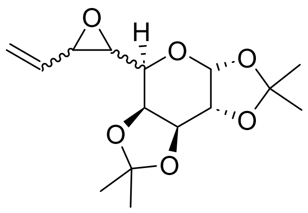

(3aR,5aS,8aS,8bR)-2,2,7,7-tetramethyl-5-(3-vinyloxiran-2-yl)tetrahydro-5H-bis([1,3]dioxolo)[4,5-b:4',5'-d]pyran (2l) was prepared according to the general procedure for Corey-Chaykovsky epoxidation of carbonyls. Silica plug was flushed with hexanes/EtOAc/Et_3N (70:29:1, 250 mL) Yield 73% (1:1.2 d.r.), yellow oil.

^{1}H NMR (500 MHz, $CDCl_3$) δ 5.83 (ddd, J = 17.2, 10.6, 5.8 Hz, 0.5H), 5.60 (ddd, J = 17.4, 10.2, 7.3 Hz, 0.5H), 5.55 – 5.44 (m, 2H), 5.41 – 5.33 (m, 1H), 5.28 (dd, J = 10.1, 1.6 Hz, 0.5H), 4.62 (t, J = 2.5 Hz, 0.5H), 4.61 (t, J = 2.4 Hz, 0.5H), 4.35 (t, J = 1.9 Hz, 0.5H), 4.33 (t, J = 1.9 Hz, 0.5H), 4.31 (dd, J = 4.9, 2.5 Hz, 0.5H), 4.29 (dd, J = 4.9, 2.3 Hz, 0.5H), 3.58 – 3.54 (m, 1H), 3.51 (dd, J = 6.3, 2.0 Hz, 0.5H), 3.40 (dd, J = 8.0, 4.1 Hz, 0.5H), 3.32 (dd, J = 7.3, 2.1 Hz, 0.5H), 3.15 (dd, J = 6.3, 2.1 Hz, 0.5H), 1.49 (s, 1.5H), 1.48 (s, 1.5H), 1.47 (s, 1.5H), 1.46 (s, 1.5H), 1.37 (s, 1.5H), 1.36 (s, 1.5H), 1.32 (s, 1.5H), 1.30 (s, 1.5H) (mixture of diastereomers).

^{13}C{^{1}H} NMR (126 MHz, $CDCl_3$) δ 134.8, 131.5, 120.7, 119.9, 109.7, 109.6, 108.8, 108.7, 96.3, 96.2, 71.7, 71.4, 70.6, 70.6, 70.6, 68.5, 65.0, 58.4, 57.8, 57.1, 56.1, 26.2, 26.1, 26.1, 26.1, 25.0, 25.0, 24.5, 24.4 (mixture of diastereomers).

HRMS (ESI) m/z calculated for $C_{15}H_{22}O_6$([M+Na]$^+$): 321.1309 found: 321.1303.

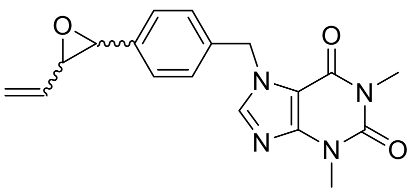

1,3-dimethyl-7-(4-(3-vinyloxiran-2-yl)benzyl)-3,7-dihydro-1H-purine-2,6-dione (**2m**) was prepared according to the general procedure for Corey-Chaykovsky epoxidation of carbonyls. Silica plug was flushed with $CHCl_3/Et_3N$ (99:1, 500 mL) Yield 92% (40:60 d.r. (cis/trans)), yellow solid.

^{1}H NMR (500 MHz, $CDCl_3$) δ 7.57 (s, 0.6H, trans isomer), 7.56 (s, 0.4H, cis isomer), 7.35 – 7.25 (m, 4H, cis/trans isomers), 5.70 (ddd, J = 17.6, 10.4, 7.4 Hz, 0.6H, trans isomer), 5.56 – 5.45 (m, 3H, cis/trans isomers), 5.40 – 5.23 (m, 1.4H, cis/trans isomers), 4.21 (d, J = 4.2 Hz, 0.4H, cis isomer), 3.75 (d, J = 1.9 Hz, 0.6H, trans isomer), 3.66 (dd, J = 7.9, 4.2 Hz, 0.4H, cis isomer), 3.58 (s, 1.2H, cis isomer), 3.58 (s, 1.8H, trans isomer), 3.40 (s, 1.2H, cis isomer), 3.39 (s, 1.8H, trans isomer), 3.31 (dd, J = 7.4, 1.9 Hz, 0.6H trans isomer) (mixture of diastereomers).

^{13}C{^{1}H} NMR (126 MHz, $CDCl_3$) δ 155.4, 155.4, 151.8, 149.1, 149.0, 140.9, 140.9, 137.8, 135.9, 135.5, 135.0, 134.9, 131.8, 128.3, 127.9, 127.4, 126.4, 122.3, 120.0, 107.1, 63.1, 59.9, 59.9, 58.6, 50.1, 50.1, 29.9, 28.2 (mixture of diastereomers).

HRMS (ESI) m/z calculated for $C_{18}H_{18}N_4O_3$([M+Na]$^+$): 361.1271, found: 361.1268.

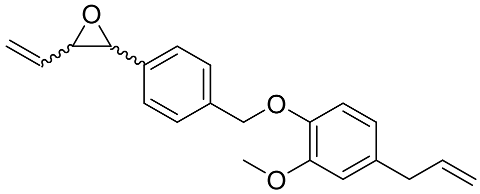

2-(4-((4-allyl-2-methoxyphenoxy)methyl)phenyl)-3-vinyloxirane (**2n**) was prepared according to the general procedure for Corey-Chaykovsky epoxidation of carbonyls. Silica plug was flushed with hexanes/EtOAc/Et_3N (80:19:1, 250 mL) Yield 94% (1:1.9 d.r. (cis/trans)), light yellow oil.

^{1}H NMR (500 MHz, $CDCl_3$) δ 7.45 – 7.41 (m, 2H, cis/trans isomers), 7.33 (d, J = 8.1 Hz, 0.8H, cis isomer), 7.28 (d, J = 8.2 Hz, 1.2H, trans isomer), 6.83 (d, J = 8.1 Hz, 0.4H, cis isomer), 6.79 (d, J = 8.1 Hz, 0.6H, trans isomer), 6.75 – 6.73 (m, 1H, cis/trans isomers), 6.69 – 6.67 (m, 1H, cis/trans isomers), 5.96 (ddt, J = 16.9, 10.3, 6.7 Hz, 1H, cis/trans isomers), 5.73 (ddd, J = 17.5, 10.4, 7.4 Hz, 0.6H, trans isomer), 5.58 – 5.49 (m, 1H, cis/trans isomers), 5.40 (ddd, J = 17.2, 10.4, 8.3 Hz, 0.4H, cis isomer), 5.34 (dd, J = 10.5, 1.2 Hz, 0.6H, trans isomer), 5.28 (dd, J = 10.4, 1.6 Hz, 0.4H, cis isomer), 5.13 (s, 1.2H, trans isomer), 5.11 (s, 0.8H, cis isomer), 5.11 – 5.02 (m, 2H, cis/trans isomers), 4.24 (d, J = 4.2 Hz, 0.4H, cis isomer), 3.88 (s, 3H, cis/trans isomers), 3.77 (d, J = 1.9 Hz, 0.6H, trans isomer), 3.66 (dd, J = 8.2, 4.3 Hz, 0.4H, cis isomer), 3.38 – 3.30 (m, 2.6H, cis/trans isomers) (mixture of diastereomers).

^{13}C{^{1}H} NMR (126 MHz, $CDCl_3$) δ 149.8, 149.7, 146.6, 146.5, 137.7, 137.7, 137.1, 136.7, 135.2, 134.8, 133.6, 133.5, 132.2, 127.5, 127.3, 126.7, 125.8, 122.1, 120.5, 119.7, 115.8, 114.5, 114.4, 112.6, 112.5, 71.2, 71.0, 63.0, 60.2, 60.0, 58.9, 56.1, 40.0, 39.9 (mixture of diastereomers).

HRMS (ESI) m/z calculated for $C_{21}H_{22}O_3$([M+Na]$^+$): 345.1461, found: 345.1452.

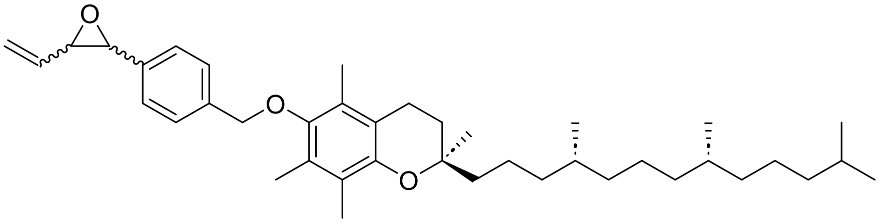

(2R)-2,5,7,8-tetramethyl-2-((4R,8R)-4,8,12-trimethyltridecyl)-6-((4-(3-vinyloxiran-2-yl)benzyl)oxy)chromane (2o) was prepared according to the general procedure for Corey-Chaykovsky epoxidation of carbonyls. Silica plug was flushed with hexanes/EtOAc/Et_3N (80:19:1, 250 mL) Yield 62% (1:1.5 d.r. (cis/trans)), light yellow oil.

^{1}H NMR (500 MHz, $CDCl_3$) δ 7.52 – 7.46 (m, 2H, cis/trans isomers), 7.37 (d, J = 8.0 Hz, 0.8H, cis isomer), 7.32 (d, J = 8.2 Hz, 1.2H, trans isomer), 5.75 (ddd, J = 17.7, 10.4, 7.5 Hz, 0.6H, trans isomer), 5.61 – 5.51 (m, 1H, cis/trans isomers), 5.43 (ddd, J = 17.1, 10.4, 8.2 Hz, 0.4H, cis isomer), 5.36 (dd, J = 10.4, 1.3 Hz, 0.6H, trans isomer), 5.29 (dd, J = 10.4, 1.7 Hz, 0.4H, cis isomer), 4.69 (s, 2H, cis/trans isomers), 4.28 (d, J = 4.2 Hz, 0.4H, cis isomer), 3.80 (d, J = 2.0 Hz, 0.6H, trans isomer), 3.68 (dd, J = 8.2, 4.3 Hz, 0.4H, cis isomer), 3.37 (dd, J = 7.4, 2.0 Hz, 0.6H), 2.59 (t, J = 6.9 Hz, 2H, cis/trans isomers), 2.22 (s, 1.2H, cis isomer), 2.21 (s, 1.8H, trans isomer), 2.17 (s, 1.2H, cis isomer), 2.16 (s, 1.8H, trans isomer), 2.11 (s, 3H, cis/trans isomers), 1.89 – 1.73 (m, 2H), 1.65 – 1.03 (m, 24H, cis/trans isomers), 0.89 – 0.83 (m, 12H, cis/trans isomers).

^{13}C{^{1}H} NMR (126 MHz, $CDCl_3$) δ 148.2, 148.2, 148.1, 138.4, 137.7, 136.7, 135.2, 134.8, 132.2, 128.0, 128.0, 128.0, 127.8, 126.7, 126.1, 126.1, 125.7, 123.1, 122.1, 119.7, 117.8, 75.0, 74.6, 74.5, 63.1, 60.3, 60.0, 58.9, 40.2, 40.2, 39.5, 37.7, 37.7, 37.6, 37.6, 37.6, 37.6, 37.5, 37.5, 37.5, 37.4, 32.9, 32.9, 32.9, 32.8, 31.5, 31.4, 28.1, 25.0, 25.0, 24.6, 24.0, 22.9, 22.8, 21.2, 20.8, 19.9, 19.8, 19.8, 19.8, 19.8, 13.0, 12.1, 12.0.

Mixture of diastereomers

HRMS (ESI) m/z calculated for $C_{40}H_{60}O_3$([M+Na]$^+$): 612.4435, found: 612.4461.

Characterisation data for compounds 3aa-3br

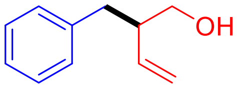

2-benzylbut-3-en-1-ol (3aa) was prepared according to General Procedure A. Yield 83%, light yellow oil.

^{1}H NMR (500 MHz, $CDCl_3$) δ 7.33 – 7.24 (m, 2H), 7.24 – 7.16 (m, 3H), 5.72 (ddd, *J* = 17.3, 10.4, 8.2 Hz, 1H), 5.18 – 5.04 (m, 2H), 3.62 (dd, *J* = 10.7, 5.0 Hz, 1H), 3.49 (dd, *J* = 10.7, 7.3 Hz, 1H), 2.77 (dd, *J* = 13.6, 7.0 Hz, 1H), 2.67 (dd, *J* = 13.6, 7.6 Hz, 1H), 2.62 – 2.51 (m, 1H), 1.65 (s, 1H).

^{13}C{^{1}H} NMR (126 MHz, $CDCl_3$) δ 139.8, 139.3, 129.2, 128.4, 126.1, 117.4, 64.9, 48.1, 37.4.

HRMS (ESI) m/z calculated for $C_{11}H_{14}O$([M+Na]$^+$): 185.0937, found: 185.0935.

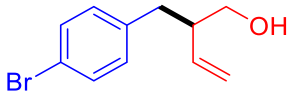

2-(4-bromobenzyl)but-3-en-1-ol (3ab) was prepared according to General Procedure A. Yield 74%, light yellow oil.

^{1}H NMR (500 MHz, $CDCl_3$) δ 7.39 (d, J = 8.4 Hz, 2H), 7.04 (d, J = 8.4 Hz, 2H), 5.66 (ddd, J = 17.2, 10.4, 8.2 Hz, 1H), 5.16 – 5.09 (m, 1H), 5.09 – 5.02 (m, 1H), 3.59 (dd, J = 10.7, 5.0 Hz, 1H), 3.48 (dd, J = 10.7, 7.1 Hz, 1H), 2.73 (dd, J = 13.6, 6.5 Hz, 1H), 2.59 (dd, J = 13.6, 7.9 Hz, 1H), 2.55 – 2.45 (m, 1H), 1.57 (s, 1H).

^{13}C{^{1}H} NMR (126 MHz, $CDCl_3$) δ 138.8, 138.8, 131.4, 131.0, 119.9, 117.8, 64.8, 48.0, 36.7.

HRMS (ESI) m/z calculated for $C_{11}H_{13}BrO([M+Na]^+)$: 263.0042, found: 263.0033.

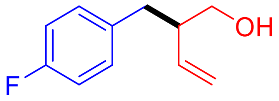

2-(4-fluorobenzyl)but-3-en-1-ol (3ac) was prepared according to General Procedure B. Yield 85%, light yellow oil.

^{1}H NMR (500 MHz, $CDCl_3$) δ 7.16 – 7.07 (m, 2H), 7.00 – 6.92 (m, 2H), 5.68 (ddd, J = 17.2, 10.4, 8.2 Hz, 1H), 5.14 (ddd, J = 10.4, 1.6, 0.7 Hz, 1H), 5.07 (ddd, J = 17.3, 1.7, 1.0 Hz, 1H), 3.64 – 3.56 (m, 1H), 3.48 (dd, J = 10.2, 7.8 Hz, 1H), 2.74 (dd, J = 13.7, 6.7 Hz, 1H), 2.62 (dd, J = 13.7, 7.7 Hz, 1H), 2.56 – 2.45 (m, 1H), 1.43 (s, 1H).

^{13}C{^{1}H} NMR (126 MHz, $CDCl_3$) δ 161.4 (d, $^1J_{CF}$ = 243.8 Hz), 138.9, 135.3 (d, $^4J_{CF}$ = 3.2 Hz), 130.5 (d, $^3J_{CF}$ = 7.8 Hz), 117.6 115.0 (d, $^2J_{CF}$ = 21.1 Hz), 64.7, 48.2, 36.4.

^{19}F NMR (471 MHz, $CDCl_3$) δ -117.37.

HRMS (ESI) m/z calculated for $C_{11}H_{13}FO([M+Na]^+)$: 203.0843, found: 203.0833.

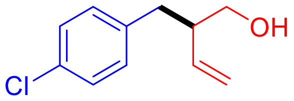

2-(4-chlorobenzyl)but-3-en-1-ol (3ad) was prepared according to General Procedure A. Yield 71%, light yellow oil.

^{1}H NMR (500 MHz, $CDCl_3$) δ 7.24 (d, J = 8.4 Hz, 2H), 7.09 (d, J = 8.4 Hz, 2H), 5.67 (ddd, J = 17.2, 10.3, 8.2 Hz, 1H), 5.13 (ddd, J = 10.4, 1.6, 0.7 Hz, 1H), 5.06 (ddd, J = 17.2, 1.6, 1.0 Hz, 1H), 3.59 (dd, J = 10.5, 5.1 Hz, 1H), 3.48 (dd, J = 10.5, 5.1 Hz, 1H), 2.75 (dd, J = 13.7, 6.6 Hz, 1H), 2.61 (dd, J = 13.7, 7.9 Hz, 1H), 2.56 – 2.46 (m, 1H), 1.54 (s, 1H).

^{13}C{^{1}H} NMR (126 MHz, $CDCl_3$) δ 138.8, 138.3, 131.9, 130.6, 128.5, 117.8, 64.8, 48.1, 36.7.

HRMS (ESI) m/z calculated for $C_{11}H_{13}ClO([M+Na]^+)$: 219.0547, found: 219.0551.

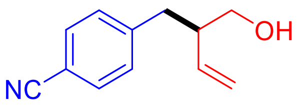

4-(2-(hydroxymethyl)but-3-en-1-yl)benzonitrile (3ae) was prepared according to General Procedure B. Yield 75%, light yellow oil.

^{1}H NMR (500 MHz, $CDCl_3$) δ 7.59 (d, J = 8.2 Hz, 2H), 7.29 (d, J = 8.1 Hz, 2H), 5.68 (ddd, J = 17.2, 10.4, 8.2 Hz, 1H), 5.20 – 5.12 (m, 1H), 5.11 – 5.03 (m, 1H), 3.62 (dd, J = 10.7, 5.2 Hz, 1H), 3.54 (dd, J = 10.6, 6.9 Hz, 1H), 2.90 (dd, J = 13.6, 6.3 Hz, 1H), 2.70 (dd, J = 13.6, 8.3 Hz, 1H), 2.62 – 2.51 (m, 1H), 1.52 (s, 1H).

^{13}C{^{1}H} NMR (126 MHz, $CDCl_3$) δ 145.7, 138.2, 132.2, 130.1, 119.2, 118.2, 110.1, 64.8, 47.9, 37.4.

HRMS (ESI) m/z calculated for $C_{12}H_{13}NO([M+Na]^+)$: 210.0889, found: 210.0888.

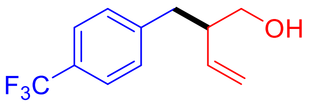

2-(4-(trifluoromethyl)benzyl)but-3-en-1-ol (3af) was prepared according to General Procedure A. Yield 93%, yellow oil.

^{1}H NMR (500 MHz, $CDCl_3$) δ 7.55 (d, J = 7.9 Hz, 2H), 7.30 (d, J = 8.0 Hz, 2H), 5.70 (ddd, J = 17.2, 10.4, 8.1 Hz, 1H), 5.20 – 5.14 (m, 1H), 5.14 – 5.06 (m, 1H), 3.63 (dd, J = 10.6, 5.1 Hz, 1H), 3.53 (dd, J = 10.7, 7.1 Hz, 1H), 2.88 (dd, J = 13.6, 6.5 Hz, 1H), 2.72 (dd, J = 13.6, 8.0 Hz, 1H), 2.64 – 2.53 (m, 1H), 1.50 (s, 1H).

^{13}C{^{1}H} NMR (126 MHz, $CDCl_3$) δ 144.03, 138.56, 129.61, 128.58 (q, $^2J_{CF}$ = 33.1 Hz), 125.33 (q, $^3J_{CF}$ = 3.7 Hz), 124.5 (q, $^1J_{CF}$ = 233.1 Hz) 118.04, 64.84, 47.97, 37.15.

^{19}F NMR (471 MHz, $CDCl_3$) δ -62.3.

HRMS (ESI) m/z calculated for $C_{12}H_{13}F_3O([M+Na]^+)$: 253.0811, found: 253.0808.

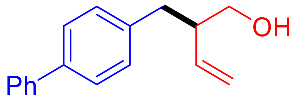

2-([1,1'-biphenyl]-4-ylmethyl)but-3-en-1-ol (3ag) was prepared according to General Procedure A. Yield 84%, white solid.

^{1}H NMR (500 MHz, $CDCl_3$) δ 7.63 – 7.57 (m, 2H), 7.54 (d, J = 7.8 Hz, 2H), 7.45 (t, J = 7.6 Hz, 2H), 7.35 (t, J = 7.6 Hz, 1H), 7.26 (d, J = 7.9 Hz, 2H), 5.76 (ddd, J = 17.4, 10.4, 8.1 Hz, 1H), 5.24 – 5.17 (m, 1H), 5.17 – 5.10 (m, 1H), 3.66 (dd, J = 10.7, 4.9 Hz, 1H), 3.54 (dd, J = 10.7, 7.3 Hz, 1H), 2.82 (dd, J = 13.7, 7.0 Hz, 1H), 2.72 (dd, J = 13.7, 7.5 Hz, 1H), 2.66 – 2.56 (m, 1H), 1.58 (s, 1H).

$^{13}C\{^1H\}$ **NMR** (126 MHz, $CDCl_3$) δ 141.1, 139.2, 139.1, 138.9, 129.7, 128.8, 127.2, 127.1, 127.1, 117.6, 64.9, 48.1, 37.0.

HRMS (ESI) m/z calculated for $C_{17}H_{18}O([M+Na]^+)$: 261.1250, found: 261.1258.

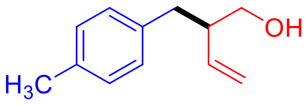

2-(4-methylbenzyl)but-3-en-1-ol (3ah) was prepared according to General Procedure A. Yield 79%, yellow oil.

^1H **NMR** (500 MHz, $CDCl_3$) δ 7.09 (d, J = 8.1 Hz, 2H), 7.06 (d, J = 8.1 Hz, 2H), 5.71 (ddd, J = 17.2, 10.4, 8.1 Hz, 1H), 5.17 – 5.06 (m, 2H), 3.68 – 3.57 (m, 1H), 3.47 (dd, J = 10.7, 7.5 Hz, 1H), 2.71 (dd, J = 13.6, 7.1 Hz, 1H), 2.63 (dd, J = 13.7, 7.4 Hz, 1H), 2.59 – 2.48 (m, 1H), 2.32 (s, 3H), 1.39 (s, 1H).

$^{13}C\{^1H\}$ **NMR** (126 MHz, $CDCl_3$) δ 139.4, 136.7, 135.7, 129.1, 129.1, 117.5, 65.0, 48.2, 37.0, 21.2.

HRMS (ESI) m/z calculated for $C_{12}H_{16}O([M+Na]^+)$: 199.1077, found: 199.1088.

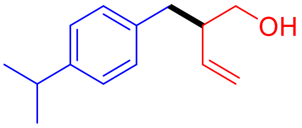

2-(4-isopropylbenzyl)but-3-en-1-ol (3ai) was prepared according to General Procedure A. Yield 59%, yellow oil.

^1H **NMR** (500 MHz, $CDCl_3$) δ 7.14 (d, J = 8.1 Hz, 2H), 7.10 (d, J = 8.1 Hz, 2H), 5.73 (ddd, J = 17.2, 10.4, 8.1 Hz, 1H), 5.18 – 5.08 (m, 2H), 3.61 (dd, J = 10.7, 4.8 Hz, 1H), 3.47 (dd, J = 10.7, 7.5 Hz, 1H), 2.88 (hept, J = 6.9 Hz, 1H), 2.70 (dd, J = 13.7, 7.4 Hz, 1H), 2.65 (dd, J = 13.7, 7.1 Hz, 1H), 2.60 – 2.49 (m, 1H), 1.42 (s, 1H), 1.24 (d, J = 6.9 Hz, 6H).

$^{13}C\{^1H\}$ **NMR** (126 MHz, $CDCl_3$) δ 146.7, 139.5, 137.0, 129.2, 126.5, 117.4, 64.9, 48.1, 37.0, 33.8, 24.2.

HRMS (ESI) m/z calculated for $C_{14}H_{20}O([M+Na]^+)$: 227.1406, found: 227.1399.

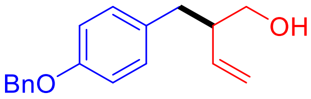

2-(4-(benzyloxy)benzyl)but-3-en-1-ol (3aj) was prepared according to General Procedure A. Yield 62%, white solid.

^1H **NMR** (500 MHz, $CDCl_3$) δ 7.48 – 7.42 (m, 2H), 7.41 – 7.36 (m, 2H), 7.35 – 7.30 (m, 1H), 7.09 (d, J = 8.6 Hz, 2H), 6.90 (d, J = 8.6 Hz, 2H), 5.70 (ddd, J = 17.2, 10.4, 8.1 Hz, 1H), 5.31 – 5.07 (m, 2H), 5.04 (s, 2H), 3.61 (dd, J = 10.7, 4.8 Hz, 1H), 3.47 (dd, J = 10.6, 7.4 Hz, 1H), 2.69 (dd, J = 13.8, 7.1 Hz, 1H), 2.61 (dd, J = 13.7, 7.3 Hz, 1H), 2.58 – 2.38 (m, 1H), 1.42 (s, 1H).

$^{13}C\{^1H\}$ NMR (126 MHz, $CDCl_3$) δ 157.3, 139.4, 137.3, 132.1, 130.2, 128.7, 128.1, 127.6, 117.5, 114.8, 70.2, 64.9, 48.3, 36.6.

HRMS (ESI) m/z calculated for $C_{18}H_{20}O_2$([M+Na]$^+$): 291.1356, found: 291.1352.

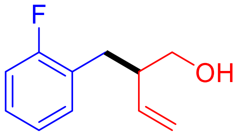

2-(2-fluorobenzyl)but-3-en-1-ol (3am) was prepared according to General Procedure A. Yield 75%, yellow oil.

^1H NMR (500 MHz, $CDCl_3$) δ 7.22 – 7.13 (m, 2H), 7.10 – 6.79 (m, 2H), 5.76 – 5.65 (m, 1H), 5.17 – 5.04 (m, 2H), 3.66 – 3.58 (m, 1H), 3.51 (dd, J = 10.7, 7.4 Hz, 1H), 2.80 (dd, J = 13.6, 6.9 Hz, 1H), 2.68 (dd, J = 13.6, 7.8 Hz, 1H), 2.63 – 2.52 (m, 1H), 1.49 (s, 1H).

$^{13}C\{^1H\}$ NMR (126 MHz, $CDCl_3$) δ 161.4 (d, $^1J_{CF}$ = 244.5 Hz), 138.9, 131.6 (d, $^3J_{CF}$ = 5.0 Hz), 128.0 (d, $^3J_{CF}$ = 8.0 Hz), 126.7 (d, $^2J_{CF}$ = 15.9 Hz), 124.0 (d, J = 3.6 Hz), 117.7, 115.4 (d, $^2J_{CF}$ = 22.4 Hz), 65.0, 47.31, 30.5 (d, $^3J_{CF}$ = 1.9 Hz).

^{19}F NMR (471 MHz, $CDCl_3$) δ -117.9

HRMS (ESI) m/z calculated for $C_{11}H_{13}FO$([M+Na]$^+$): 203.0843, found: 203.0839.

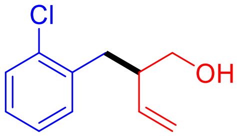

2-(2-chlorobenzyl)but-3-en-1-ol (3an) was prepared according to General Procedure A. Yield 76%, yellow oil.

^1H NMR (500 MHz, $CDCl_3$) δ 7.34 (dd, J = 7.4, 1.6 Hz, 1H), 7.23 – 6.87 (m, 3H), 5.74 (ddd, J = 17.3, 10.4, 8.3 Hz, 1H), 5.25 – 5.00 (m, 2H), 3.76 – 3.59 (m, 1H), 3.52 (dd, J = 10.7, 7.4 Hz, 1H), 2.91 (dd, J = 13.6, 6.9 Hz, 1H), 2.75 (dd, J = 13.6, 7.7 Hz, 1H), 2.70 – 2.57 (m, 1H), 1.45 (s, 1H).

$^{13}C\{^1H\}$ NMR (126 MHz, $CDCl_3$) δ 138.9, 137.6, 134.3, 131.6, 129.7, 127.7, 126.7, 117.8, 65.0, 46.8, 35.0.

HRMS (ESI) m/z calculated for $C_{11}H_{13}ClO$([M+Na]$^+$): 219.0547, found: 219.0552.

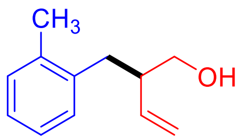

2-(2-methylbenzyl)but-3-en-1-ol (3ao) was prepared according to General Procedure B. Yield 68%, yellow oil.

^{1}H NMR (500 MHz, $CDCl_3$) δ 7.22 – 7.09 (m, 4H), 5.77 (ddd, J = 17.2, 10.4, 8.2 Hz, 1H), 5.32 – 5.15 (m, 1H), 5.15 – 5.06 (m, 1H), 3.66 (dd, J = 10.6, 4.8 Hz, 1H), 3.54 (dd, J = 10.7, 7.5 Hz, 1H), 2.78 (dd, J = 13.9, 7.3 Hz, 1H), 2.67 (dd, J = 13.9, 7.3 Hz, 1H), 2.62 – 2.51 (m, 1H), 2.34 (s, 3H), 1.50 (s, 1H).

^{13}C{^{1}H} NMR (126 MHz, $CDCl_3$) δ 139.5, 138.1, 136.3, 130.4, 130.0, 126.3, 125.8, 117.4, 65.0, 47.1, 34.7, 19.7.

HRMS (ESI) m/z calculated for $C_{12}H_{16}O$([M+Na]$^+$): 199.1077, found: 199.1093.

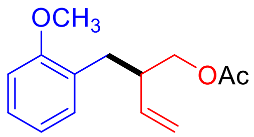

2-(2-methoxybenzyl)but-3-en-1-yl acetate (3ap) was prepared according to General Procedure A. Yield 78%, colourless oil.

^{1}H NMR (800 MHz, $CDCl_3$) δ 7.18 (ddd, J = 8.2, 7.4, 1.8 Hz, 1H), 7.08 (dd, J = 7.4, 1.8 Hz, 1H), 6.87 (td, J = 7.4, 1.1 Hz, 1H), 6.84 (dd, J = 8.2, 1.1 Hz, 1H), 5.73 (ddd, J = 17.3, 10.4, 7.6 Hz, 1H), 5.08 – 4.94 (m, 2H), 4.05 (dd, J = 10.9, 6.7 Hz, 1H), 4.01 (dd, J = 10.9, 5.1 Hz, 1H), 3.82 (s, 3H), 2.80 – 2.73 (m, 2H), 2.70 – 2.64 (m, 1H), 2.03 (s, 3H).

^{13}C{^{1}H} NMR (201 MHz, $CDCl_3$) δ 171.2, 157.7, 139.1, 131.0, 127.9, 127.6, 120.4, 116.0, 110.4, 66.9, 55.3, 42.9, 32.4, 21.1.

HRMS (ESI) m/z calculated for $C_{14}H_{18}O_3$([M+Na]$^+$): 257.1148, found: 257.1157.

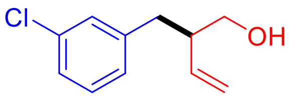

2-(2-chlorobenzyl)but-3-en-1-ol (3aq) was prepared according to General Procedure A. Yield 82%, yellow oil.

^{1}H NMR (500 MHz, $CDCl_3$) δ 7.23 – 7.15 (m, 3H), 7.05 (dt, J = 7.3, 1.6 Hz, 1H), 5.68 (ddd, J = 17.2, 10.4, 8.1 Hz, 1H), 5.35 – 5.12 (m, 1H), 5.12 – 4.98 (m, 1H), 3.60 (dd, J = 10.7, 5.0 Hz, 1H), 3.49 (dd, J = 10.6, 7.2 Hz, 1H), 2.75 (dd, J = 13.6, 6.8 Hz, 1H), 2.63 (dd, J = 13.6, 7.4 Hz, 1H), 2.62 – 2.48 (m, 1H), 1.46 (s, 1H).

^{13}C{^{1}H} NMR (126 MHz, $CDCl_3$) δ 141.9, 138.7, 134.2, 129.6, 129.4, 127.5, 126.4, 117.9, 64.8, 48.0, 37.0.

HRMS (ESI) m/z calculated for $C_{11}H_{13}ClO([M+Na]^+)$: 219.0547, found: 219.0538.

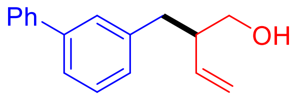

2-([1,1'-biphenyl]-3-ylmethyl)but-3-en-1-ol (3ar) was prepared according to General Procedure A. Yield 69%, yellow oil.

^{1}H NMR (500 MHz, $CDCl_3$) δ 7.62 – 7.56 (m, 2H), 7.49 – 7.42 (m, 3H), 7.41 (t, J = 1.9 Hz, 1H), 7.38 – 7.29 (m, 2H), 7.17 (dt, J = 7.5, 1.5 Hz, 1H), 5.75 (ddd, J = 17.2, 10.4, 8.2 Hz, 1H), 5.23 – 5.16 (m, 1H), 5.16 – 5.09 (m, 1H), 3.65 (dd, J = 10.4, 5.0 Hz, 1H), 3.53 (dd, J = 10.7, 7.5 Hz, 1H), 2.83 (dd, J = 13.6, 7.0 Hz, 1H), 2.74 (dd, J = 13.7, 7.5 Hz, 1H), 2.68 – 2.55 (m, 1H), 1.47 (s, 1H).

^{13}C{^{1}H} NMR (126 MHz, $CDCl_3$) δ 141.4, 140.3, 139.2, 128.9, 128.8, 128.2, 128.2, 127.4, 127.3, 127.3, 125.1, 117.6, 64.9, 48.2, 37.5.

HRMS (ESI) m/z calculated for $C_{17}H_{18}O([M+Na]^+)$: 261.1250, found: 261.1251.

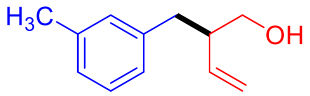

2-(3-methylbenzyl)but-3-en-1-ol (3as) was prepared according to General Procedure A. Yield 72%, yellow oil.

^{1}H NMR (500 MHz, $CDCl_3$) δ 7.17 (t, J = 7.5 Hz, 1H), 7.04 – 6.94 (m, 3H), 5.72 (ddd, J = 17.2, 10.4, 8.1 Hz, 1H), 5.21 – 5.13 (m, 1H), 5.13 – 5.07 (m, 1H), 3.61 (dd, J = 10.7, 4.8 Hz, 1H), 3.48 (dd, J = 10.6, 7.4 Hz, 1H), 2.70 (dd, J = 13.6, 7.3 Hz, 1H), 2.64 (dd, J = 13.6, 7.2 Hz, 1H), 2.60 – 2.50 (m, 1H), 2.33 (s, 3H), 1.43 (s, 1H).

^{13}C{^{1}H} NMR (126 MHz, $CDCl_3$) δ 139.7, 139.4, 138.0, 130.1, 128.3, 127.0, 126.3, 117.4, 64.9, 48.1, 37.4, 21.5.

HRMS (ESI) m/z calculated for $C_{12}H_{16}O([M+Na]^+)$: 199.1093, found: 199.1090.

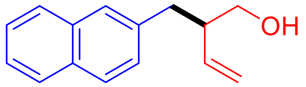

2-(naphthalen-2-ylmethyl)but-3-en-1-ol (3at) was prepared according to General Procedure A. Yield 80%, yellow oil.

^{1}H NMR (500 MHz, $CDCl_3$) δ 7.85 – 7.76 (m, 3H), 7.62 (s, 1H), 7.50 – 7.41 (m, 2H), 7.33 (dd, J = 8.4, 1.8 Hz, 1H), 5.76 (ddd, J = 17.2, 10.4, 8.2 Hz, 1H), 5.18 – 5.08 (m, 2H), 3.66 (dd, J = 10.7, 5.0 Hz, 1H), 3.54 (dd, J = 10.7, 7.3 Hz, 1H), 2.94 (dd, J = 13.7, 7.0 Hz, 1H), 2.83 (dd, J = 13.7, 7.5 Hz, 1H), 2.73 – 2.62 (m, 1H), 1.58 (s, 1H).

$^{13}C\{^1H\}$ **NMR** (126 MHz, $CDCl_3$) δ 139.2, 137.4, 133.6, 132.2, 128.0, 127.9, 127.7, 127.6, 127.5, 126.1, 125.4, 117.6, 64.9, 48.1, 37.6.

HRMS (ESI) m/z calculated for $C_{15}H_{16}O([M+Na]^+)$: 235.1093, found: 235.1087.

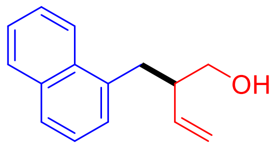

2-(naphthalen-2-ylmethyl)but-3-en-1-ol (3au) was prepared according to General Procedure A. Yield 73%, yellow oil.

^1H **NMR** (500 MHz, $CDCl_3$) δ 8.05 (d, J = 8.3 Hz, 1H), 7.87 (d, J = 8.3 Hz, 1H), 7.74 (d, J = 8.2 Hz, 1H), 7.57 – 7.45 (m, 2H), 7.40 (dd, J = 8.2, 7.0 Hz, 1H), 7.31 (d, J = 7.0 Hz, 1H), 5.81 (ddd, J = 17.2, 10.4, 8.2 Hz, 1H), 5.18 – 5.11 (m, 1H), 5.14 – 4.77 (m, 1H), 3.67 (dd, J = 10.6, 4.9 Hz, 1H), 3.57 (dd, J = 10.7, 7.2 Hz, 1H), 3.24 (dd, J = 13.9, 7.3 Hz, 1H), 3.09 (dd, J = 14.0, 7.1 Hz, 1H), 2.80 – 2.69 (m, 1H), 1.57 (s, 1H).

$^{13}C\{^1H\}$ **NMR** (126 MHz, $CDCl_3$) δ 139.4, 135.9, 134.1, 132.1, 129.0, 127.4, 127.1, 126.0, 125.6, 125.4, 124.0, 117.5, 65.1, 47.2, 34.5.

HRMS (ESI) m/z calculated for $C_{15}H_{16}O([M+Na]^+)$: 235.1093, found: 235.1097.

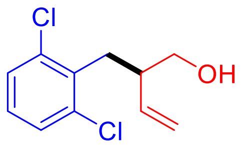

2-(2,6-dichlorobenzyl)but-3-en-1-ol (3av) was prepared according to General Procedure A. Yield 62%, light yellow solid.

^1H **NMR** (500 MHz, $CDCl_3$) δ 7.27 (d, J = 8.0 Hz, 2H), 7.07 (t, J = 8.0 Hz, 1H), 5.80 (ddd, J = 17.1, 10.2, 8.8 Hz, 1H), 5.19 – 5.07 (m, 1H), 5.07 – 4.98 (m, 1H), 3.64 (dd, J = 10.8, 4.9 Hz, 1H), 3.59 (dd, J = 10.7, 7.7 Hz, 1H), 3.03 (dd, J = 13.5, 7.3 Hz, 1H), 2.98 (dd, J = 13.5, 7.7 Hz, 1H), 2.81 – 2.71 (m, 1H), 1.57 (s, 1H).

$^{13}C\{^1H\}$ **NMR** (126 MHz, $CDCl_3$) δ 138.6, 136.2, 135.9, 128.4, 128.0, 117.8, 65.2, 46.7, 32.7.

HRMS (ESI) m/z calculated for $C_{11}H_{12}Cl_2O([M+Na]^+)$: 253.0157, found: 253.0160.

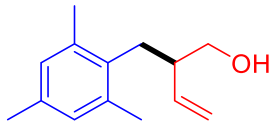

2-(2,4,6-trimethylbenzyl)but-3-en-1-ol (3aw) was prepared according to General Procedure A. Yield 50%, light yellow oil.

^{1}H NMR (500 MHz, $CDCl_3$) δ 6.83 (s, 2H), 5.77 (ddd, J = 17.2, 10.3, 8.4 Hz, 1H), 5.22 – 5.11 (m, 1H), 5.10 – 4.91 (m, 1H), 3.69 – 3.59 (m, 1H), 3.53 (dd, J = 10.3, 7.7 Hz, 1H), 2.72 (dd, J = 14.0, 7.6 Hz, 1H), 2.64 (dd, J = 14.0, 7.0 Hz, 1H), 2.54 – 2.44 (m, 1H), 2.28 (s, 6H), 2.25 (s, 3H), 1.42 (s, 1H).

^{13}C{^{1}H} NMR (126 MHz, $CDCl_3$) δ 139.6, 136.6, 135.4, 133.7, 129.2, 117.2, 65.1, 47.4, 30.7, 20.9, 20.5.

HRMS (ESI) m/z calculated for $C_{14}H_{20}O([M+Na]^+)$: 227.1406, found: 227.1404.

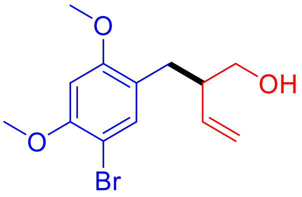

2-(5-bromo-2,4-dimethoxybenzyl)but-3-en-1-ol (3ax) was prepared according to General Procedure A. Yield 46%, light yellow oil.

^{1}H NMR (500 MHz, $CDCl_3$) δ 7.23 (s, 1H), 6.46 (s, 1H), 5.77 – 5.67 (m, 1H), 5.14 – 5.04 (m, 2H), 3.89 (s, 3H), 3.84 (s, 3H), 3.52 (dd, J = 10.9, 4.8 Hz, 1H), 3.43 (dd, J = 11.0, 6.7 Hz, 1H), 2.68 (dd, J = 13.5, 7.3 Hz, 1H), 2.56 (dd, J = 13.5, 6.9 Hz, 1H), 2.53 – 2.37 (m, 1H), 1.82 (s, 1H).

^{13}C{^{1}H} NMR (126 MHz, $CDCl_3$) δ 157.8, 155.2, 139.5, 134.6, 121.8, 116.9, 101.8, 96.7, 64.7, 56.5, 55.9, 47.0, 30.4.

HRMS (ESI) m/z calculated for $C_{13}H_{17}BrO_3([M+Na]^+)$: 323.0253, found: 323.0245.

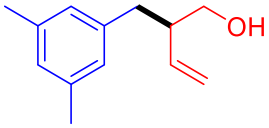

2-(3,5-dimethylbenzyl)but-3-en-1-ol (3ay) was prepared according to General Procedure A. Yield 45%, light yellow oil.

^{1}H NMR (500 MHz, $CDCl_3$) δ 6.86 (s, 1H), 6.81 (s, 2H), 5.74 (ddd, J = 17.2, 10.4, 8.0 Hz, 1H), 5.21 – 5.11 (m, 2H), 3.67 – 3.59 (m, 1H), 3.49 (dd, J = 10.7, 7.4 Hz, 1H), 2.68 (dd, J = 13.6, 7.8 Hz, 1H), 2.63 (dd, J = 13.4, 6.8 Hz, 1H), 2.61 – 2.45 (m, 1H), 2.31 (s, 6H), 1.43 (s, 1H).

^{13}C{^{1}H} NMR (126 MHz, $CDCl_3$) δ 139.7, 139.6, 137.9, 127.9, 127.1, 117.3, 64.9, 48.0, 37.3, 21.4.

HRMS (ESI) m/z calculated for $C_{13}H_{18}O([M+Na]^+)$: 213.1250, found: 213.1252.

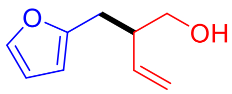

2-(furan-2-ylmethyl)but-3-en-1-ol (3az) was prepared according to General Procedure B. Yield 69%, light yellow oil.

^{1}H NMR (500 MHz, $CDCl_3$) δ 7.31 (dd, J = 2.0, 0.8 Hz, 1H), 6.28 (dd, J = 3.2, 1.9 Hz, 1H), 6.03 (dd, J = 3.2, 0.9 Hz, 1H), 5.72 (ddd, J = 16.8, 10.8, 8.1 Hz, 1H), 5.20 – 5.11 (m, 2H), 3.62 (dd, J = 10.8, 5.2 Hz, 1H), 3.51 (dd, J = 10.8, 7.0 Hz, 1H), 2.78 (dd, J = 15.0, 7.0 Hz, 1H), 2.72 (dd, J = 15.0, 7.0 Hz, 1H), 2.69 – 2.60 (m, 1H), 1.51 (s, 1H).

^{13}C{^{1}H} NMR (126 MHz, $CDCl_3$) δ 153.8, 141.3, 138.8, 117.5, 110.3, 106.5, 65.0, 45.6, 29.6.

HRMS (ESI) m/z calculated for $C_9H_{12}O_2$([M+Na]$^+$): 175.0730, found: 175.0726.

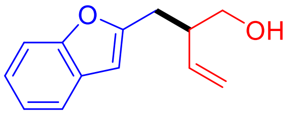

2-(benzofuran-2-ylmethyl)but-3-en-1-ol (3ba) was prepared according to General Procedure A. Yield 69%, light yellow oil.

^{1}H NMR (500 MHz, $CDCl_3$) δ 7.52 – 7.45 (m, 1H), 7.42 (d, J = 8.2 Hz, 1H), 7.25 – 7.15 (m, 2H), 6.44 (d, J = 1.0 Hz, 1H), 5.77 (ddd, J = 17.8, 10.8, 8.0 Hz, 1H), 5.22 – 5.14 (m, 2H), 3.68 (dd, J = 10.8, 5.2 Hz, 1H), 3.59 (dd, J = 10.8, 6.8 Hz, 1H), 2.95 (dd, J = 15.0, 6.7 Hz, 1H), 2.86 (dd, J = 15.0, 7.3 Hz, 1H), 2.78 (h, J = 6.9 Hz, 1H), 1.59 (s, 1H).

^{13}C{^{1}H} NMR (126 MHz, $CDCl_3$) δ 157.0, 154.8, 138.5, 128.9, 123.5, 122.6, 120.5, 117.7, 110.9, 103.6, 65.0, 45.1, 30.1.

HRMS (ESI) m/z calculated for $C_{13}H_{14}O_2$([M+Na]$^+$): 225.0886, found: 225.0877.

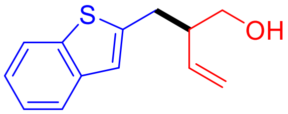

2-(benzothiophen-2-ylmethyl)but-3-en-1-ol (3bb) was prepared according to General Procedure A. Yield 60%, light yellow oil.

^{1}H NMR (500 MHz, $CDCl_3$) δ 7.76 (d, J = 7.9 Hz, 1H), 7.67 (d, J = 7.7 Hz, 1H), 7.36 – 7.23 (m, 3H), 7.03 (d, J = 0.9 Hz, 1H), 5.77 (ddd, J = 17.8, 9.9, 8.0 Hz, 1H), 5.23 – 5.16 (m, 2H), 3.62 – 3.55 (m, 1H), 3.09 (dd, J = 14.7, 6.6 Hz, 1H), 2.97 (dd, J = 14.7, 7.7 Hz, 1H), 2.68 (h, J = 6.9 Hz, 1H), 1.45 (s, 1H).

^{13}C{^{1}H} NMR (126 MHz, $CDCl_3$) δ 143.5, 140.2, 139.7, 138.5, 124.3, 123.7, 123.0, 122.2, 122.1, 118.1, 64.9, 47.7, 32.3.

HRMS (ESI) m/z calculated for $C_{13}H_{14}OS([M+Na]^+)$: 241.0658, found: 241.0646.

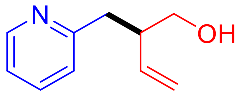

2-(pyridin-2-ylmethyl)but-3-en-1-ol (3bd) was prepared according to General Procedure A. Yield 71%, light yellow oil.

^{1}H NMR (500 MHz, $CDCl_3$) δ 8.49 (ddd, J = 4.9, 1.9, 0.9 Hz, 1H), 7.60 (td, J = 7.7, 1.9 Hz, 1H), 7.18 – 7.10 (m, 2H), 5.76 (ddd, J = 17.0, 10.6, 8.0 Hz, 1H), 5.14 – 5.02 (m, 2H), 3.58 (dd, J = 11.1, 5.7 Hz, 1H), 3.54 (dd, J = 11.1, 6.3 Hz, 1H), 2.97 (s, 1H), 2.96 (s, 1H), 2.79 – 2.69 (m, 1H).

^{13}C{^{1}H} NMR (126 MHz, $CDCl_3$) δ 159.8, 148.9, 139.2, 136.8, 124.1, 121.5, 116.3, 65.1, 46.0, 40.1.

HRMS (ESI) m/z calculated for $C_{10}H_{13}NO([M+Na]^+)$: 186.0889, found: 186.0891.

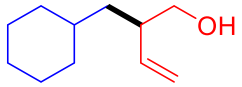

2-(cyclohexylmethyl)but-3-en-1-ol (3be) was prepared according to General Procedure C. Yield 55%, white solid.

^{1}H NMR (500 MHz, $CDCl_3$) δ 5.55 (ddd, J = 17.1, 10.4, 8.8 Hz, 1H), 5.18 – 5.06 (m, 2H), 3.52 (dd, J = 10.6, 4.9 Hz, 1H), 3.35 (dd, J = 10.5, 8.4 Hz, 1H), 2.40 – 2.29 (m, 1H), 1.84 – 1.58 (m, 5H), 1.56 (s, 1H), 1.34 – 1.08 (m, 6H), 0.99 – 0.85 (m, 1H), 0.85 – 0.75 (m, 1H).

^{13}C{^{1}H} NMR (126 MHz, $CDCl_3$) δ 140.5, 117.3, 66.0, 44.3, 38.5, 34.9, 34.3, 32.8, 26.8, 26.5, 26.3.

HRMS (ESI) m/z calculated for $C_{11}H_{20}O([M+Na]^+)$: 191.1406, found: 191.1402.

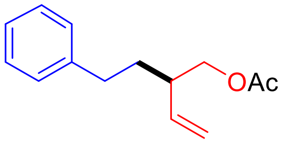

2-phenethylbut-3-en-1-yl acetate (3bf) was prepared according to General Procedure C. Yield 56%, colourless oil.

^{1}H NMR (800 MHz, $CDCl_3$) δ 7.28 (t, J = 7.6 Hz, 2H), 7.21 – 7.15 (m, 3H), 5.67 (ddd, J = 17.2, 10.4, 8.5 Hz, 1H), 5.16 (dd, J = 10.4, 1.7 Hz, 1H), 5.13 (dd, J = 17.3, 1.2 Hz, 1H), 4.05 (dd, J = 10.9, 7.0 Hz, 1H), 4.01 (dd, J = 10.9, 6.1 Hz, 1H), 2.70 (ddd, J = 14.0, 10.3, 5.3 Hz, 1H), 2.56 (ddd, J = 13.9, 10.2, 6.6 Hz, 1H), 2.44 – 2.37 (m, 1H), 2.04 (s, 3H), 1.79 (dddd, J = 13.6, 10.2, 6.6, 4.6 Hz, 1H), 1.61 (dtd, J = 13.7, 9.8, 5.3 Hz, 1H).

^{13}C{^{1}H} NMR (201 MHz, $CDCl_3$) δ 171.2, 142.2, 138.8, 128.5, 126.0, 117.2, 67.3, 42.9, 33.2, 32.9, 21.1.

HRMS (ESI) m/z calculated for $C_{14}H_{18}O_2([M+Na]^+)$: 241.1199, found: 241.1202.

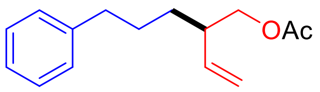

5-phenyl-2-vinylpentyl acetate (3bg) was prepared according to General Procedure C. Yield 65%, colourless oil.

^{1}H NMR (500 MHz, $CDCl_3$) δ 7.33 – 7.25 (m, 2H), 7.21 – 7.13 (m, 3H), 5.59 (ddd, J = 16.7, 10.7, 8.4 Hz, 1H), 5.12 – 5.03 (m, 2H), 3.99 (d, J = 6.7 Hz, 2H), 2.68 – 2.53 (m, 2H), 2.44 – 2.33 (m, 1H), 2.03 (s, 3H), 1.75 – 1.64 (m, 1H), 1.63 – 1.54 (m, 1H), 1.51 – 1.44 (m, 1H), 1.39 – 1.24 (m, 1H).

^{13}C{^{1}H} NMR (126 MHz, $CDCl_3$) δ 171.3, 142.5, 139.0, 128.5, 128.4, 125.9, 116.7, 67.4, 43.2, 36.0, 30.7, 28.8, 21.1.

HRMS (ESI) m/z calculated for $C_{15}H_{20}O_2$([M+Na]$^+$): 255.1356, found: 255.1359.

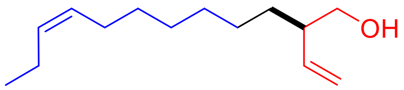

(*Z*)-2-vinyldodec-9-en-1-ol (3bh) was prepared according to General Procedure C. Yield 35%, colourless oil.

^{1}H NMR (500 MHz, $CDCl_3$) δ 5.58 (ddd, J = 17.0, 10.4, 8.7 Hz, 1H), 5.47 – 5.26 (m, 2H), 5.23 – 5.05 (m, 2H), 3.64 – 3.52 (m, 1H), 3.40 (t, J = 9.9 Hz, 1H), 2.21 (qt, J = 8.5, 4.6 Hz, 1H), 2.02 (dt, J = 13.7, 7.2 Hz, 4H), 1.46 – 1.16 (m, 11H), 0.95 (t, J = 7.5 Hz, 3H).

^{13}C{^{1}H} NMR (126 MHz, $CDCl_3$) δ 140.3, 131.7, 129.4, 117.5, 65.8, 47.2, 30.8, 29.9, 29.7, 29.3, 27.2, 27.1, 20.7, 14.5.

HRMS (ESI) m/z calculated for $C_{14}H_{26}O$([M+Na]$^+$): 233.1876, found: 233.1878.

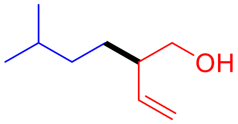

5-methyl-2-vinylhexan-1-ol (3bi) was prepared according to General Procedure C. Yield 43%, colourless oil.

^{1}H NMR (500 MHz, $CDCl_3$) δ 5.58 (ddd, J = 17.1, 10.4, 8.7 Hz, 1H), 5.19 – 5.10 (m, 2H), 3.58 (dd, J = 10.5, 5.0 Hz, 1H), 3.41 (dd, J = 10.5, 8.2 Hz, 1H), 2.22 – 2.13 (m, 1H), 1.56 – 1.47 (m, 1H), 1.45 – 1.37 (m, 1H), 1.22 – 1.10 (m, 3H), 0.88 (d, J = 6.5 Hz, 3H), 0.86 (d, J = 6.5 Hz, 3H).

^{13}C{^{1}H} NMR (126 MHz, $CDCl_3$) δ 140.3, 117.5, 65.8, 47.5, 36.4, 28.6, 28.2, 22.9, 22.6.

HRMS (ESI) m/z calculated for $C_9H_{18}O$([M+Na]$^+$): 165.1250, found: 165.1255.

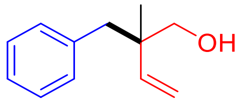

2-benzyl-2-methylbut-3-en-1-ol (3bj) was prepared according to General Procedure A. Yield 61%, yellow solid.

^{1}H NMR (500 MHz, $CDCl_3$) δ 7.32 – 7.24 (m, 2H), 7.24 – 7.18 (m, 1H), 7.18 – 7.13 (m, 2H), 5.84 (dd, J = 17.6, 10.9 Hz, 1H), 5.17 (dd, J = 10.9, 1.2 Hz, 1H), 5.03 (dd, J = 17.7, 1.2 Hz, 1H), 3.43 (d, J = 10.7 Hz, 1H), 3.37 (d, J = 10.7 Hz, 1H), 2.73 (d, J = 13.1 Hz, 1H), 2.65 (d, J = 13.1 Hz, 1H), 1.44 (s, 1H), 0.98 (s, 3H).

^{13}C{^{1}H} NMR (126 MHz, $CDCl_3$) δ 144.0, 137.9, 130.7, 127.9, 126.3, 114.8, 69.1, 43.3, 43.2, 20.3.

HRMS (ESI) m/z calculated for $C_{12}H_{16}O([M+Na]^+)$: 199.1093, found: 191.1090.

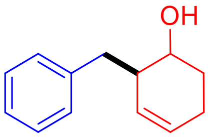

2-benzylcyclohex-3-en-1-ol (3bk) was prepared according to General Procedure A. Yield 70%, off-white solid.

^{1}H NMR (500 MHz, $CDCl_3$) δ 7.34 – 7.24 (m, 2H), 7.25 – 7.18 (m, 3H), 5.69 – 5.61 (m, 1H), 5.49 – 5.42 (m, 1H), 3.74 – 3.54 (m, 1H), 2.98 (dd, J = 13.4, 5.9 Hz, 1H), 2.55 (dd, J = 13.4, 8.8 Hz, 1H), 2.44 – 2.34 (m, 1H), 2.21 – 2.03 (m, 2H), 1.94 – 1.86 (m, 1H), 1.71 – 1.60 (m, 1H), 1.56 (s, 1H).

^{13}C{^{1}H} NMR (126 MHz, $CDCl_3$) δ 140.2, 129.4, 128.5, 128.2, 126.8, 126.2, 71.4, 45.8, 39.8, 29.9, 23.9.

HRMS (ESI) m/z calculated for $C_{13}H_{16}O([M+Na]^+)$: 211.1093, found: 211.1095.

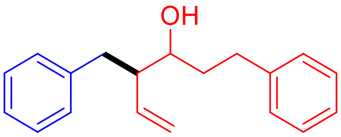

4-benzyl-1-phenylhex-5-en-3-ol (3bl) was prepared according to General Procedure A. Yield 79%, yellow oil.

^{1}H NMR (500 MHz, $CDCl_3$) δ 7.36 – 7.23 (m, 4H), 7.23 – 7.11 (m, 6H), 5.77 (ddd, J = 17.2, 10.3, 8.9 Hz, 1H), 5.14 (dd, J = 10.4, 1.9 Hz, 1H), 5.01 (ddd, J = 17.3, 1.9, 0.9 Hz, 1H), 3.57 (dt, J = 8.5, 4.3 Hz, 1H), 2.89 (dd, J = 13.6, 6.5 Hz, 1H), 2.79 (ddd, J = 13.6, 9.8, 5.9 Hz, 1H), 2.72 – 2.60 (m, 2H), 2.40 (tdd, J = 8.6, 6.5, 4.2 Hz, 1H), 1.89 – 1.73 (m, 2H), 1.54 (s, 1H).

^{13}C{^{1}H} NMR (126 MHz, $CDCl_3$) δ 142.2, 140.3, 137.5, 129.4, 128.6, 128.5, 128.4, 126.1, 126.0, 118.4, 72.2, 51.6, 37.7, 37.3, 32.4.

HRMS (ESI) m/z calculated for $C_{19}H_{22}O([M+Na]^+)$: 289.1563, found: 289.1556.

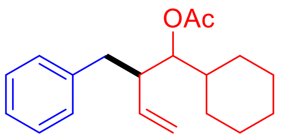

2-benzyl-1-cyclohexylbut-3-en-1-yl acetate (3bm) was prepared according to General Procedure A. Yield 88%, clear oil.

^{1}H NMR (500 MHz, $CDCl_3$) δ 7.26 (t, J = 7.4 Hz, 2H), 7.21 – 7.14 (m, 1H), 7.14 – 7.08 (m, 2H), 5.71 (ddd, J = 17.2, 10.3, 9.3 Hz, 1H), 5.02 (dd, J = 10.3, 1.9 Hz, 1H), 4.86 – 4.77 (m, 2H), 2.73 (dd, J = 13.3, 5.1 Hz, 1H), 2.63 (tdd, J = 9.0, 5.2, 3.8 Hz, 1H), 2.51 (dd, J = 13.3, 8.9 Hz, 1H), 2.11 (s, 3H), 1.80 – 1.60 (m, 5H), 1.60 – 1.47 (m, 1H), 1.42 – 1.06 (m, 3H), 1.05 – 0.88 (m, 2H).

^{13}C{^{1}H} NMR (126 MHz, $CDCl_3$) δ 171.1, 140.0, 137.0, 129.4, 128.2, 126.1, 117.6, 79.3, 47.6, 39.5, 38.7, 29.2, 28.6, 26.4, 26.0, 25.9, 21.1.

HRMS (ESI) m/z calculated for $C_{19}H_{26}O_2$([M+Na]$^+$): 309.1825, found: 309.1840.

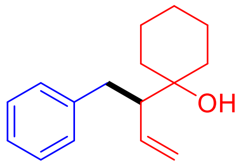

1-(1-phenylbut-3-en-2-yl)cyclohexan-1-ol (3bn) was prepared according to General Procedure A. Yield 82%, colourless oil.

^{1}H NMR (500 MHz, $CDCl_3$) δ 7.26 – 7.20 (m, 2H), 7.17 – 7.05 (m, 3H), 5.66 (dt, J = 17.1, 10.0 Hz, 1H), 5.00 (dd, J = 10.2, 2.0 Hz, 1H), 4.75 (dd, J = 17.2, 2.0 Hz, 1H), 3.08 (dd, J = 13.5, 3.0 Hz, 1H), 2.47 (dd, J = 13.4, 11.0 Hz, 1H), 2.25 (ddd, J = 11.0, 9.5, 3.0 Hz, 1H), 1.70 – 1.49 (m, 9H), 1.38 (s, 1H), 1.31 – 1.16 (m, 1H).

^{13}C{^{1}H} NMR (126 MHz, $CDCl_3$) δ 141.4, 137.9, 129.4, 128.2, 125.8, 118.6, 72.7, 57.6, 35.4, 35.1, 35.0, 26.0, 22.0, 22.0.

HRMS (ESI) m/z calculated for $C_{16}H_{22}O$ ([M+Na]$^+$): 253.1563, found: 253.1568.

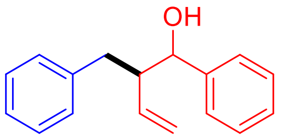

2-benzyl-1-phenylbut-3-en-1-ol (3bo) was prepared according to General Procedure A. Yield 76%, yellow oil.

^{1}H NMR (500 MHz, $CDCl_3$) δ 7.40 – 7.33 (m, 4H), 7.33 – 7.28 (m, 1H), 7.28 – 7.21 (m, 2H), 7.21 – 7.12 (m, 1H), 7.12 – 7.06 (m, 2H), 5.73 (ddd, J = 17.2, 10.3, 8.4 Hz, 1H), 5.15 (dd, J = 10.4, 1.7 Hz, 1H), 4.99 (ddd, J = 17.2, 1.8, 0.8 Hz, 1H), 4.55 (d, J = 6.3 Hz, 1H), 2.73 (dd, J = 13.0, 5.3 Hz, 1H), 2.68 (tt, J = 8.6, 5.8 Hz, 1H), 2.55 (dd, J = 13.0, 8.8 Hz, 1H), 2.15 (s, 1H).

^{13}**C{^{1}H} NMR** (126 MHz, $CDCl_3$) δ 142.2, 140.3, 137.5, 129.4, 128.6, 128.5, 128.4, 126.1, 126.0, 118.4, 72.2, 51.6, 37.7, 37.3, 32.4.

HRMS (ESI) m/z calculated for $C_{17}H_{18}O([M+Na]^+)$: 261.1250, found: 261.1254.

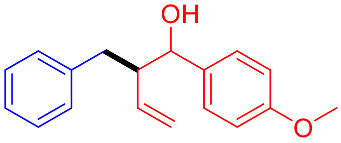

2-benzyl-1-(4-methoxyphenyl)but-3-en-1-ol (3bp) was prepared according to General Procedure A. Yield 84%, yellow oil.

^{1}**H NMR** (500 MHz, $CDCl_3$) δ 7.28 (d, J = 8.7 Hz, 2H), 7.26 – 7.20 (m, 2H), 7.20 – 7.11 (m, 1H), 7.09 – 7.03 (m, 2H), 6.90 (d, J = 8.7 Hz, 2H), .72 (ddd, J = 17.2, 10.3, 8.6 Hz, 1H), 5.14 (dd, J = 10.3, 1.7 Hz, 1H), 4.99 (ddd, J = 17.2, 1.7, 0.8 Hz, 1H), 4.49 (d, J = 6.8 Hz, 1H), 3.82 (s, 3H), 2.69 (dd, J = 13.2, 4.9 Hz, 1H), 2.66 – 2.57 (m, 1H), 2.49 (dd, J = 13.1, 9.1 Hz, 1H), 2.12 (s, 1H).

^{13}**C{^{1}H} NMR** (126 MHz, $CDCl_3$) δ 159.3, 140.1, 138.2, 134.7, 129.3, 128.3, 128.1, 126.0, 119.1, 113.9, 75.5, 55.4, 54.2, 37.4.

HRMS (ESI) m/z calculated for $C_{18}H_{20}O_2([M+Na]^+)$: 291.1356, found: 291.1365.

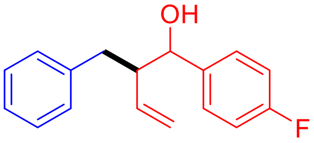

2-benzyl-1-(4-fluorophenyl)but-3-en-1-ol (3bq) was prepared according to General Procedure A. Yield 89%, yellow oil.

^{1}**H NMR** (500 MHz, $CDCl_3$) δ 7.35 – 7.28 (m, 2H), 7.27 – 7.22 (m, 2H), 7.20 – 7.13 (m, 1H), 7.10 – 7.00 (m, 4H), 5.71 (ddd, J = 17.2, 10.3, 8.5 Hz, 1H), 5.15 (dd, J = 10.3, 1.7 Hz, 1H), 4.98 (d, J = 17.2 Hz, 1H), 4.53 (d, J = 6.4 Hz, 1H), 2.70 (dd, J = 13.0, 5.1 Hz, 1H), 2.66 – 2.57 (m, 1H), 2.53 (dd, J = 13.0, 9.0 Hz, 1H), 2.14 (s, 1H).

^{13}**C{^{1}H} NMR** (126 MHz, $CDCl_3$) δ 162.4 (d, $^{1}J_{CF}$ = 245.4 Hz), 139.9, 138.38 (d, $^{4}J_{CF}$ = 3.2 Hz), 137.7, 129.2, 128.5 (d, $^{3}J_{CF}$ = 8.0 Hz), 128.4, 126.2, 119.4, 115.3 (d, $^{2}J_{CF}$ = 21.5 Hz), 75.0, 54.2, 37.4.

^{19}**F NMR** (471 MHz, $CDCl_3$) δ -115.0

HRMS (ESI) m/z calculated for $C_{17}H_{17}FO([M+Na]^+)$: 279.1156, found: 279.1151.

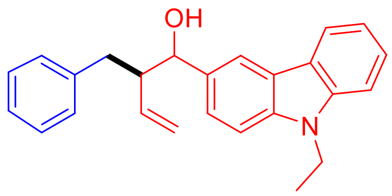

2-benzyl-1-(9-ethyl-9H-carbazol-3-yl)but-3-en-1-ol (3br) was prepared according to General Procedure A. Yield 53%, yellow oil.

^{1}H NMR (800 MHz, $CDCl_3$) δ 8.17 – 8.08 (m, 2H), 7.51 – 7.46 (m, 2H), 7.45 – 7.39 (m, 2H), 7.28 – 7.21 (m, 3H), 7.18 – 7.13 (m, 1H), 7.10 – 7.06 (m, 2H), 5.81 (dtt, J = 17.1, 10.4, 1.5 Hz, 1H), 5.18 (d, J = 10.4 Hz, 1H), 5.05 (d, J = 17.1 Hz, 1H), 4.74 (d, J = 7.0 Hz, 1H), 4.39 (q, J = 7.3 Hz, 2H), 2.81 (dt, J = 15.2, 5.9 Hz, 1H), 2.77 – 2.72 (m, 1H), 2.58 – 2.52 (m, 1H), 2.30 (s, 1H), 1.45 (t, J = 7.3 Hz, 3H).

^{13}C{^{1}H} NMR (201 MHz, $CDCl_3$) δ 140.4, 140.3, 139.8, 138.6, 133.0, 129.3, 128.2, 125.9, 125.8, 124.7, 123.0, 120.6, 119.0, 118.9, 108.7, 108.4, 77.3, 77.0, 76.6, 54.6, 37.7, 37.6, 14.0.

HRMS (ESI) m/z calculated for $C_{25}H_{25}NO([M+Na]^+)$: 378.1828, found: 378.1841.

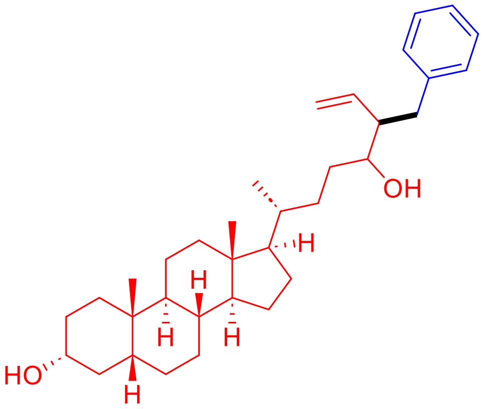

(3R,5R,8R,9S,10S,13R,14S,17R)-17-((2R)-6-benzyl-5-hydroxyoct-7-en-2-yl)-10,13-dimethylhexadecahydro-1H-cyclopenta[a]phenanthren-3-ol (3bs) was prepared according to General Procedure A. Yield 77%, off-white solid, R_f = 0.23 (3:1 Hexanes/EtOAc), 1:1 d.r.

^{1}H NMR (500 MHz, $CDCl_3$) δ 7.31 – 7.22 (m, 2H), 7.21 – 7.14 (m, 3H), 5.79 (ddd, J = 17.2, 10.4, 8.8 Hz, 0.5H), 5.75 (ddd, J = 17.2, 10.4, 8.8 Hz, 0.5H), 5.13 (dd, J = 10.4, 1.9 Hz, 0.5H), 5.11 (dd, J = 10.4, 1.9 Hz, 0.5H), 5.00 (ddd, J = 17.2, 2.0, 0.8 Hz, 0.5H), 4.98 (ddd, J = 17.2, 2.0, 0.8 Hz, 0.5H), 3.67 – 3.57 (m, 1H), 3.50 – 3.42 (m, 1H), 2.89 (dd, J = 13.6, 6.7 Hz, 0.5H), 2.88 (dd, J = 13.6, 6.7 Hz, 0.5H) 2.71 (dd, J = 14.0, 8.2 Hz, 0.5H), 2.65 (dd, J = 14.0, 8.2 Hz, 0.5H) 2.45 – 2.31 (m, 1H), 1.98 – 1.92 (m, 1H), 1.90 – 0.95 (m, 29H), 0.92 (s, 3H), 0.90 (d, J = 6.2 Hz, 1.5H), 0.88 (d, J = 6.2 Hz, 1.5H), 0.63 (s, 1.5H), 0.62 (s, 1.5H) (mixture of diastereomers).

^{13}C{^{1}H} NMR (126 MHz, $CDCl_3$) δ 140.5, 140.5, 137.8, 137.6, 129.4, 128.3, 128.3, 126.0, 118.1, 118.0, 77.4, 76.9, 73.1, 73.1, 72.0, 56.6, 56.3, 56.2, 51.5, 50.9, 42.8, 42.2, 40.6, 40.3, 37.9, 37.8, 36.6, 36.0, 35.9, 35.9, 35.5, 34.7, 32.0, 32.0, 31.8, 30.7, 28.5, 28.4, 27.3, 26.6, 24.4, 23.5, 21.0, 18.8, 12.2, 12.2 (mixture of diastereomers).

HRMS (APCI) m/z calculated for $C_{34}H_{52}O_2$([M+Na]$^+$): 515.3860, found: 515.3863.

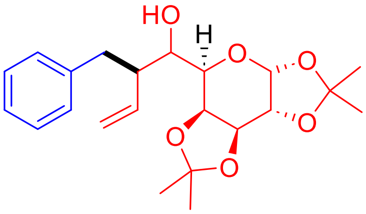

2-benzyl-1-((3aR,5R,5aS,8aS,8bR)-2,2,7,7-tetramethyltetrahydro-5H-bis([1,3]dioxolo)[4,5-b:4',5'-d]pyran-5-yl)but-3-en-1-ol (3bt) was prepared according to General Procedure A. Purified via silica gel column chromatography 10-60% ethyl acetate/hexanes Yield 85%, white solid.

^{1}H NMR (500 MHz, $CDCl_3$) δ 7.28 – 7.21 (m, 2H), 7.21 – 7.12 (m, 3H), 5.83 (ddd, J = 17.3, 10.4, 8.9 Hz, 1H), 5.50 (d, J = 5.1 Hz, 1H), 5.11 (dd, J = 10.4, 2.1 Hz, 1H), 5.09 – 5.01 (m, 1H), 4.61 (dd, J = 7.9, 2.5 Hz, 1H), 4.40 (dd, J = 7.9, 2.1 Hz, 1H), 4.30 (dd, J = 5.2, 2.5 Hz, 1H), 3.82 (ddd, J = 9.0, 5.8, 2.0 Hz, 1H), 3.69 (dd, J = 9.1, 2.1 Hz, 1H), 2.90 (dd, J = 13.4, 6.8 Hz, 1H), 2.86 (dd, J = 13.2, 8.4 Hz, 1H), 2.83 – 2.72 (m, 1H), 1.98 (d, J = 5.8 Hz, 1H), 1.47 (s, 3H), 1.39 (s, 3H), 1.35 (s, 3H), 1.30 (s, 3H).

^{13}C{^{1}H} NMR (126 MHz, $CDCl_3$) δ 140.5, 136.3, 129.4, 128.2, 125.9, 119.0, 109.4, 108.8, 96.5, 72.0, 71.4, 70.9, 70.5, 68.0, 46.4, 38.3, 26.1, 26.0, 25.1, 24.8.

HRMS (ESI) m/z calculated for $C_{22}H_{30}O_6$([M+Na]$^+$): 413.1935, found: 413.1922.

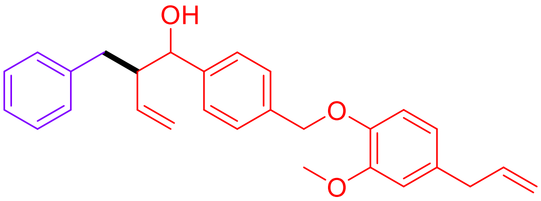

1-(4-((4-allyl-2-methoxyphenoxy)methyl)phenyl)-2-benzylbut-3-en-1-ol (3bu) was prepared according to General Procedure A. Yield 82%, light yellow oil.

^{1}H NMR (500 MHz, $CDCl_3$) δ 7.44 (d, J = 8.0 Hz, 2H), 7.34 (d, J = 8.2 Hz, 2H), 7.26 (t, J = 7.3 Hz, 2H), 7.18 (t, J = 7.4 Hz, 1H), 7.09 (d, J = 6.7 Hz, 1H), 6.83 (d, J = 8.2 Hz, 1H), 6.75 (d, J = 2.0 Hz, 1H), 6.68 (dd, J = 8.2, 2.0 Hz, 1H), 5.97 (ddt, J = 16.8, 9.9, 6.7 Hz, 1H), 5.73 (ddd, J = 17.2, 10.4, 8.5 Hz, 1H), 5.19 – 5.05 (m, 5H), 5.02 – 4.95 (m, 1H), 4.55 (d, J = 6.3 Hz, 1H), 3.89 (s, 3H), 3.34 (dd, J = 6.7, 1.6 Hz, 2H), 2.74 (dd, J = 13.2, 5.3 Hz, 1H), 2.67 (ddd, J = 14.4, 8.7, 5.9 Hz, 1H), 2.55 (dd, J = 13.2, 8.9 Hz, 1H), 2.18 (s, 1H).

^{13}C{^{1}H} NMR (126 MHz, $CDCl_3$) δ 149.8, 146.6, 142.2, 140.0, 137.8, 137.7, 136.9, 133.5, 129.3, 128.3, 127.4, 127.0, 126.0, 120.5, 119.1, 115.8, 114.5, 112.5, 75.4, 71.2, 56.0, 53.9, 39.9, 37.3.

HRMS (ESI) m/z calculated for $C_{28}H_{30}O_3$([M+Na]$^+$): 437.2087, found: 437.2079.

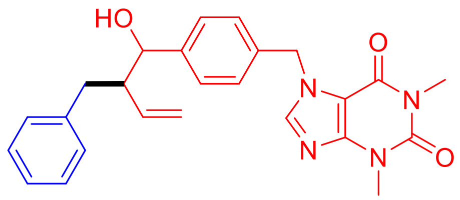

7-(4-(2-benzyl-1-hydroxybut-3-en-1-yl)benzyl)-1,3-dimethyl-3,7-dihydro-1H-purine-2,6-dione (3bv) was prepared according to General Procedure A. Purified via silica gel column chromatography 50-100% ethyl acetate/hexanes, then 0-5% MeOH/CH_2Cl_2. Yield 71%, yellow oil.

^{1}H NMR (500 MHz, $CDCl_3$) δ 7.52 (s, 1H), 7.31 (d, J = 8.5 Hz, 2H), 7.28 (d, J = 8.4 Hz, 2H), 7.22 (t, J = 7.3 Hz, 2H), 7.19 – 7.11 (m, 1H), 7.06 (d, J = 6.9 Hz, 2H), 5.68 (ddd, J = 17.2, 10.4, 8.4 Hz, 1H), 5.45 (s, 2H), 5.09 (dd, J = 10.4, 1.7 Hz, 1H), 4.93 (dd, J = 17.3, 0.8 Hz, 1H), 4.53 (d, J = 5.9 Hz, 1H), 3.55 (s, 3H), 3.37 (s, 3H), 2.71 (dd, J = 13.1, 5.4 Hz, 1H), 2.62 (tt, J = 8.4, 5.7 Hz, 1H), 2.54 (dd, J = 13.1, 8.8 Hz, 1H).

^{13}C{^{1}H} NMR (126 MHz, $CDCl_3$) δ 155.3, 151.7, 148.9, 143.5, 140.9, 139.9, 137.4, 134.6, 129.2, 128.3, 128.0, 127.5, 126.1, 119.1, 107.1, 75.0, 53.6, 50.1, 37.3, 29.9, 28.1.

HRMS (APCI) m/z calculated for $C_{25}H_{26}N_4O_3$([M+Na]$^+$): 453.1897, found: 453.1902.

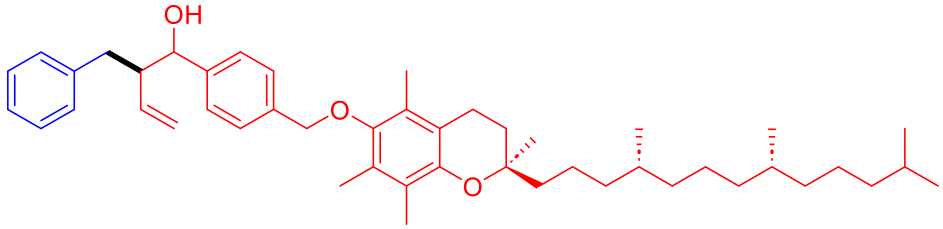

2-benzyl-1-(4-((((R)-2,5,7,8-tetramethyl-2-((4R,8R)-4,8,12-trimethyltridecyl)chroman-6-yl)oxy)methyl)phenyl)but-3-en-1-ol (3bw) was prepared according to General Procedure A. Yield 68%, yellow oil. Mixture of diastereomers

^{1}H NMR (500 MHz, $CDCl_3$) δ 7.50 (d, J = 8.1 Hz, 2H), 7.39 (d, J = 8.2 Hz, 2H), 7.27 (t, J = 7.3 Hz, 2H), 7.22 – 7.14 (m, 1H), 7.12 (d, J = 6.7 Hz, 2H), 5.75 (ddd, J = 17.2, 10.3, 8.5 Hz, 1H), 5.16 (dd, J = 10.4, 1.7 Hz, 1H), 5.01 (ddd, J = 17.2, 1.7, 0.8 Hz, 1H), 4.72 (s, 2H), 4.59 (dd, J = 6.4, 3.2 Hz, 1H), 2.78 (dd, J = 13.3, 5.3 Hz, 1H), 2.71 (tt, J = 8.6, 5.9 Hz, 1H), 2.64 – 2.54 (m, 3H), 2.23 (s, 3H), 2.18 (s, 3H), 2.16 (d, J = 3.3 Hz, 1H), 2.12 (s, 3H), 1.85 (dt, J = 14.0, 7.1 Hz, 1H), 1.78 (dt, J = 13.3, 6.5 Hz, 1H), 1.68 – 1.03 (m, 24H), 0.93 – 0.83 (m, 12H).

^{13}C{^{1}H} NMR (126 MHz, $CDCl_3$) δ 148.2, 148.1, 142.3, 140.1, 137.9, 137.6, 129.3, 128.3, 128.1, 127.9, 126.9, 126.1, 123.1, 119.1, 117.7, 75.5, 75.0, 74.6, 53.9, 40.2, 40.2, 39.5, 37.7, 37.7, 37.6, 37.6, 37.6, 37.6, 37.5, 37.5, 37.5, 37.4, 37.4, 32.9, 32.9, 32.8, 32.8, 31.5, 31.4, 28.1, 25.0, 24.9, 24.6, 24.0, 22.9, 22.8, 21.2, 21.2, 20.8, 19.9, 19.8, 19.8, 19.8, 19.8, 13.0, 12.2, 12.0.

HRMS (ESI) m/z calculated for $C_{47}H_{68}O_3$([M+Na]$^+$): 703.5061, found: 703.5046

References

(1) Lee, N. R.; Moghadam, F. A.; Braga, F. C.; Lippincott, D. J.; Zhu, B.; Gallou, F.; Lipshutz, B. H. Sustainable Palladium-Catalyzed Tsuji–Trost Reactions Enabled by Aqueous Micellar Catalysis. *Org. Lett.* **2020**, *22* (13), 4949-4954.
(2) Dai, X.-J.; Li, C.-J. En Route to a Practical Primary Alcohol Deoxygenation. *J. Am. Chem. Soc.* **2016**, *138* (16), 5433-5440.
(3) Wang, W.; Yu, Y.; Cheng, B.; Fang, H.; Zhang, X.; Qian, H.; Ma, S. Stereodefined rhodium-catalysed 1,4-H/D delivery for modular syntheses and deuterium integration. *Nat. Catal* **2021**, *4* (7), 586-594.
(4) More, J. D.; Finney, N. S. A Simple and Advantageous Protocol for the Oxidation of Alcohols with o-Iodoxybenzoic Acid (IBX). *Org. Lett.* **2002**, *4* (17), 3001-3003.
(5) Jakas, A.; Višnjevac, A.; Jerić, I. Multicomponent Approach to Homo- and Hetero-Multivalent Glycomimetics Bearing Rare Monosaccharides. *J. Org. Chem.* **2020**, *85* (5), 3766-3787.
(6) Trost, B. M.; Bunt, R. C.; Lemoine, R. C.; Calkins, T. L. Dynamic Kinetic Asymmetric Transformation of Diene Monoepoxides: A Practical Asymmetric Synthesis of Vinylglycinol, Vigabatrin, and Ethambutol. *J. Am. Chem. Soc.* **2000**, *122* (25), 5968-5976.
(7) Zhang, P.; Brozek, L. A.; Morken, J. P. Pd-Catalyzed Enantioselective Allyl−Allyl Cross-Coupling. *J. Am. Chem. Soc.* **2010**, *132* (31), 10686-10688.
(8) Brozek, L. A.; Ardolino, M. J.; Morken, J. P. Diastereocontrol in Asymmetric Allyl–Allyl Cross-Coupling: Stereocontrolled Reaction of Prochiral Allylboronates with Prochiral Allyl Chlorides. *J. Am. Chem. Soc.* **2011**, *133* (42), 16778-16781.
(9) Zhu, D.; Lv, L.; Li, C.-C.; Ung, S.; Gao, J.; Li, C.-J. Umpolung of Carbonyl Groups as Alkyl Organometallic Reagent Surrogates for Palladium-Catalyzed Allylic Alkylation. *Angew Chem. Int. Ed.* **2018**, *57* (50), 16520-16524.
(10) Lv, L.; Li, C.-J. Palladium-Catalyzed Defluorinative Alkylation of gem-Difluorocyclopropanes: Switching Regioselectivity via Simple Hydrazones. *Angew Chem. Int. Ed.* **2021**, *60* (23), 13098-13104.
(11) Pandit, S.; Adhikari, A. S.; Majumdar, N. Iridium-Catalyzed Enantioselective Ring Opening of Alkenyl Oxiranes by Unactivated Carboxylic Acids. *Org. Lett.* **2022**, *24* (40), 7388-7393.
(12) Li, K.; Deng, X.-M.; Tang, Y. A facile tetrahydrothiophene-catalyzed ylide route to vinyloxiranes. *Chem comm* **2003**, (16), 2074-2075.
(13) Okuma, K. Q., Yuxuan; Nagahora, Noriyoshi. Novel reaction course of thiiranes to vinyloxiranes: reaction of benzyne with thiiranes and aldehydes. *Heterocycles* **2021**, *102* (7), 1294-1300.

NMR spectra collection for compounds

^{1}H NMR (500 MHz, $CDCl_3$) spectrum of **3R,5R,8R,9S,10S,13R,14S,17R)-10,13-dimethyl-17-((2R)-4-(3-vinyloxiran-2-yl)butan-2-yl)hexadecahydro-1H-cyclopenta[a]phenanthren-3-ol (2k)**

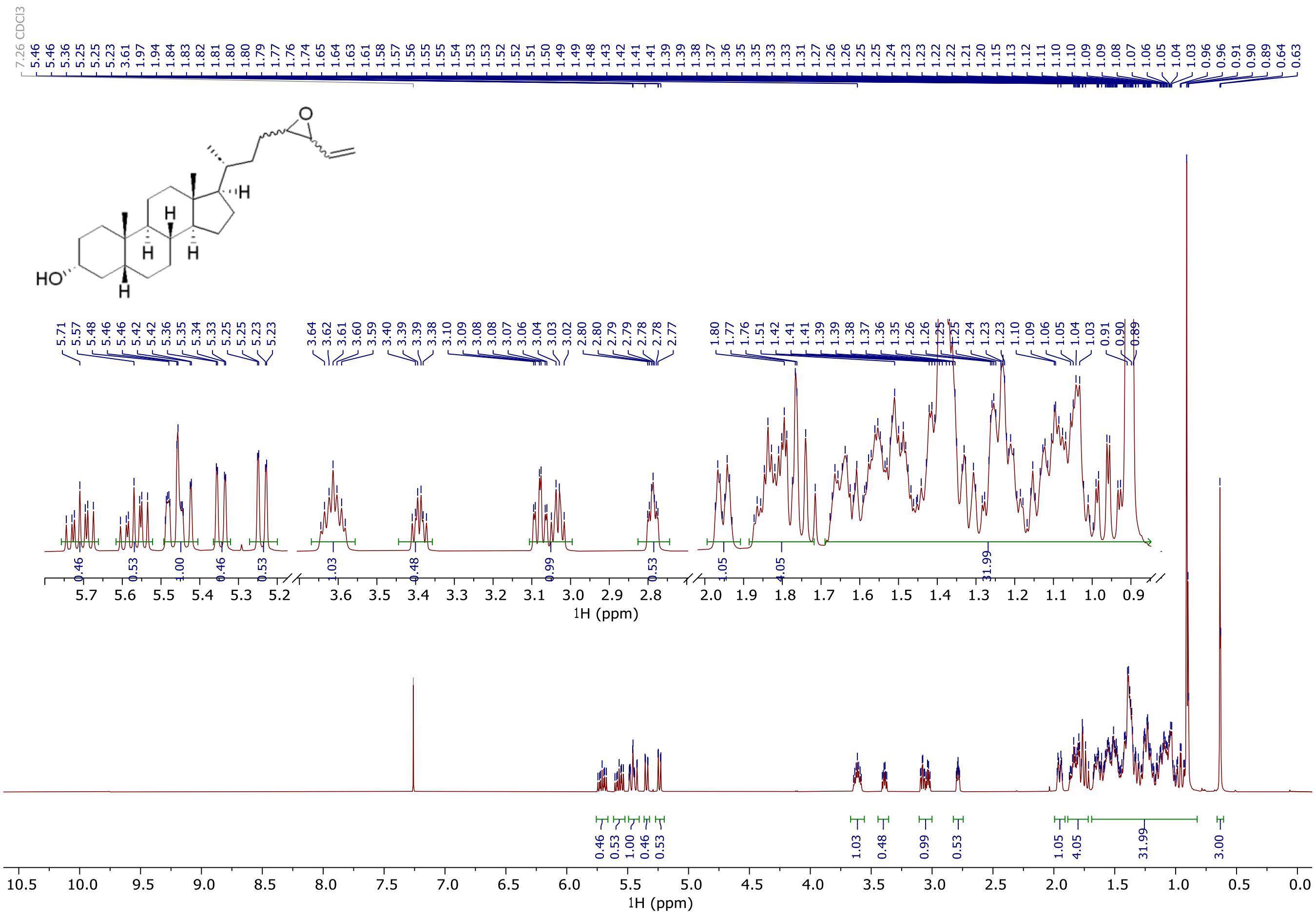

^{13}C{^{1}H} NMR (126 MHz, $CDCl_3$) spectrum of **3R,5R,8R,9S,10S,13R,14S,17R)-10,13-dimethyl-17-((2R)-4-(3-vinyloxiran-2-yl)butan-2-yl)hexadecahydro-1H-cyclopenta[a]phenanthren-3-ol (2k)**

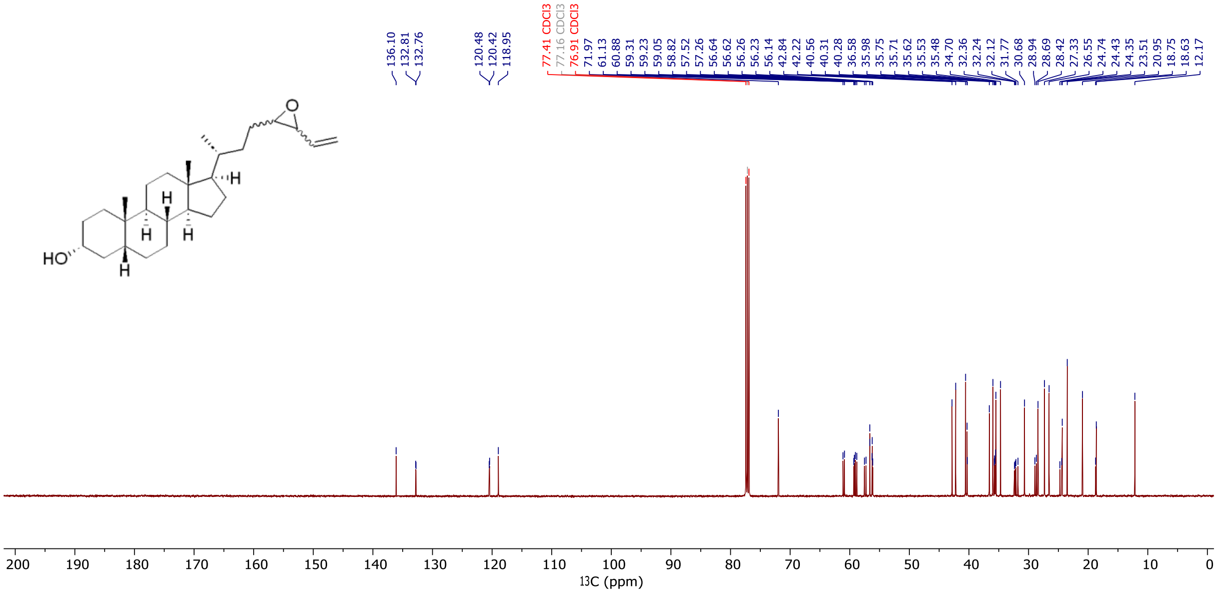

^{1}H NMR (500 MHz, $CDCl_3$) spectrum of **3aR,5aS,8aS,8bR)-2,2,7,7-tetramethyl-5-(3-vinyloxiran-2-yl)tetrahydro-5H-bis([1,3]dioxolo)[4,5-b:4',5'-d]pyran (2l)**

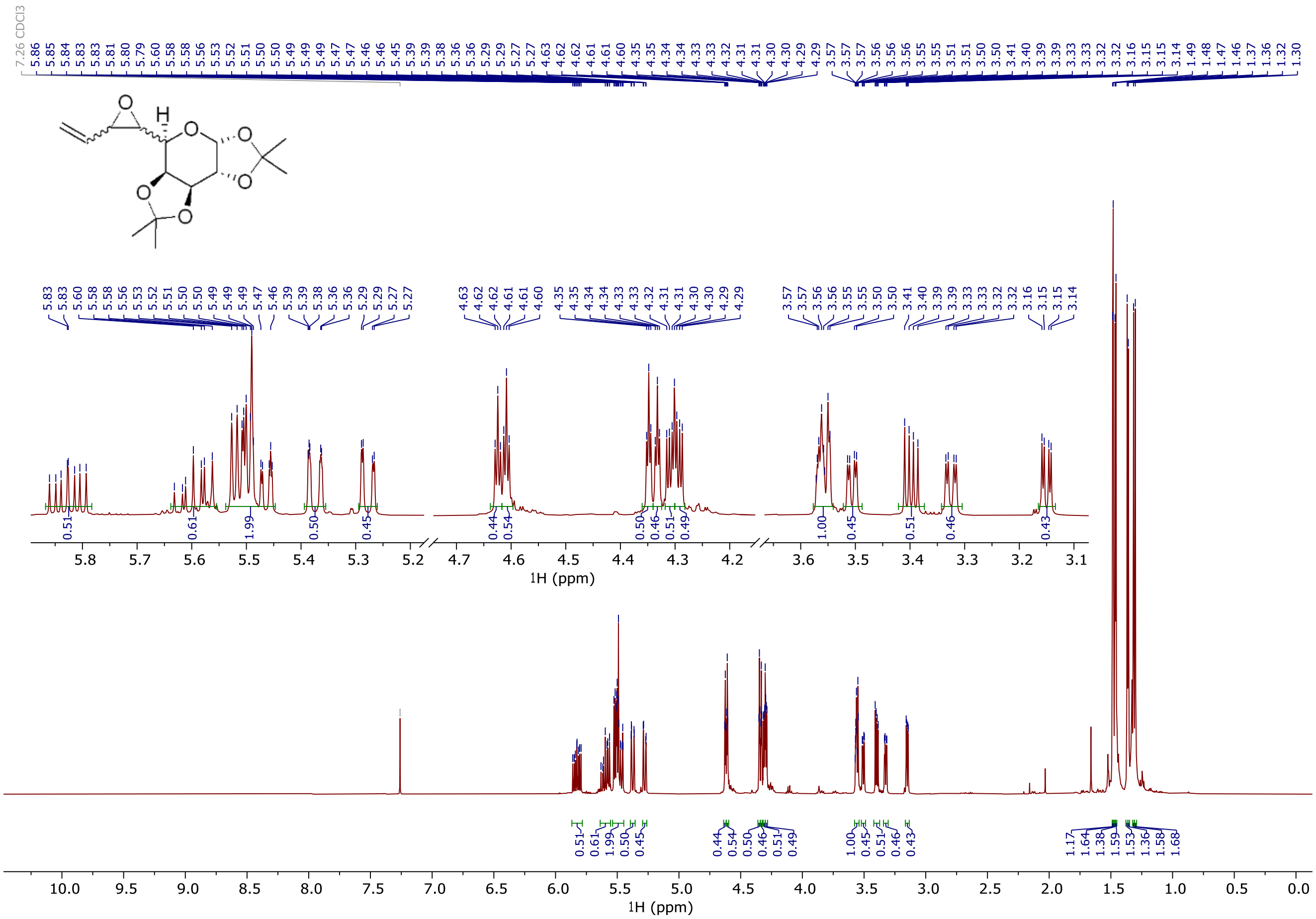

^{13}C{^{1}H} NMR (126 MHz, $CDCl_3$) spectrum of **3aR,5aS,8aS,8bR)-2,2,7,7-tetramethyl-5-(3-vinyloxiran-2-yl)tetrahydro-5H-bis([1,3]dioxolo)[4,5-b:4',5'-d]pyran (2l)**

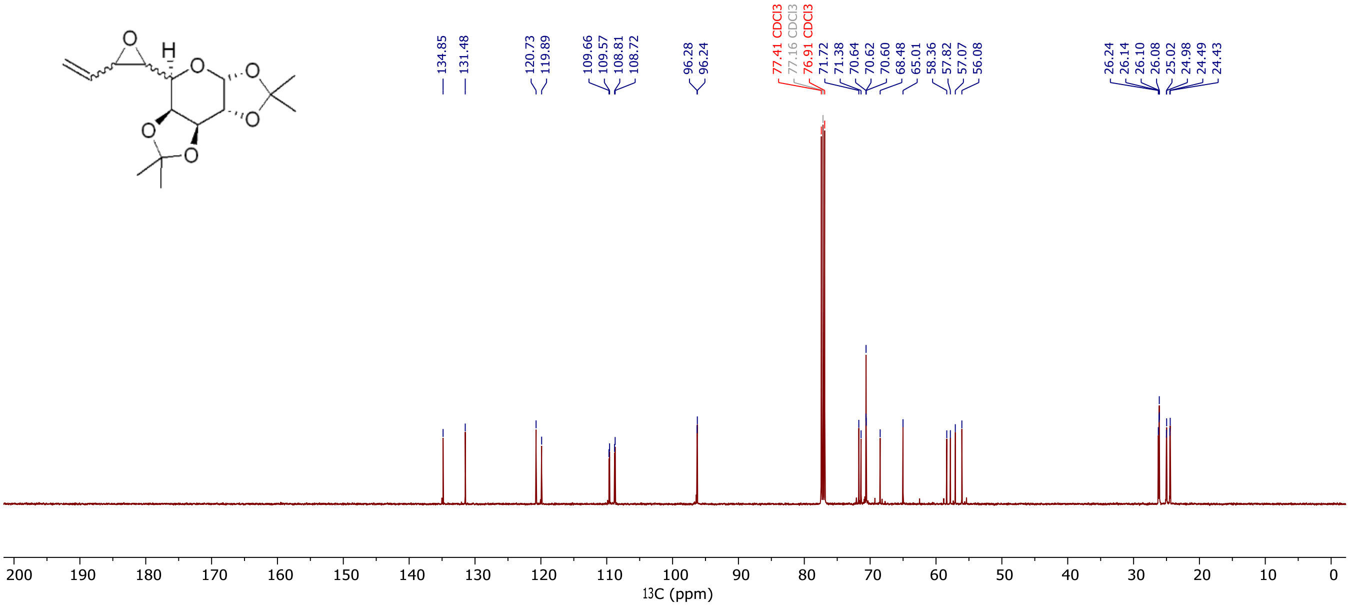

^{1}H NMR (500 MHz, $CDCl_3$) spectrum of **1,3-dimethyl-7-(4-(3-vinyloxiran-2-yl)benzyl)-3,7-dihydro-1H-purine-2,6-dione** (**2m**)

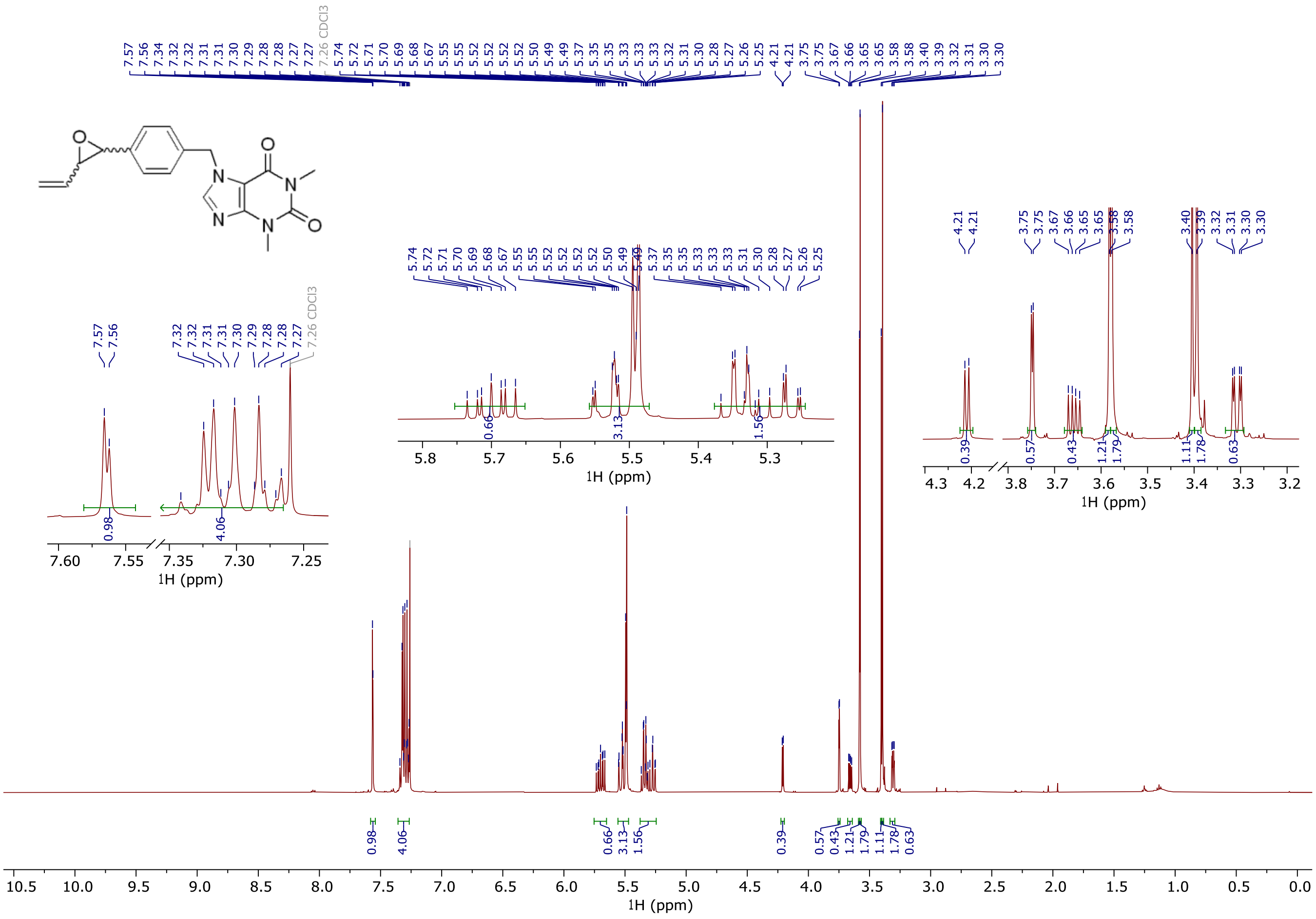

$^{13}C\{^{1}H\}$ NMR (126 MHz, $CDCl_3$) spectrum of **1,3-dimethyl-7-(4-(3-vinyloxiran-2-yl)benzyl)-3,7-dihydro-1H-purine-2,6-dione** (**2m**)

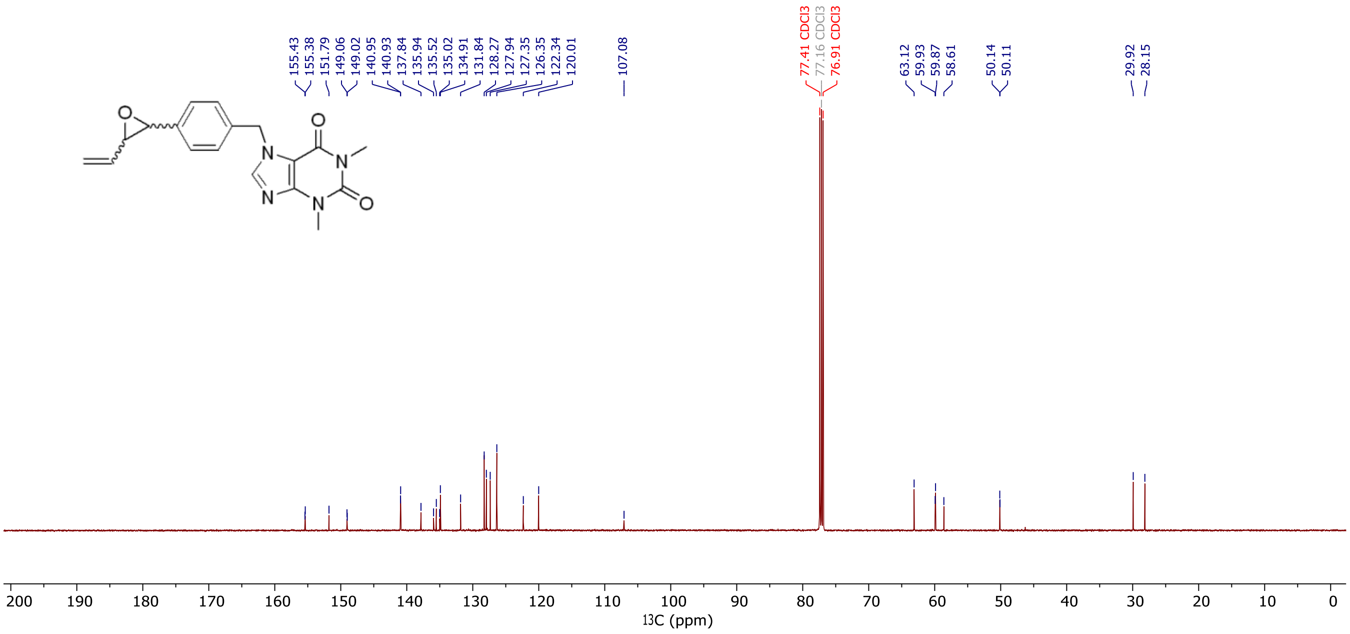

^{1}H NMR (500 MHz, $CDCl_3$) spectrum of **2-(4-((4-allyl-2-methoxyphenoxy)methyl)phenyl)-3-vinyloxirane (2n)**

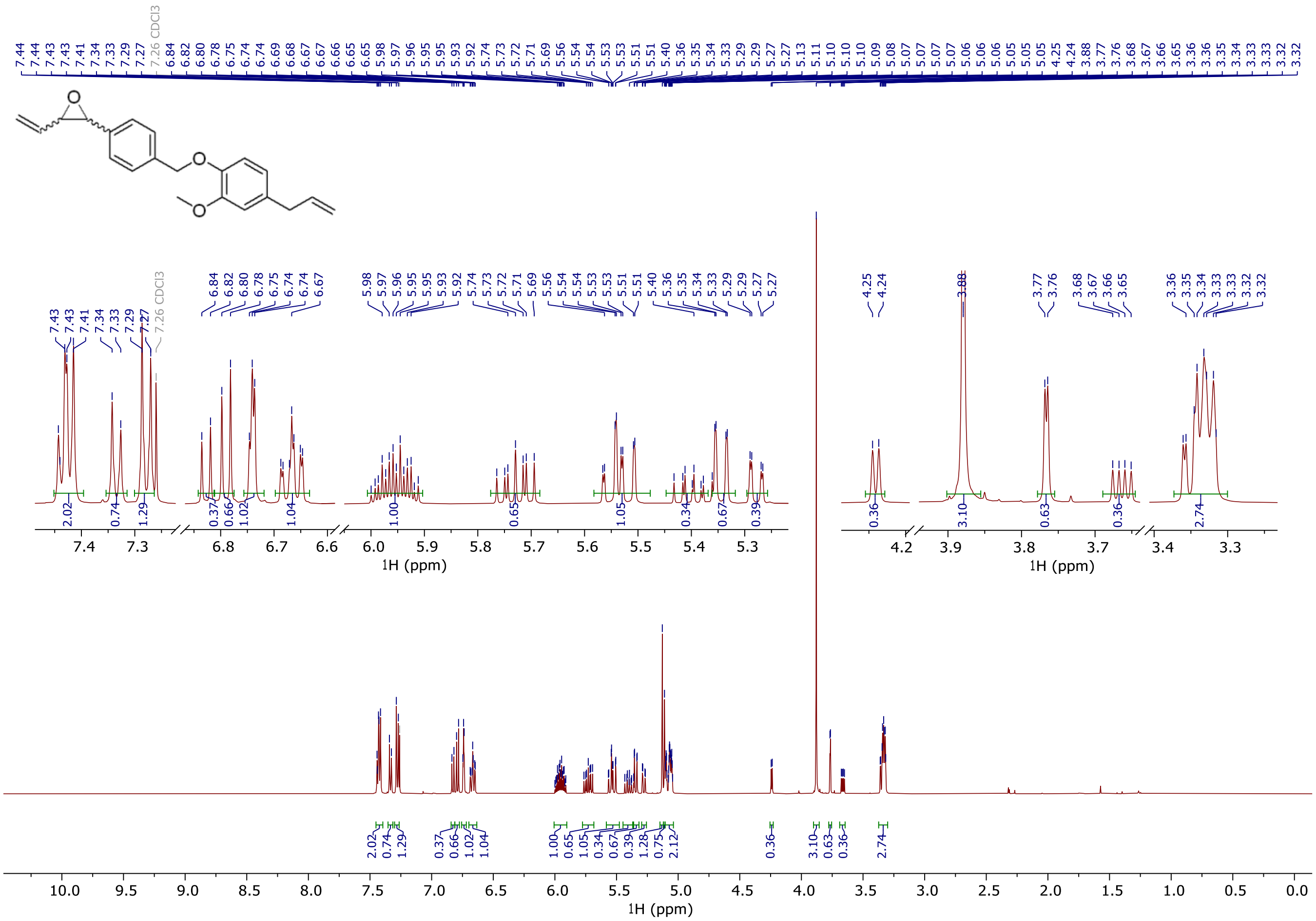

^{13}C{^{1}H} NMR (126 MHz, $CDCl_3$) spectrum of **2-(4-((4-allyl-2-methoxyphenoxy)methyl)phenyl)-3-vinyloxirane (2n)**

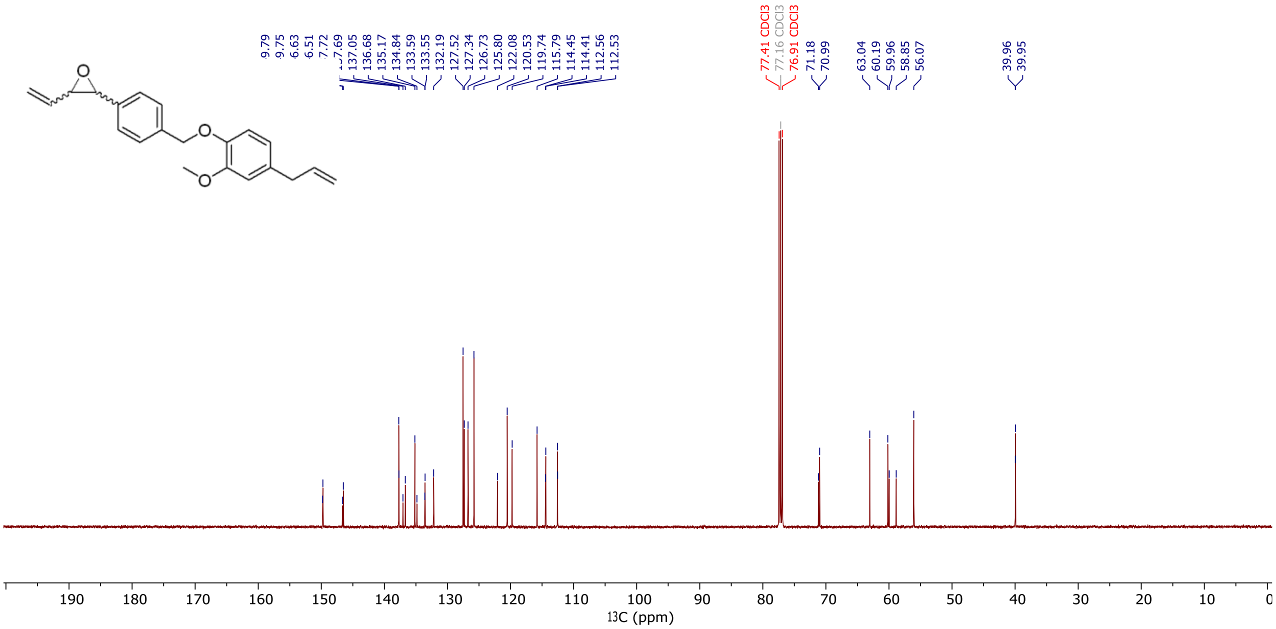

^{1}H NMR (500 MHz, $CDCl_3$) spectrum of **(2R)-2,5,7,8-tetramethyl-2-((4R,8R)-4,8,12-trimethyltridecyl)-6-((4-(3-vinyloxiran-2-yl)benzyl)oxy)chromane** (**2o**)

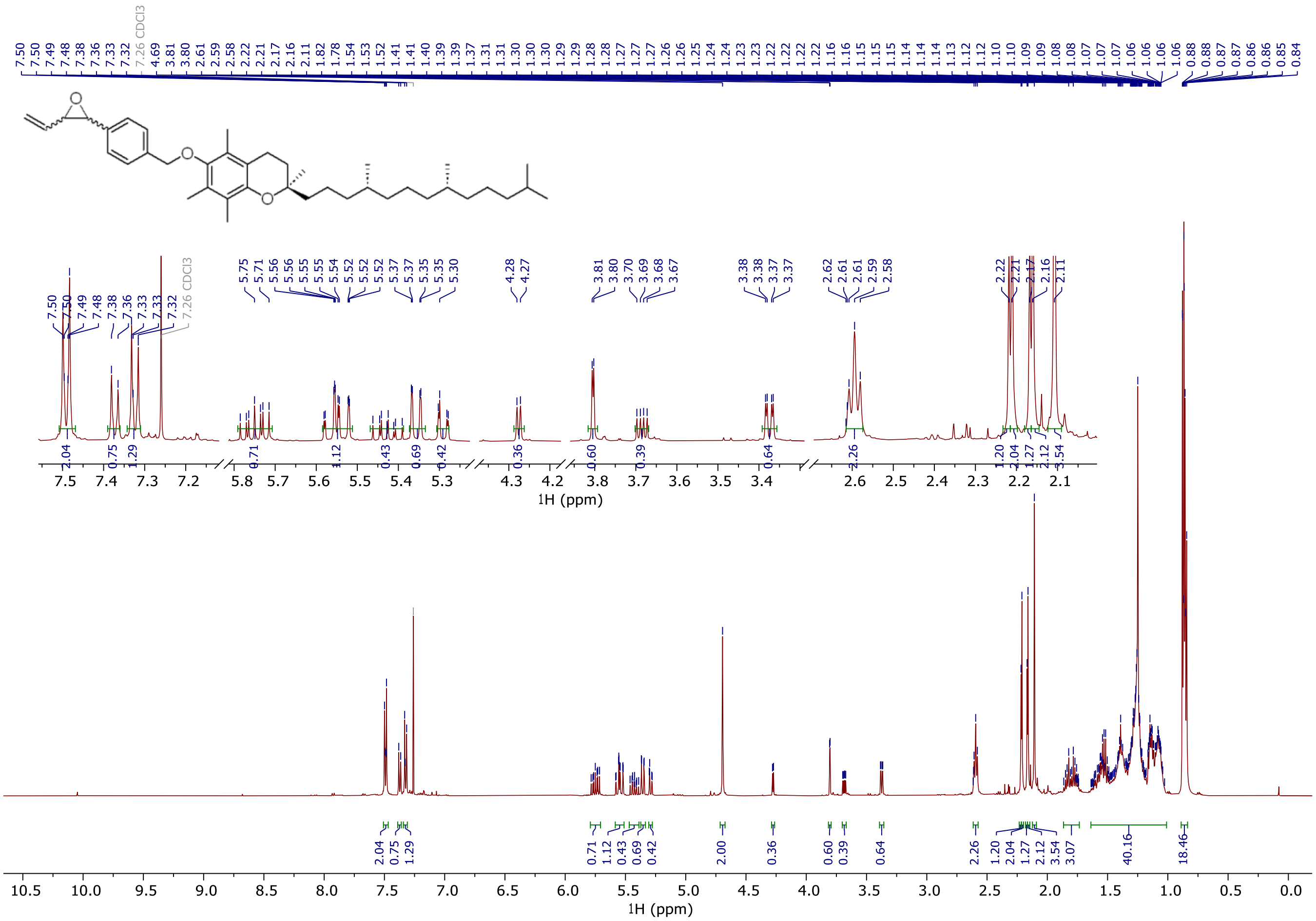

$^{13}C\{^{1}H\}$ NMR (126 MHz, $CDCl_3$) spectrum of **(2R)-2,5,7,8-tetramethyl-2-((4R,8R)-4,8,12-trimethyltridecyl)-6-((4-(3-vinyloxiran-2-yl)benzyl)oxy)chromane** (**2o**)

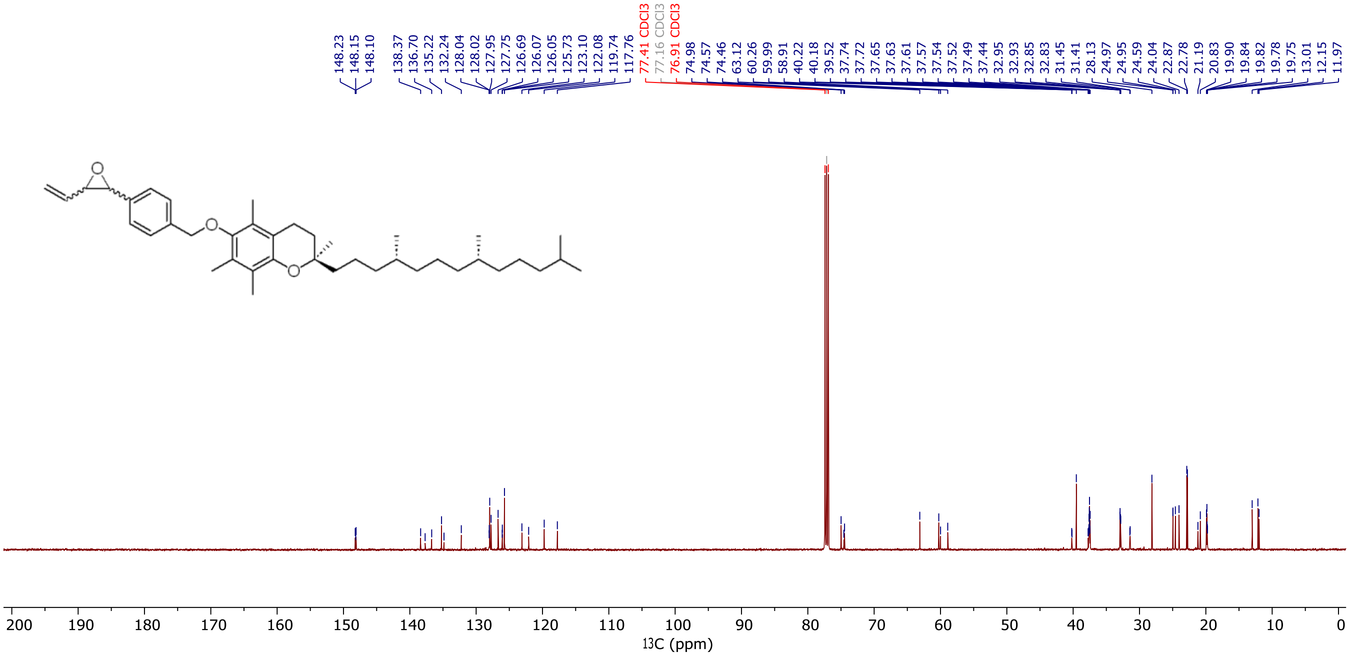

^{1}H NMR (500 MHz, $CDCl_3$) spectrum of **2-benzylbut-3-en-1-ol (3aa)**

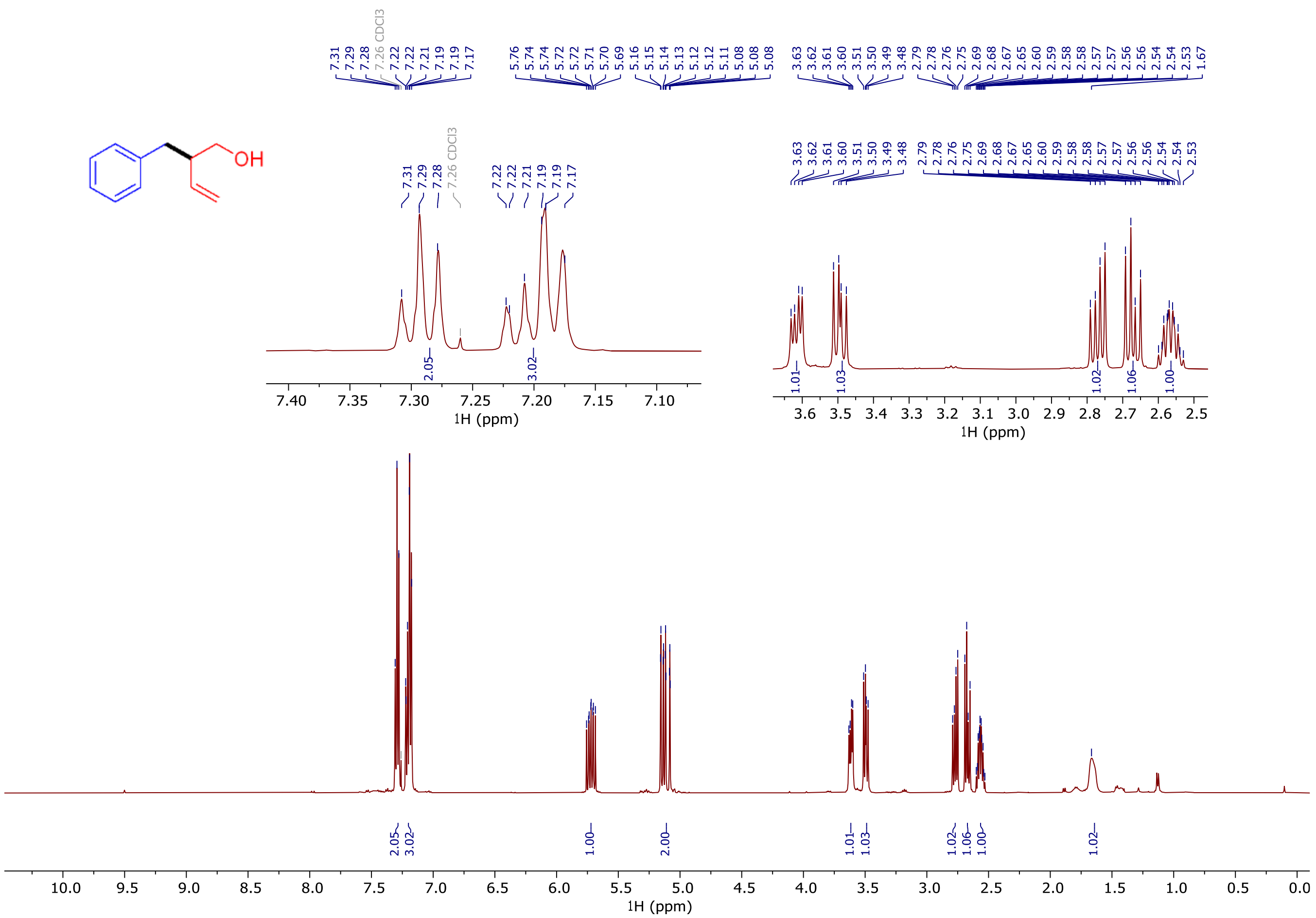

$^{13}C\{^{1}H\}$ NMR (126 MHz, $CDCl_3$) spectrum of **2-benzylbut-3-en-1-ol (3aa)**

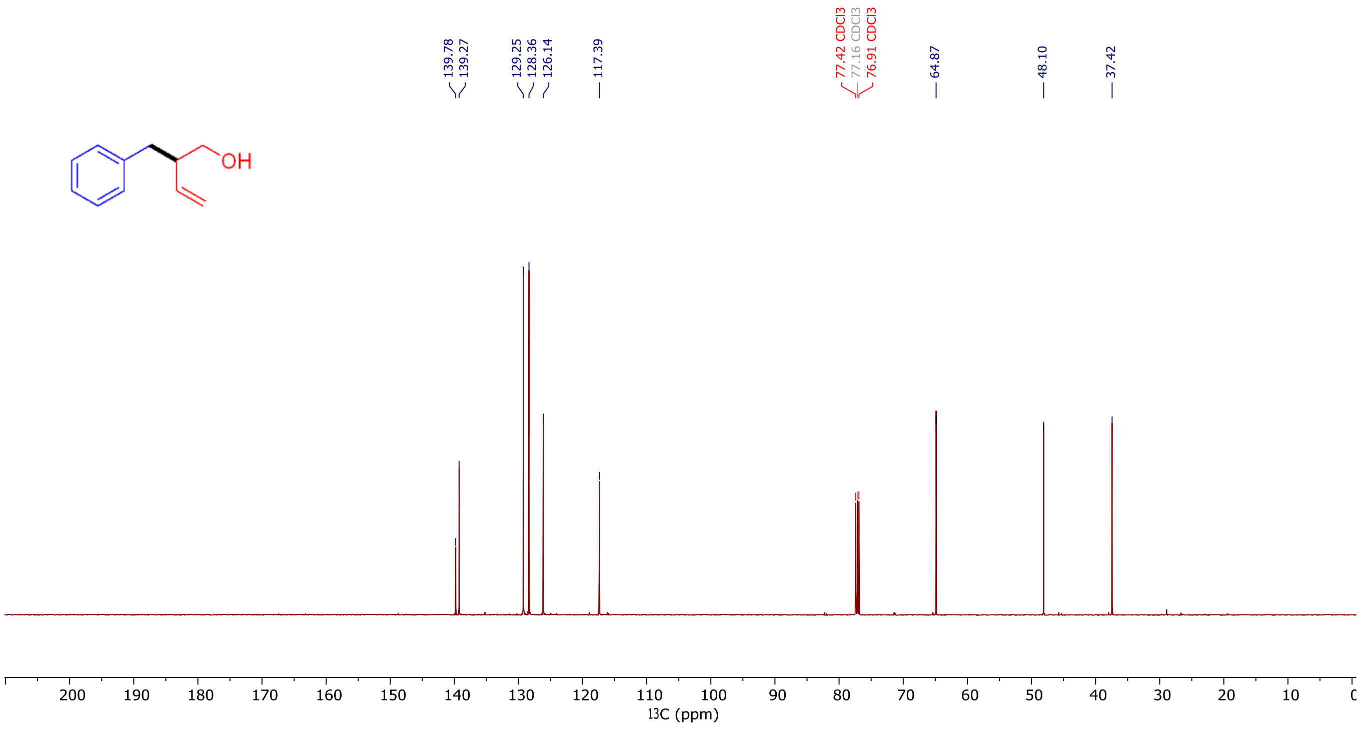

^{1}H NMR (500 MHz, $CDCl_3$) spectrum of **2-(4-bromobenzyl)but-3-en-1-ol (3ab)**

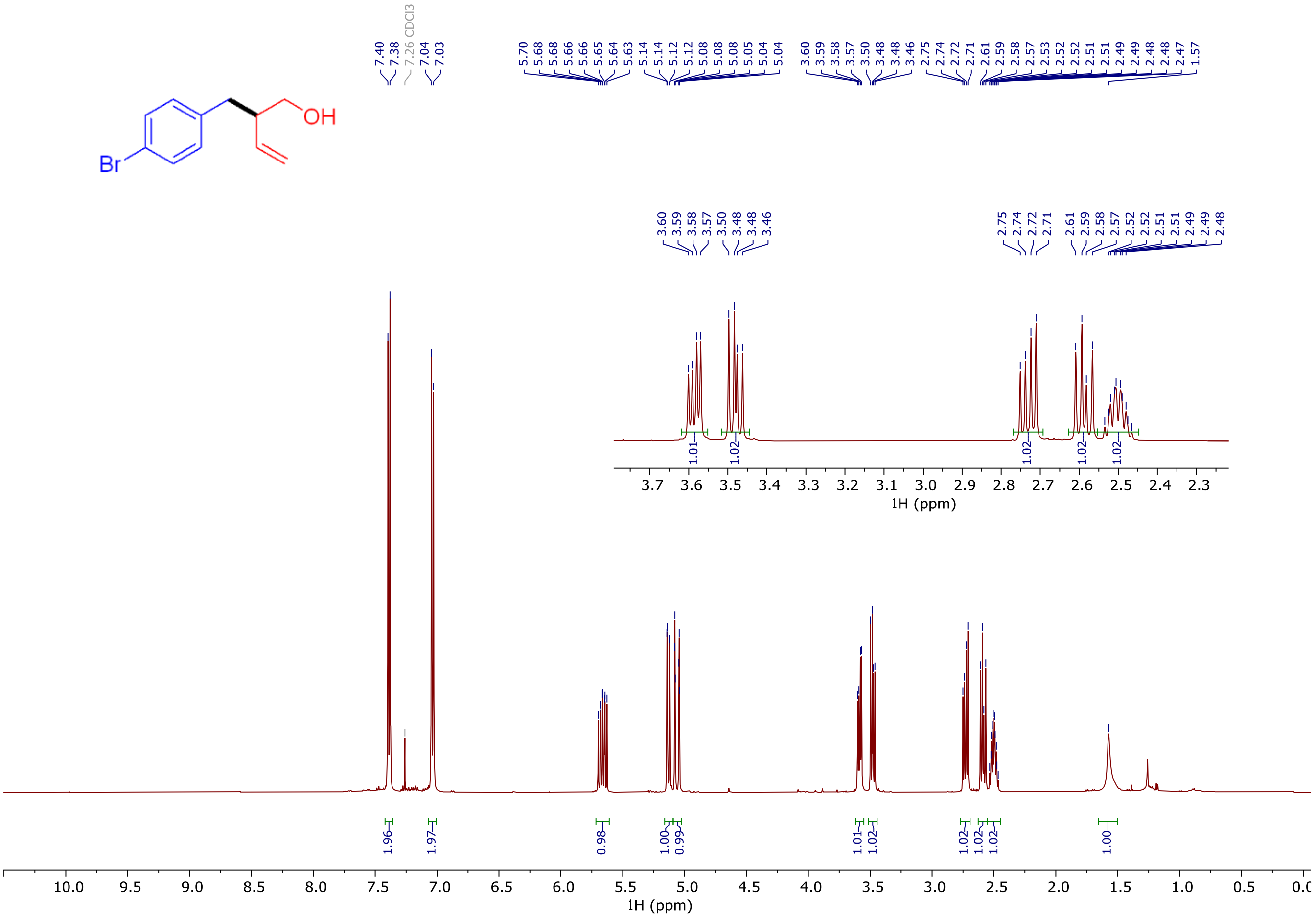

^{13}C{^{1}H} NMR (126 MHz, $CDCl_3$) spectrum of **2-(4-bromobenzyl)but-3-en-1-ol (3ab)**

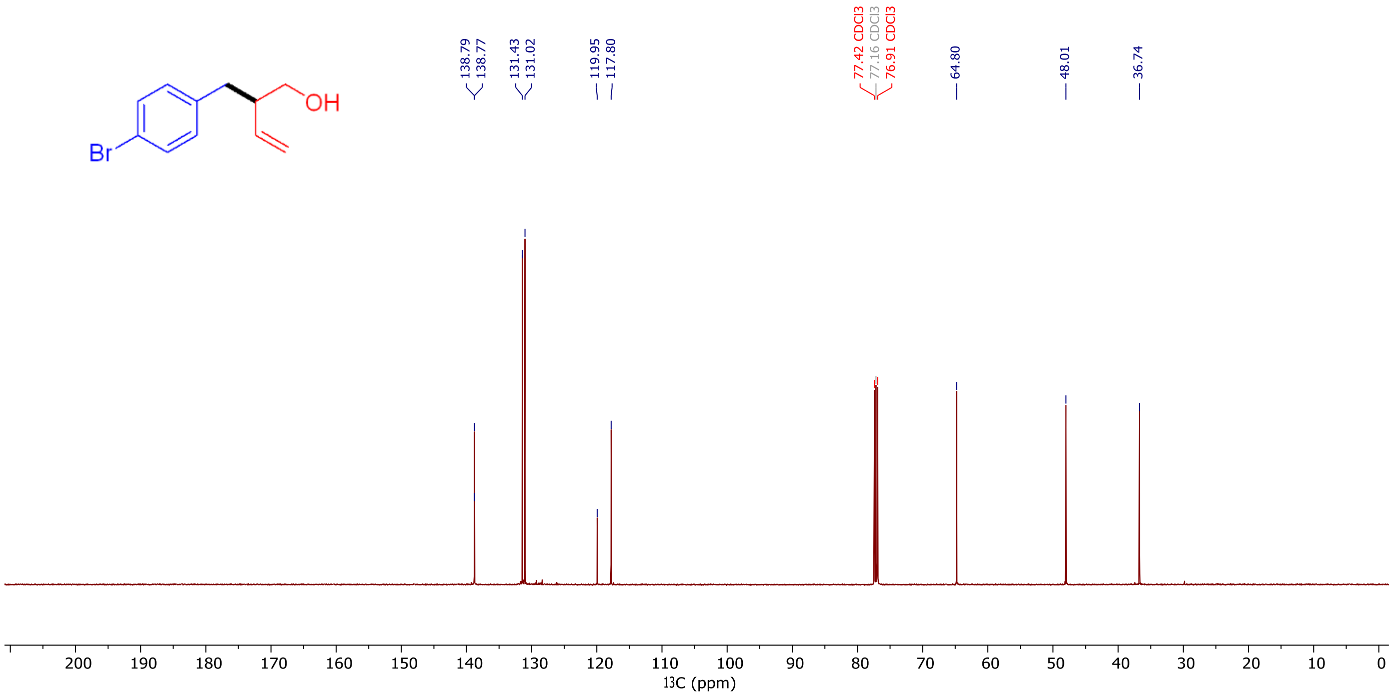

^{1}H NMR (500 MHz, $CDCl_3$) spectrum of **2-(4-fluorobenzyl)but-3-en-1-ol (3ac)**

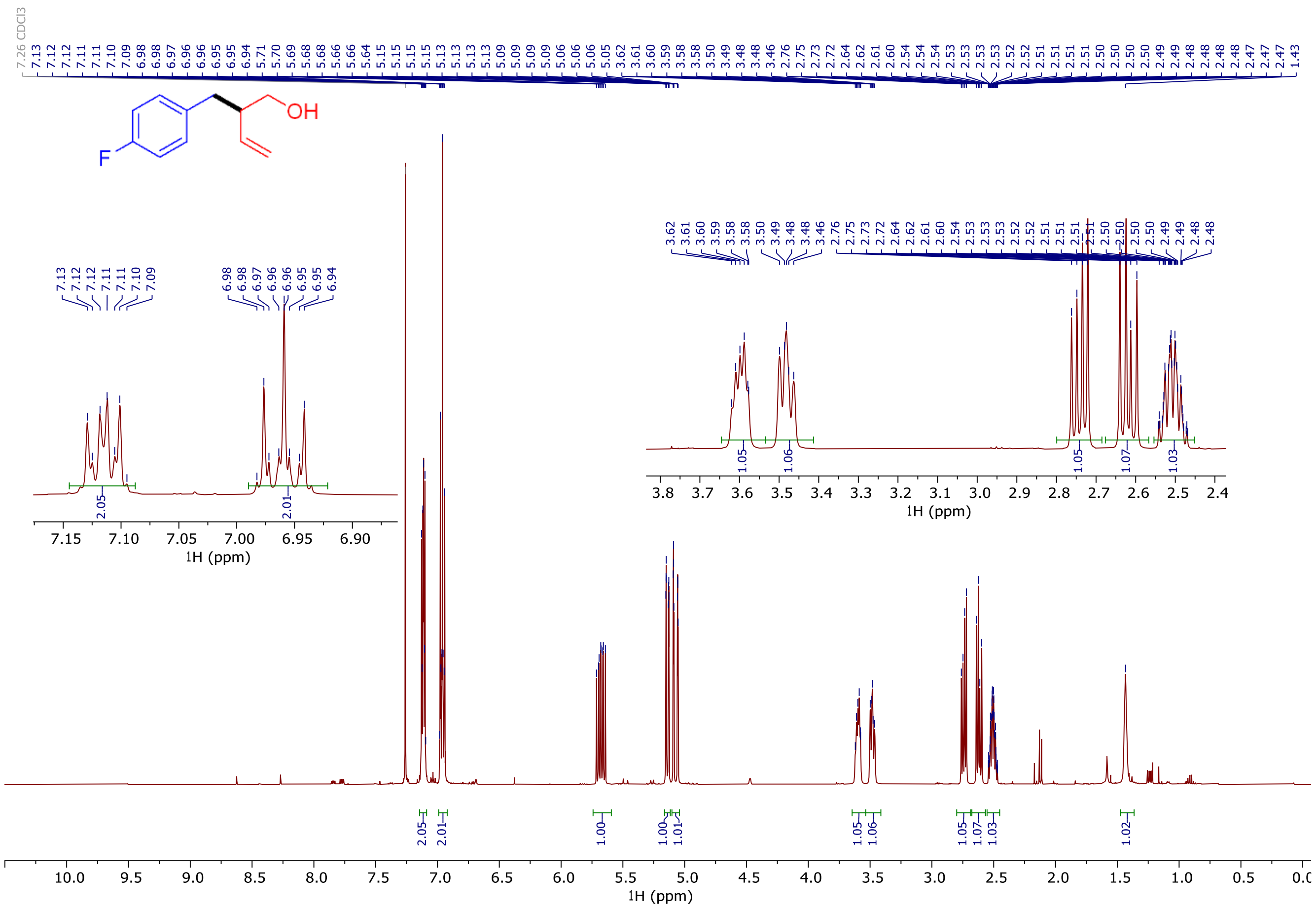

$^{13}C\{^{1}H\}$ NMR (126 MHz, $CDCl_3$) spectrum of **2-(4-fluorobenzyl)but-3-en-1-ol (3ac)**

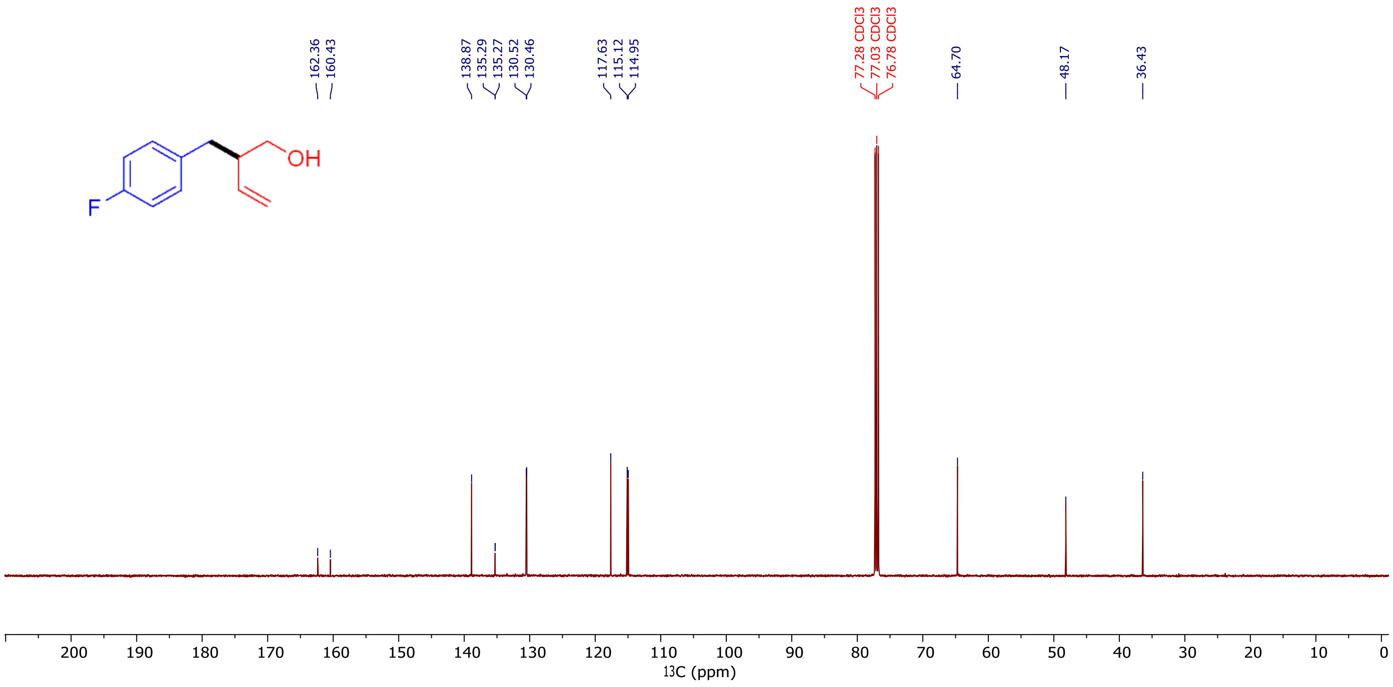

^{19}F NMR (471 MHz, $CDCl_3$) spectrum of **2-(4-fluorobenzyl)but-3-en-1-ol (3ac)**

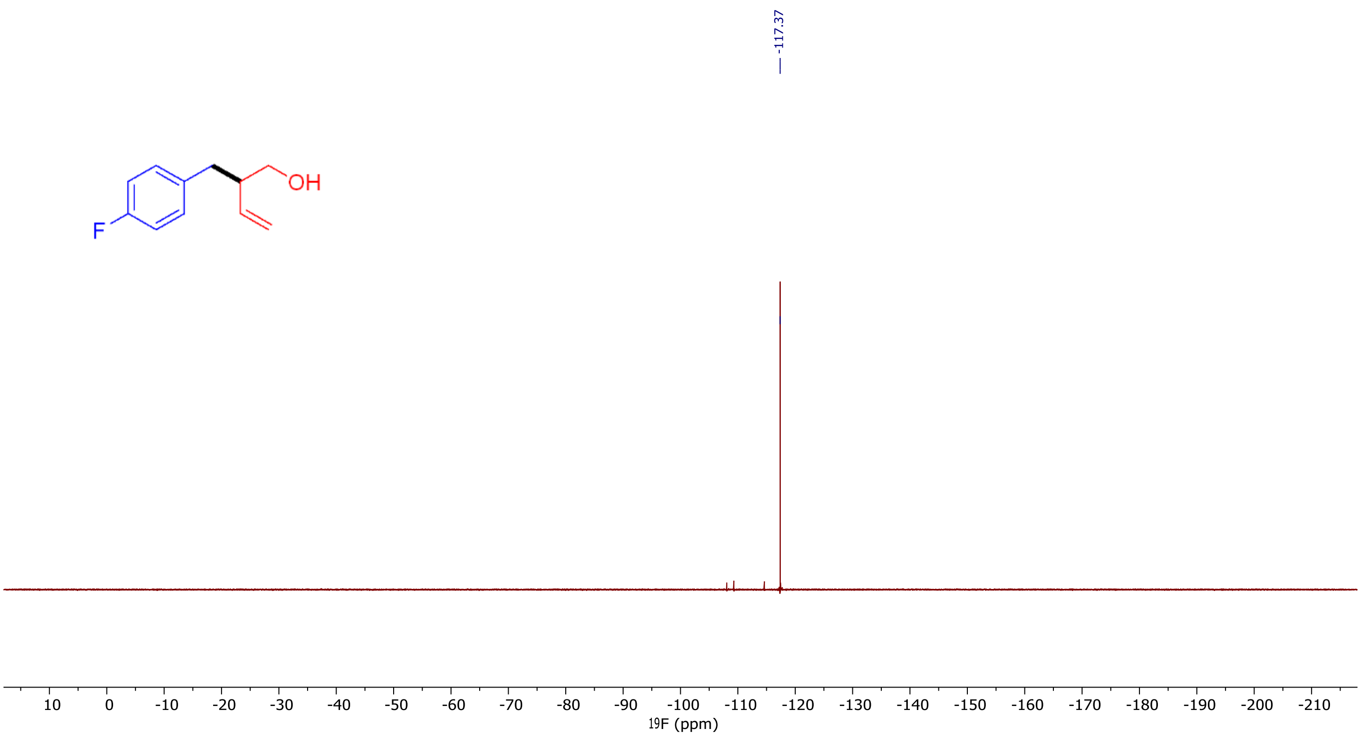

^{1}H NMR (500 MHz, $CDCl_3$) spectrum of **2-(4-chlorobenzyl)but-3-en-1-ol (3ad)**

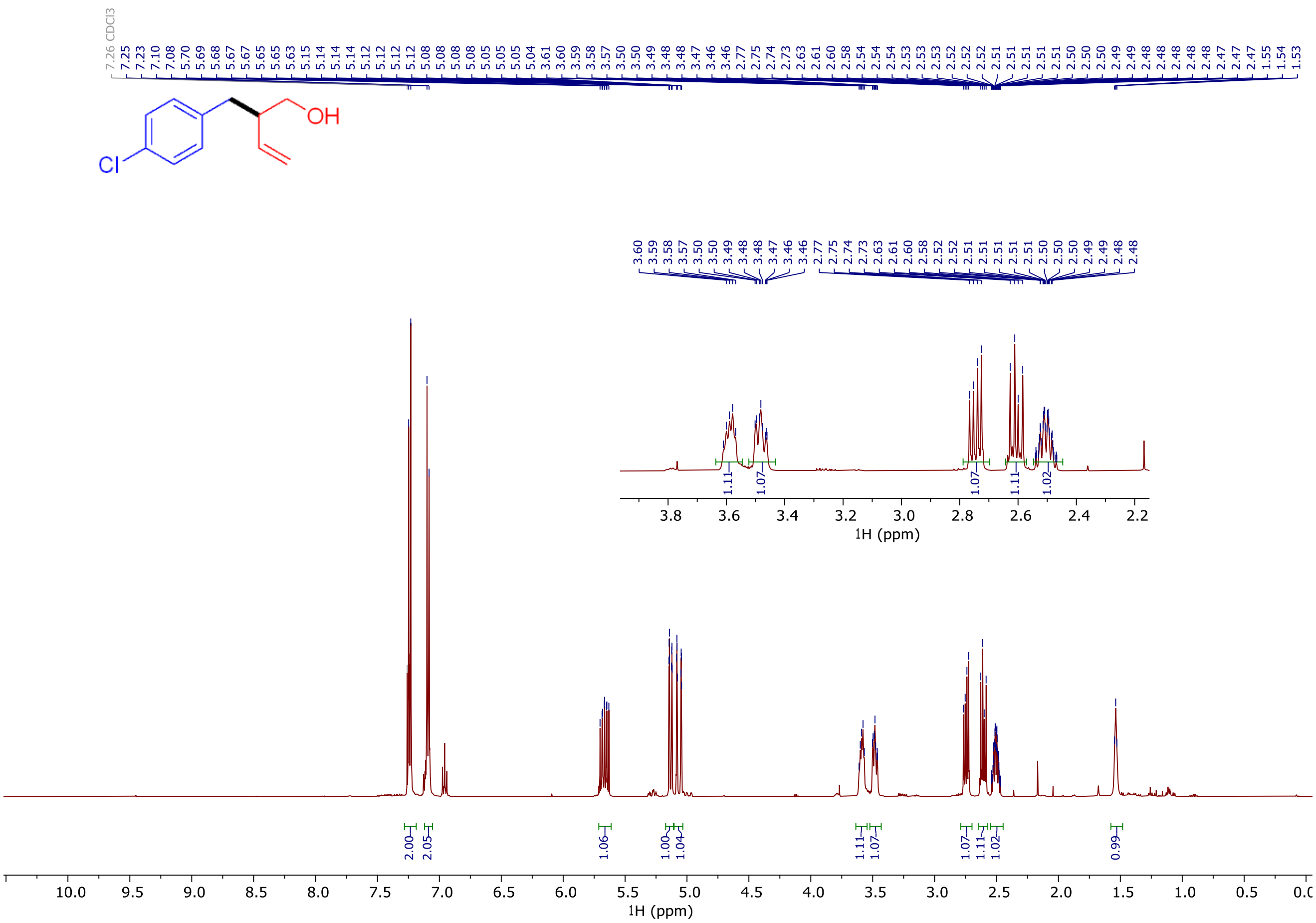

$^{13}C\{^{1}H\}$ NMR (126 MHz, $CDCl_3$) spectrum of **2-(4-chlorobenzyl)but-3-en-1-ol (3ad)**

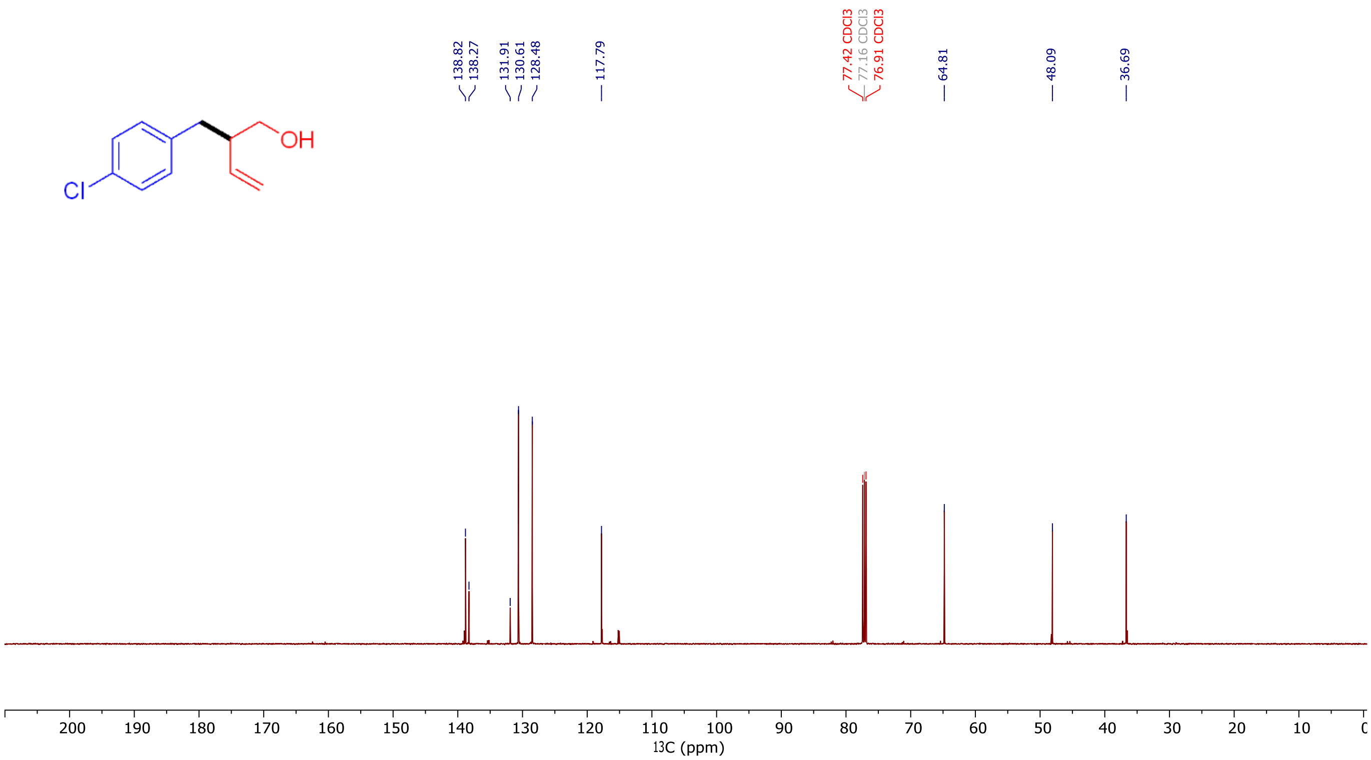

^{1}H NMR (500 MHz, $CDCl_3$) spectrum of **4-(2-(hydroxymethyl)but-3-en-1-yl)benzonitrile (3ae)**

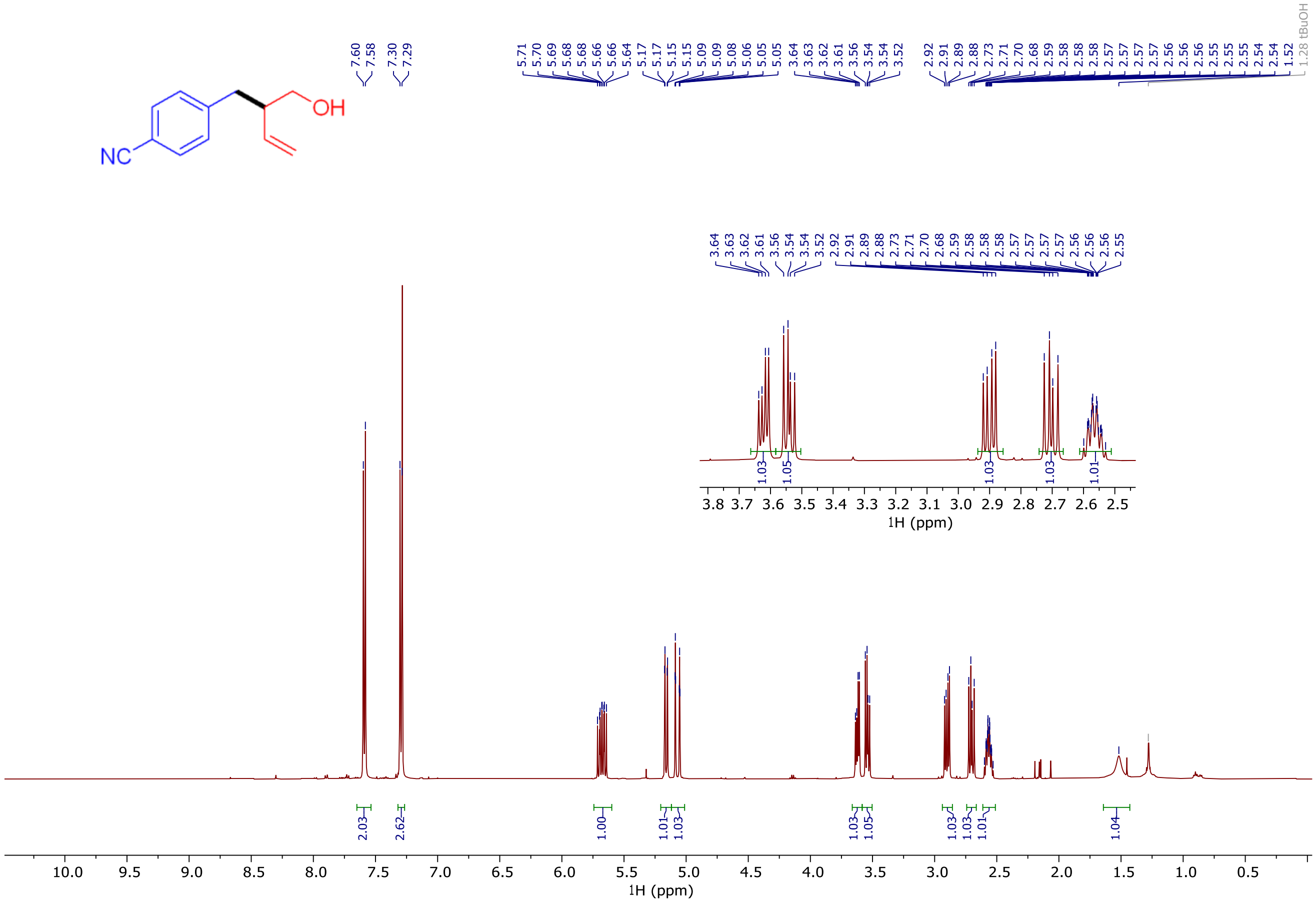

$^{13}C\{^{1}H\}$ NMR (126 MHz, $CDCl_3$) spectrum of **4-(2-(hydroxymethyl)but-3-en-1-yl)benzonitrile (3ae)**

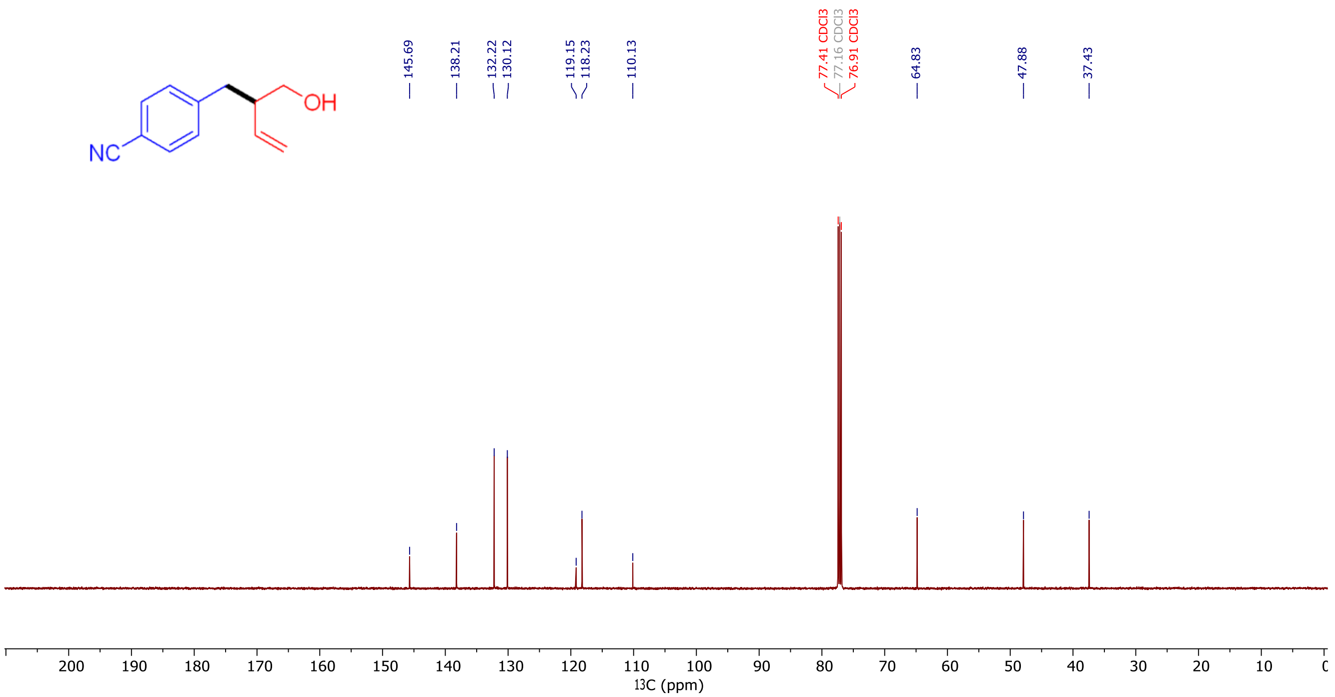

^{1}H NMR (500 MHz, $CDCl_3$) spectrum of **2-(4-(trifluoromethyl)benzyl)but-3-en-1-ol (3af)**

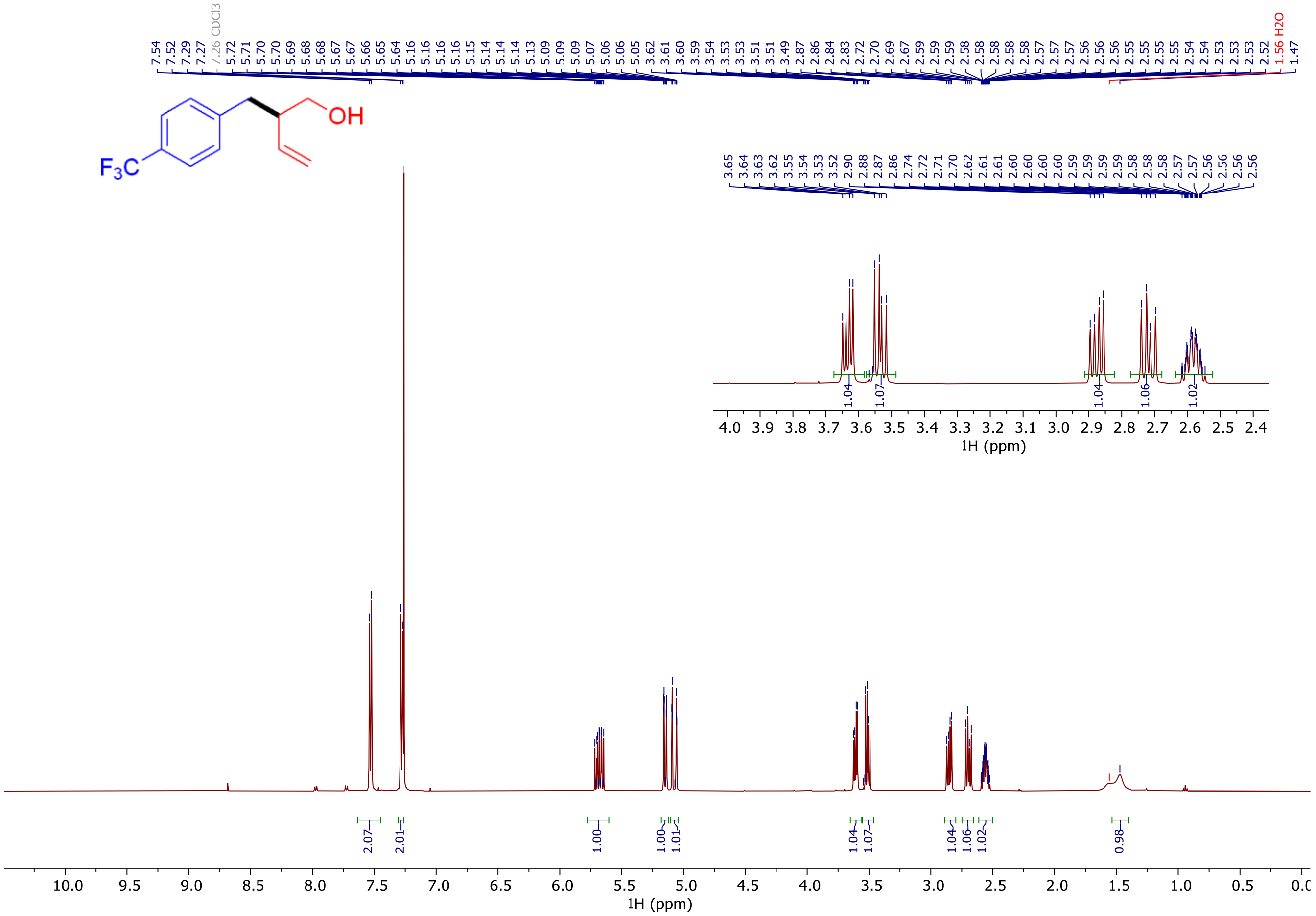

$^{13}C\{^{1}H\}$ NMR (126 MHz, $CDCl_3$) spectrum of **2-(4-(trifluoromethyl)benzyl)but-3-en-1-ol (3af)**

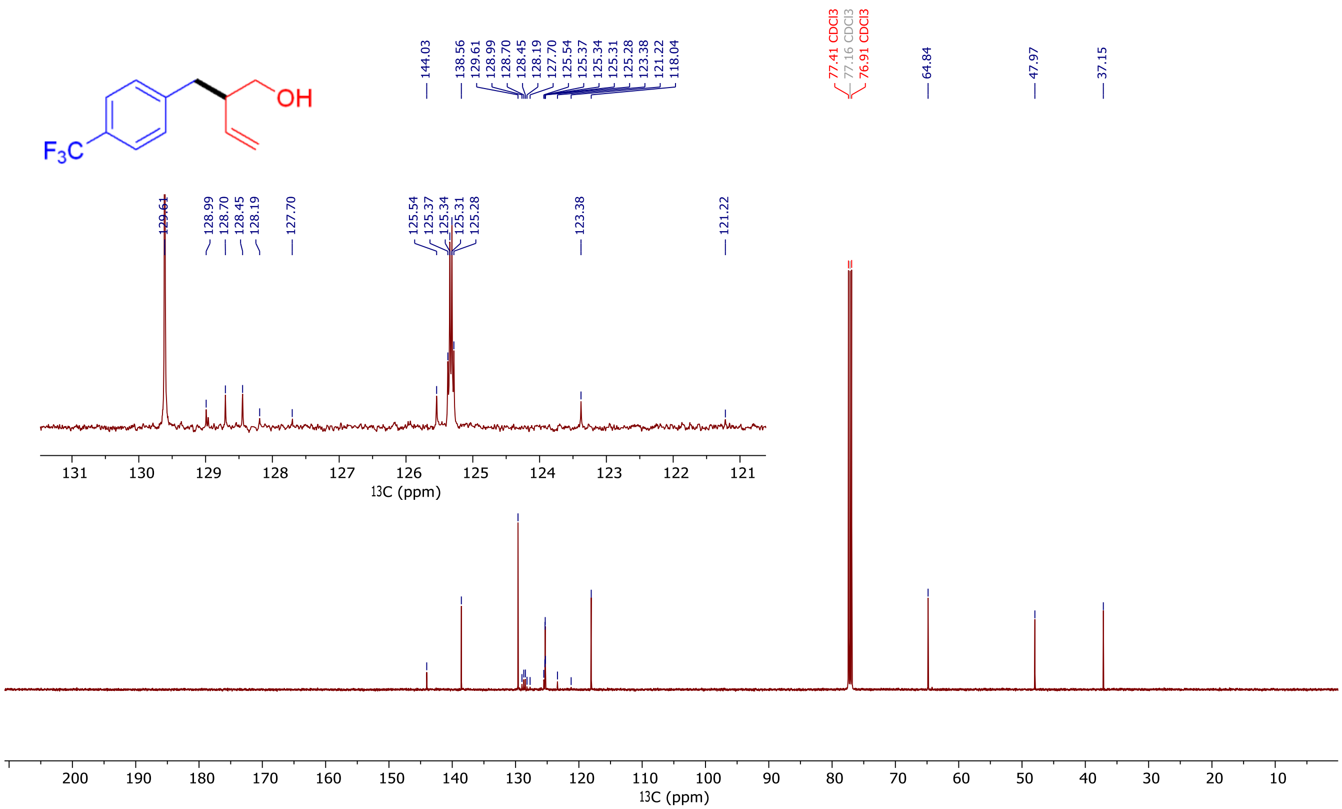

^{19}F NMR (471 MHz, $CDCl_3$) spectrum of **2-(4-(trifluoromethyl)benzyl)but-3-en-1-ol (3af)**

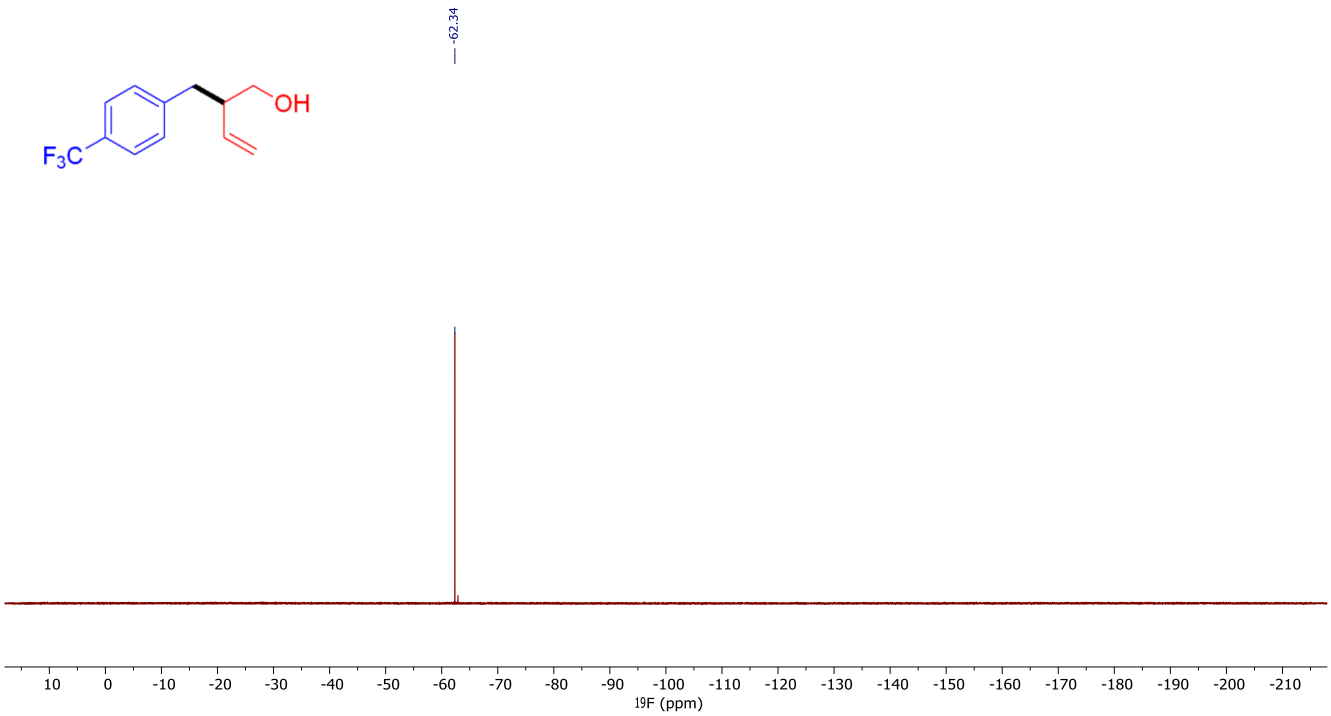

^{1}H NMR (500 MHz, $CDCl_3$) spectrum of **2-([1,1'-biphenyl]-4-ylmethyl)but-3-en-1-ol (3ag)**

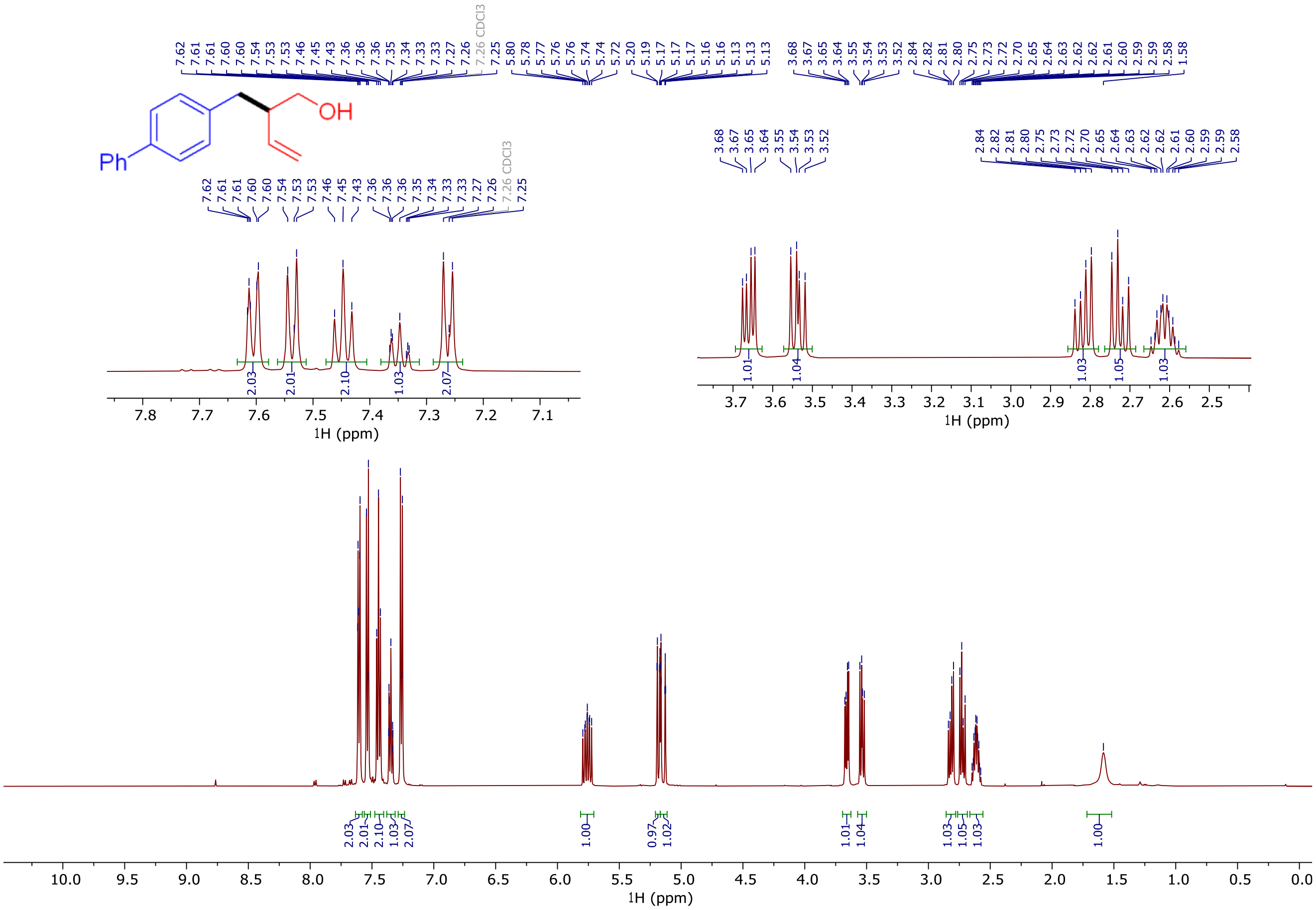

^{13}C{^{1}H} NMR (126 MHz, $CDCl_3$) spectrum of **2-([1,1'-biphenyl]-4-ylmethyl)but-3-en-1-ol (3ag)**

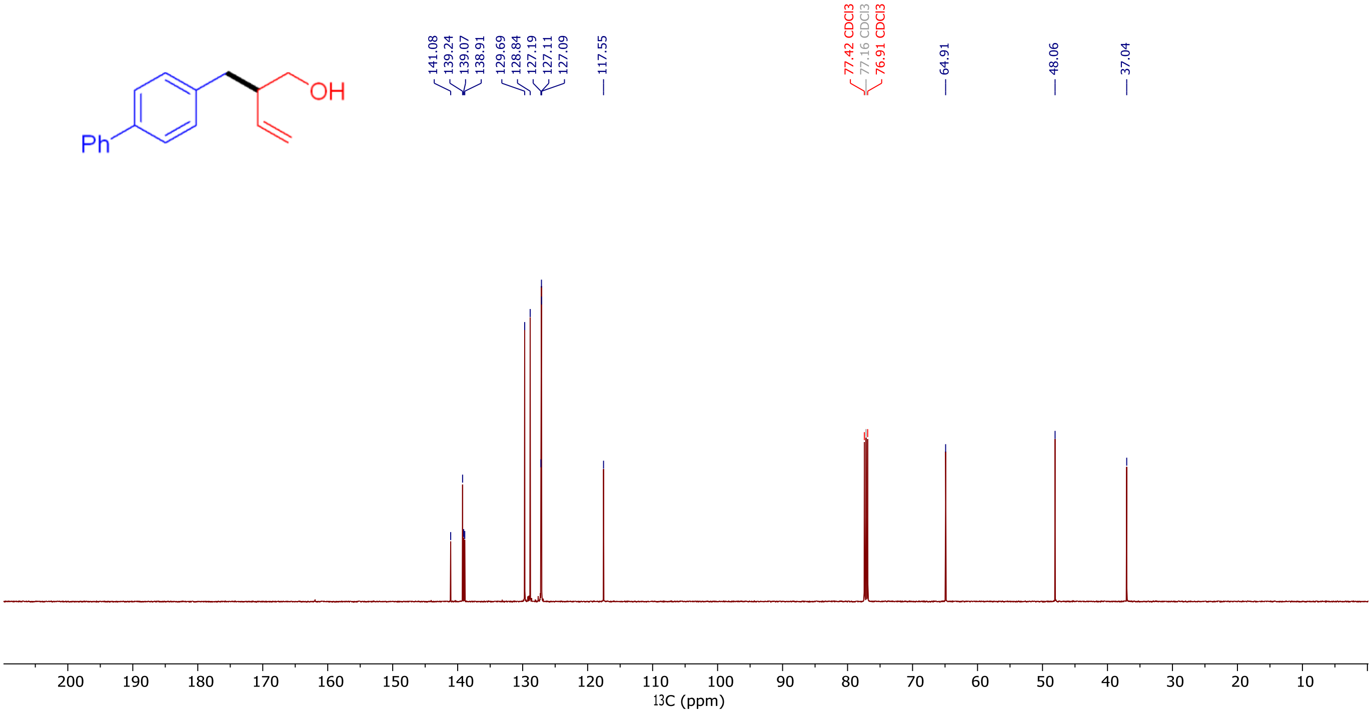

^{1}H NMR (500 MHz, $CDCl_3$) spectrum of **2-(4-methylbenzyl)but-3-en-1-ol (3ah)**

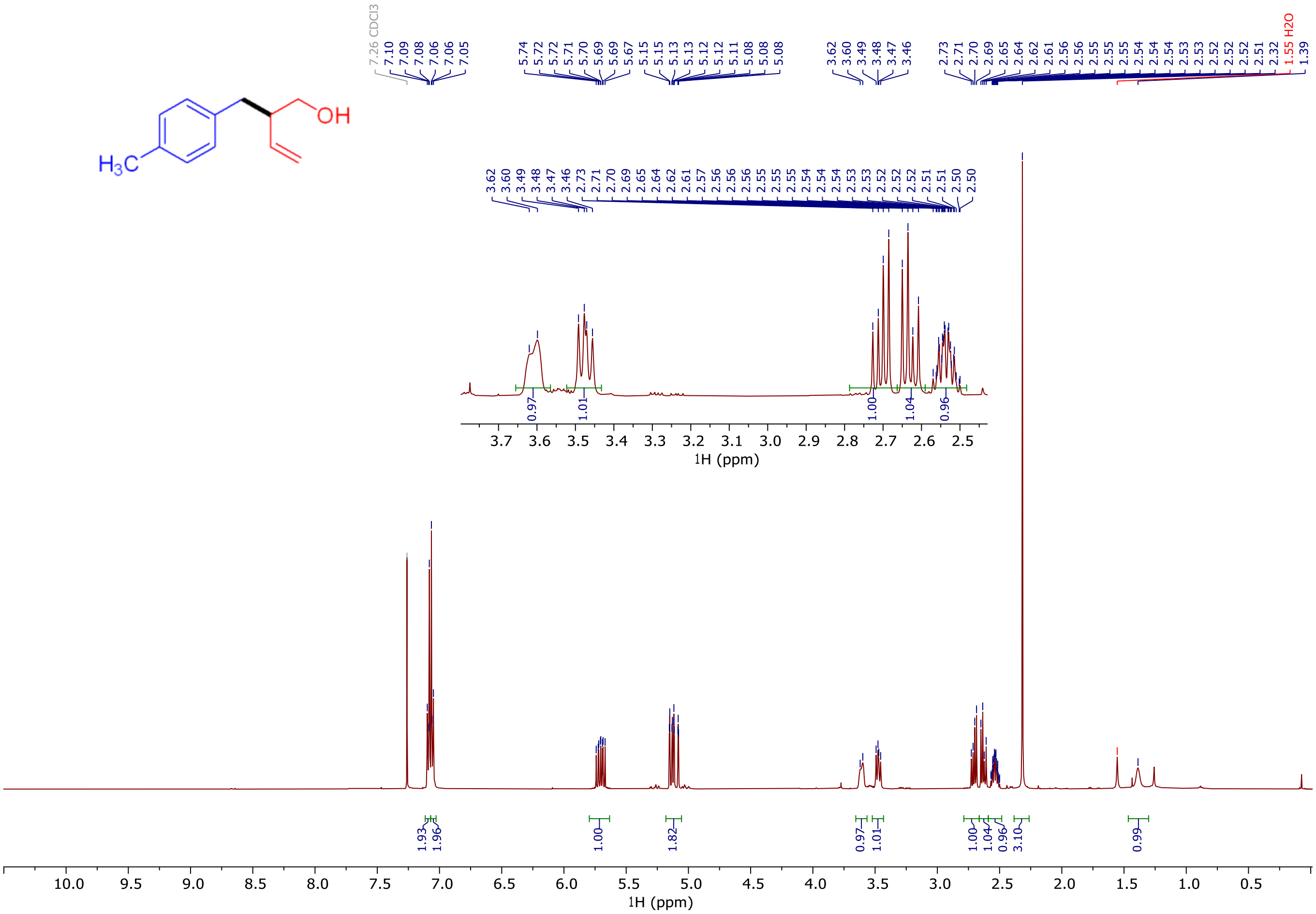

$^{13}C\{^{1}H\}$ NMR (126 MHz, $CDCl_3$) spectrum of **2-(4-methylbenzyl)but-3-en-1-ol (3ah)**

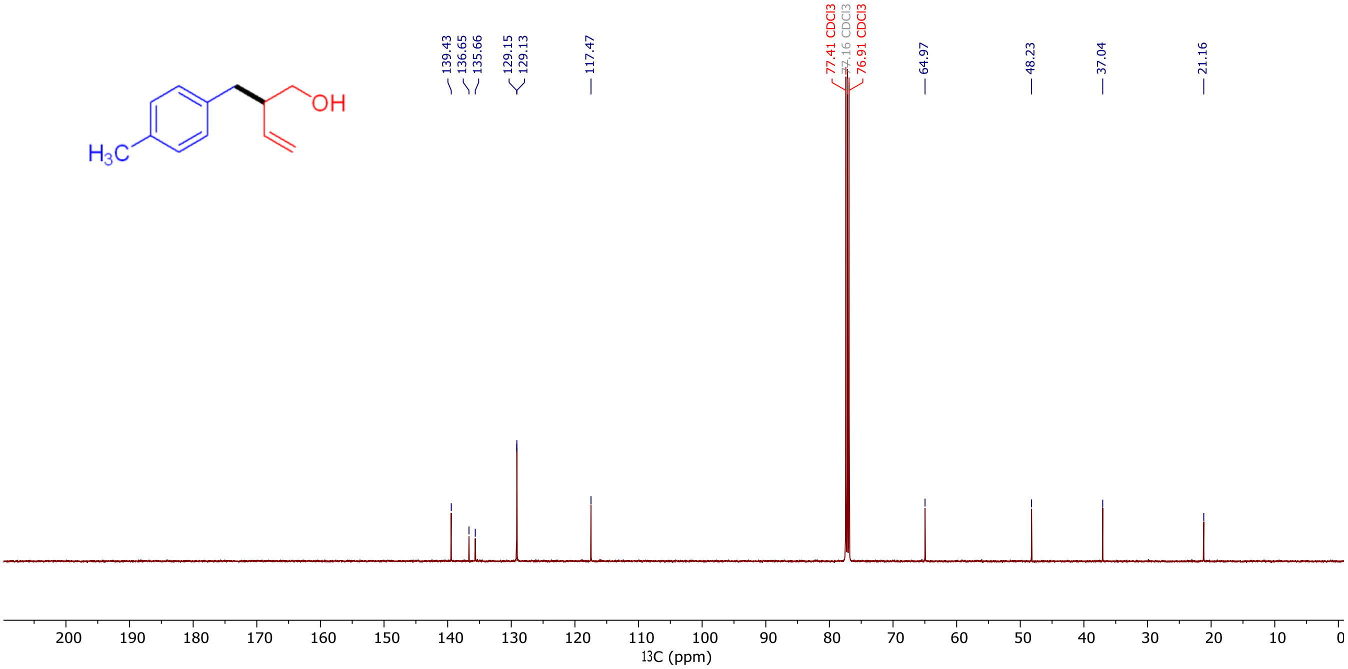

^{1}H NMR (500 MHz, $CDCl_3$) spectrum of **2-(4-isopropylbenzyl)but-3-en-1-ol (3ai)**

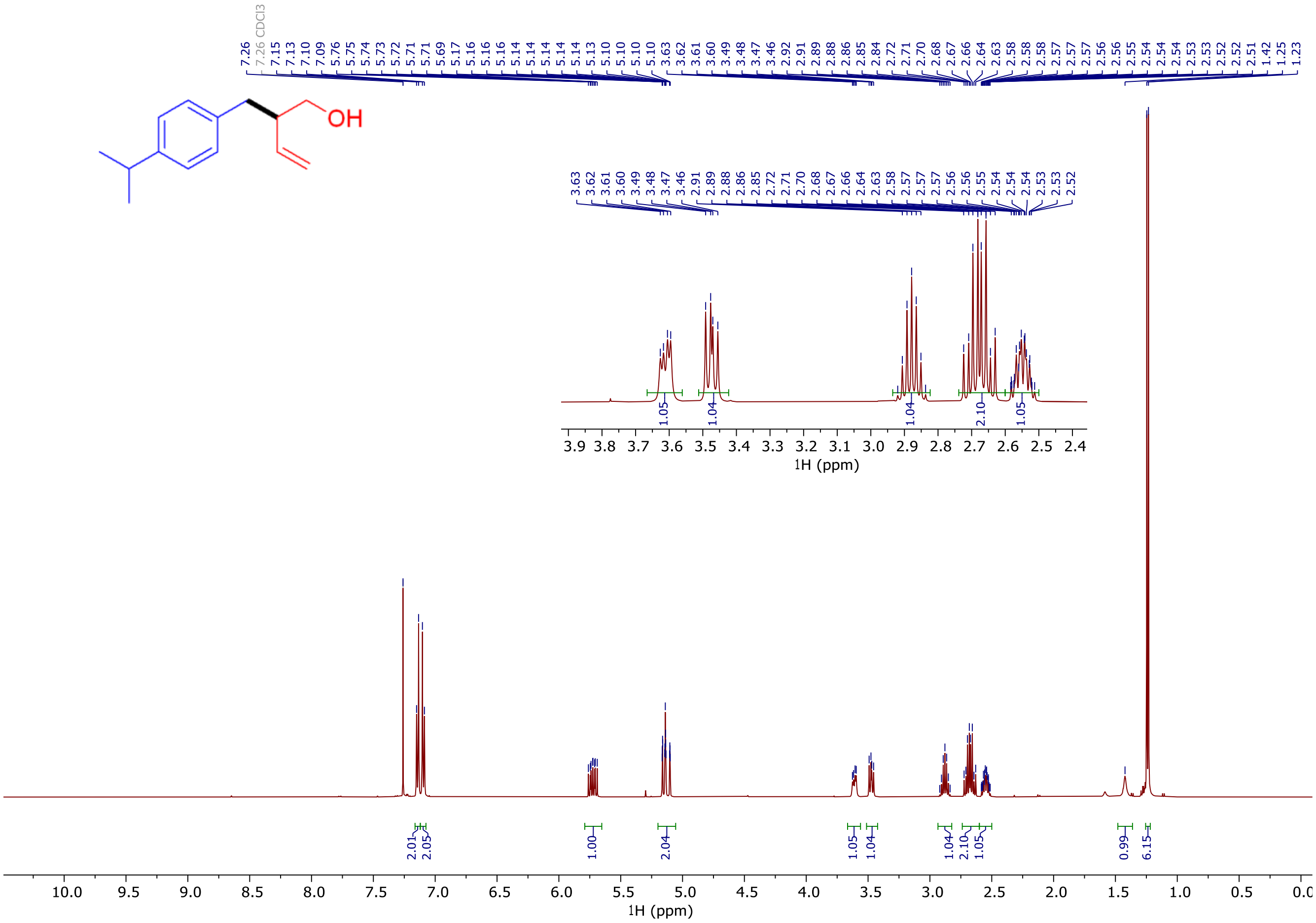

$^{13}C\{^{1}H\}$ NMR (126 MHz, $CDCl_3$) spectrum of **2-(4-isopropylbenzyl)but-3-en-1-ol (3ai)**

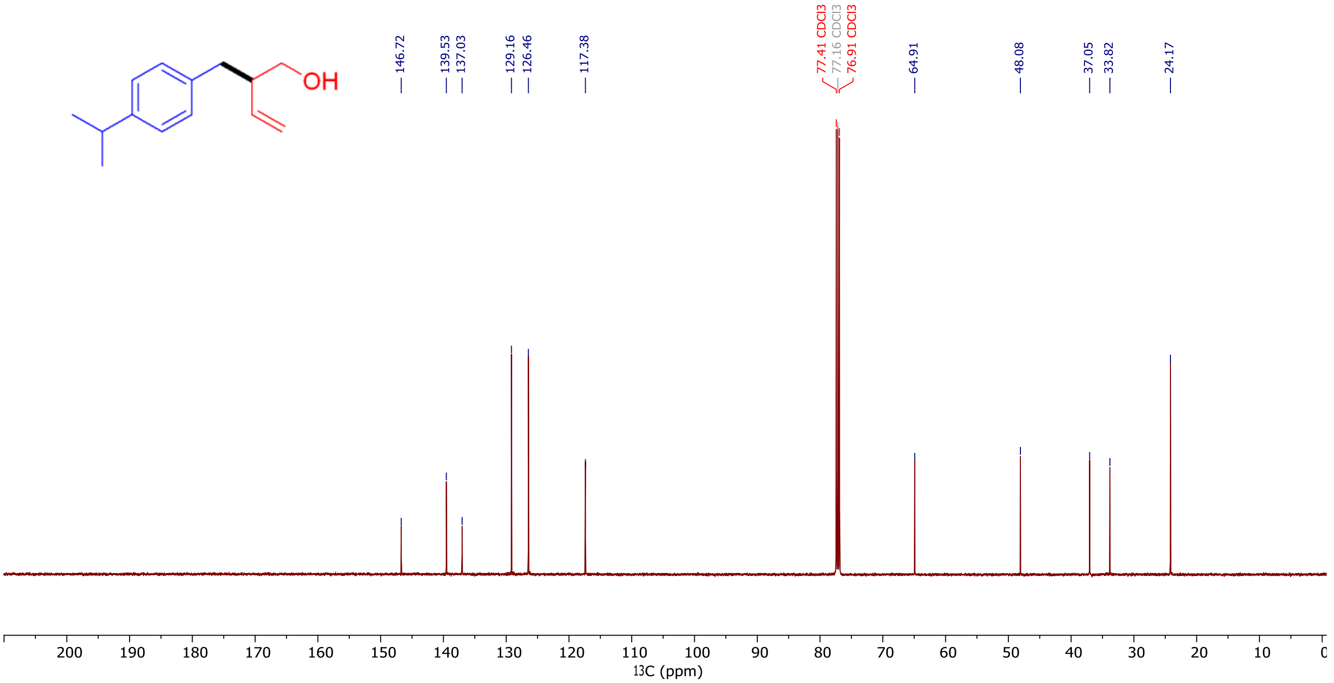

^{1}H NMR (500 MHz, $CDCl_3$) spectrum of **2-(4-(benzyloxy)benzyl)but-3-en-1-ol (3aj)**

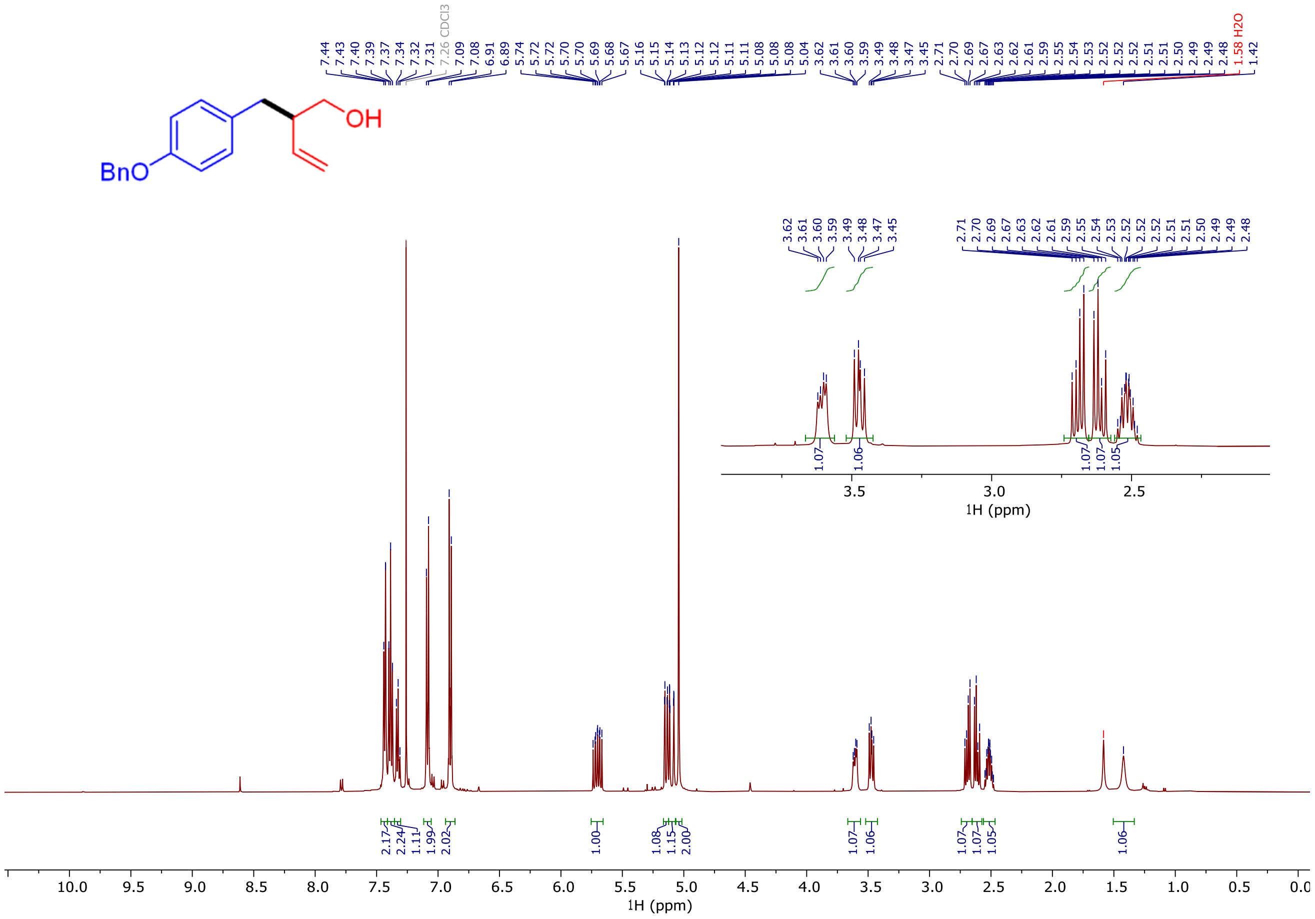

^{13}C{^{1}H} NMR (126 MHz, $CDCl_3$) spectrum of **2-(4-(benzyloxy)benzyl)but-3-en-1-ol (3aj)**

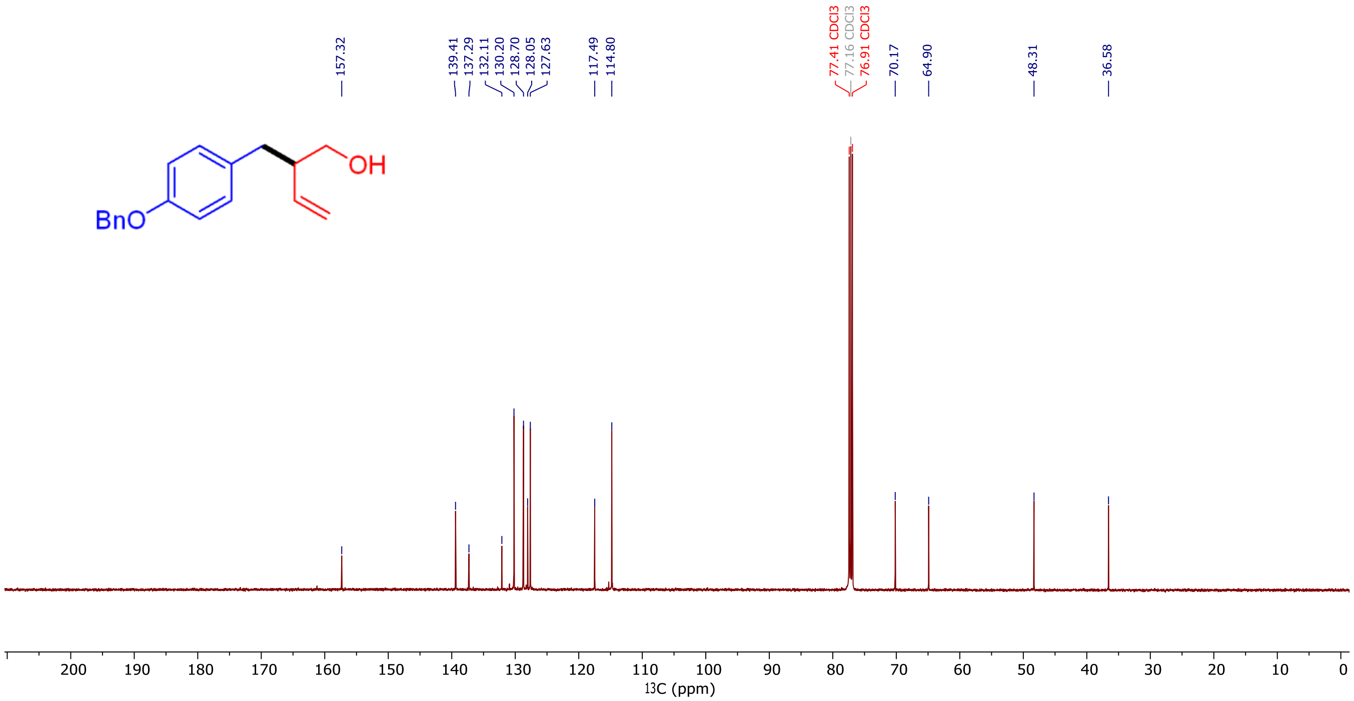

^{1}H NMR (500 MHz, $CDCl_3$) spectrum of **2-(2-fluorobenzyl)but-3-en-1-ol (3am)**

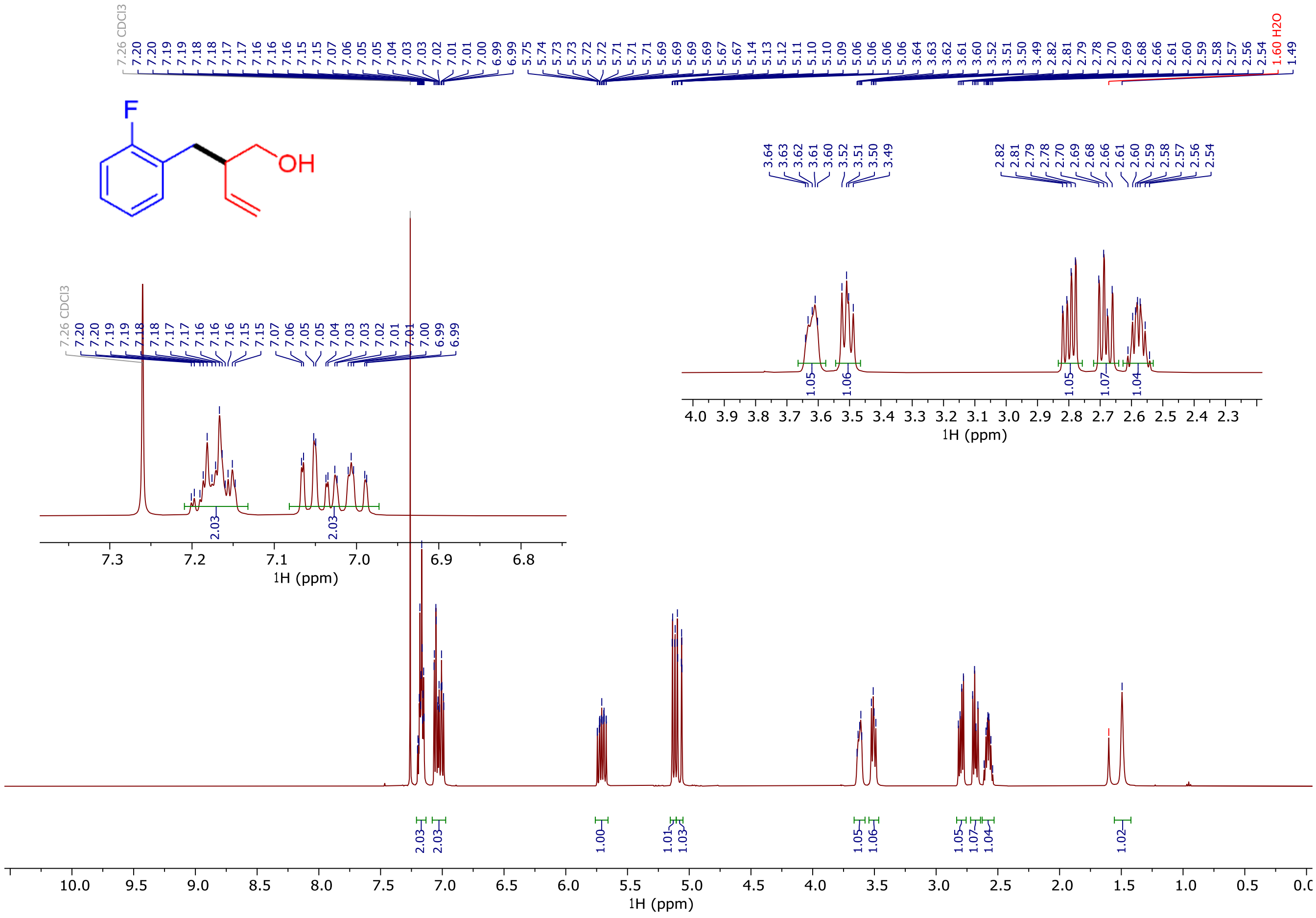

$^{13}C\{^{1}H\}$ NMR (126 MHz, $CDCl_3$) spectrum of **2-(2-fluorobenzyl)but-3-en-1-ol (3am)**

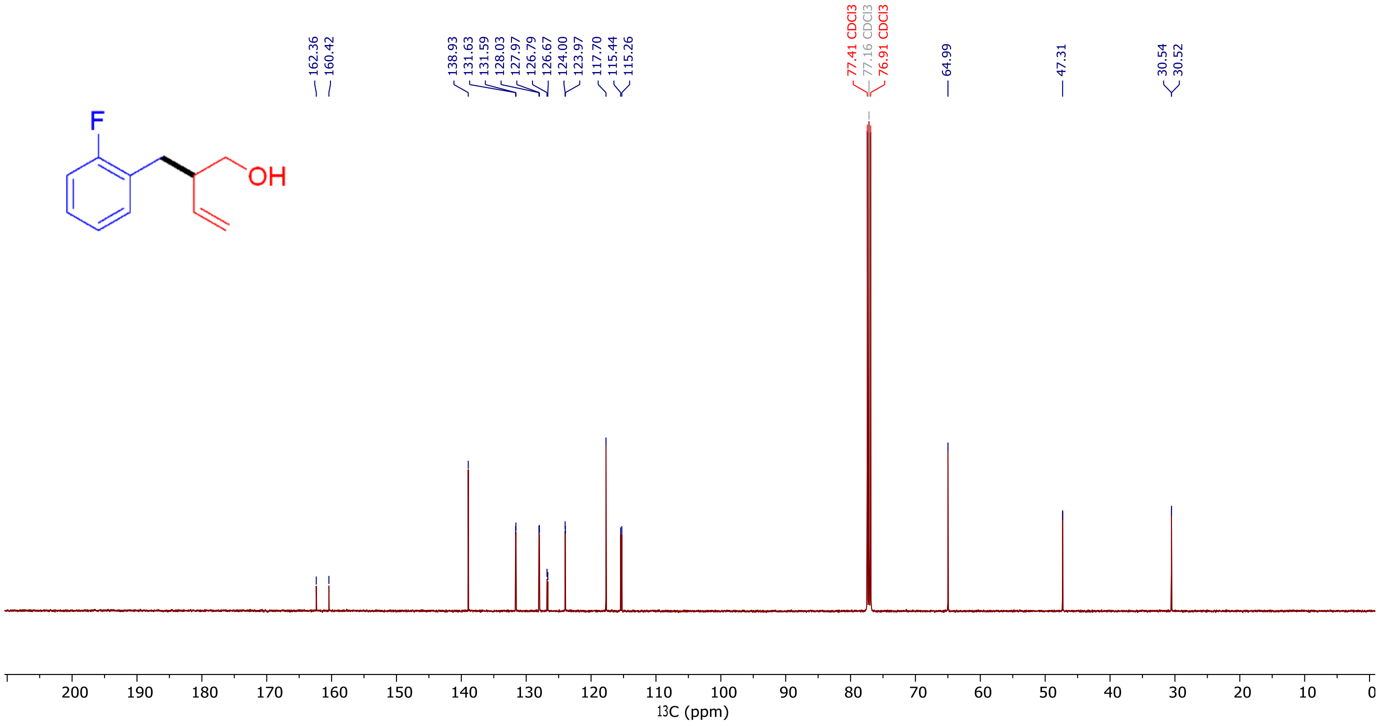

^{19}F NMR (471 MHz, $CDCl_3$) spectrum of **2-(2-fluorobenzyl)but-3-en-1-ol (3am)**

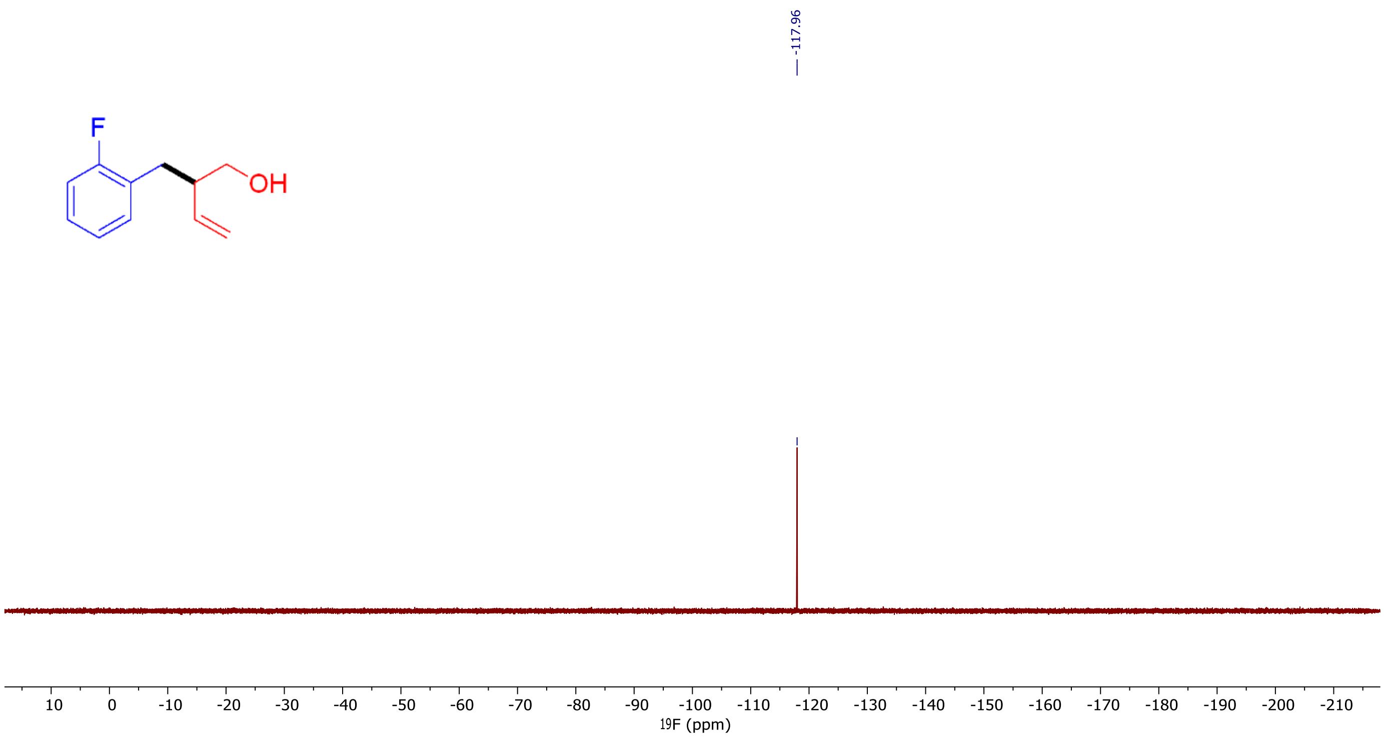

^{1}H NMR (500 MHz, $CDCl_3$) spectrum of **2-(2-chlorobenzyl)but-3-en-1-ol (3an)**

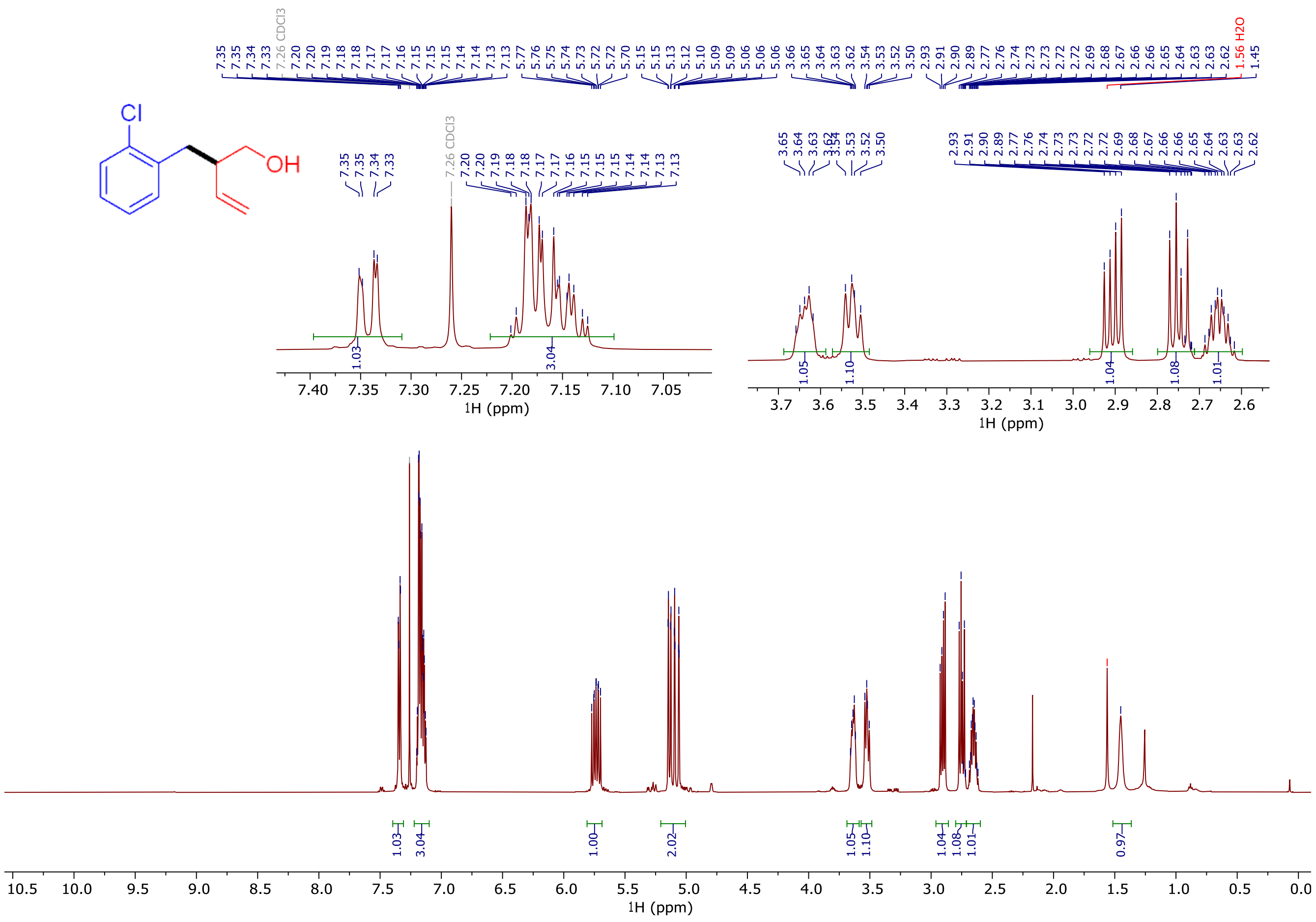

$^{13}C\{^{1}H\}$ NMR (126 MHz, $CDCl_3$) spectrum of **2-(2-chlorobenzyl)but-3-en-1-ol (3an)**

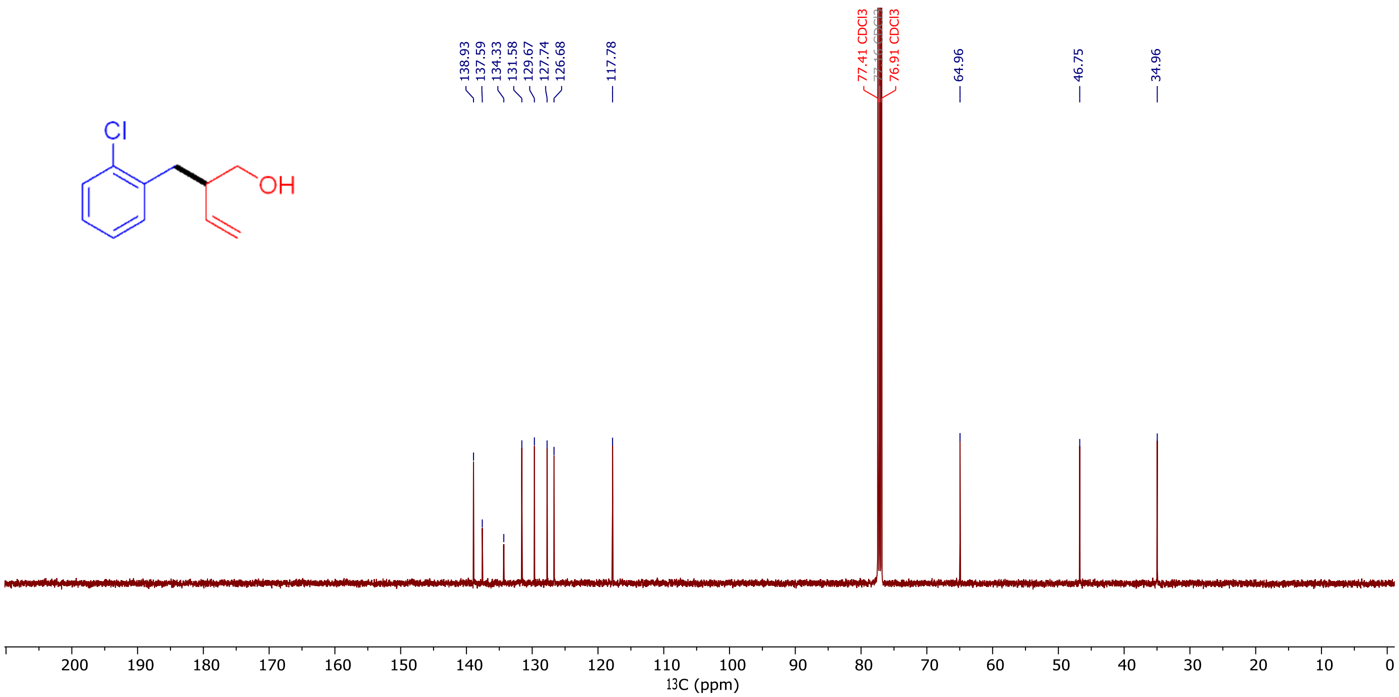

^{1}H NMR (500 MHz, $CDCl_3$) spectrum of **2-(2-methylbenzyl)but-3-en-1-ol (3ao)**

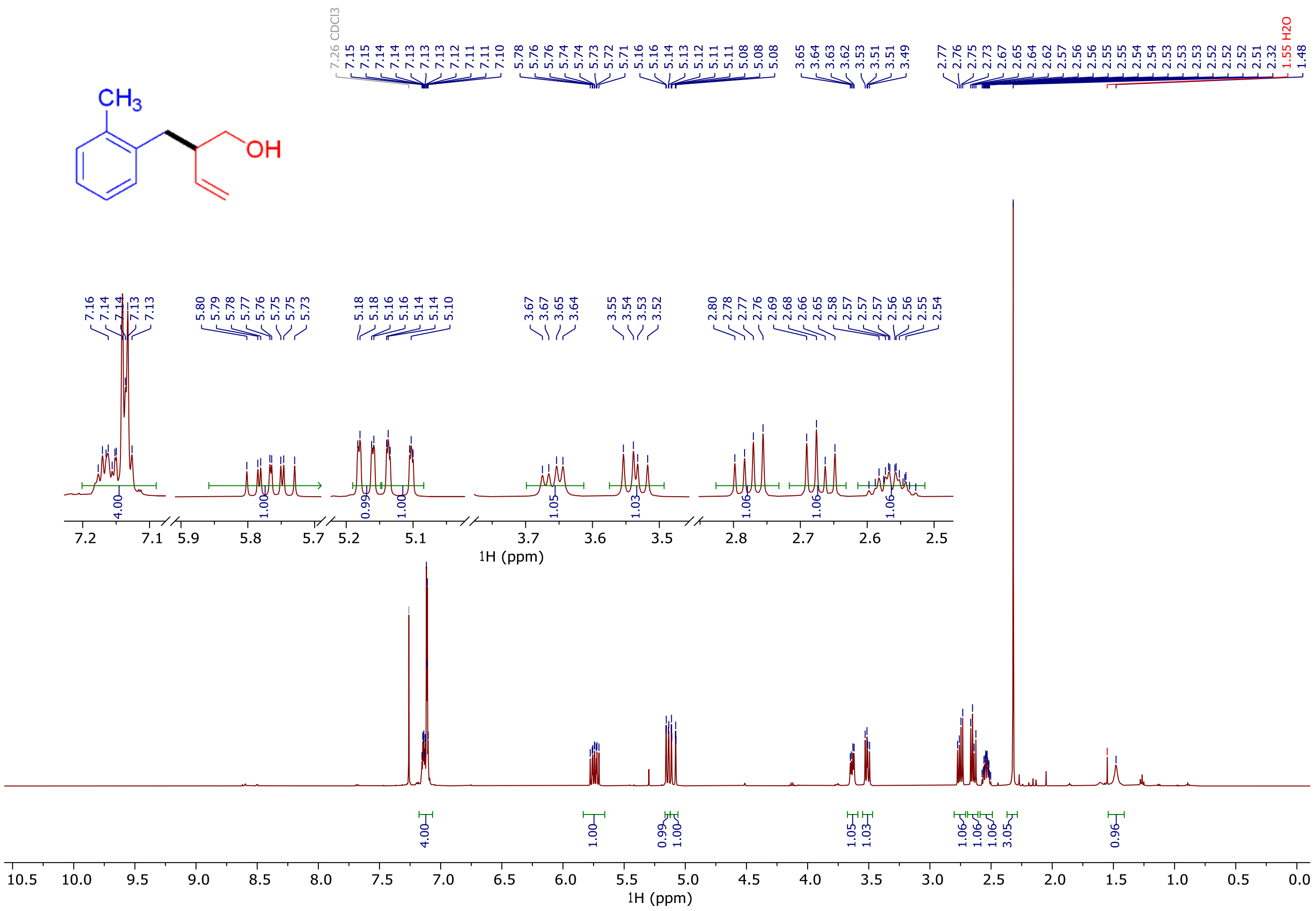

$^{13}C\{^{1}H\}$ NMR (126 MHz, $CDCl_3$) spectrum of **2-(2-methylbenzyl)but-3-en-1-ol (3ao)**

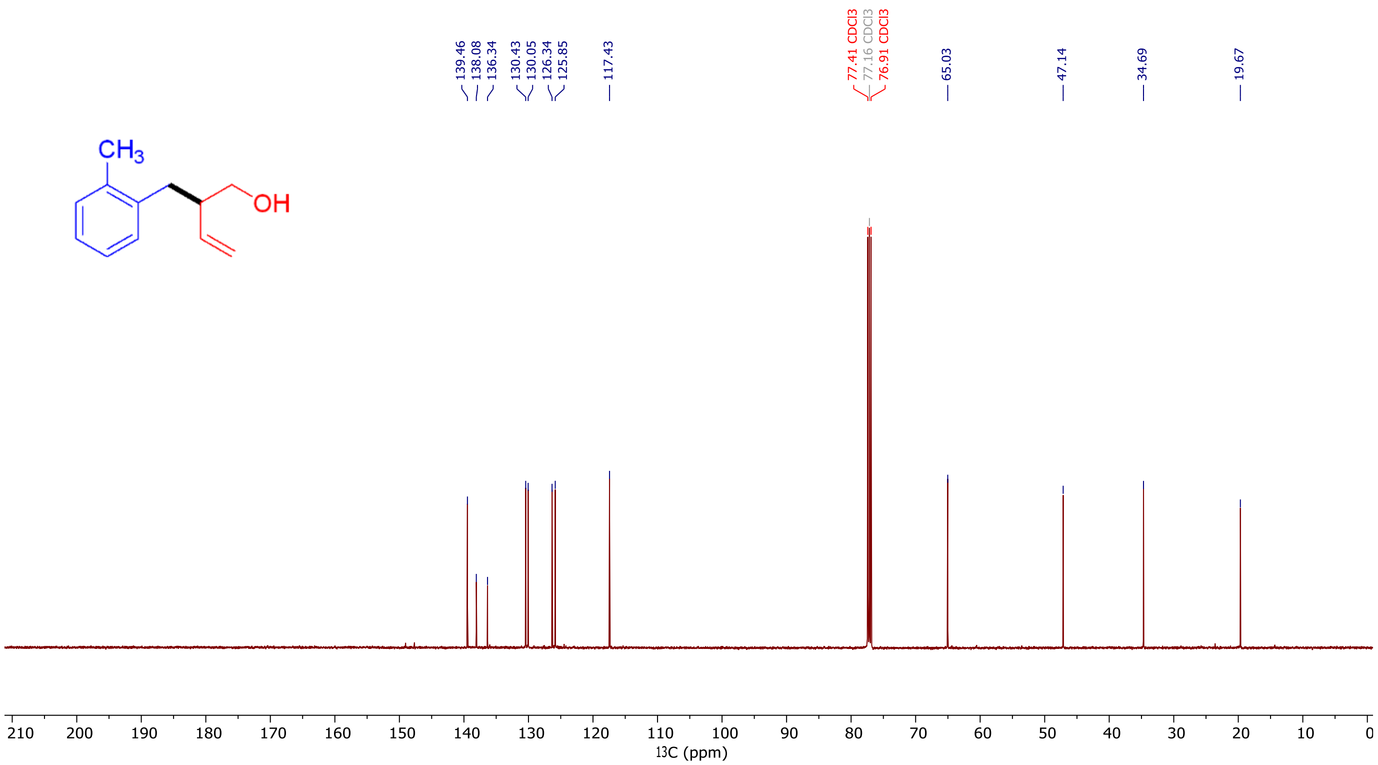

^{1}H NMR (800 MHz, $CDCl_3$) spectrum of **2-(2-methoxybenzyl)but-3-en-1-yl acetate (3ap)**

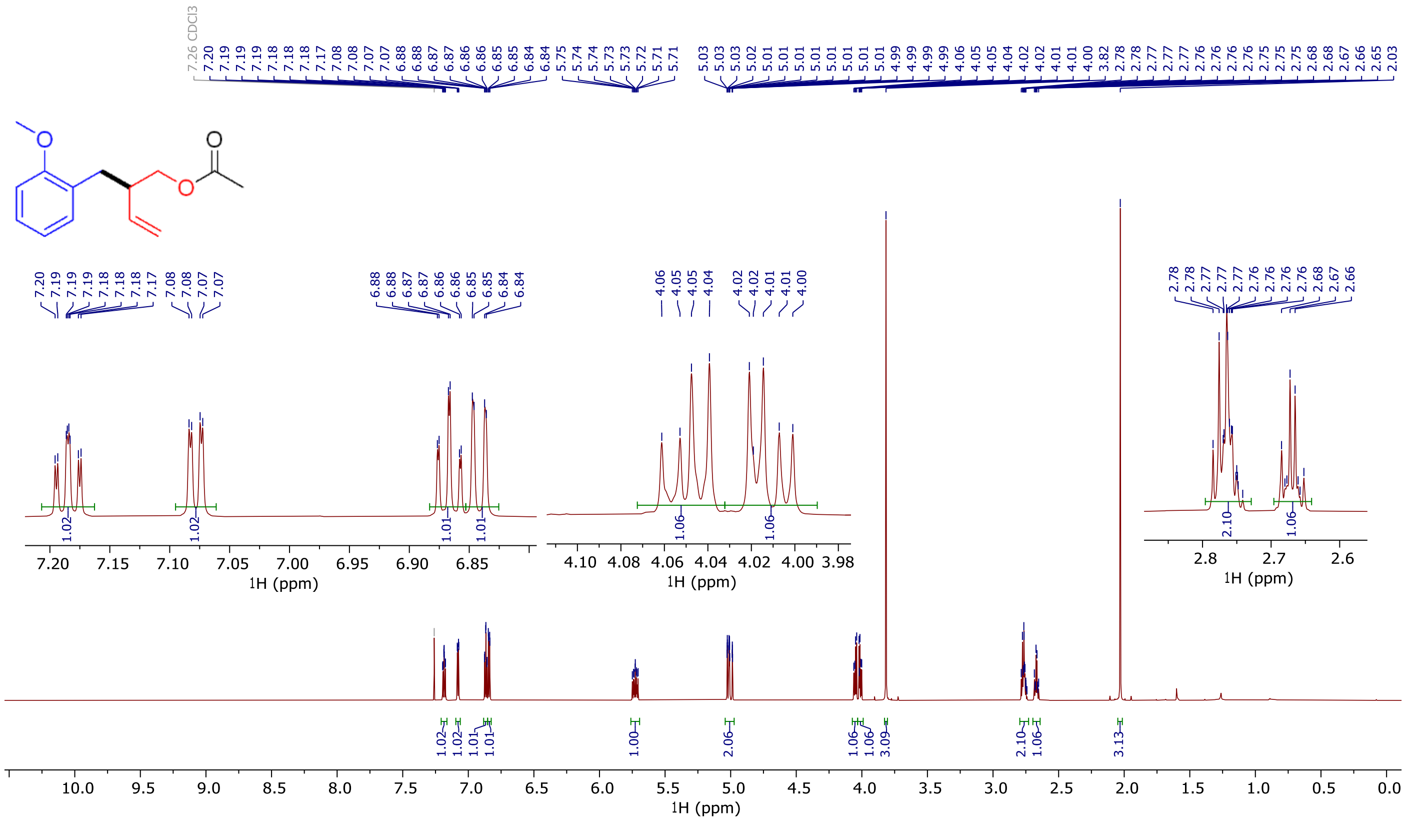

$^{13}C\{^{1}H\}$ NMR (201 MHz, $CDCl_3$) spectrum of **2-(2-methoxybenzyl)but-3-en-1-yl acetate (3ap)**

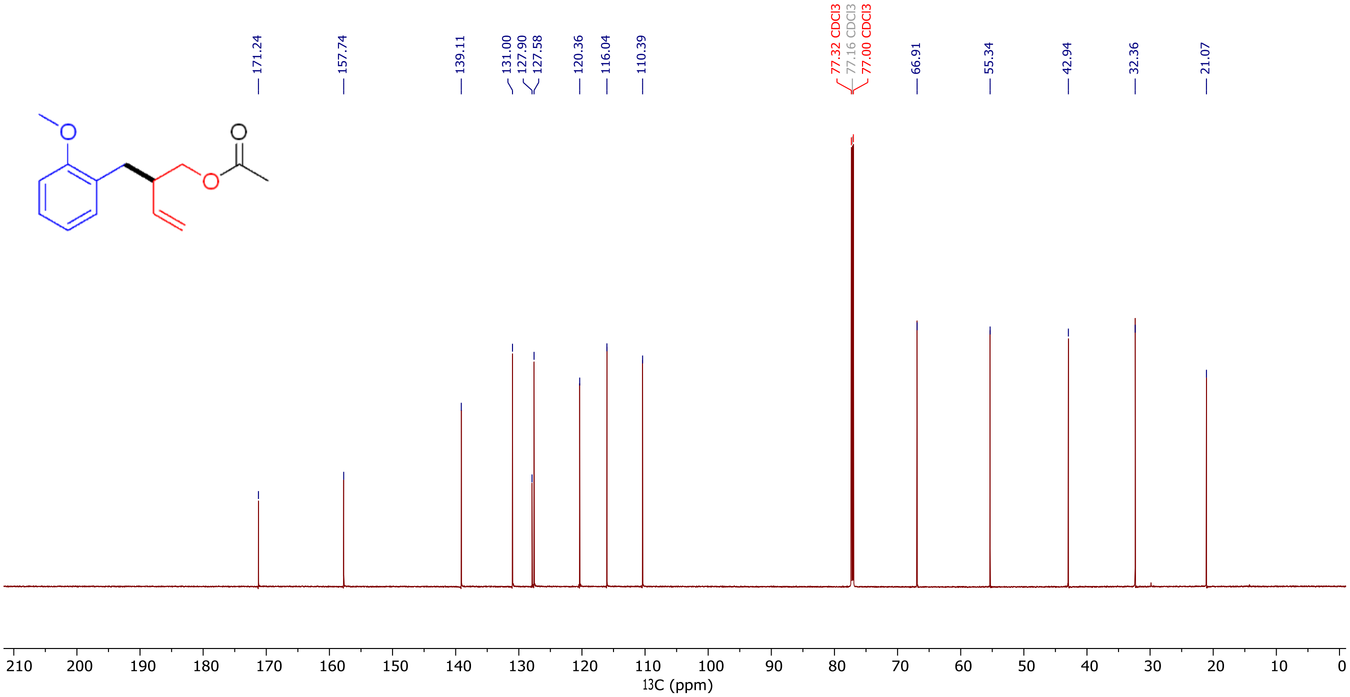

^{1}H NMR (500 MHz, $CDCl_3$) spectrum of **2-(2-chlorobenzyl)but-3-en-1-ol (3aq)**

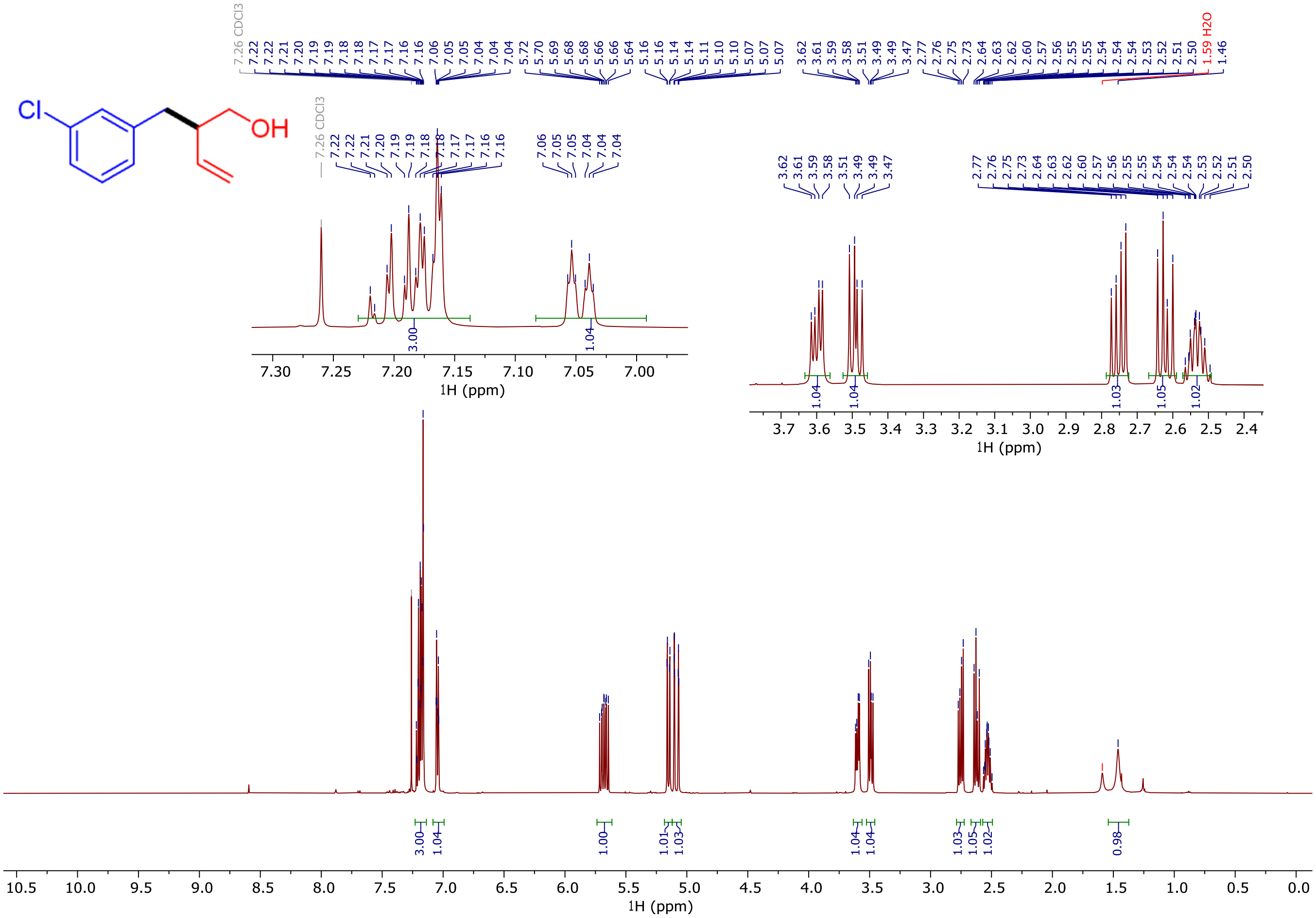

$^{13}C\{^1H\}$ NMR (126 MHz, $CDCl_3$) spectrum of **2-(2-chlorobenzyl)but-3-en-1-ol (3aq)**

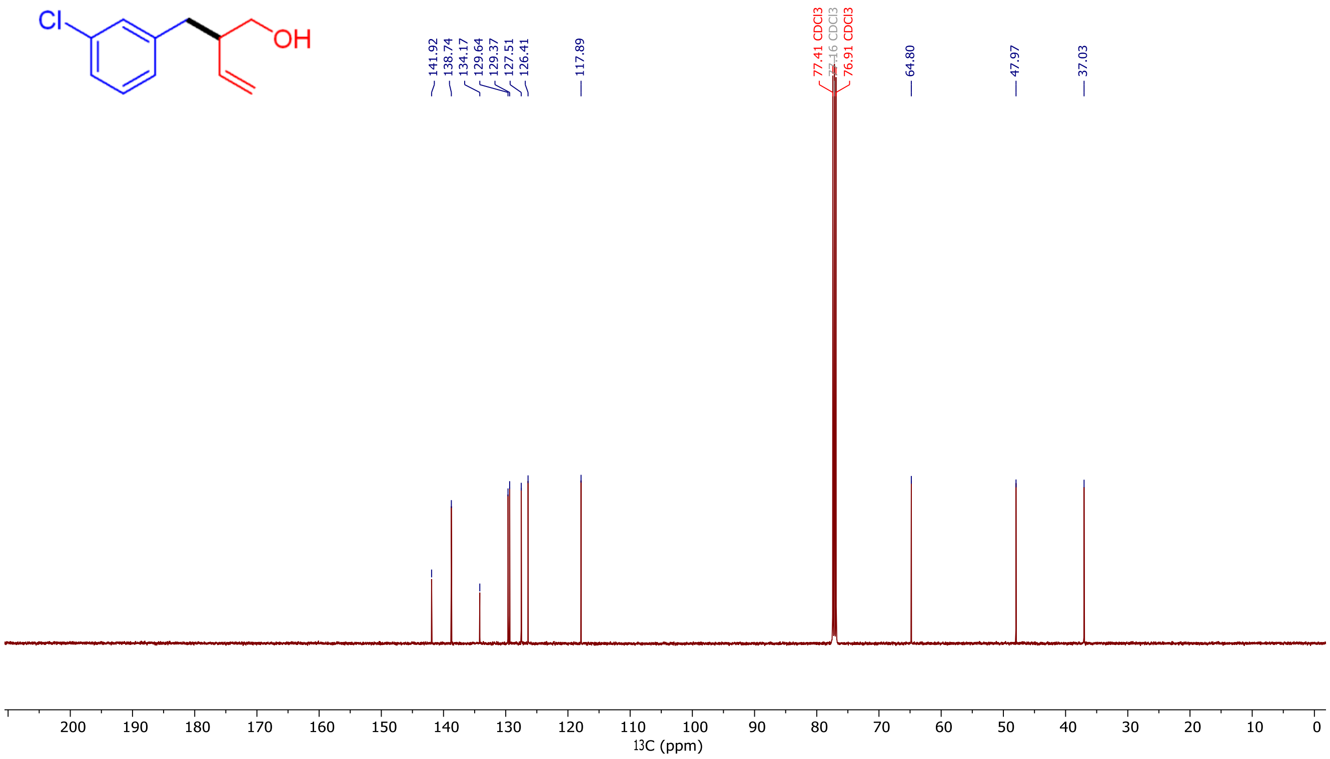

^1H NMR (500 MHz, $CDCl_3$) spectrum of **2-([1,1'-biphenyl]-3-ylmethyl)but-3-en-1-ol (3ar)**

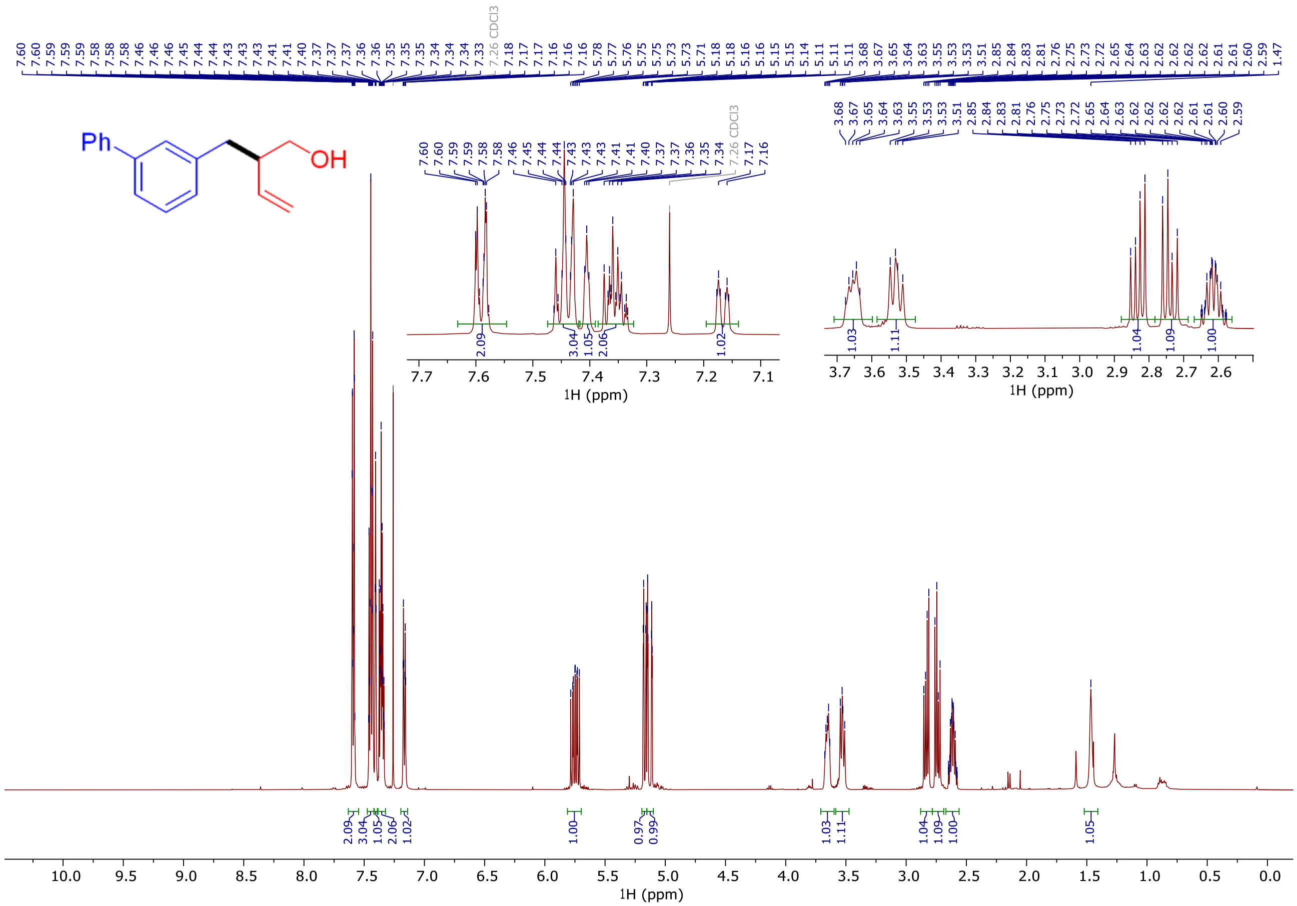

$^{13}C\{^{1}H\}$ NMR (126 MHz, $CDCl_3$) spectrum of **2-([1,1'-biphenyl]-3-ylmethyl)but-3-en-1-ol (3ar)**

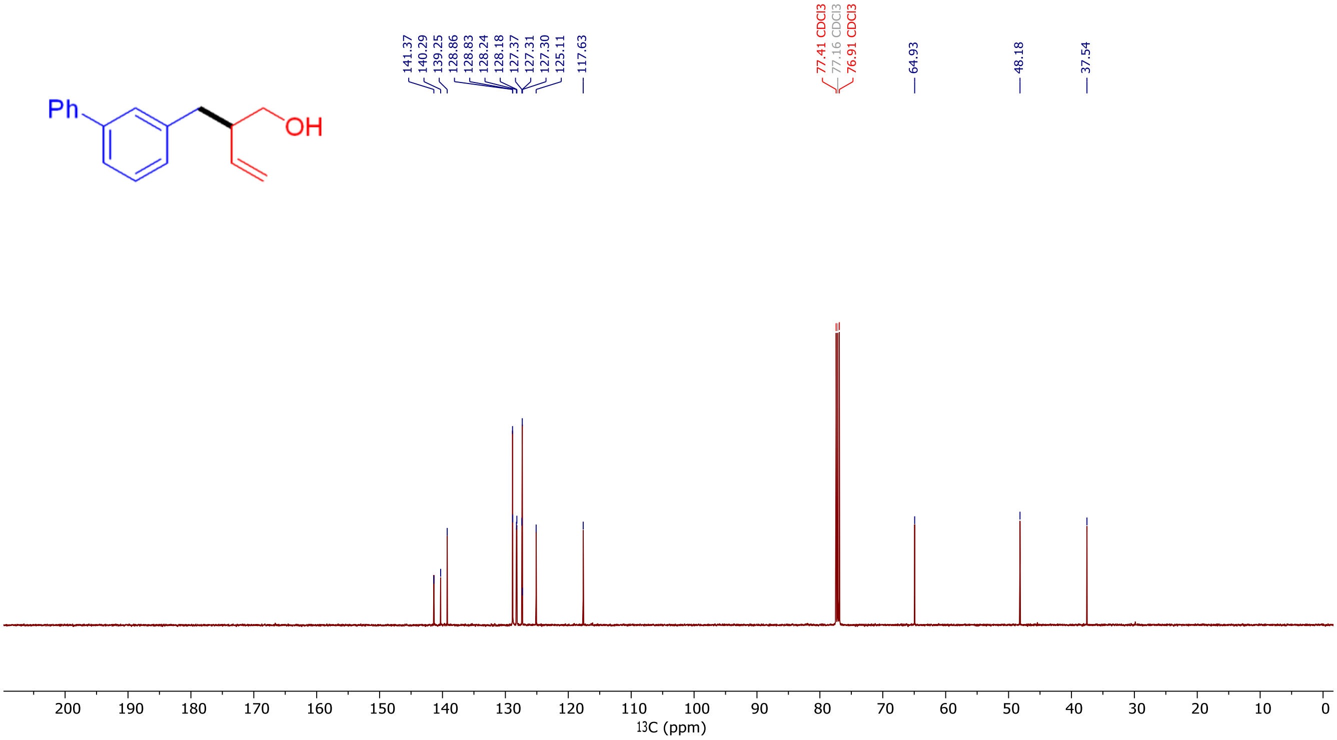

^{1}H NMR (500 MHz, $CDCl_3$) spectrum of **2-(3-methylbenzyl)but-3-en-1-ol (3as)**

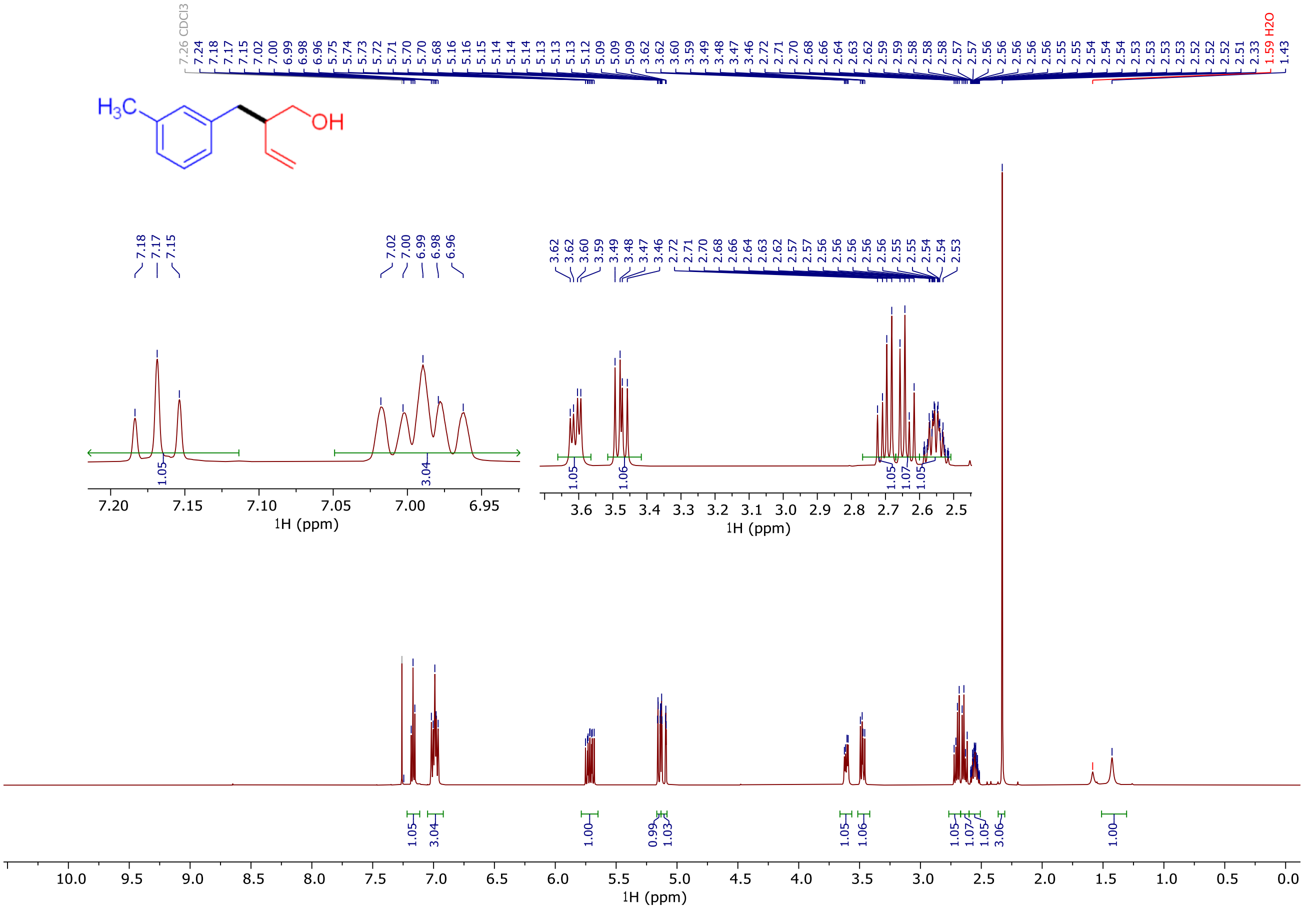

$^{13}C\{^1H\}$ NMR (126 MHz, $CDCl_3$) spectrum of **2-(3-methylbenzyl)but-3-en-1-ol (3as)**

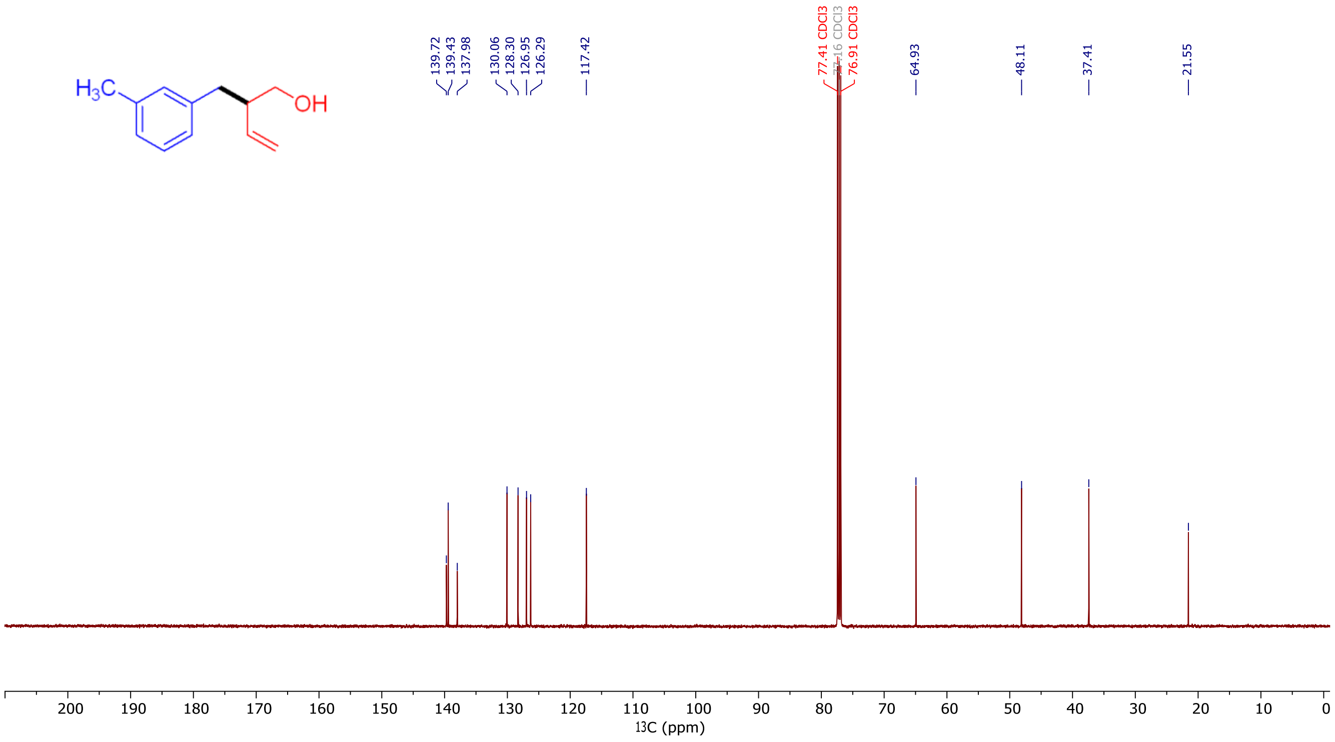

^1H NMR (500 MHz, $CDCl_3$) spectrum of **2-(naphthalen-2-ylmethyl)but-3-en-1-ol (3at)**

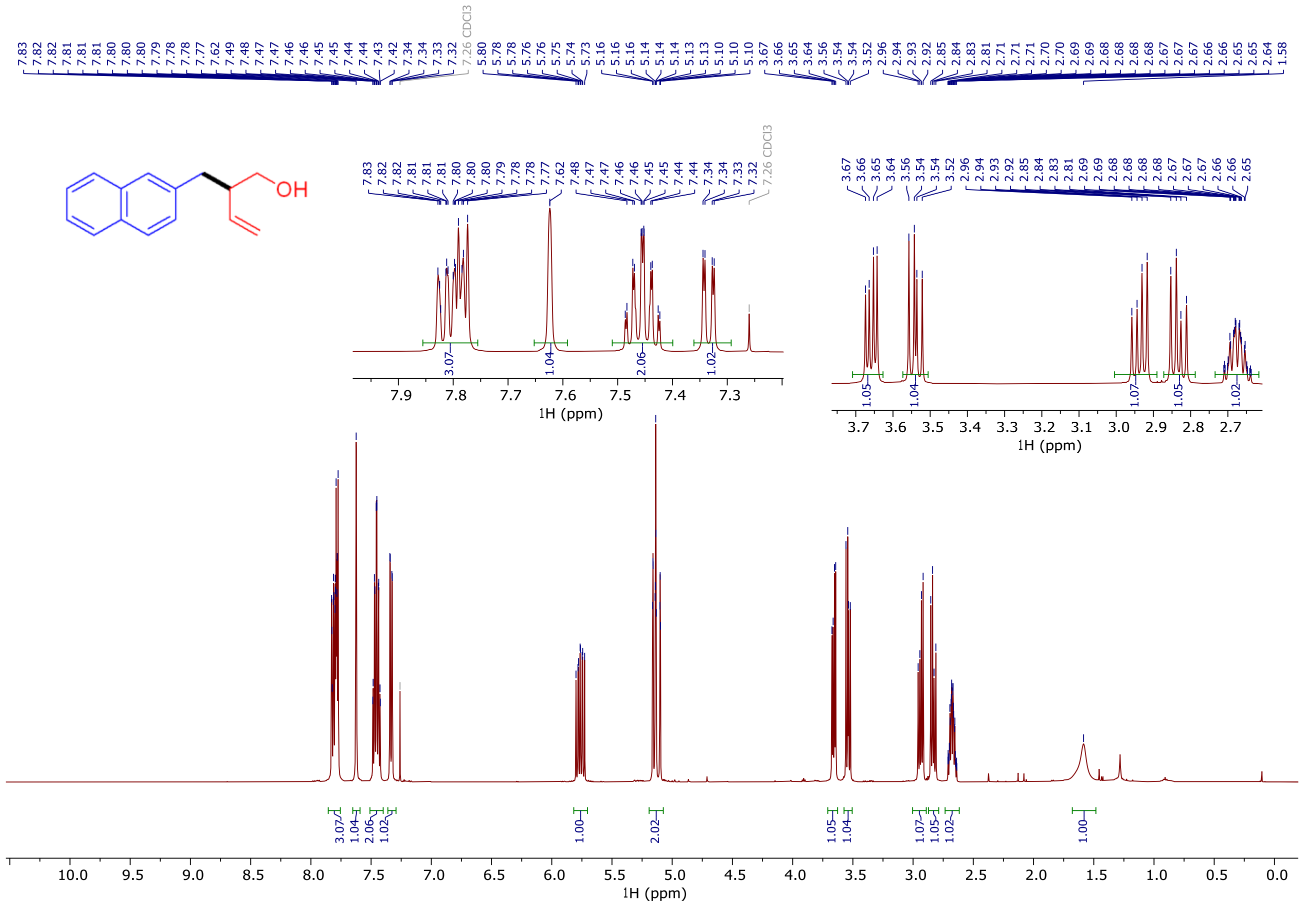

$^{13}C\{^{1}H\}$ NMR (126 MHz, $CDCl_3$) spectrum of **2-(naphthalen-2-ylmethyl)but-3-en-1-ol (3at)**

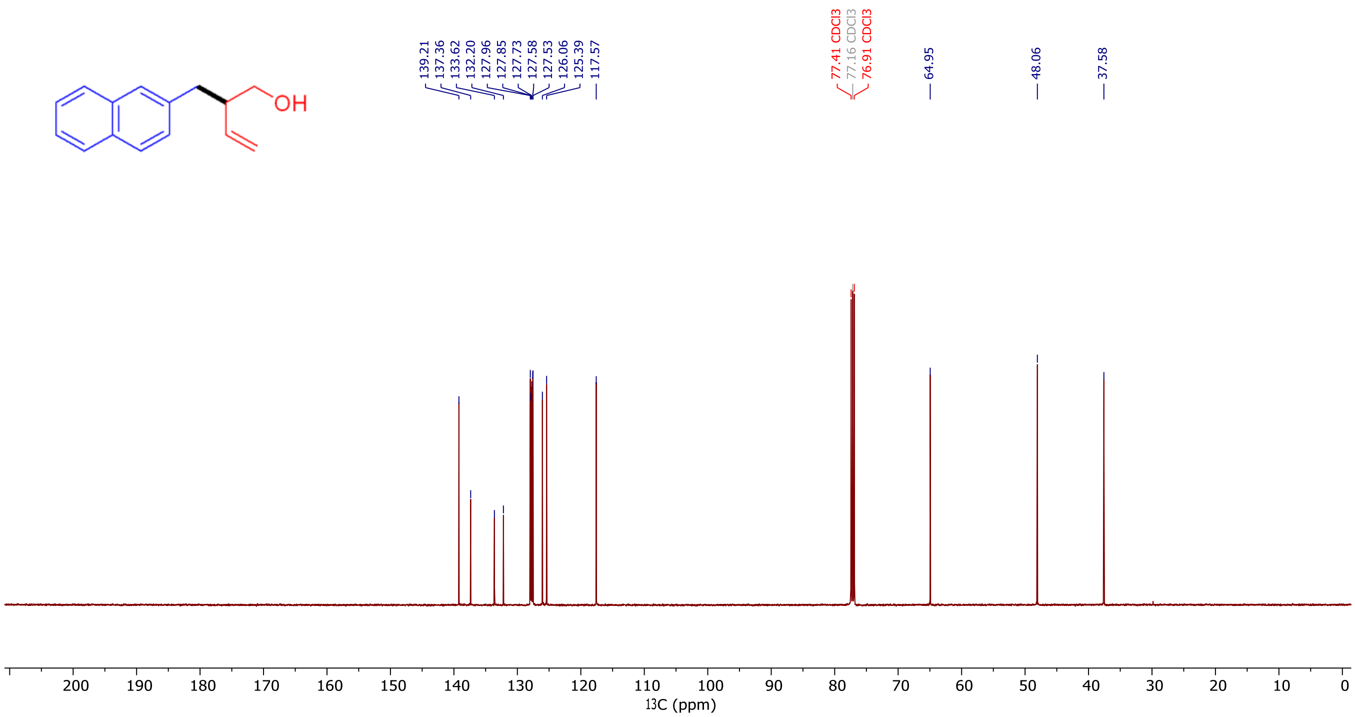

^{1}H NMR (500 MHz, $CDCl_3$) spectrum of **2-(naphthalen-1-ylmethyl)but-3-en-1-ol (3au)**

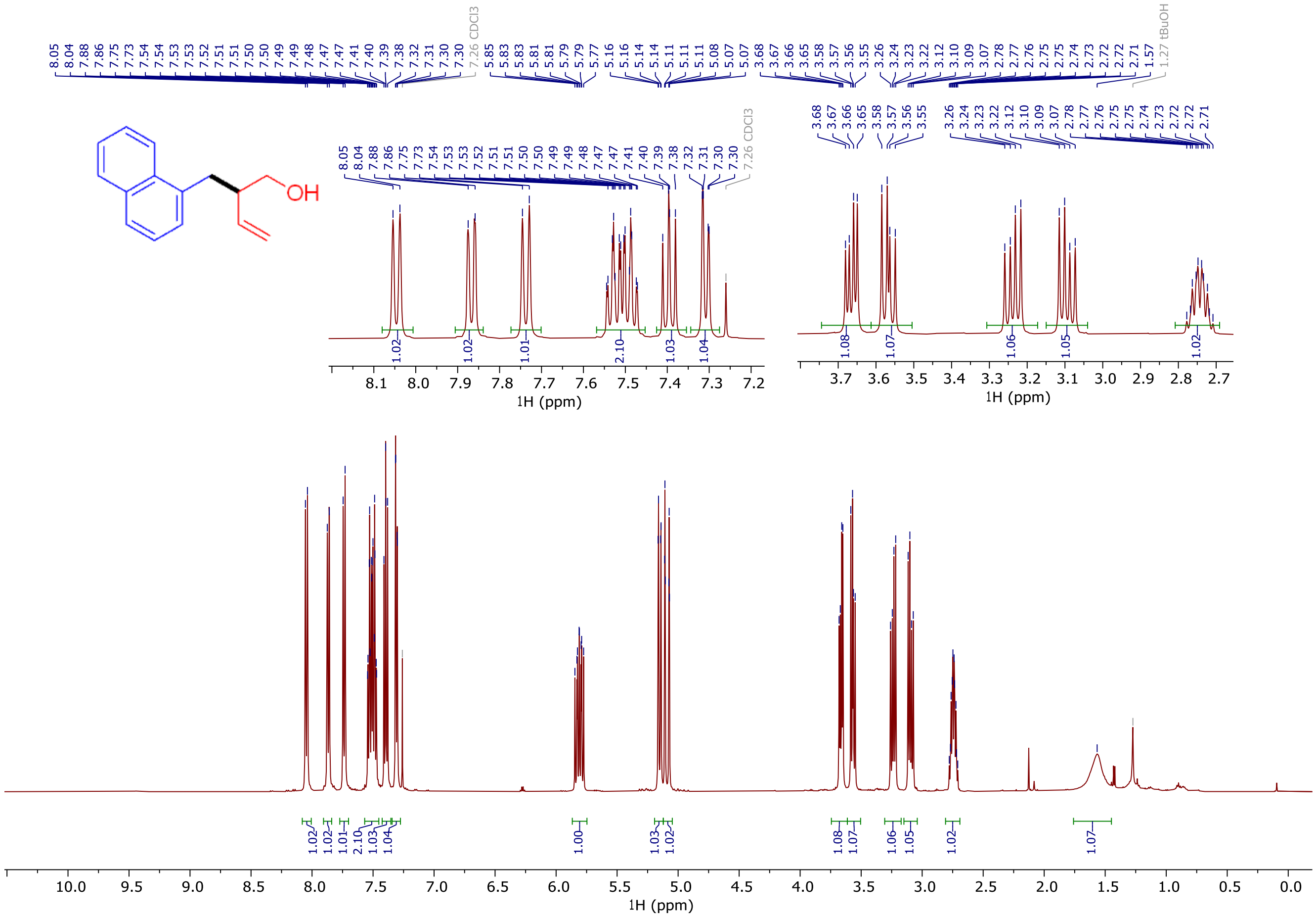

$^{13}C\{^{1}H\}$ NMR (126 MHz, $CDCl_3$) spectrum of **2-(naphthalen-1-ylmethyl)but-3-en-1-ol (3au)**

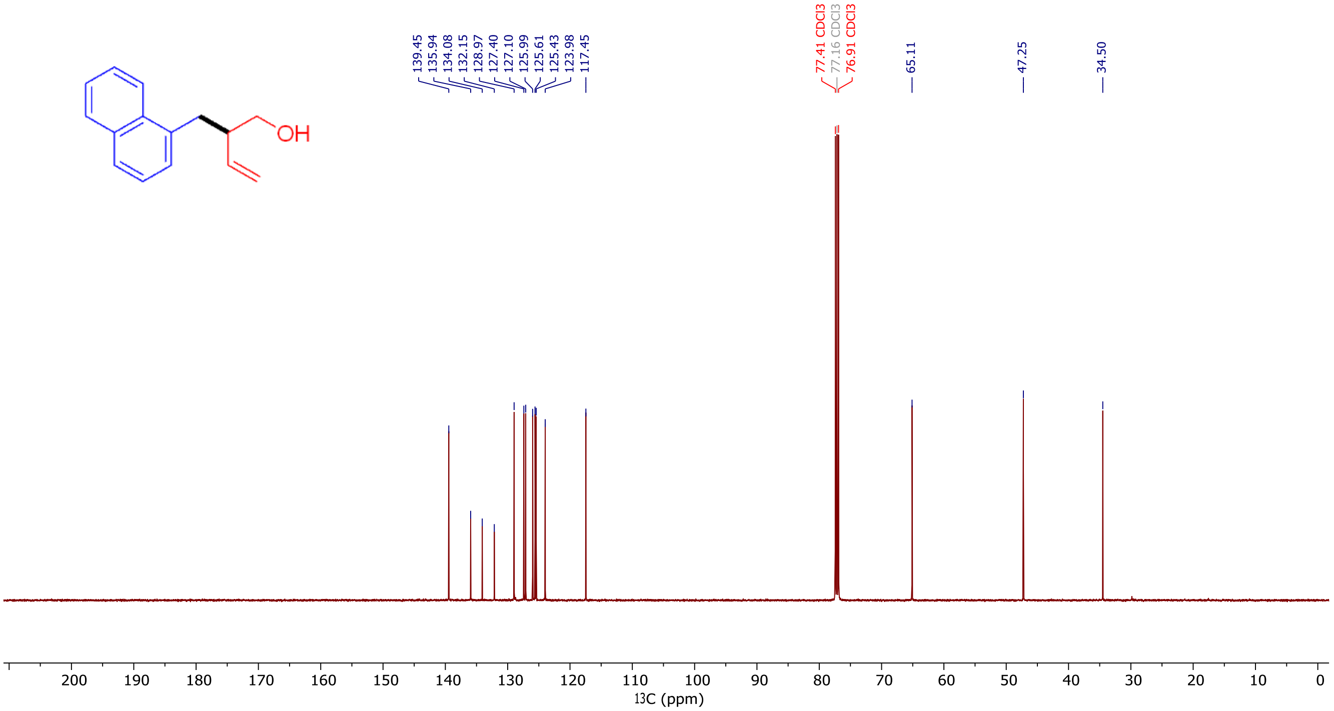

^{1}H NMR (500 MHz, $CDCl_3$) spectrum of **2-(2,6-dichlorobenzyl)but-3-en-1-ol (3av)**

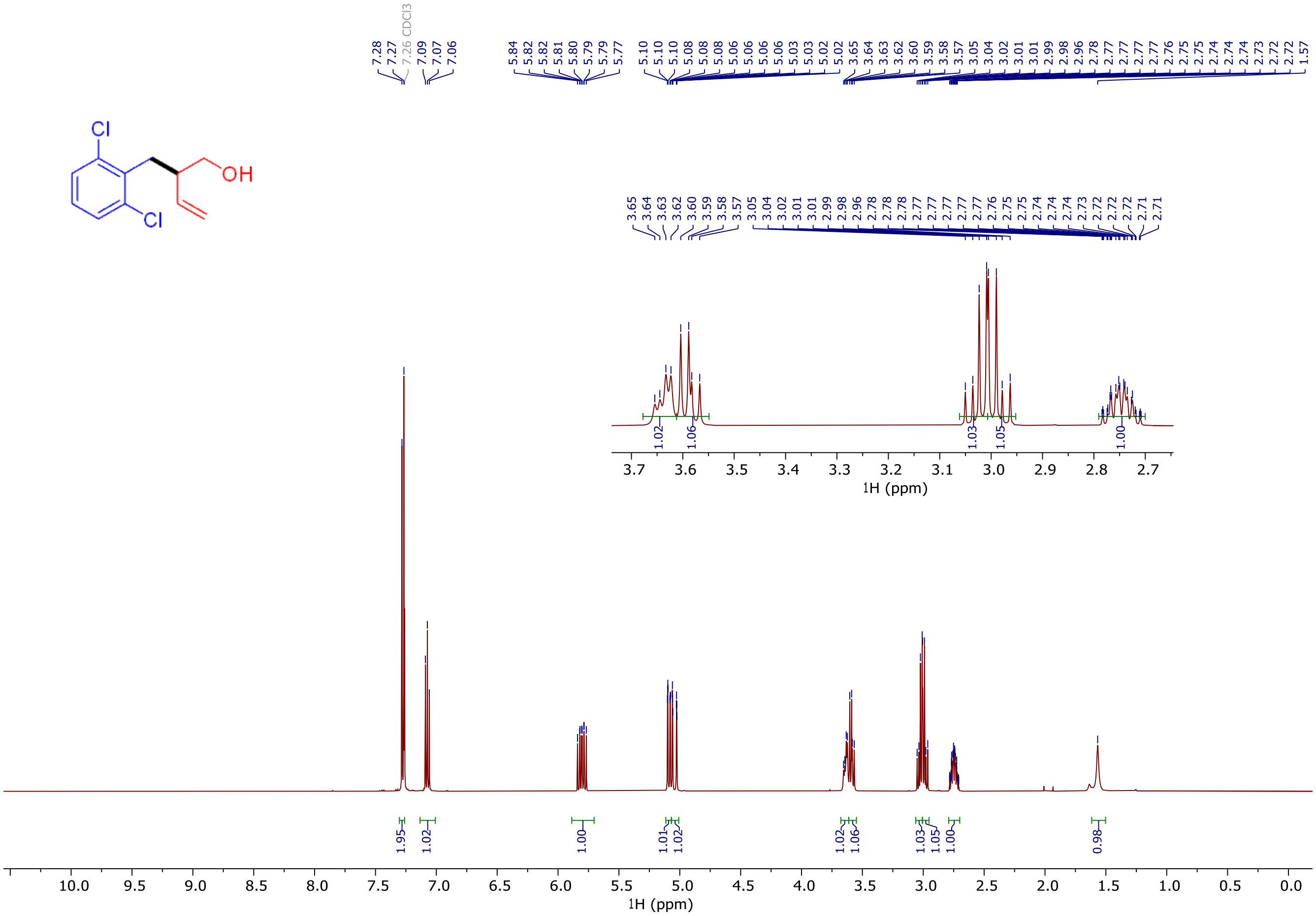

$^{13}C\{^1H\}$ NMR (126 MHz, $CDCl_3$) spectrum of **2-(2,6-dichlorobenzyl)but-3-en-1-ol (3av)**

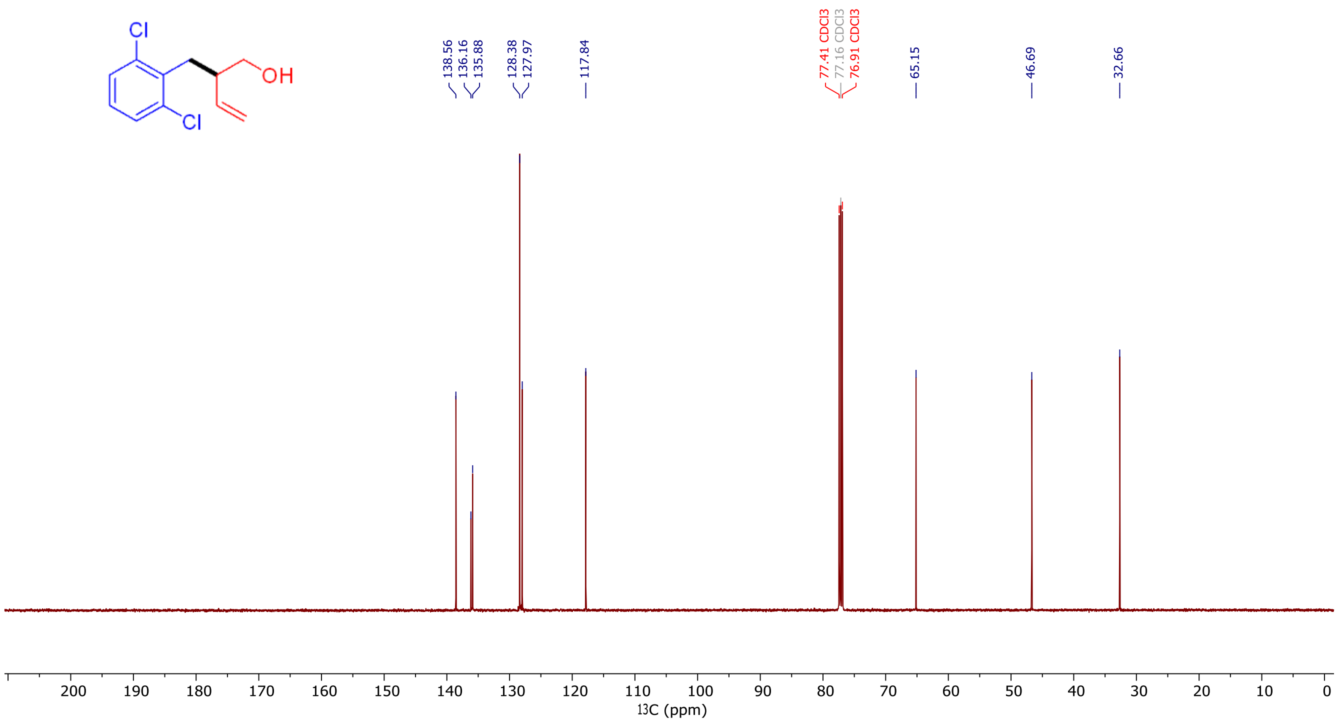

^1H NMR (500 MHz, $CDCl_3$) spectrum of **2-(2,4,6-trimethylbenzyl)but-3-en-1-ol (3aw)**

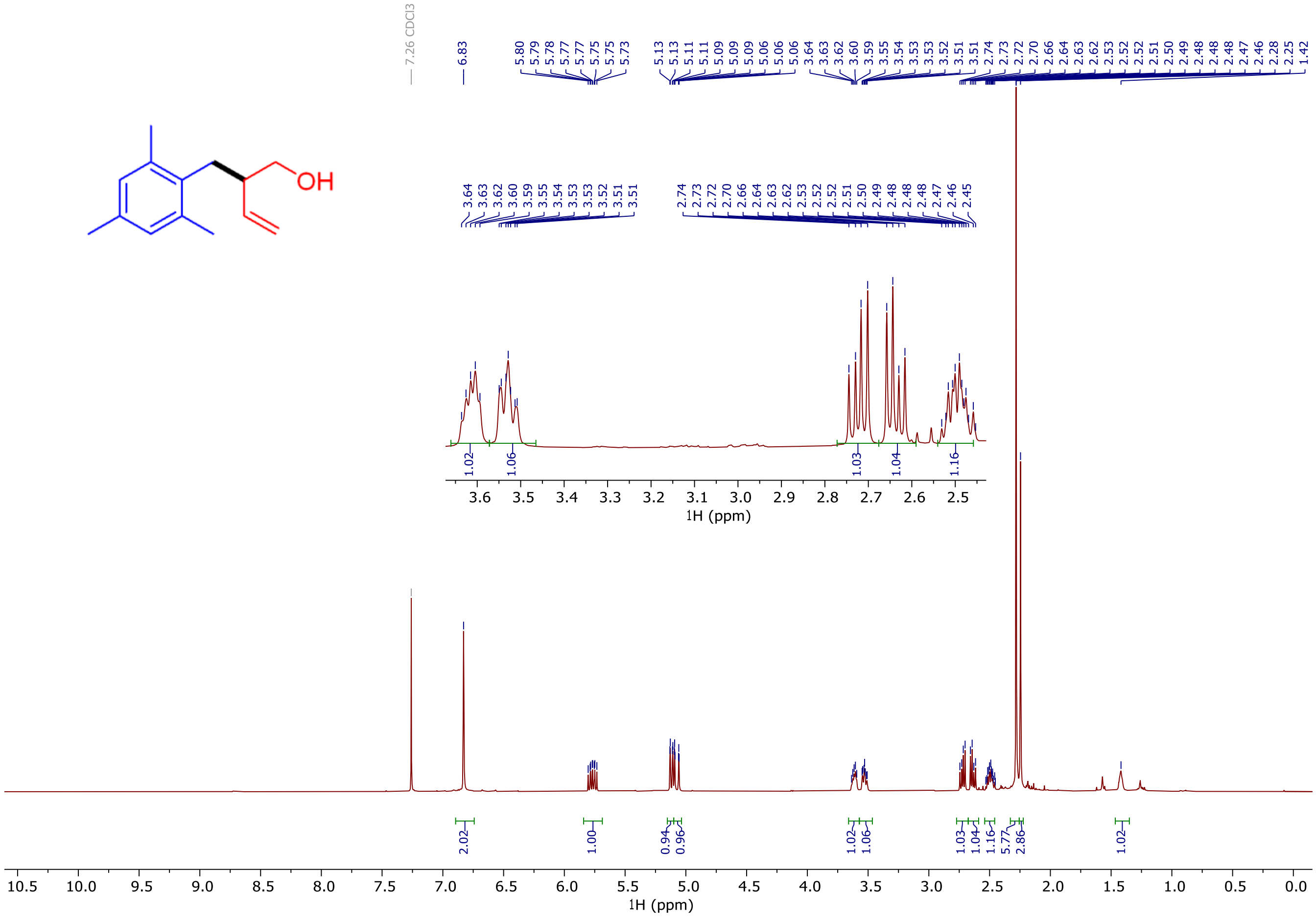

$^{13}C\{^1H\}$ NMR (126 MHz, $CDCl_3$) spectrum of **2-(2,4,6-trimethylbenzyl)but-3-en-1-ol (3aw)**

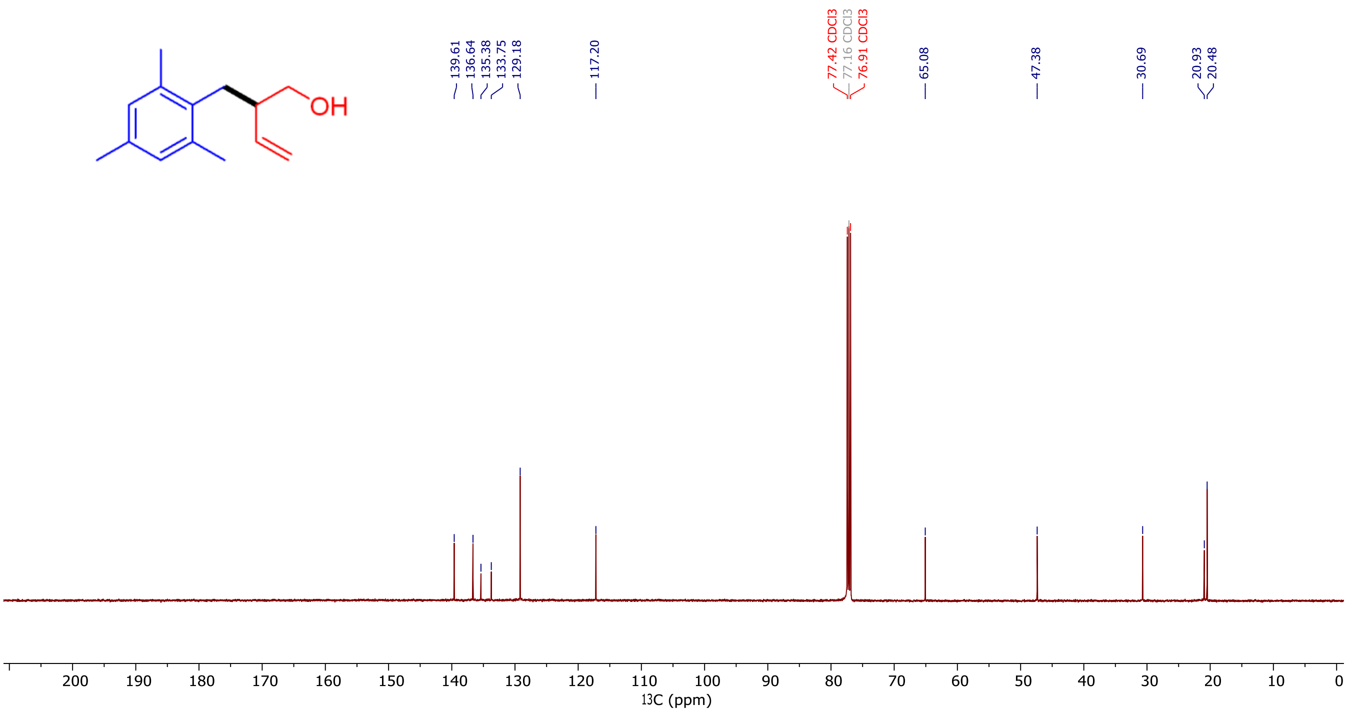

^1H NMR (500 MHz, $CDCl_3$) spectrum of **2-(5-bromo-2,4-dimethoxybenzyl)but-3-en-1-ol (3ax)**

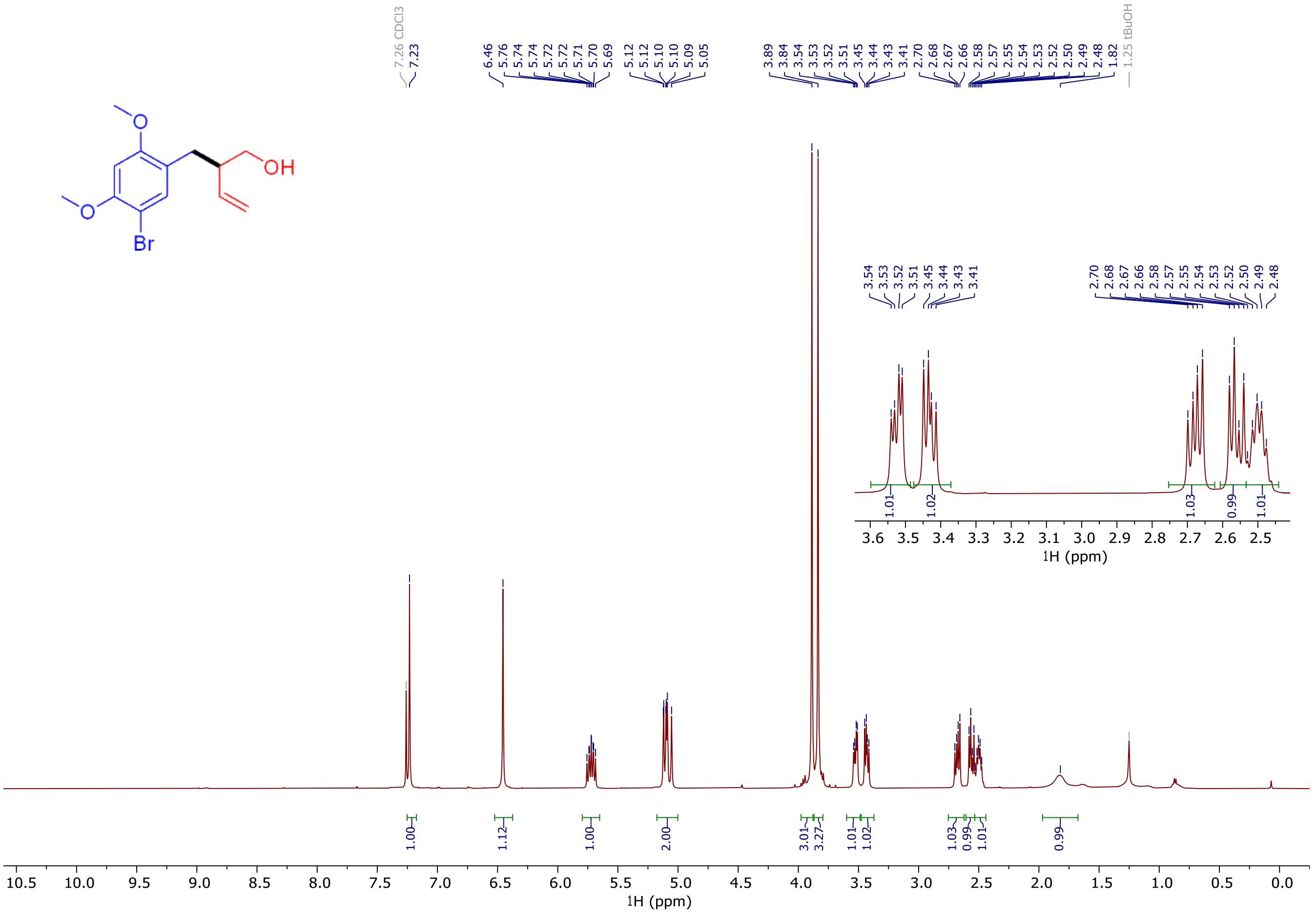

$^{13}C\{^{1}H\}$ NMR (126 MHz, $CDCl_3$) spectrum of **2-(5-bromo-2,4-dimethoxybenzyl)but-3-en-1-ol (3ax)**

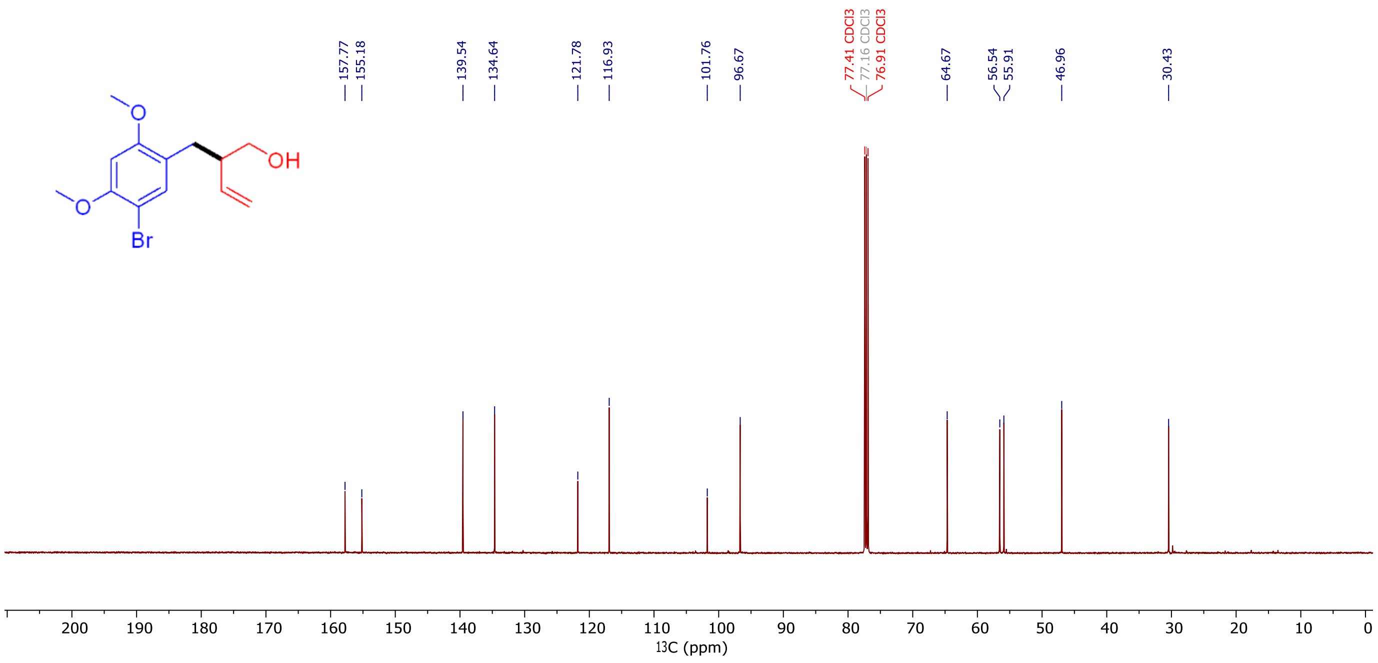

^{1}H NMR (500 MHz, $CDCl_3$) spectrum of **2-(3,5-dimethylbenzyl)but-3-en-1-ol (3ay)**

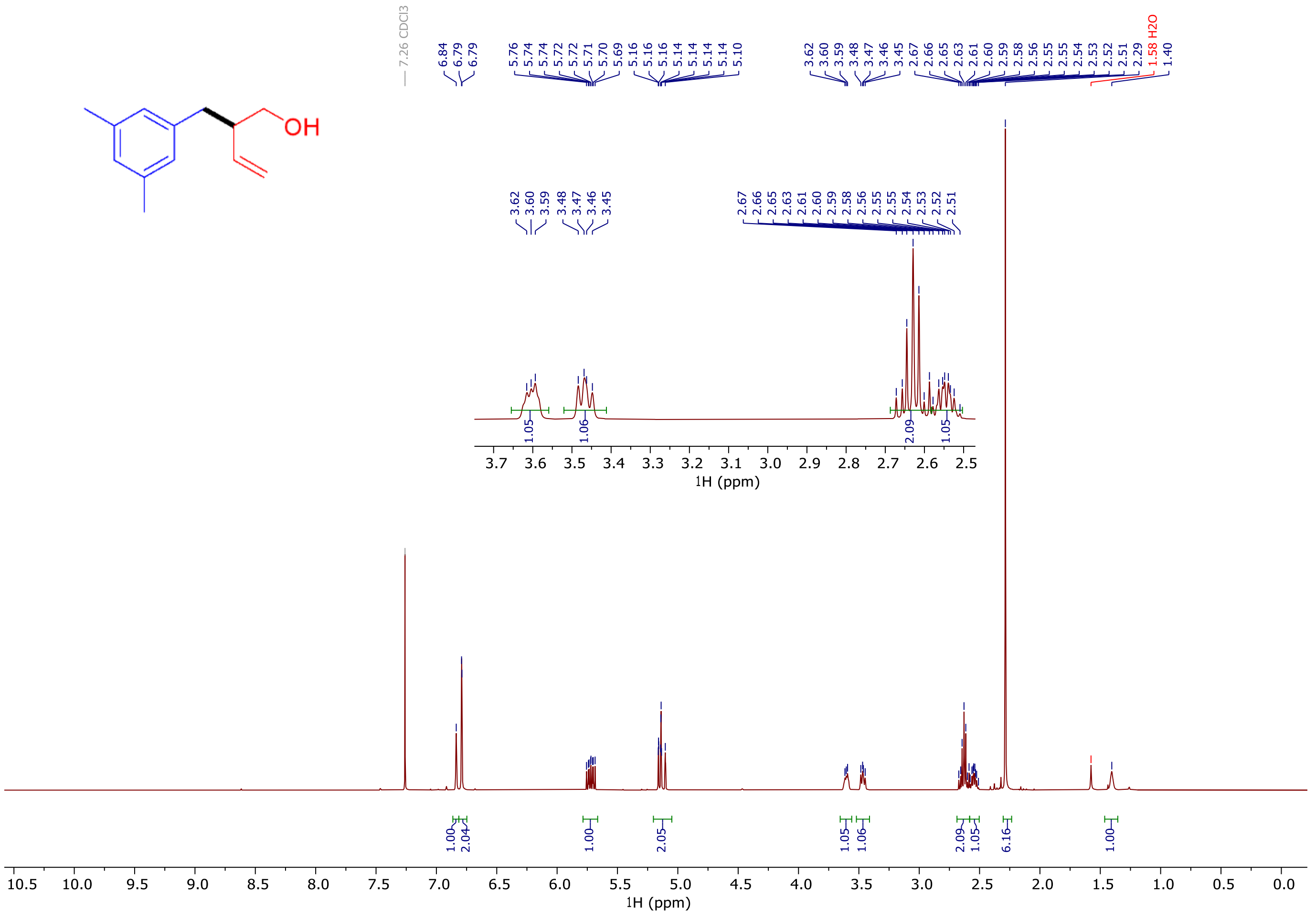

$^{13}C\{^1H\}$ NMR (126 MHz, $CDCl_3$) spectrum of **2-(3,5-dimethylbenzyl)but-3-en-1-ol (3ay)**

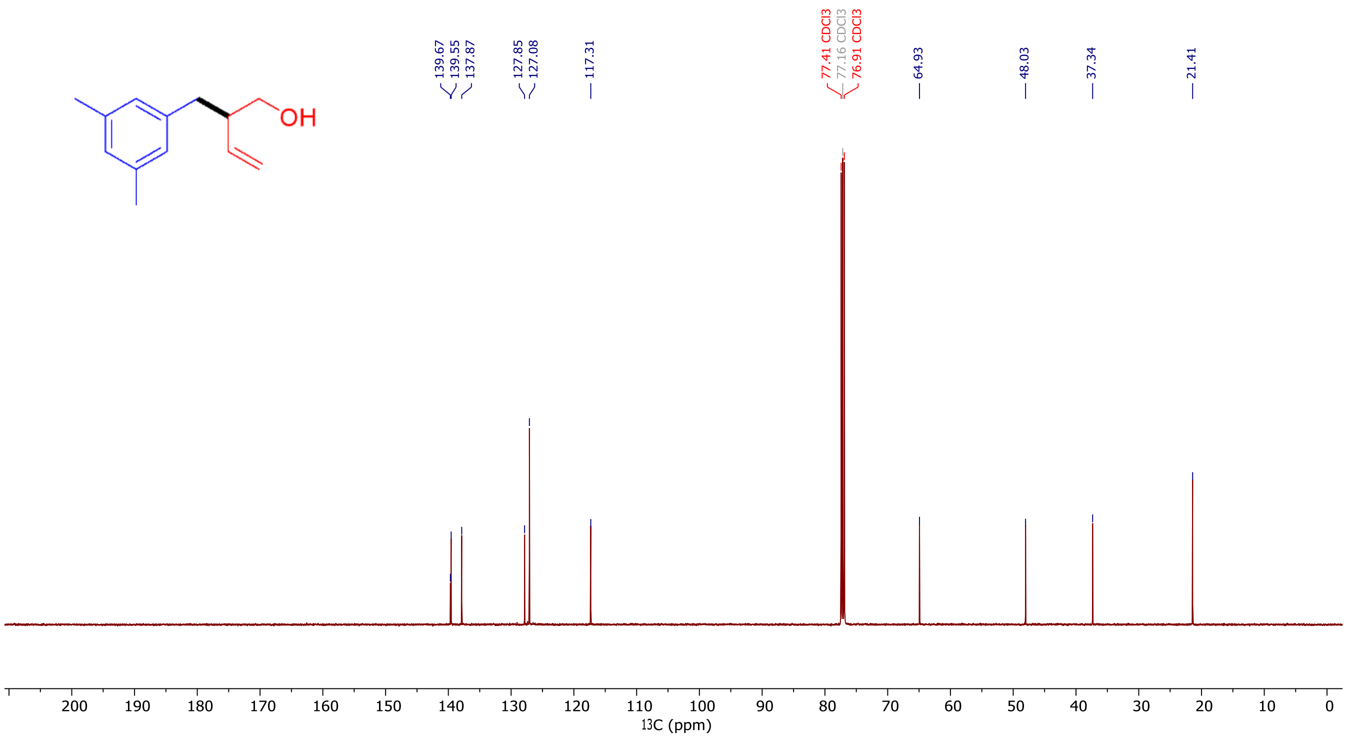

^1H NMR (500 MHz, $CDCl_3$) spectrum of **2-(furan-2-ylmethyl)but-3-en-1-ol (3az)**

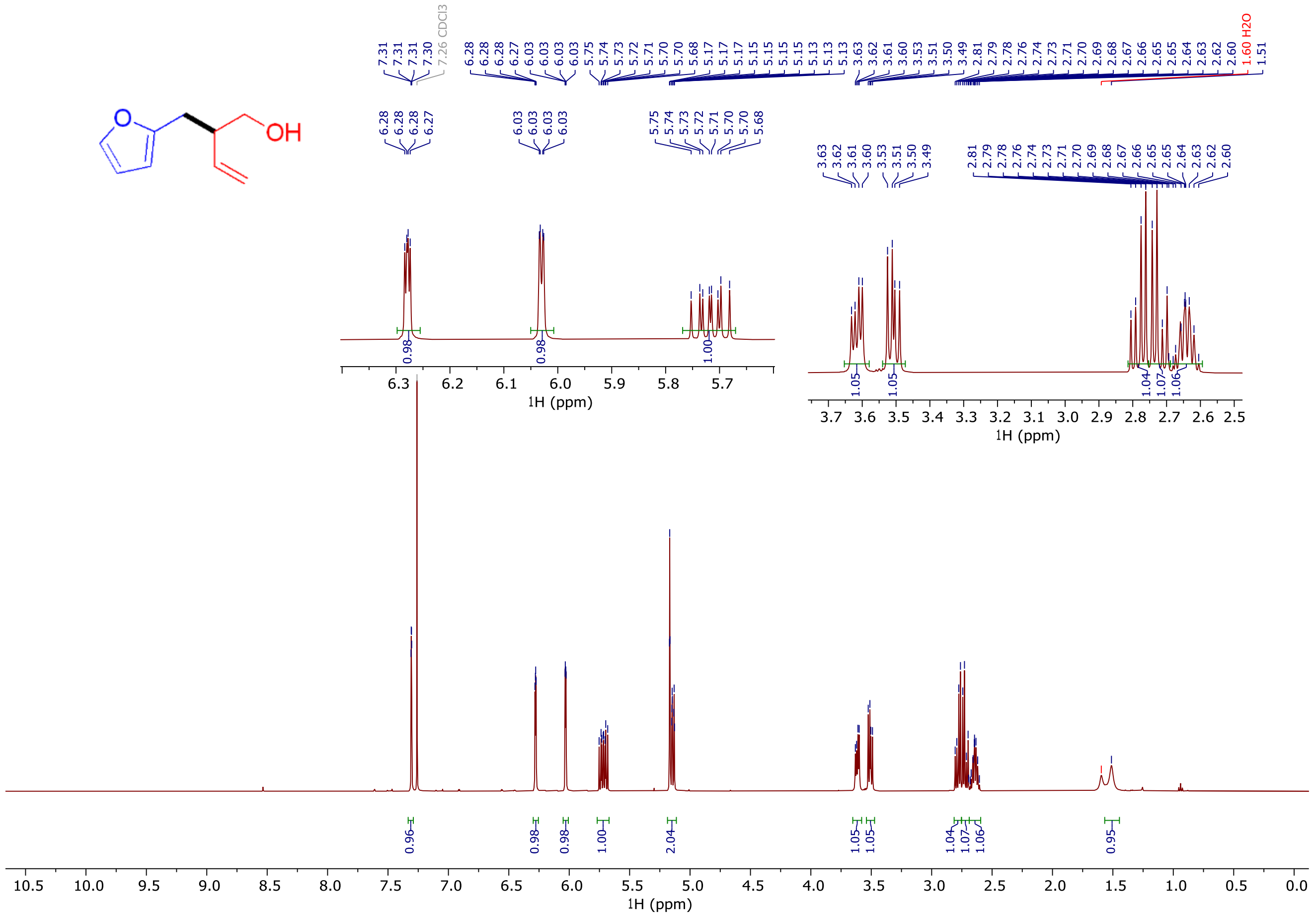

$^{13}C\{^{1}H\}$ NMR (126 MHz, $CDCl_3$) spectrum of **2-(furan-2-ylmethyl)but-3-en-1-ol (3az)**

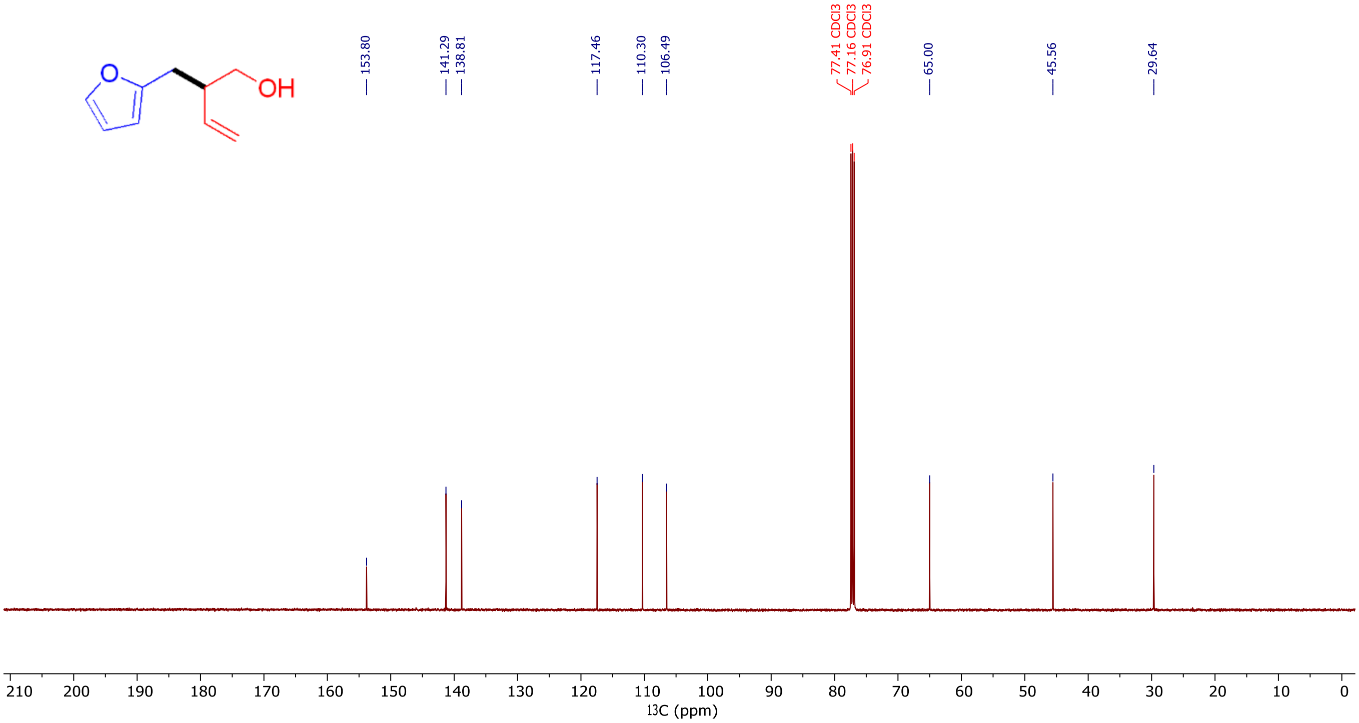

^{1}H NMR (500 MHz, $CDCl_3$) spectrum of **2-(benzofuran-2-ylmethyl)but-3-en-1-ol (3ba)**

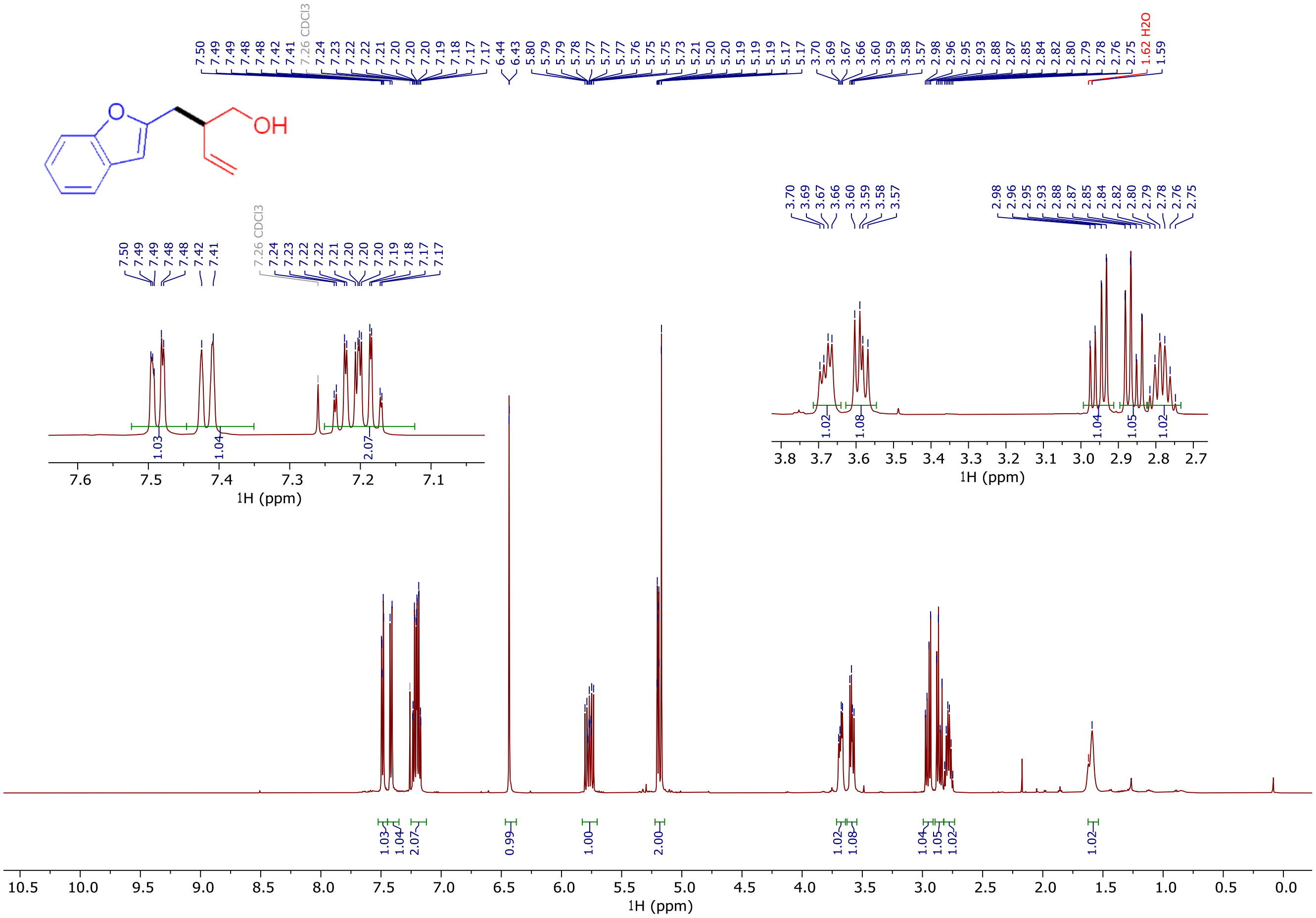

$^{13}C\{^{1}H\}$ NMR (126 MHz, $CDCl_3$) spectrum of **2-(benzofuran-2-ylmethyl)but-3-en-1-ol (3ba)**

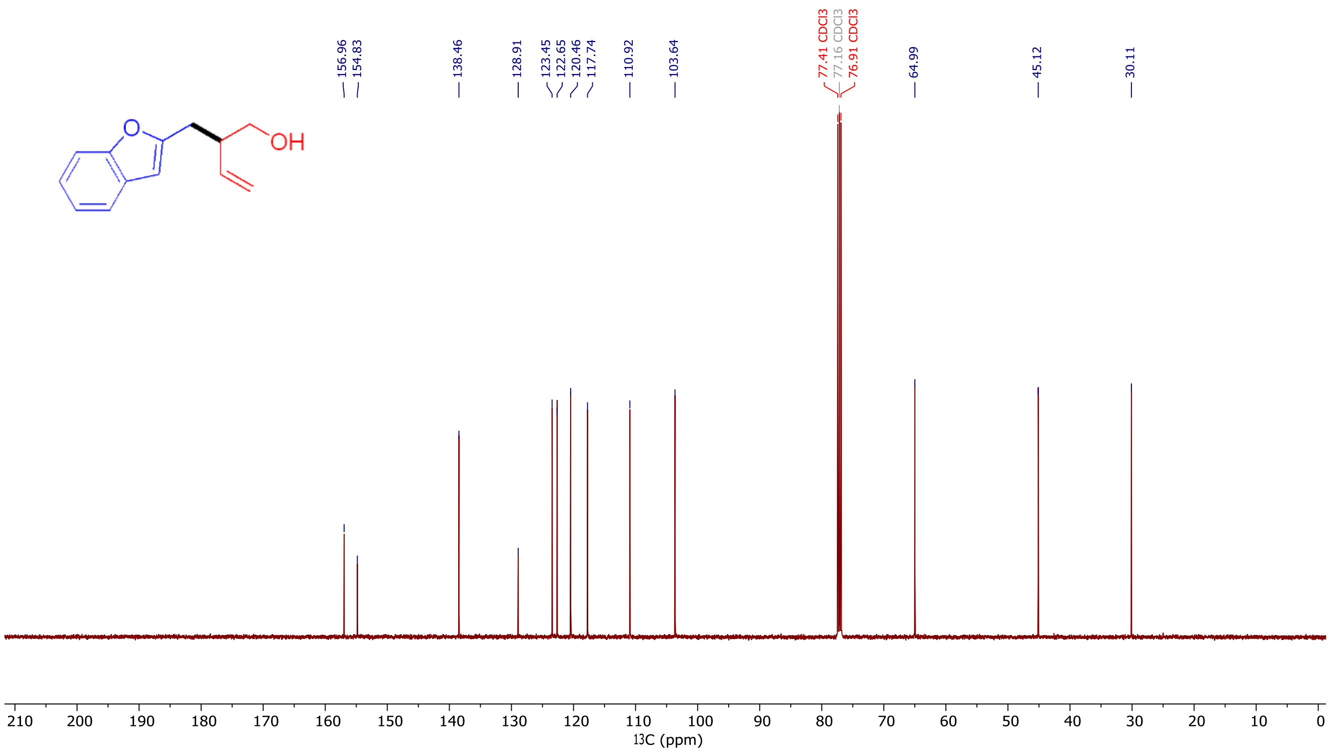

^{1}H NMR (500 MHz, $CDCl_3$) spectrum of **2-(benzothiophen-2-ylmethyl)but-3-en-1-ol (3bb)**

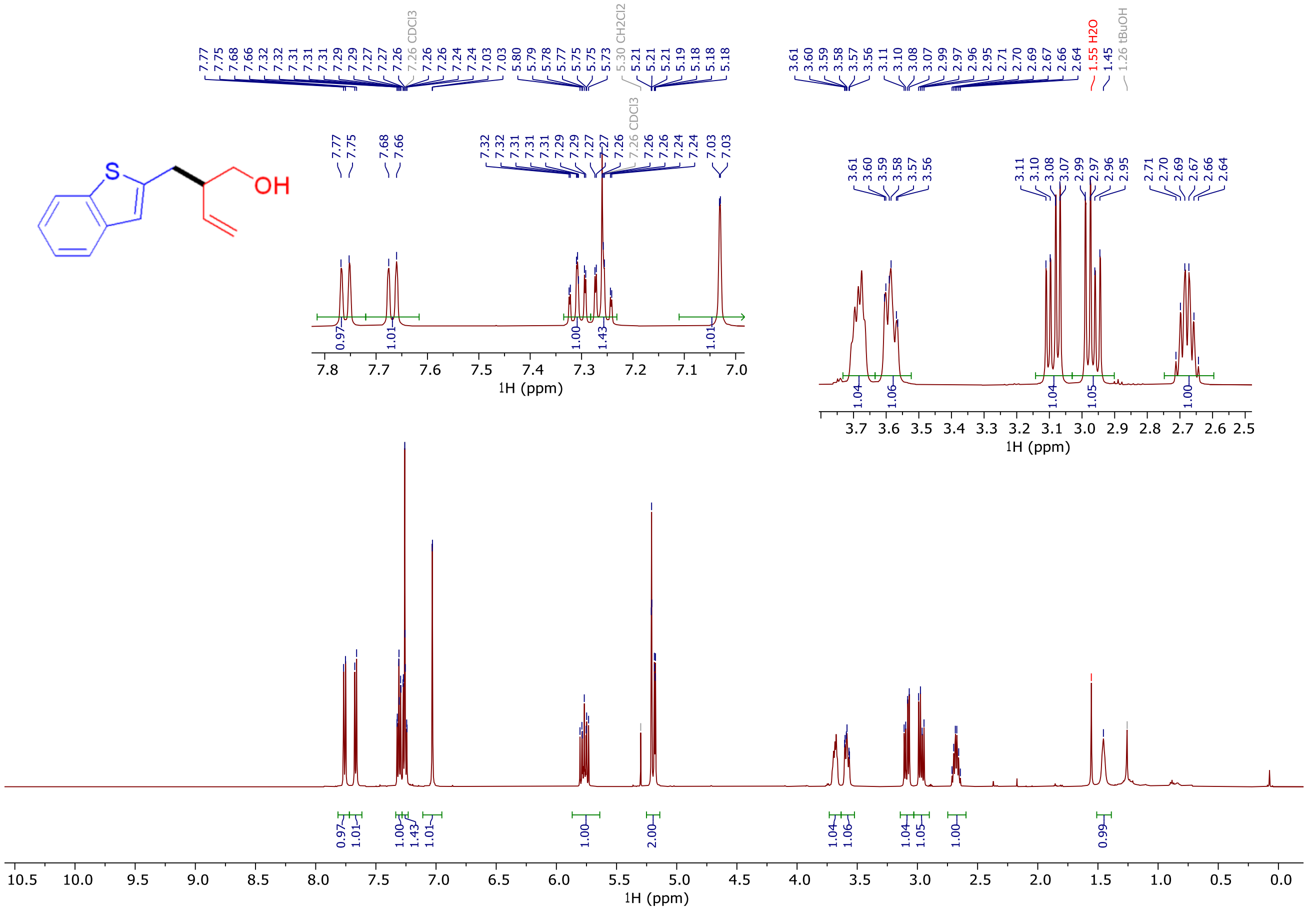

$^{13}C\{^{1}H\}$ NMR (126 MHz, $CDCl_3$) spectrum of **2-(benzothiophen-2-ylmethyl)but-3-en-1-ol (3bb)**

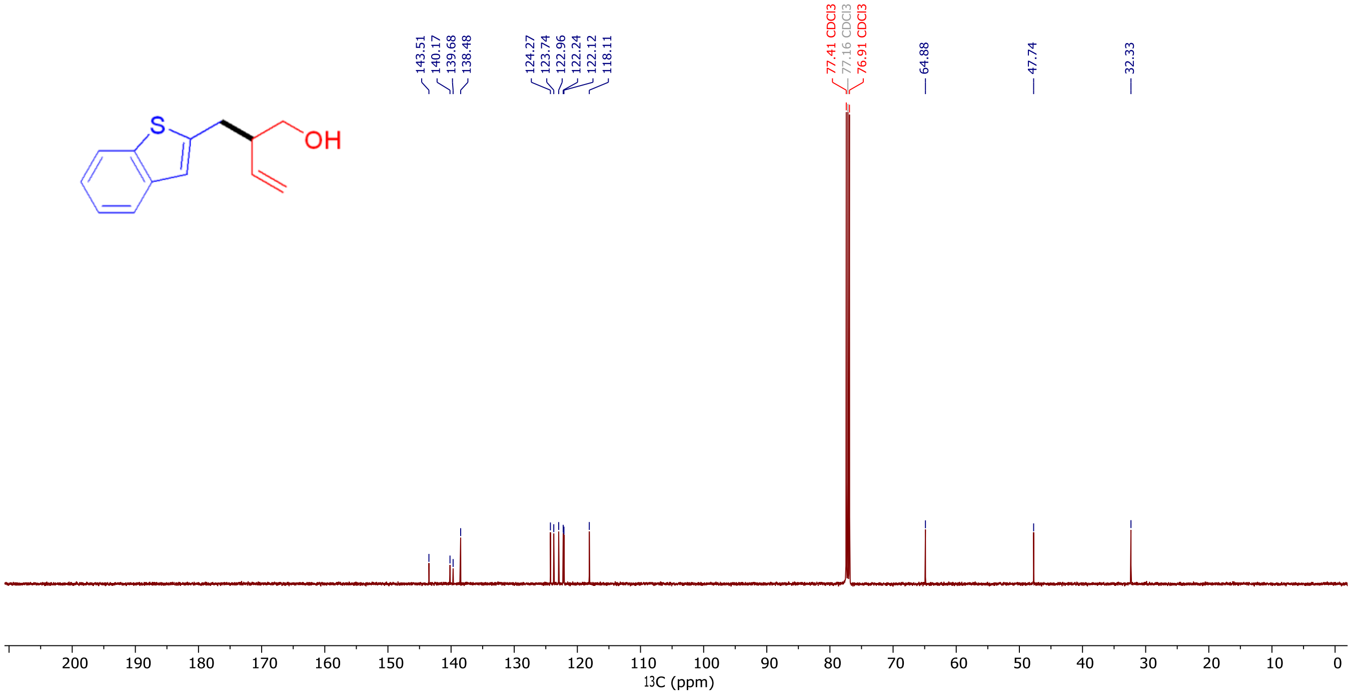

^{1}H NMR (500 MHz, $CDCl_3$) spectrum of **2-(pyridin-2-ylmethyl)but-3-en-1-ol (3bd)**

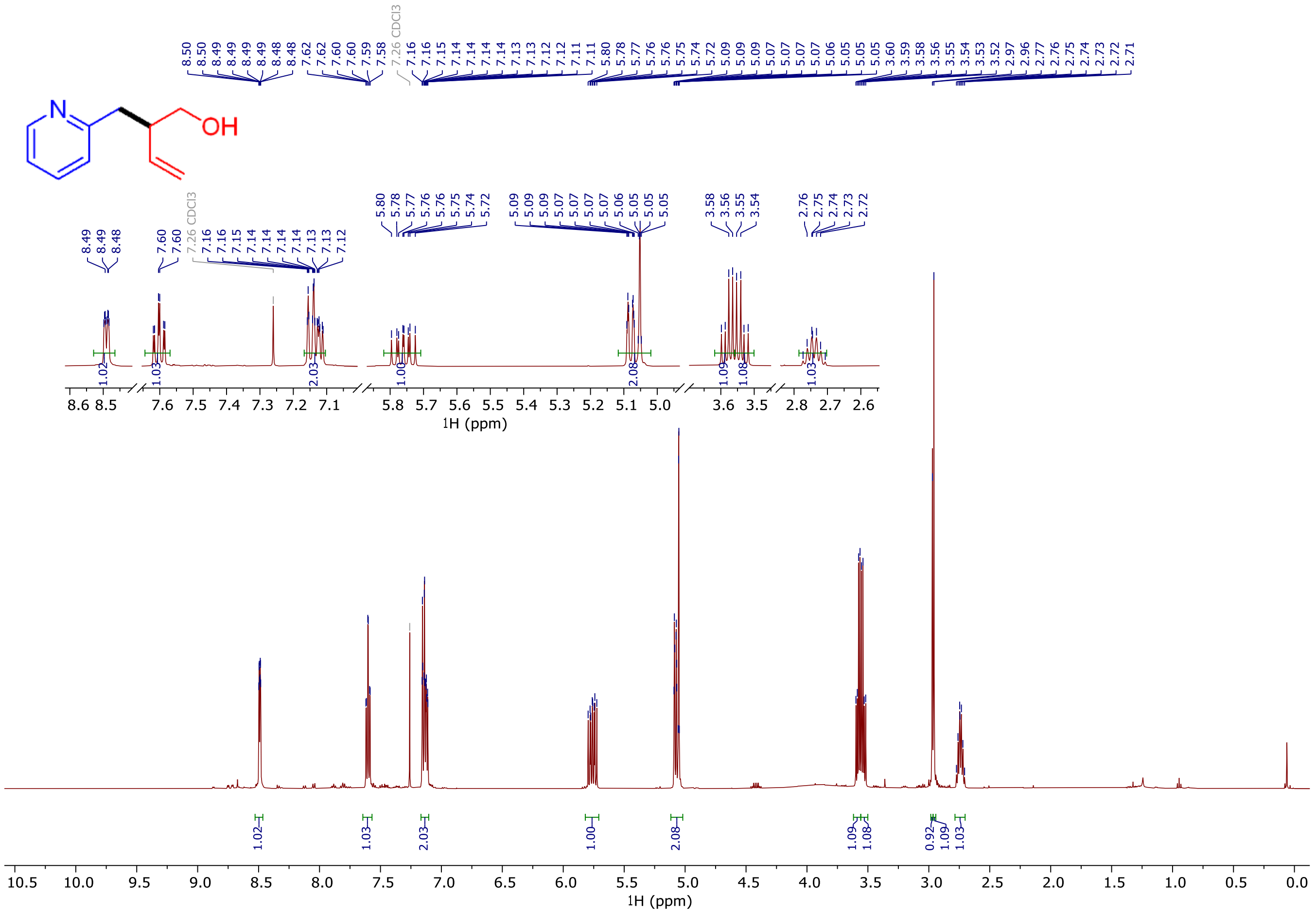

$^{13}C\{^{1}H\}$ NMR (126 MHz, $CDCl_3$) spectrum of **2-(pyridin-2-ylmethyl)but-3-en-1-ol (3bd)**

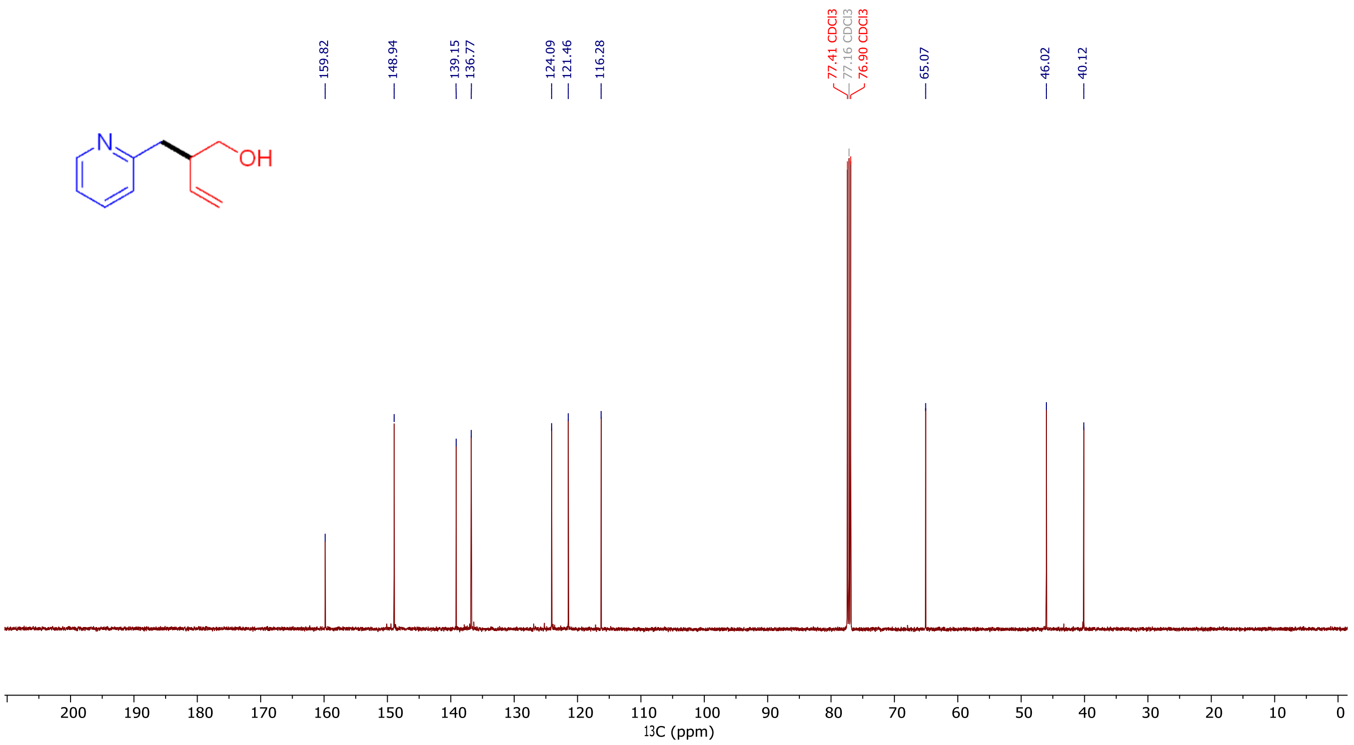

^{1}H NMR (500 MHz, $CDCl_3$) spectrum of **2-(cyclohexylmethyl)but-3-en-1-ol (3be)**

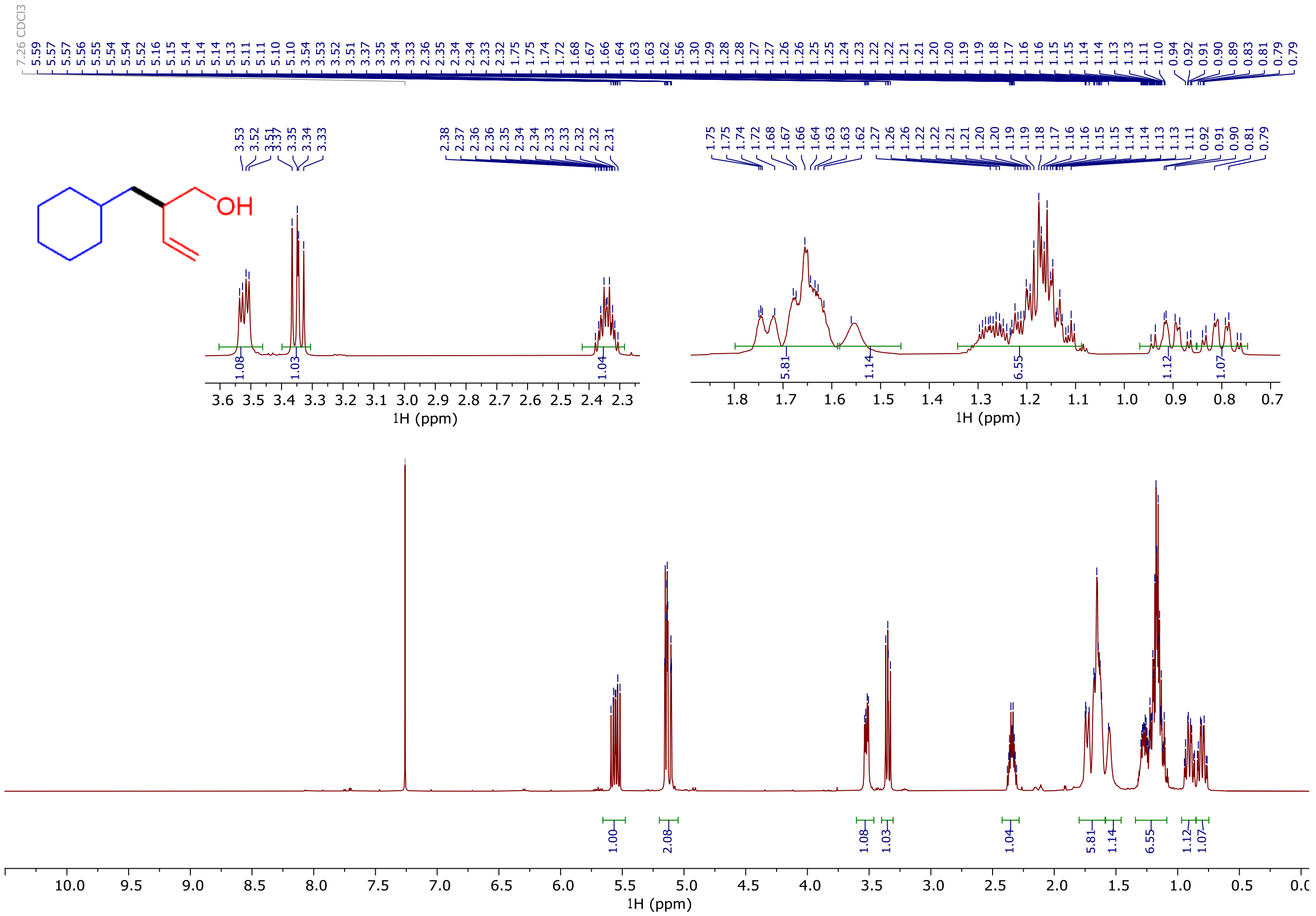

$^{13}C\{^1H\}$ NMR (126 MHz, $CDCl_3$) spectrum of **2-(cyclohexylmethyl)but-3-en-1-ol (3be)**

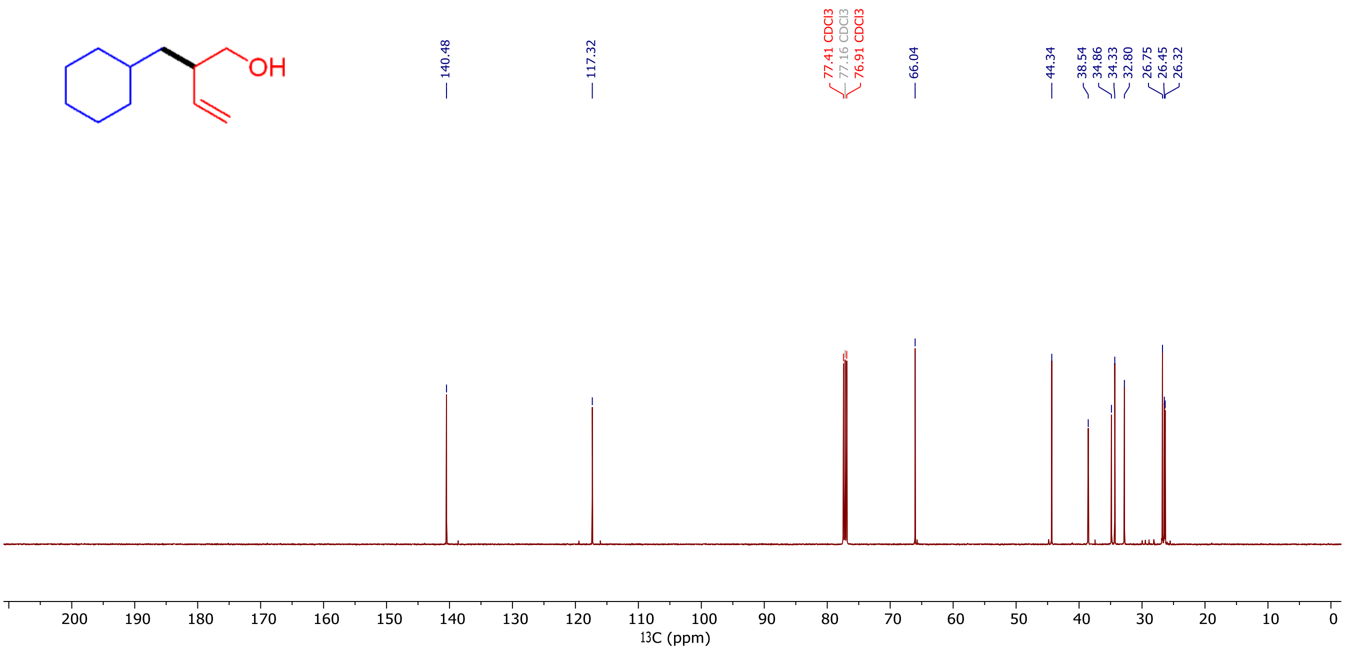

^1H NMR (800 MHz, $CDCl_3$) spectrum of **2-phenethylbut-3-en-1-yl acetate (3bf)**

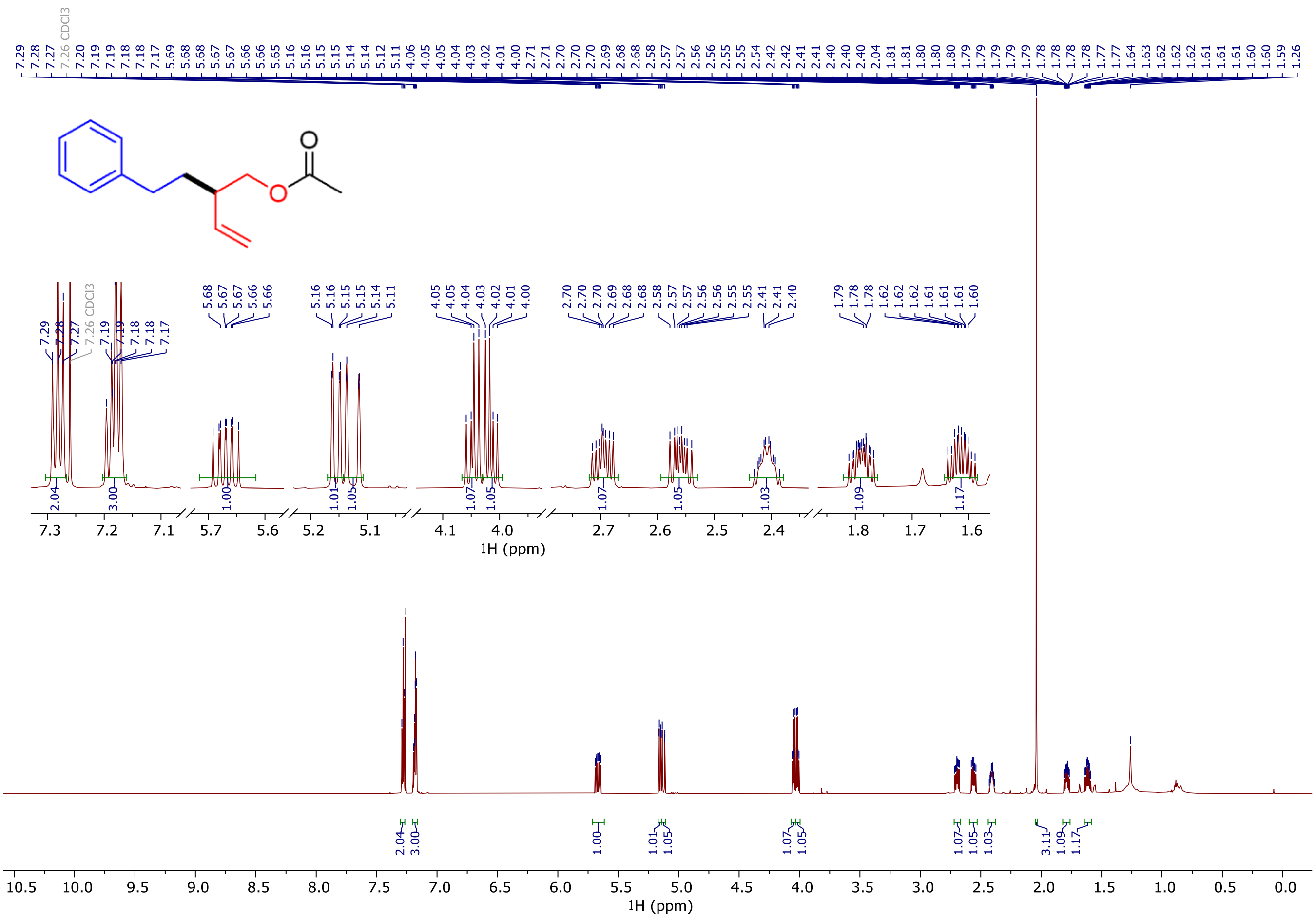

$^{13}C\{^1H\}$ NMR (201 MHz, $CDCl_3$) spectrum of **2-phenethylbut-3-en-1-yl acetate (3bf)**

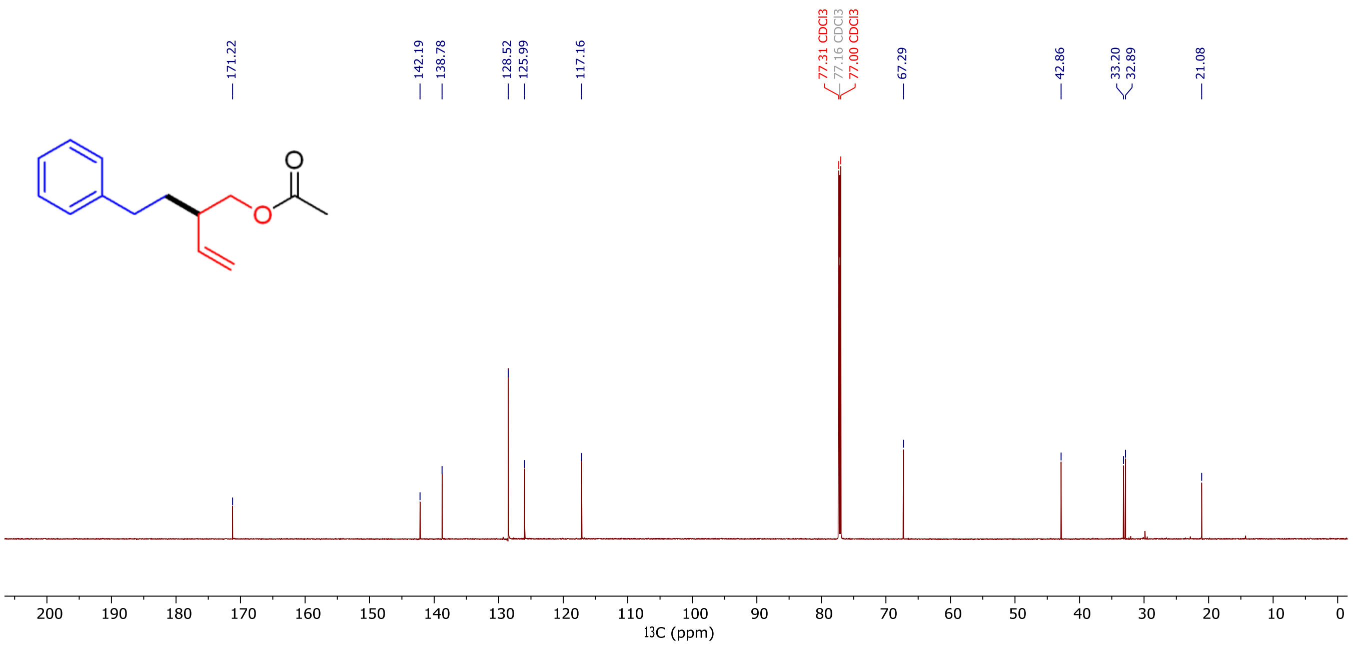

^1H NMR (500 MHz, $CDCl_3$) spectrum of **5-phenyl-2-vinylpentyl acetate (3bg)**

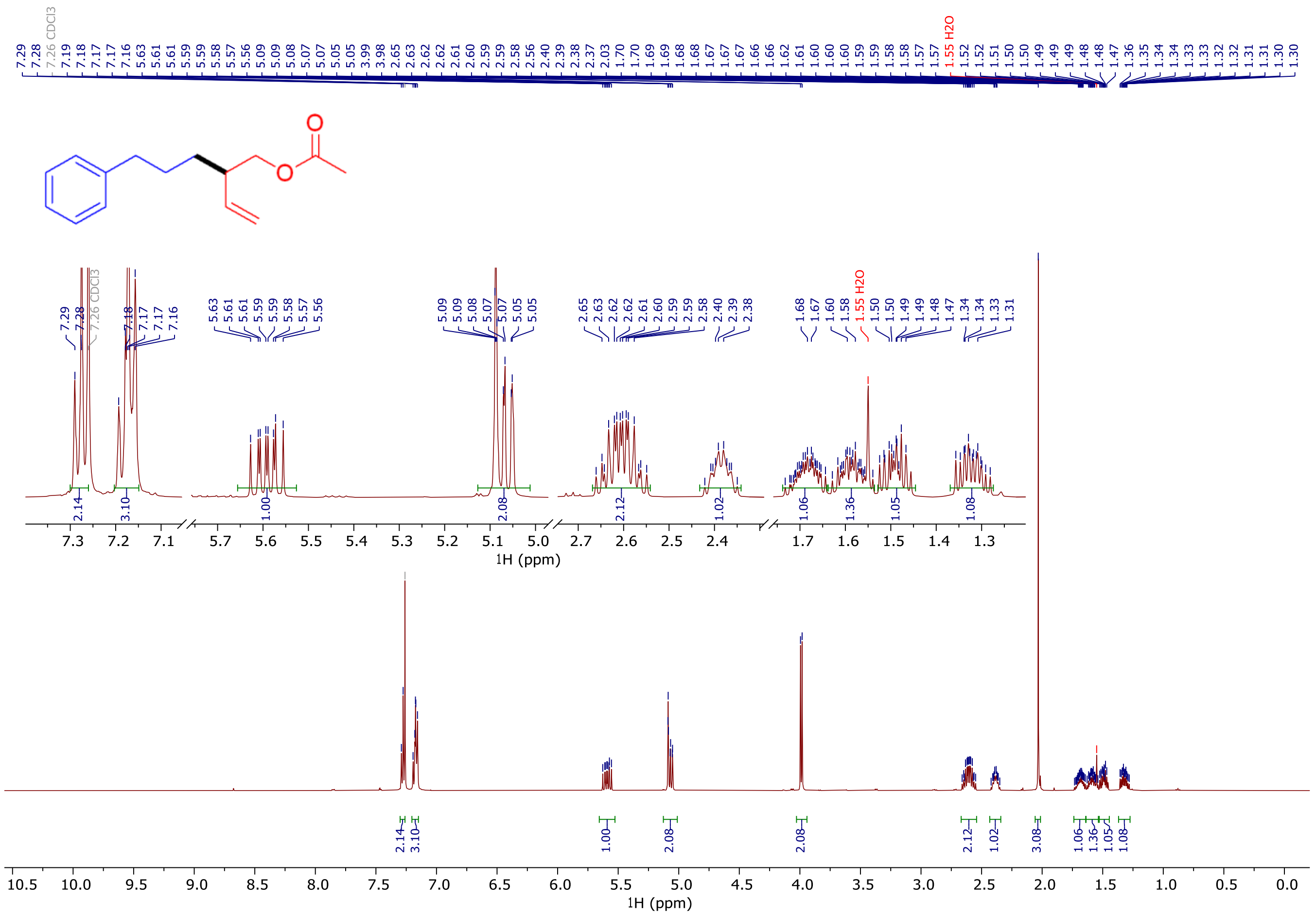

$^{13}C\{^{1}H\}$ NMR (126 MHz, $CDCl_3$) spectrum of **5-phenyl-2-vinylpentyl acetate (3bg)**

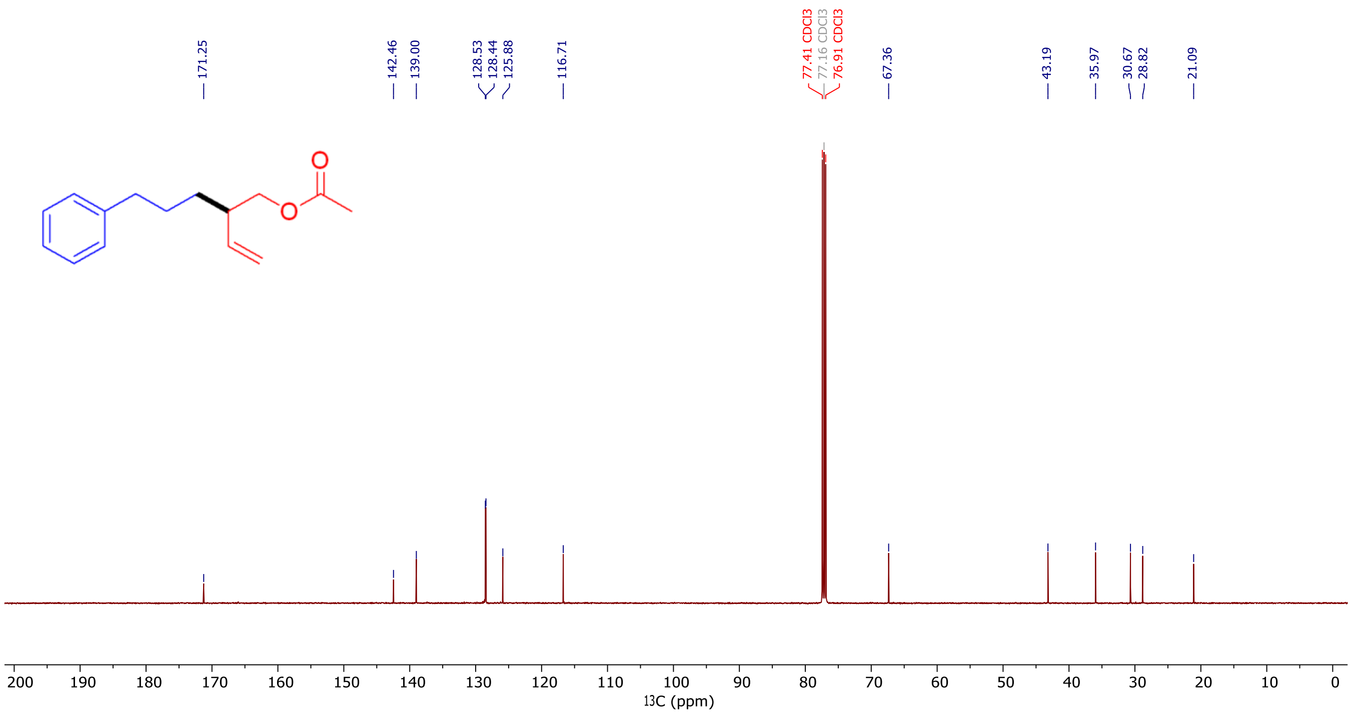

^{1}H NMR (500 MHz, $CDCl_3$) spectrum of **(*Z*)-2-vinyldodec-9-en-1-ol (3bh)**

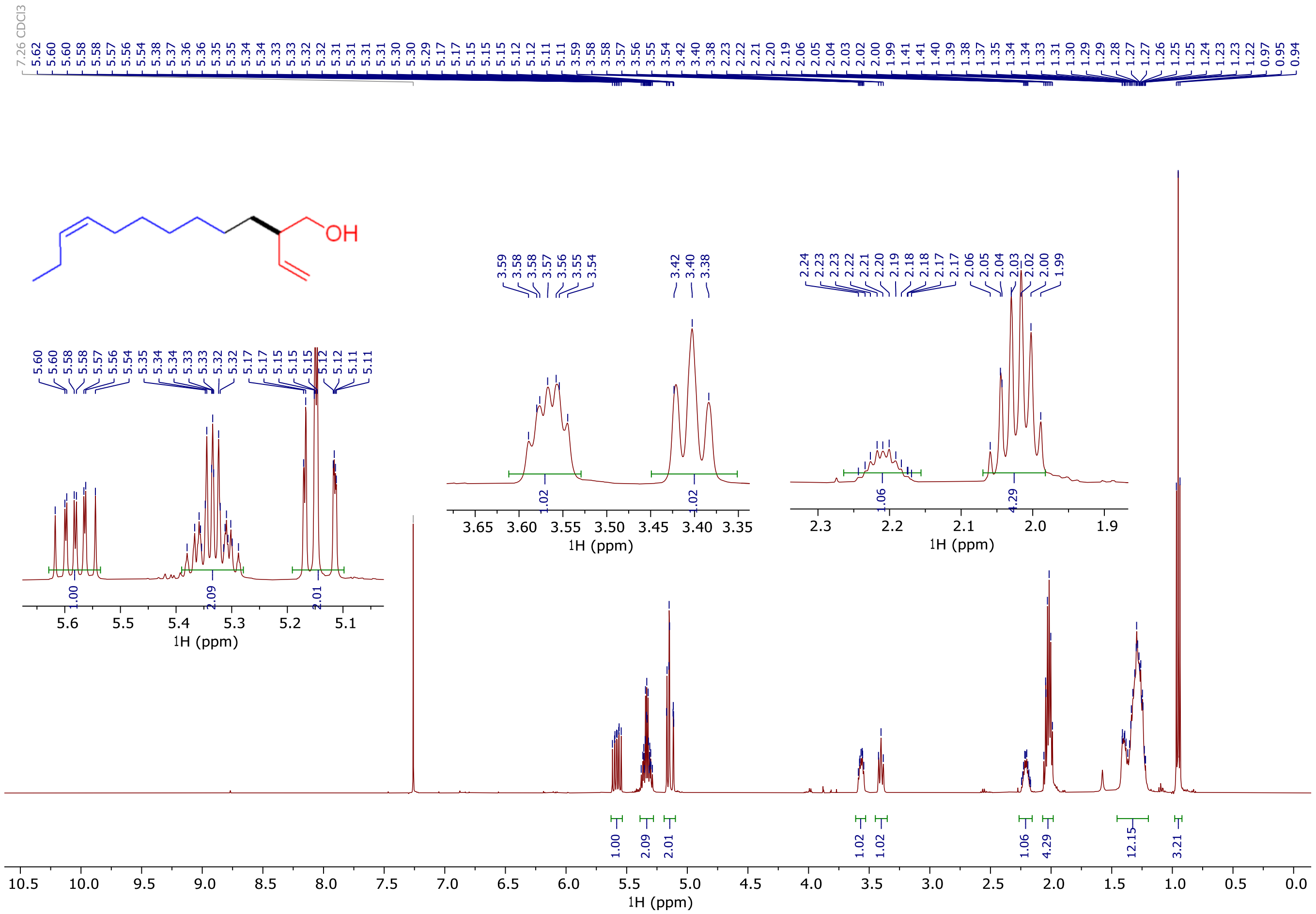

$^{13}C\{^{1}H\}$ NMR (126 MHz, $CDCl_3$) spectrum of **(*Z*)-2-vinyldodec-9-en-1-ol (3bh)**

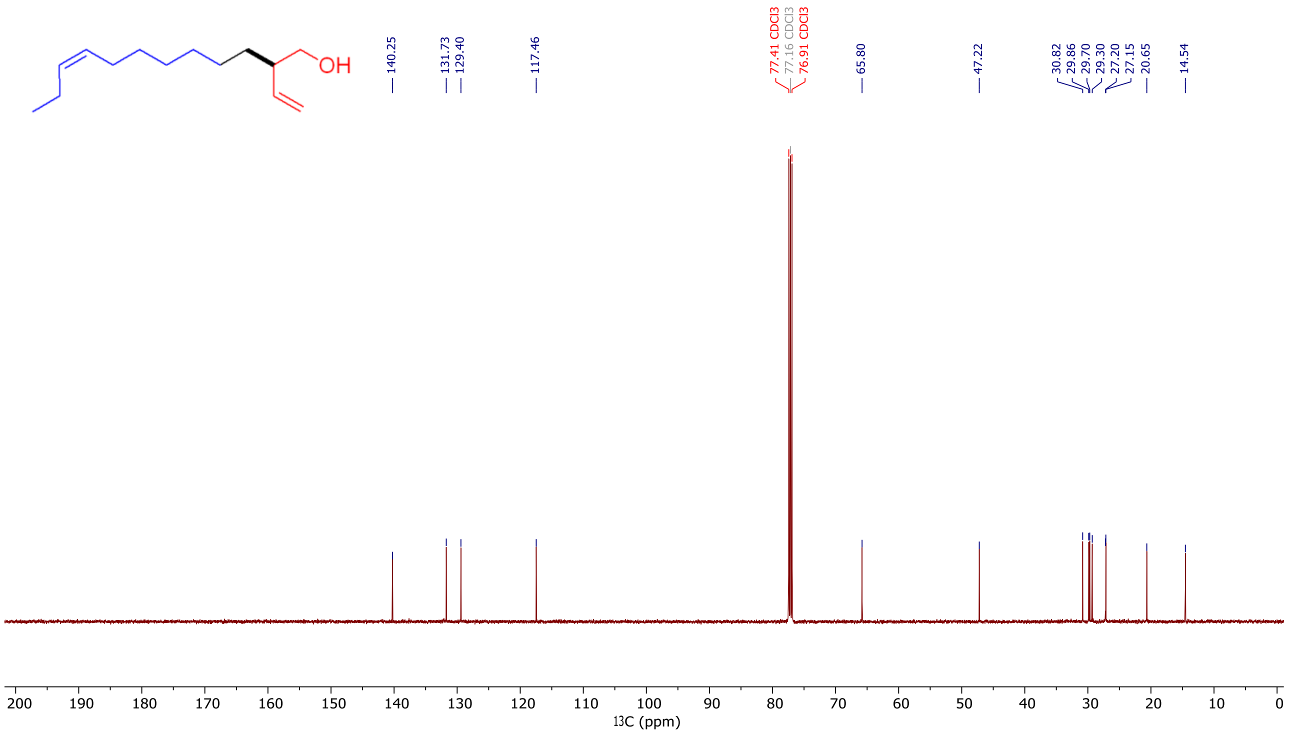

^{1}H NMR (500 MHz, $CDCl_3$) spectrum of **5-methyl-2-vinylhexan-1-ol (3bi)**

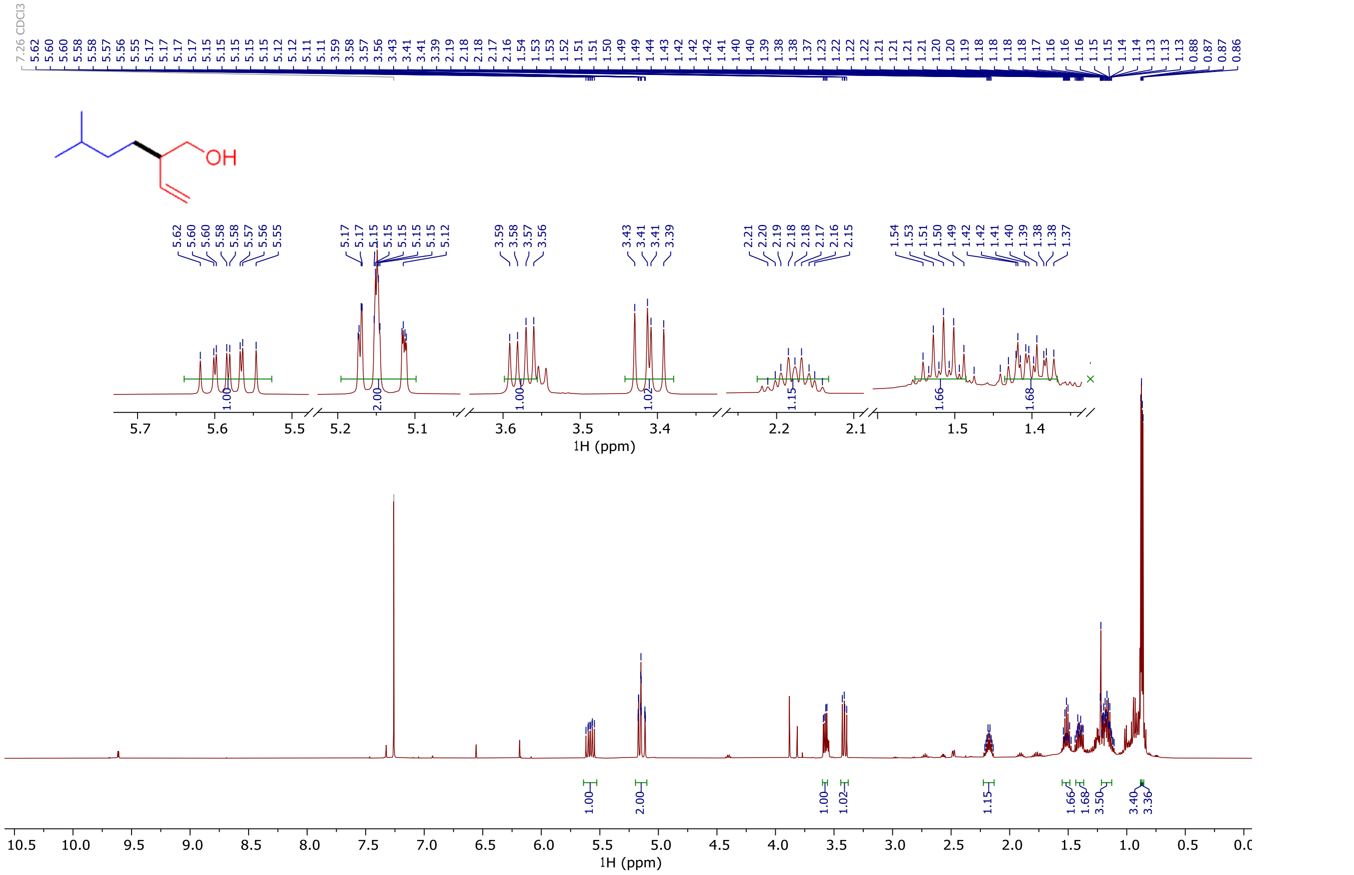

$^{13}C\{^1H\}$ NMR (126 MHz, $CDCl_3$) spectrum of **5-methyl-2-vinylhexan-1-ol (3bi)**

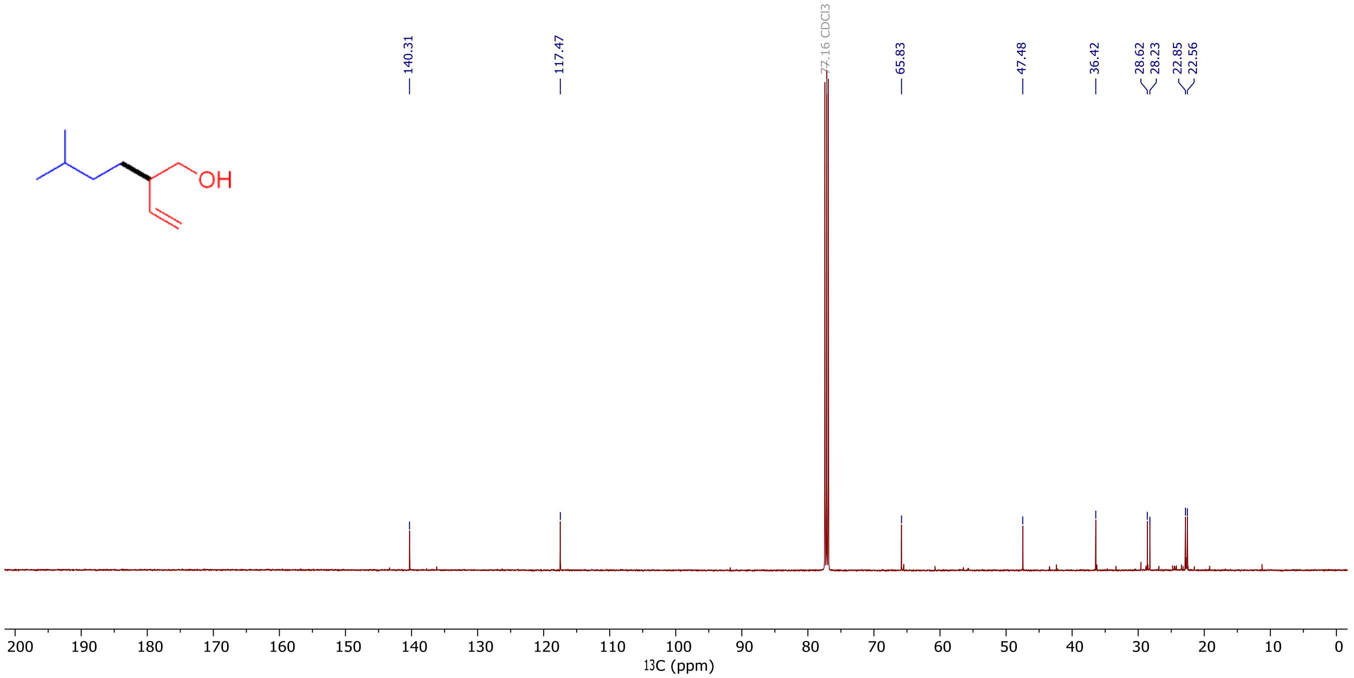

^1H NMR (500 MHz, $CDCl_3$) spectrum of **2-benzyl-2-methylbut-3-en-1-ol (3bj)**

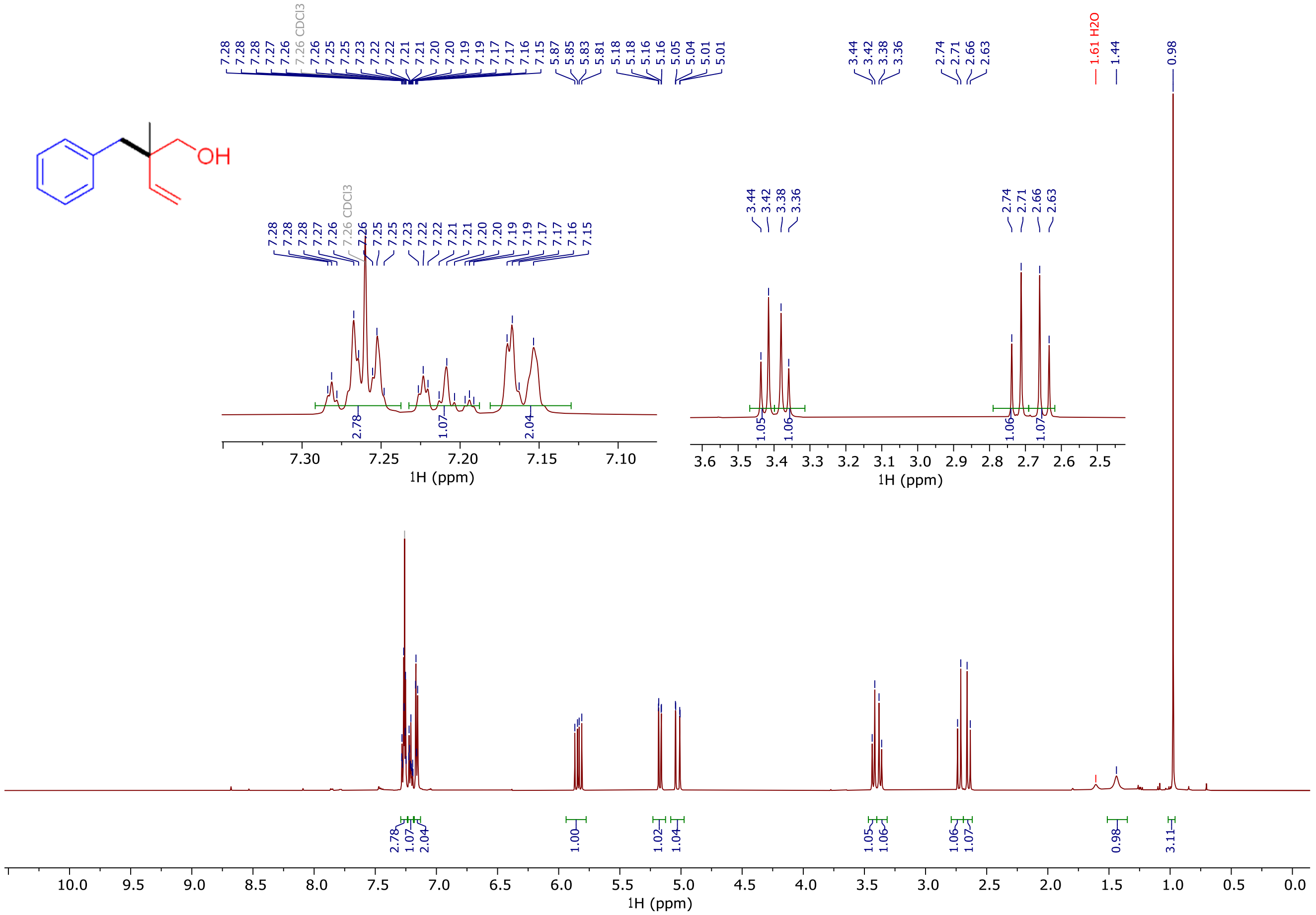

$^{13}C\{^1H\}$ NMR (126 MHz, $CDCl_3$) spectrum of **2-benzyl-2-methylbut-3-en-1-ol (3bj)**

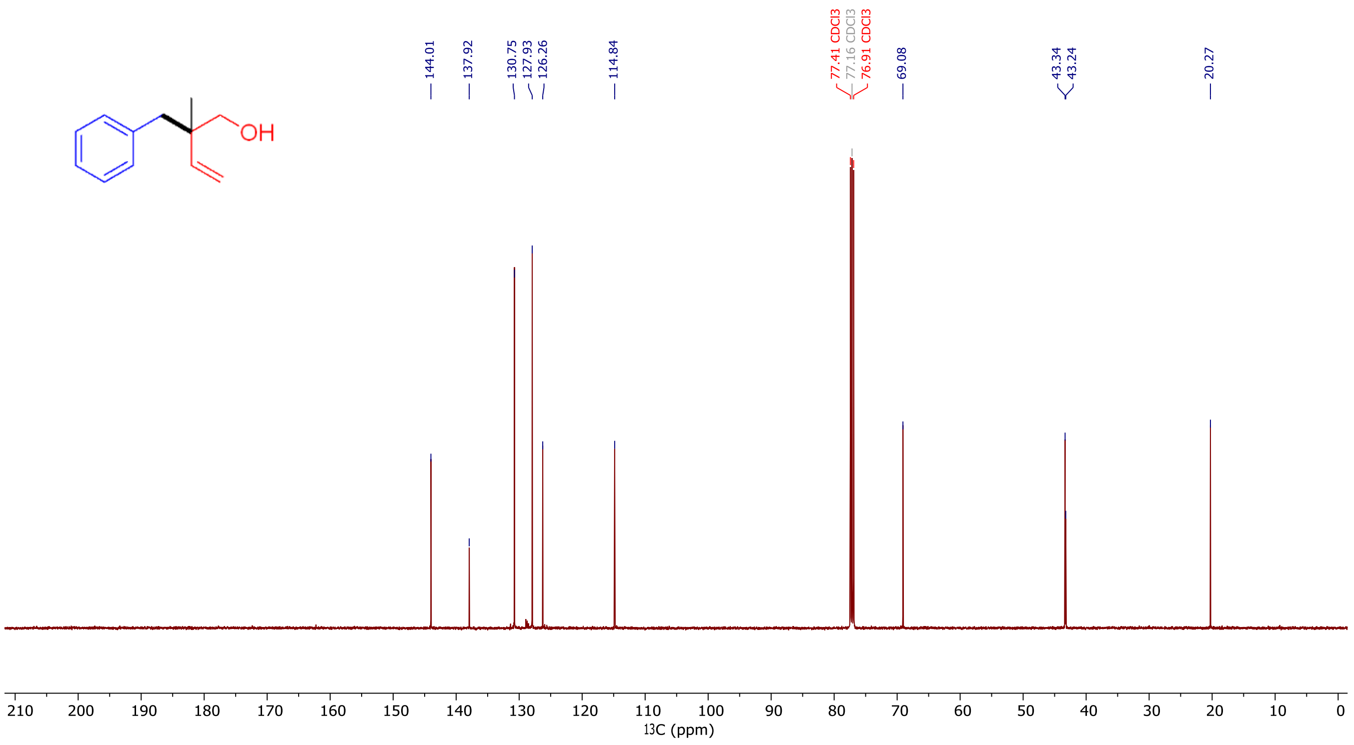

^1H NMR (500 MHz, $CDCl_3$) spectrum of **2-benzylcyclohex-3-en-1-ol (3bk)**

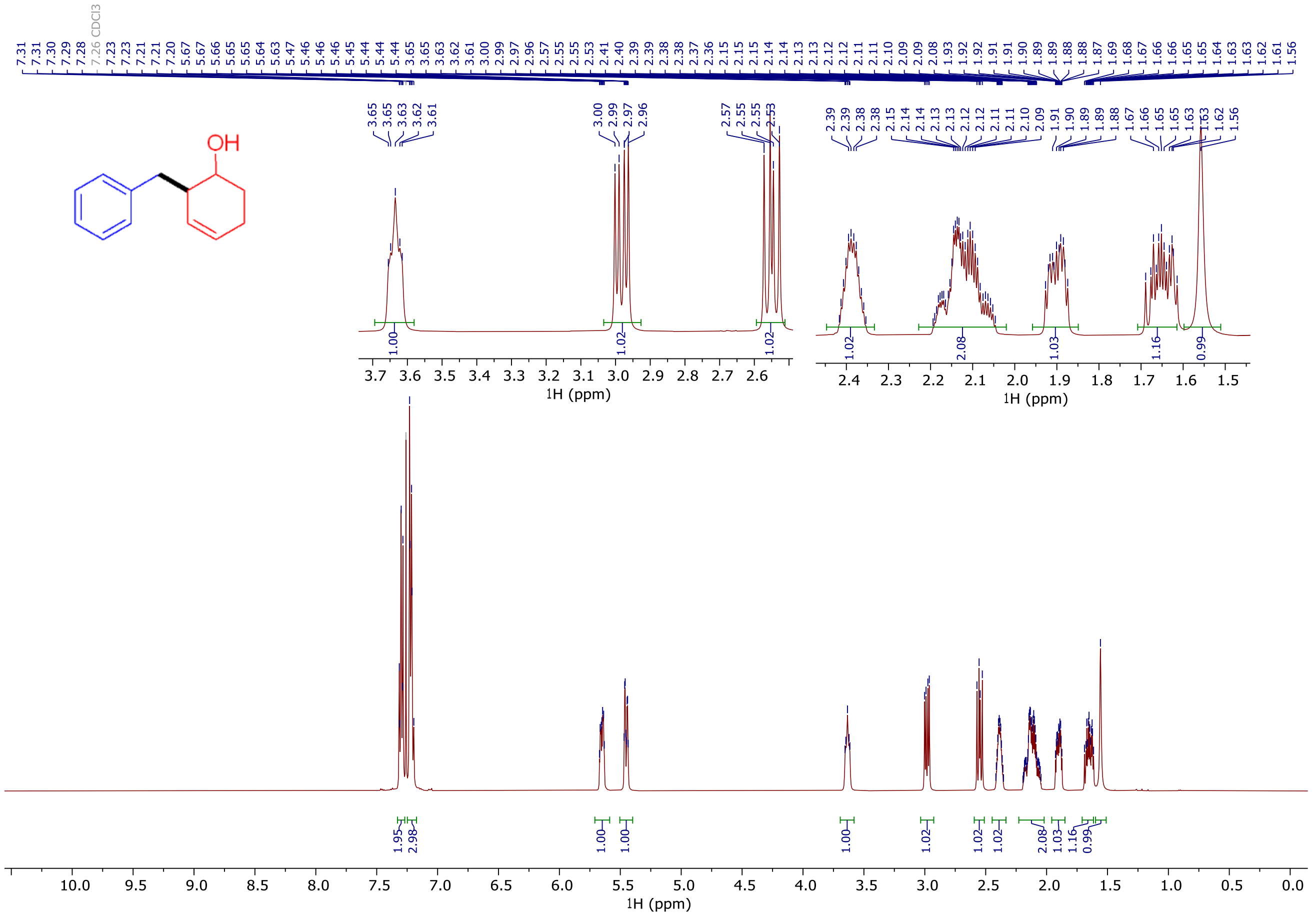

$^{13}C\{^{1}H\}$ NMR (126 MHz, $CDCl_3$) spectrum of **2-benzylcyclohex-3-en-1-ol (3bk)**

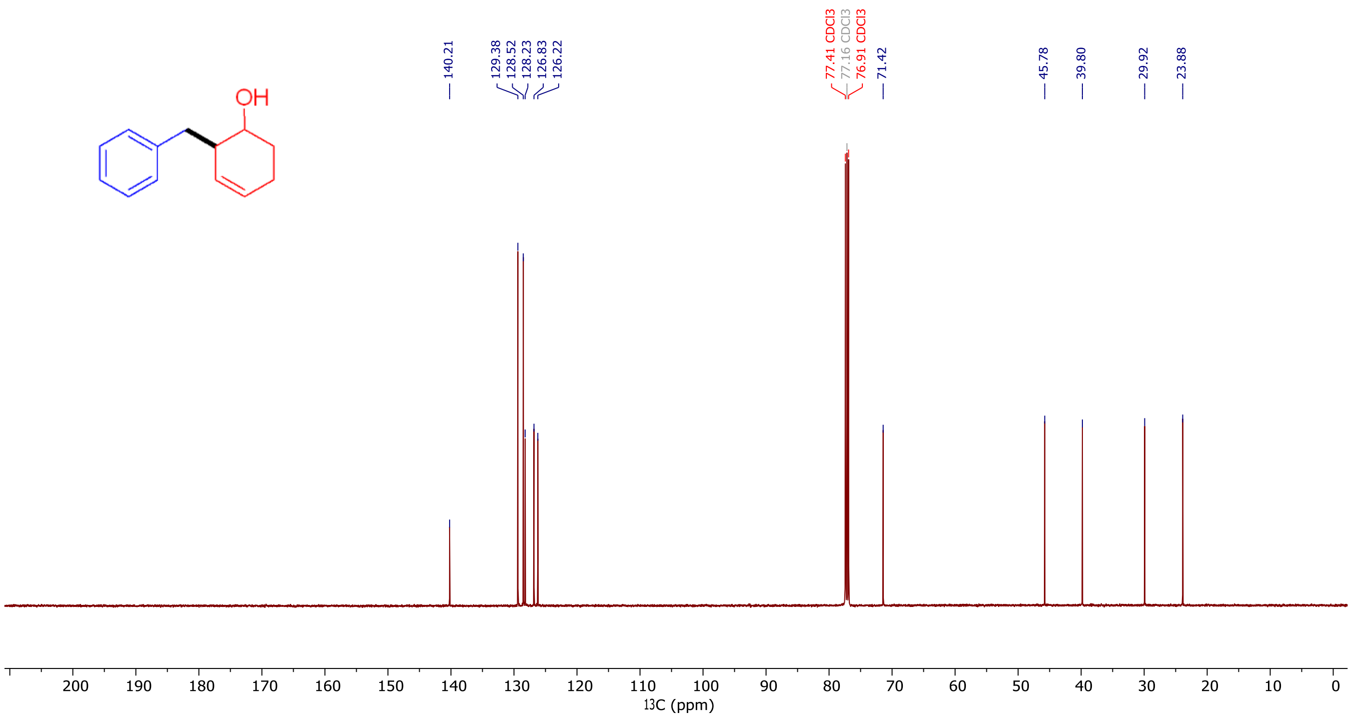

^{1}H NMR (500 MHz, $CDCl_3$) spectrum of **4-benzyl-1-phenylhex-5-en-3-ol (3bl)**

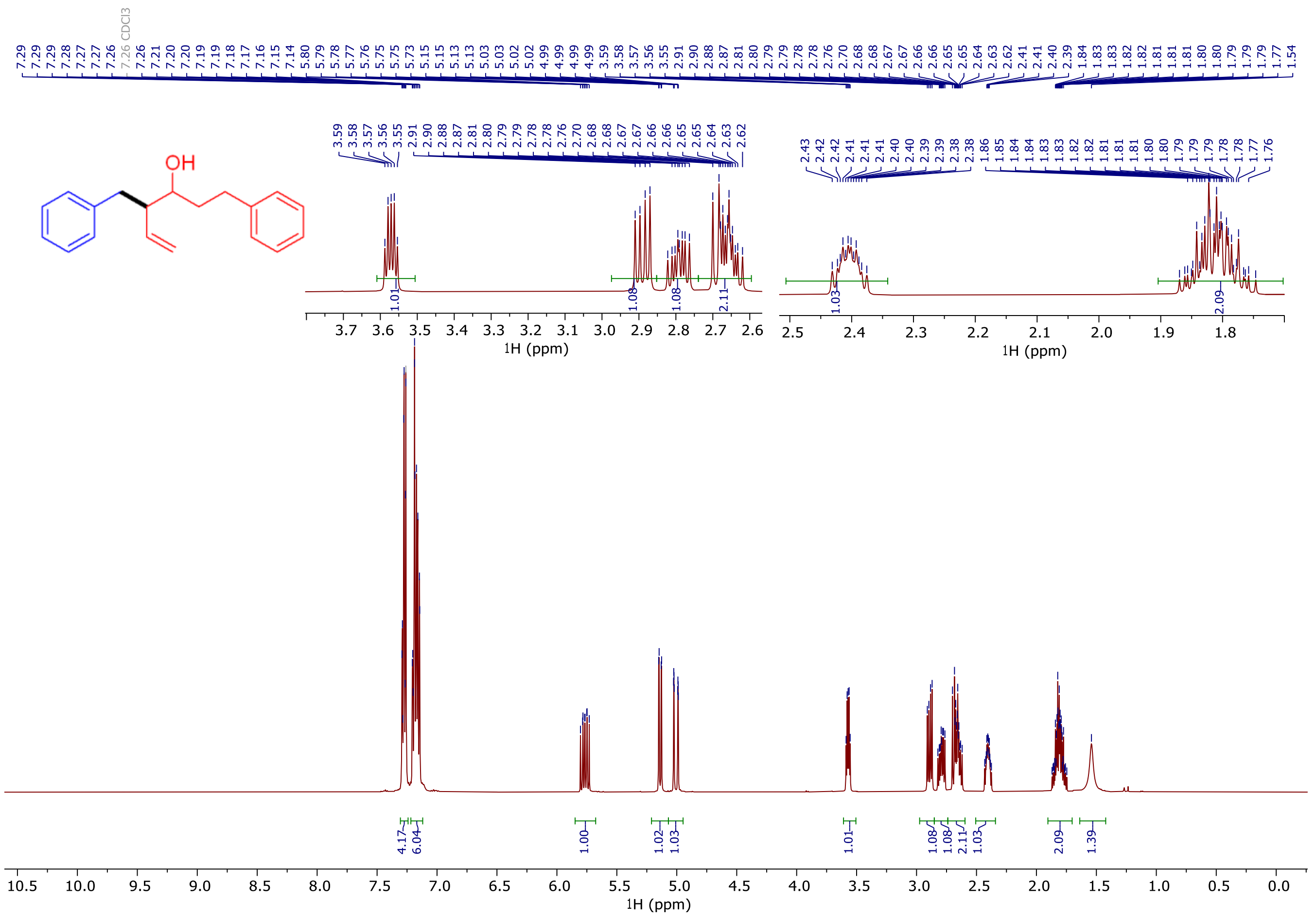

$^{13}C\{^{1}H\}$ NMR (126 MHz, $CDCl_3$) spectrum of **4-benzyl-1-phenylhex-5-en-3-ol (3bl)**

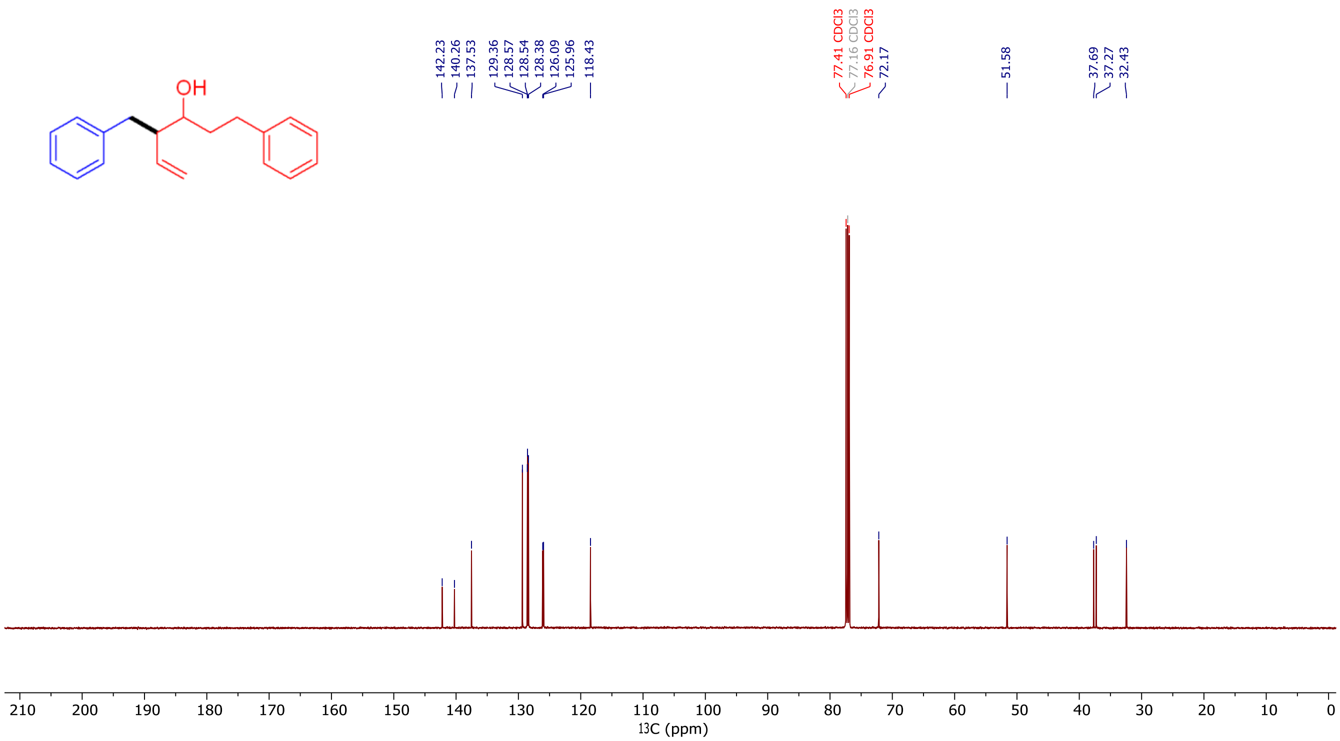

^{1}H NMR (500 MHz, $CDCl_3$) spectrum of **2-benzyl-1-cyclohexylbut-3-en-1-yl acetate (3bm)**

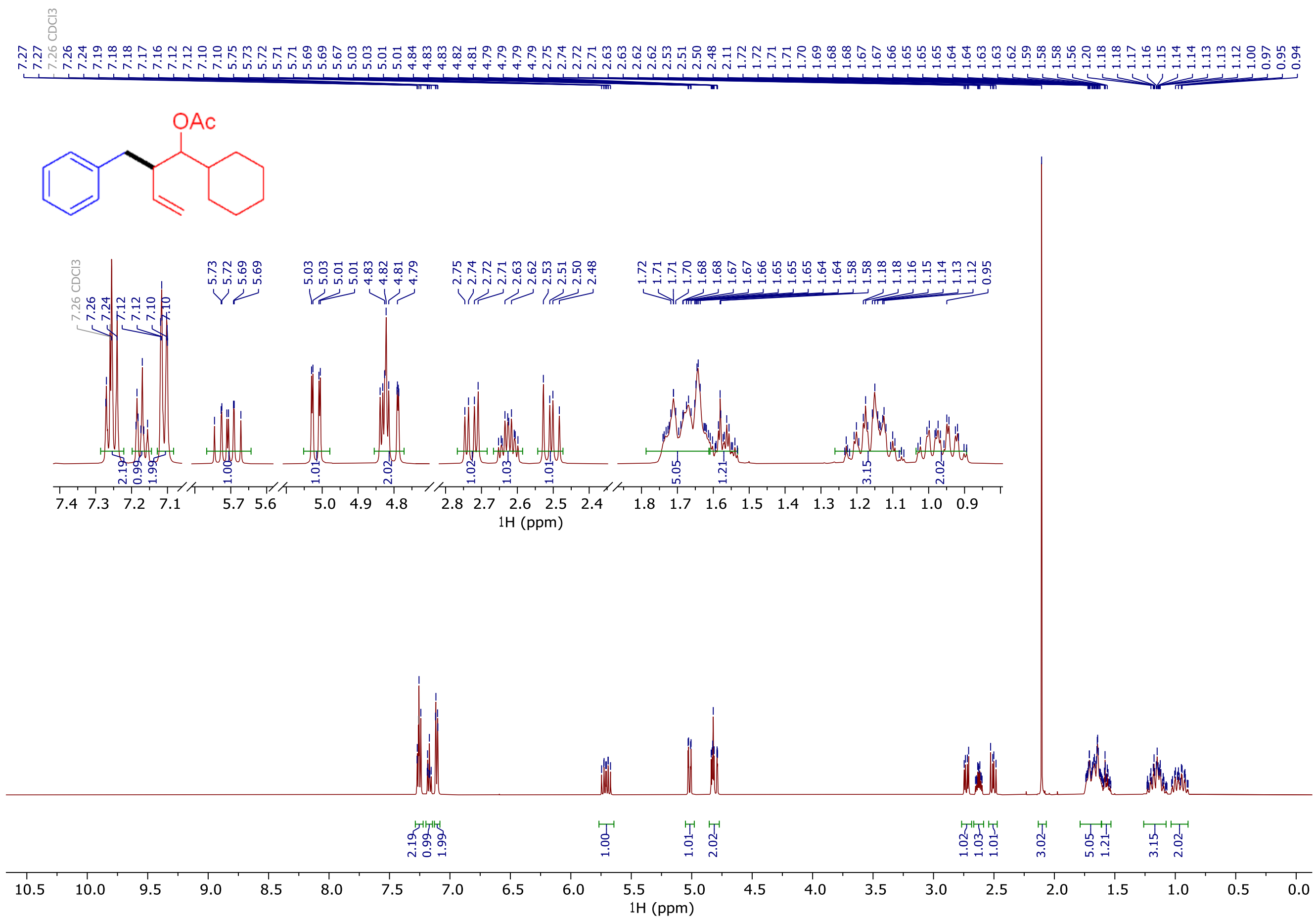

$^{13}C\{^{1}H\}$ NMR (126 MHz, $CDCl_3$) spectrum of **2-benzyl-1-cyclohexylbut-3-en-1-yl acetate (3bm)**

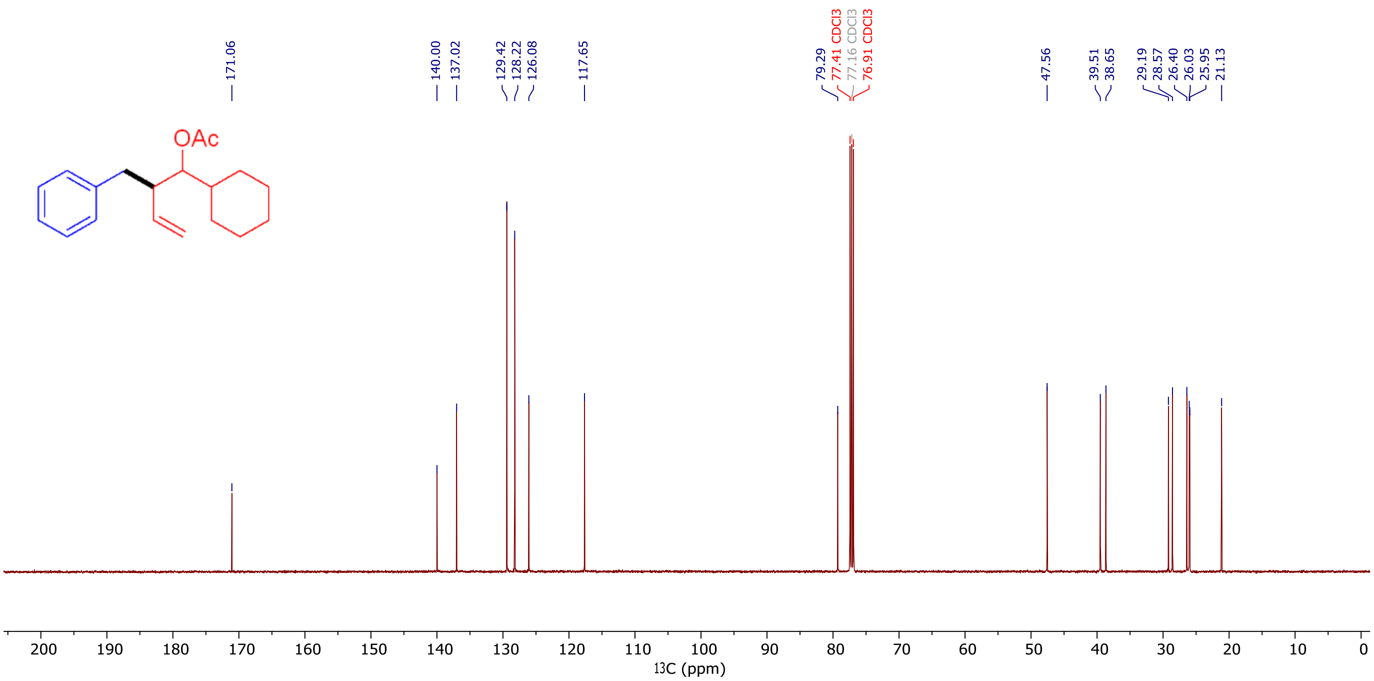

^{1}H NMR (500 MHz, $CDCl_3$) spectrum of **1-(1-phenylbut-3-en-2-yl)cyclohexan-1-ol (3bn)**

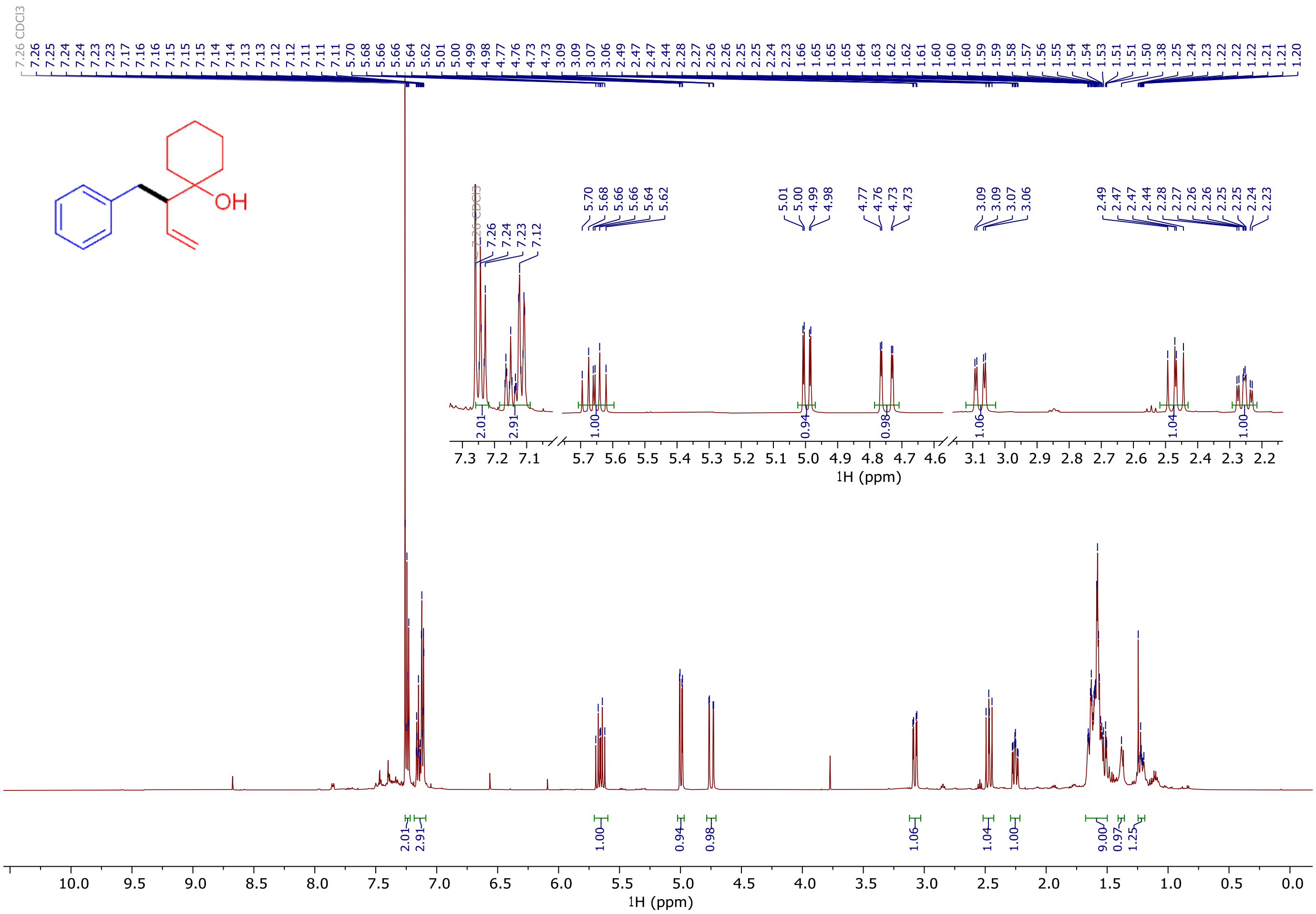

$^{13}C\{^{1}H\}$ NMR (126 MHz, $CDCl_3$) spectrum of **1-(1-phenylbut-3-en-2-yl)cyclohexan-1-ol (3bn)**

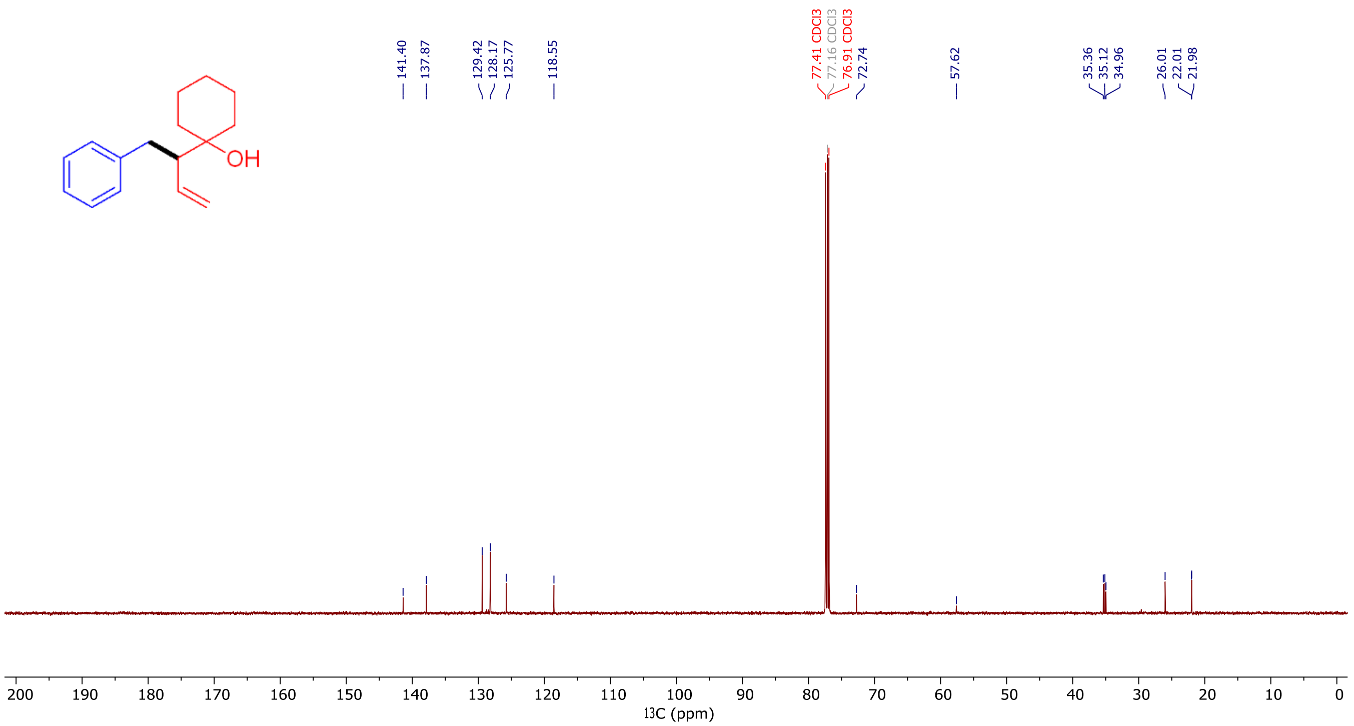

^{1}H NMR (500 MHz, $CDCl_3$) spectrum of **2-benzyl-1-phenylbut-3-en-1-ol (3bo)**

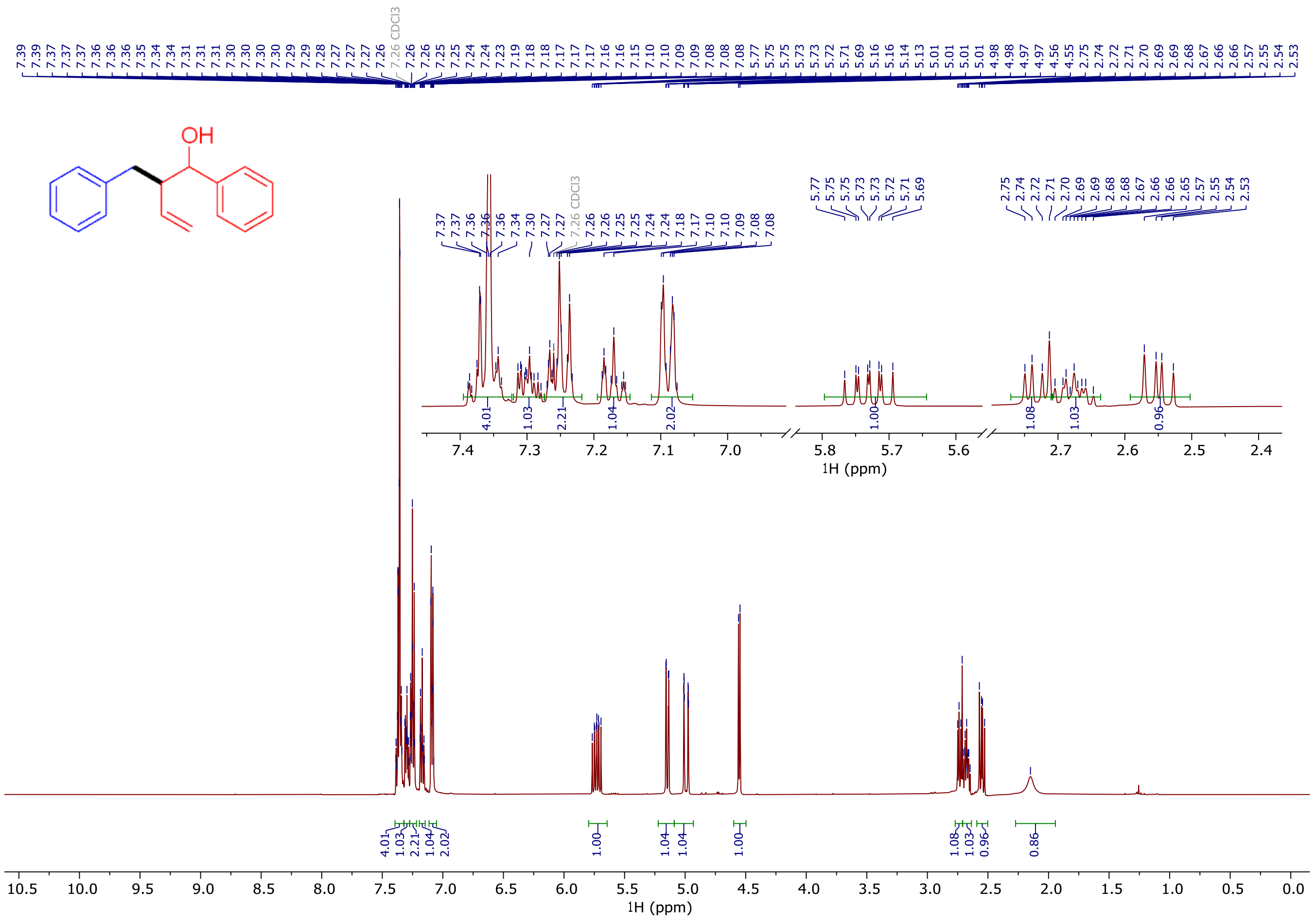

$^{13}C\{^1H\}$ NMR (126 MHz, $CDCl_3$) spectrum of **2-benzyl-1-phenylbut-3-en-1-ol (3bo)**

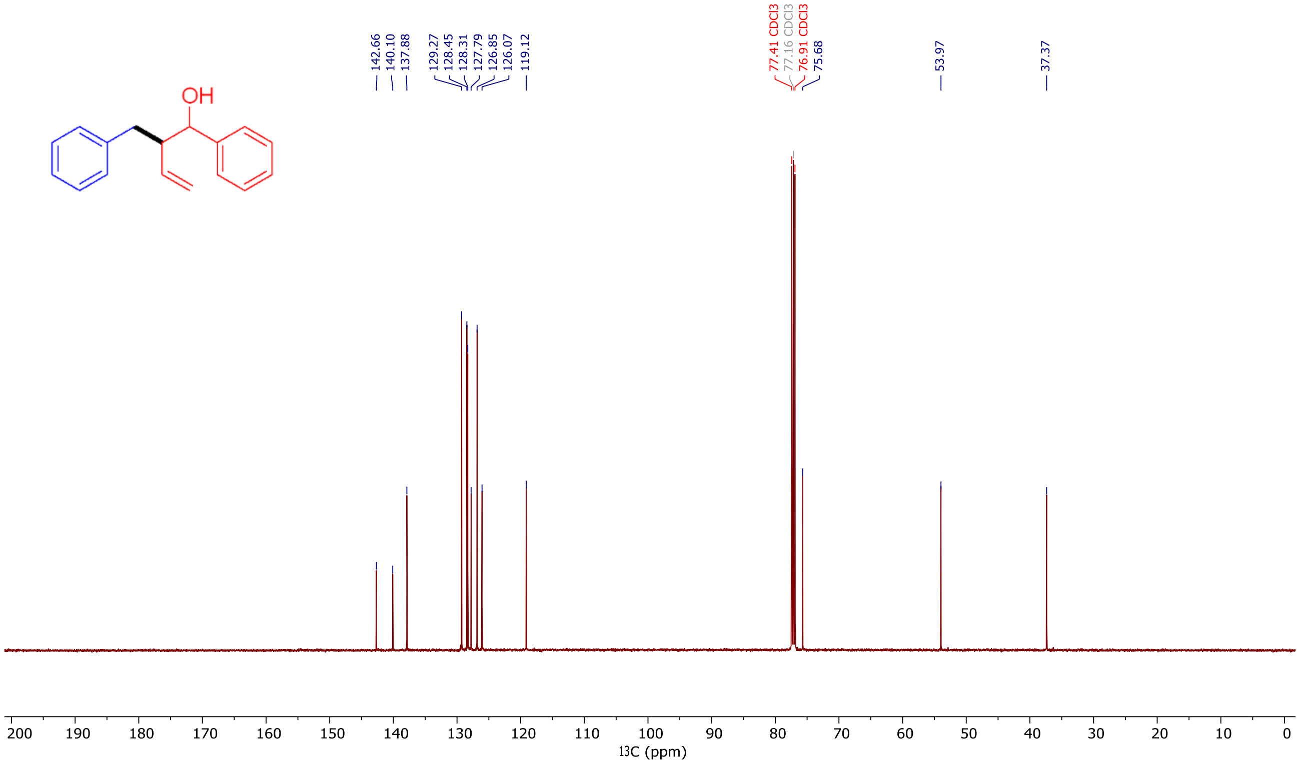

^1H NMR (500 MHz, $CDCl_3$) spectrum of **2-benzyl-1-(4-methoxyphenyl)but-3-en-1-ol (3bp)**

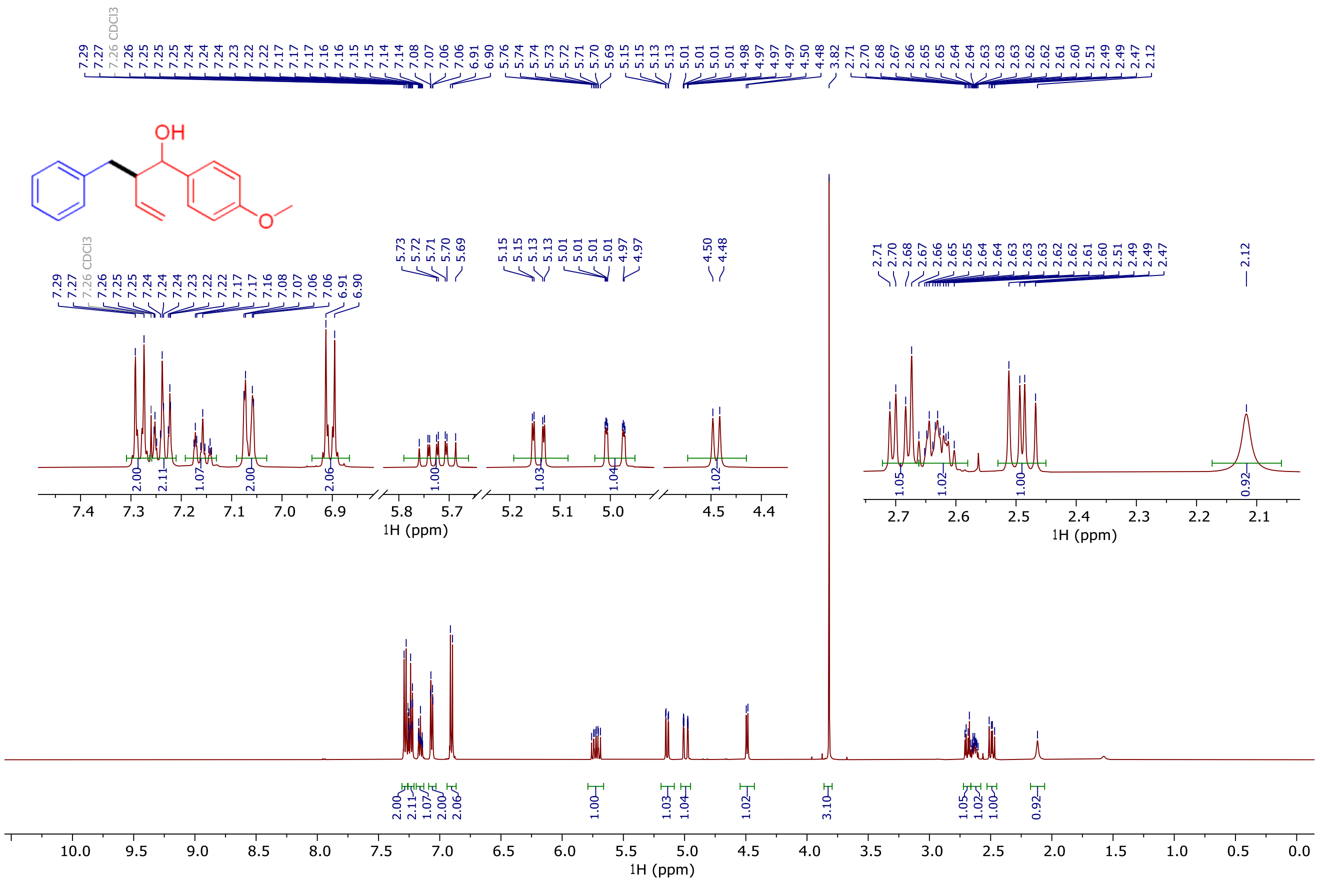

$^{13}C\{^1H\}$ NMR (126 MHz, $CDCl_3$) spectrum of **2-benzyl-1-(4-methoxyphenyl)but-3-en-1-ol (3bp)**

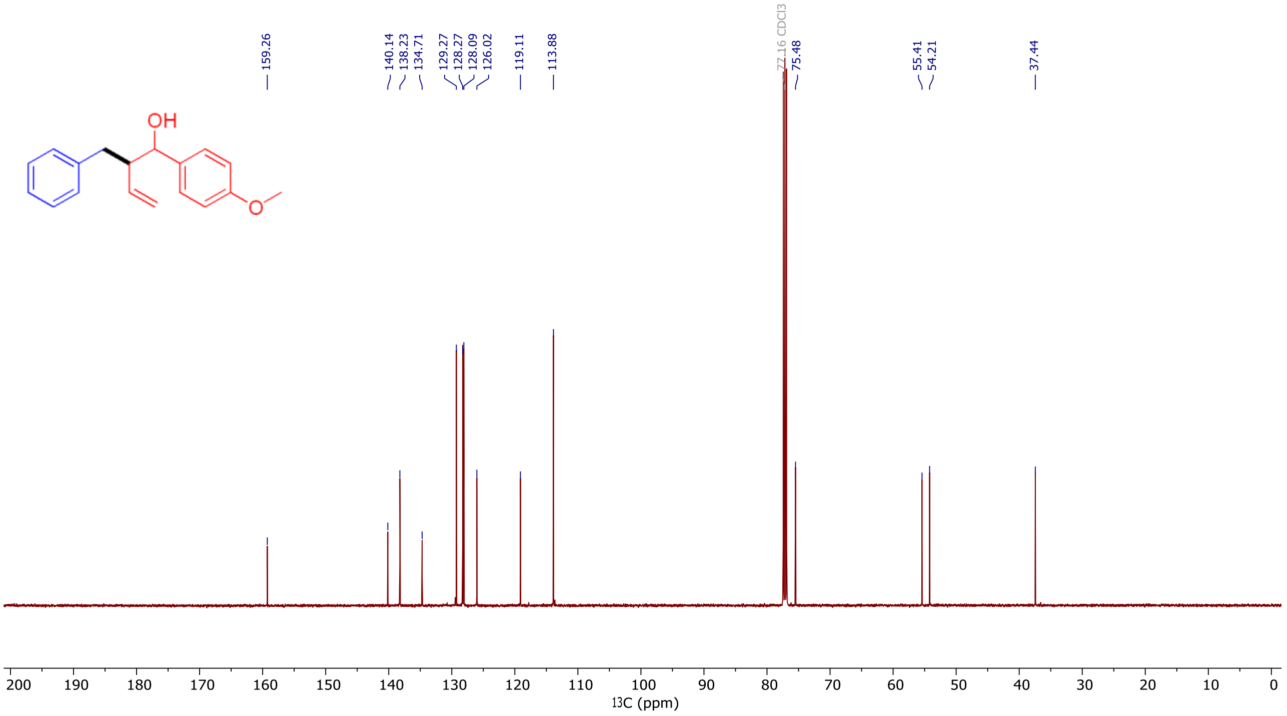

^1H NMR (500 MHz, $CDCl_3$) spectrum of **2-benzyl-1-(4-fluorophenyl)but-3-en-1-ol (3bq)**

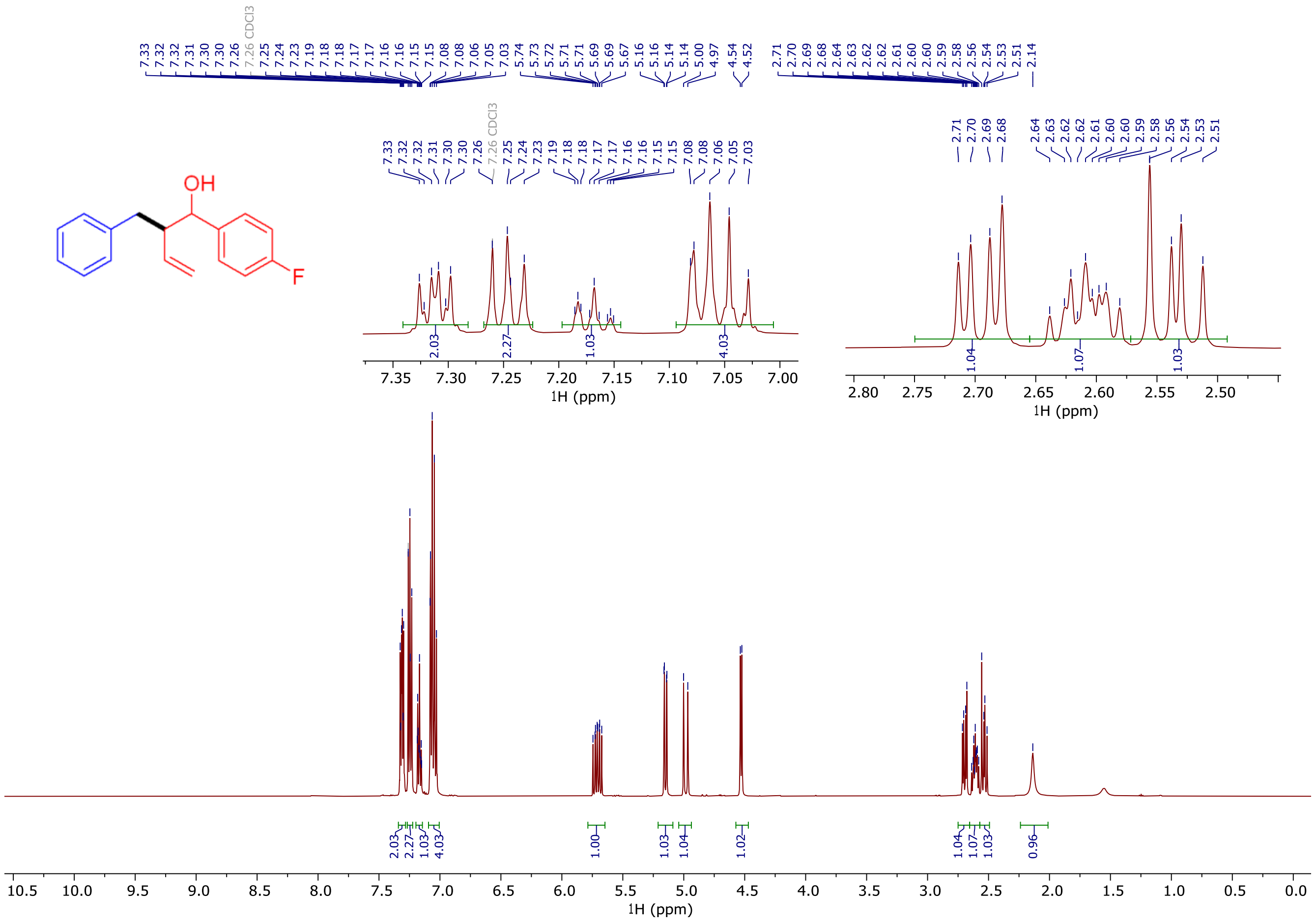

$^{13}C\{^1H\}$ NMR (126 MHz, $CDCl_3$) spectrum of **2-benzyl-1-(4-fluorophenyl)but-3-en-1-ol (3bq)**

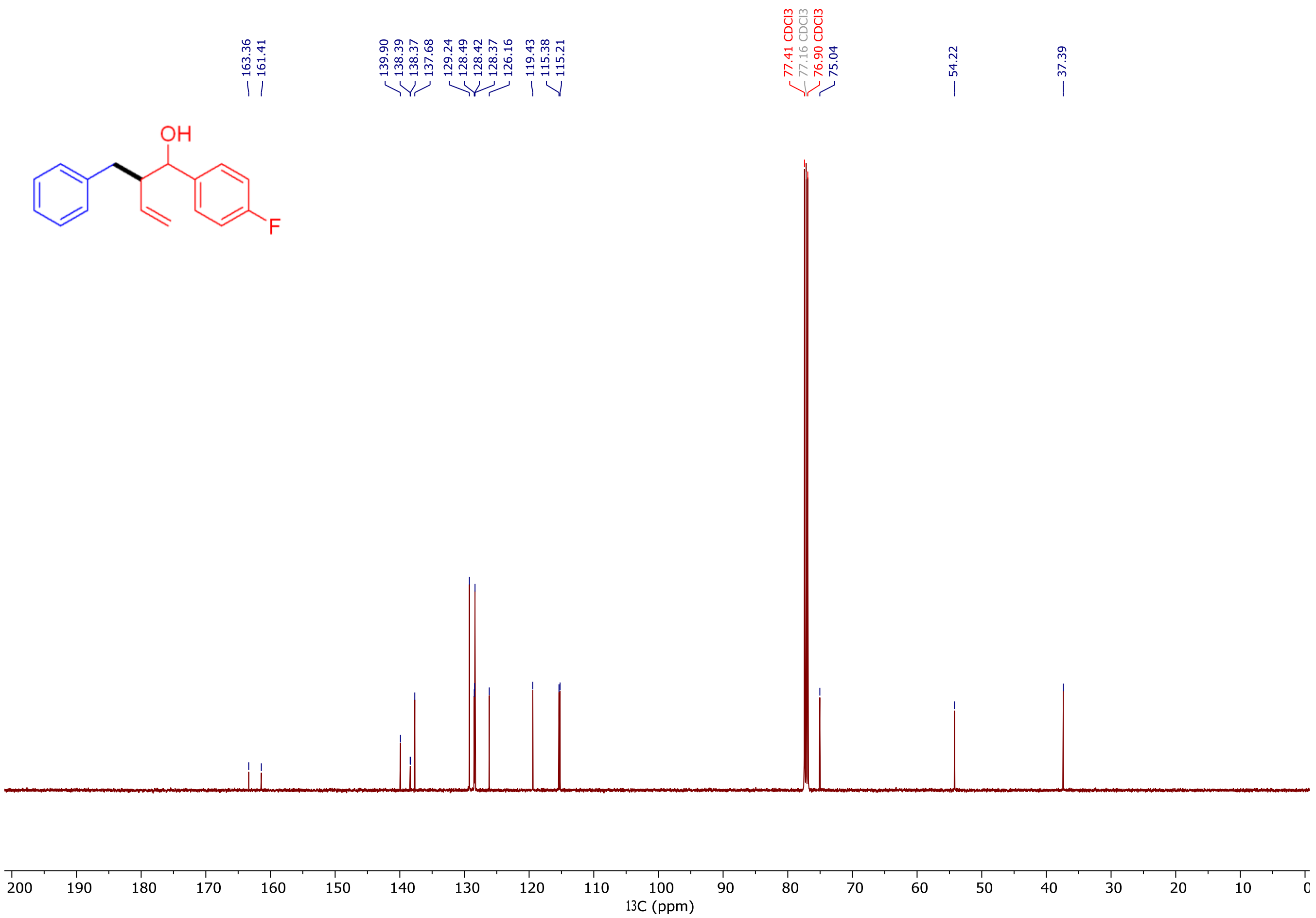

^{19}F NMR (471 MHz, $CDCl_3$) spectrum of **2-benzyl-1-(4-fluorophenyl)but-3-en-1-ol (3bq)**

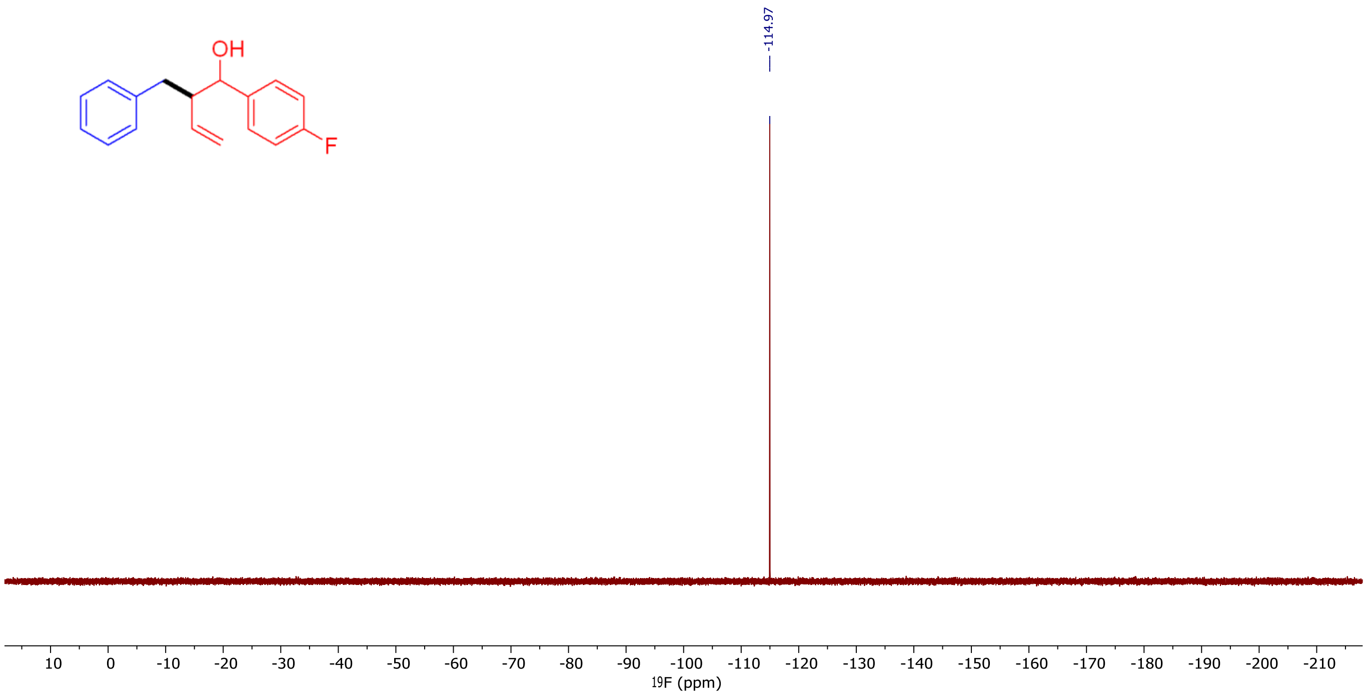

^{1}H NMR (800 MHz, $CDCl_3$) spectrum of **2-benzyl-1-(9-ethyl-9H-carbazol-3-yl)but-3-en-1-ol (3br)**

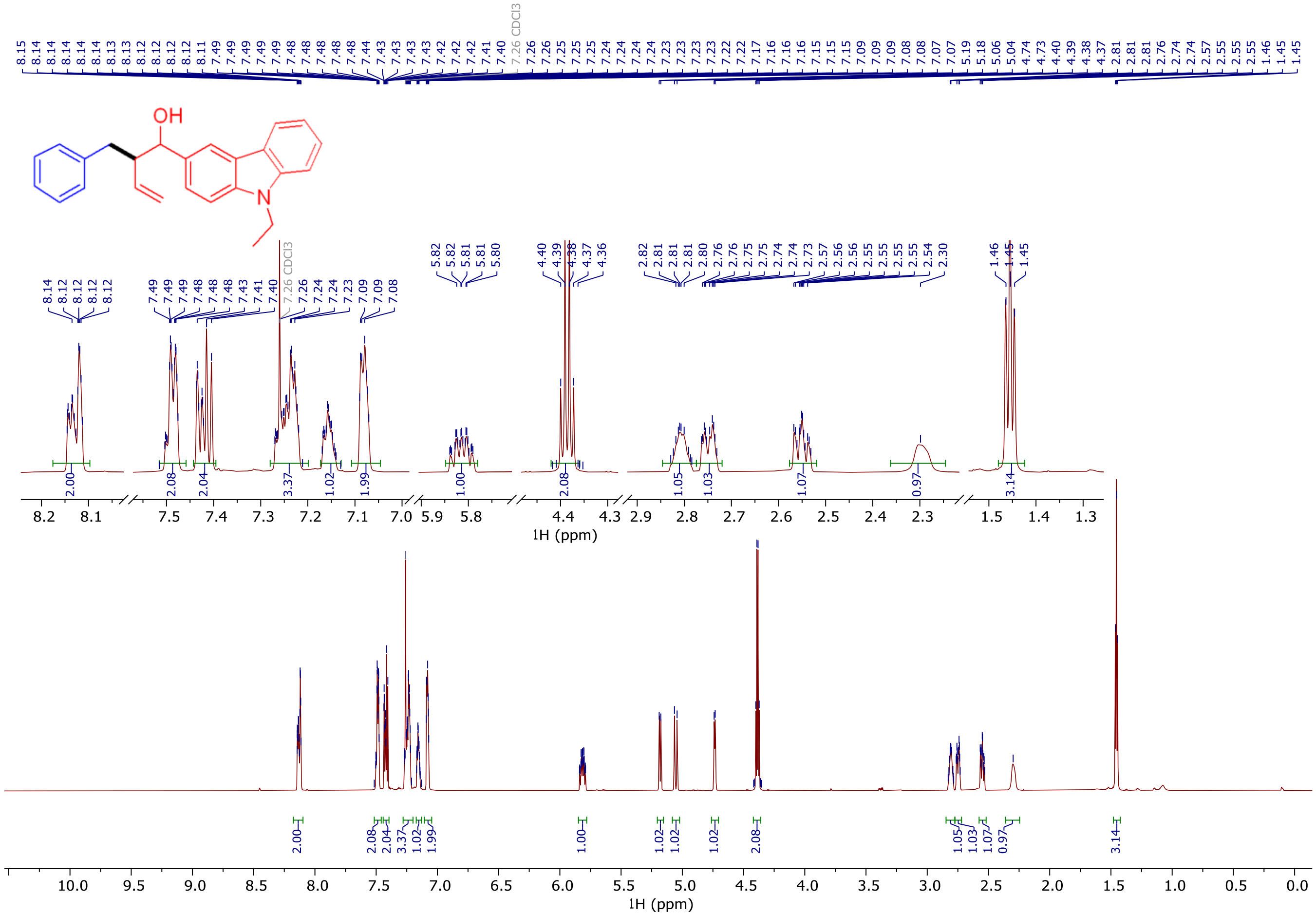

$^{13}C\{^{1}H\}$ NMR (201 MHz, $CDCl_3$) spectrum of **2-benzyl-1-(9-ethyl-9H-carbazol-3-yl)but-3-en-1-ol (3br)**

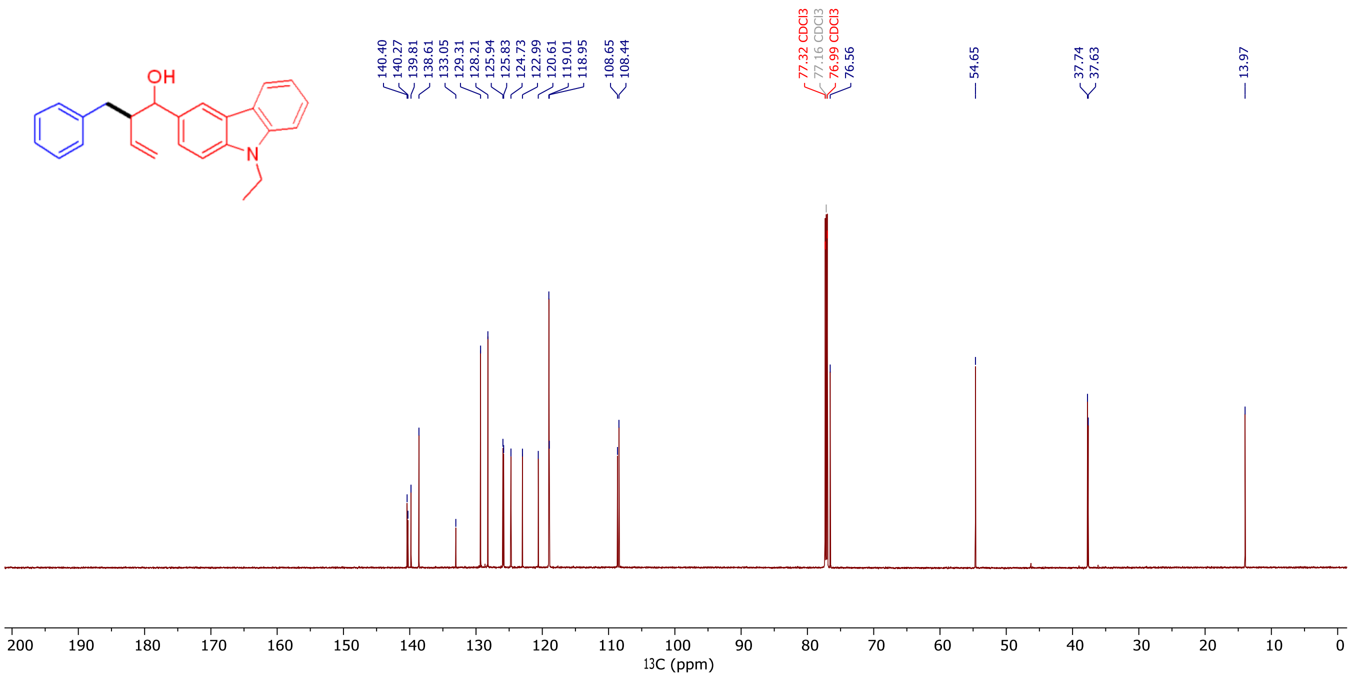

^{1}H NMR (500 MHz, $CDCl_3$) spectrum of **(3R,5R,8R,9S,10S,13R,14S,17R)-17-((2R)-6-benzyl-5-hydroxyoct-7-en-2-yl)-10,13-dimethylhexadecahydro-1H-cyclopenta[a]phenanthren-3-ol (3bs)**

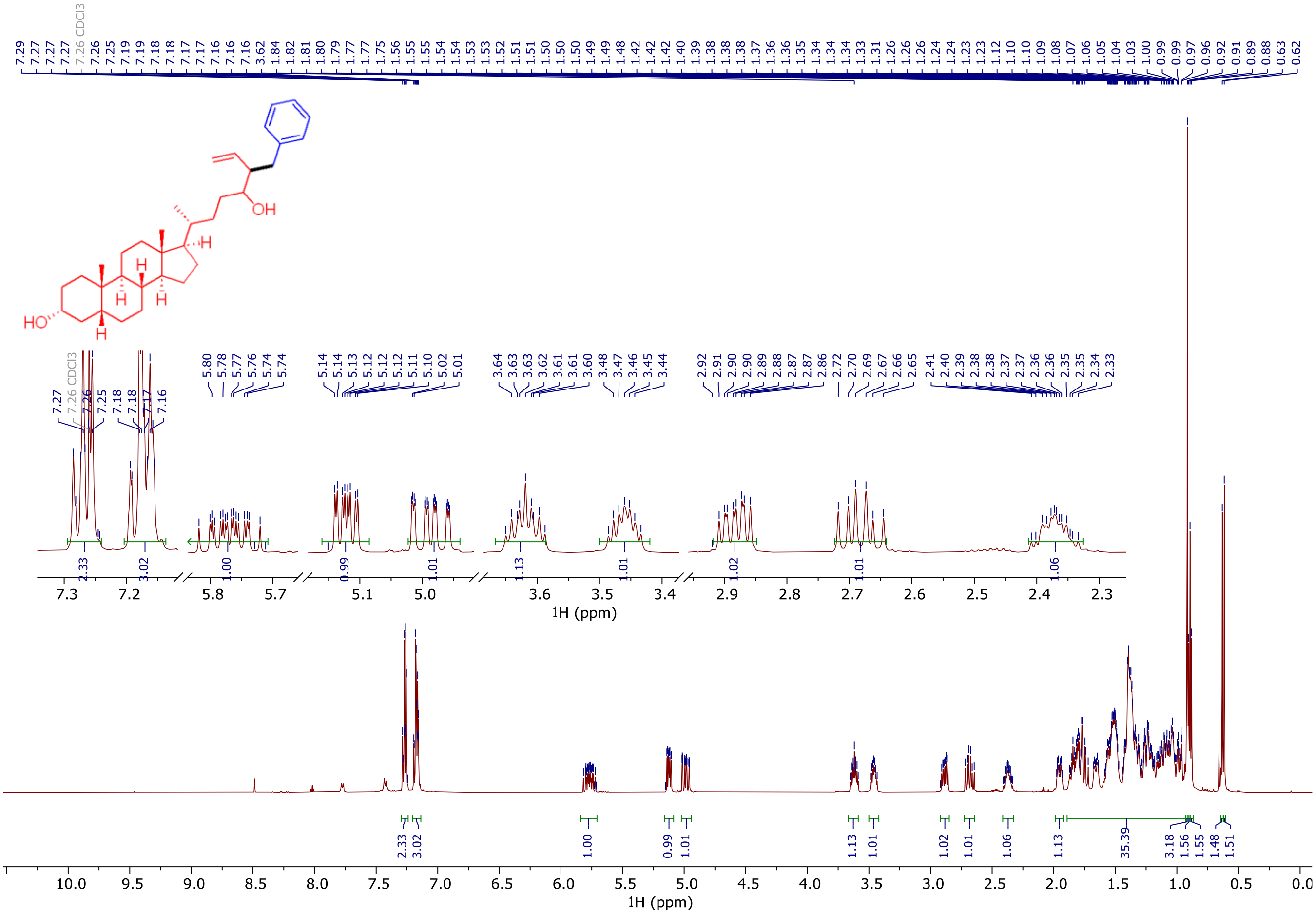

$^{13}C\{^{1}H\}$ NMR (126 MHz, $CDCl_3$) spectrum of **(3R,5R,8R,9S,10S,13R,14S,17R)-17-((2R)-6-benzyl-5-hydroxyoct-7-en-2-yl)-10,13-dimethylhexadecahydro-1H-cyclopenta[a]phenanthren-3-ol (3bs)**

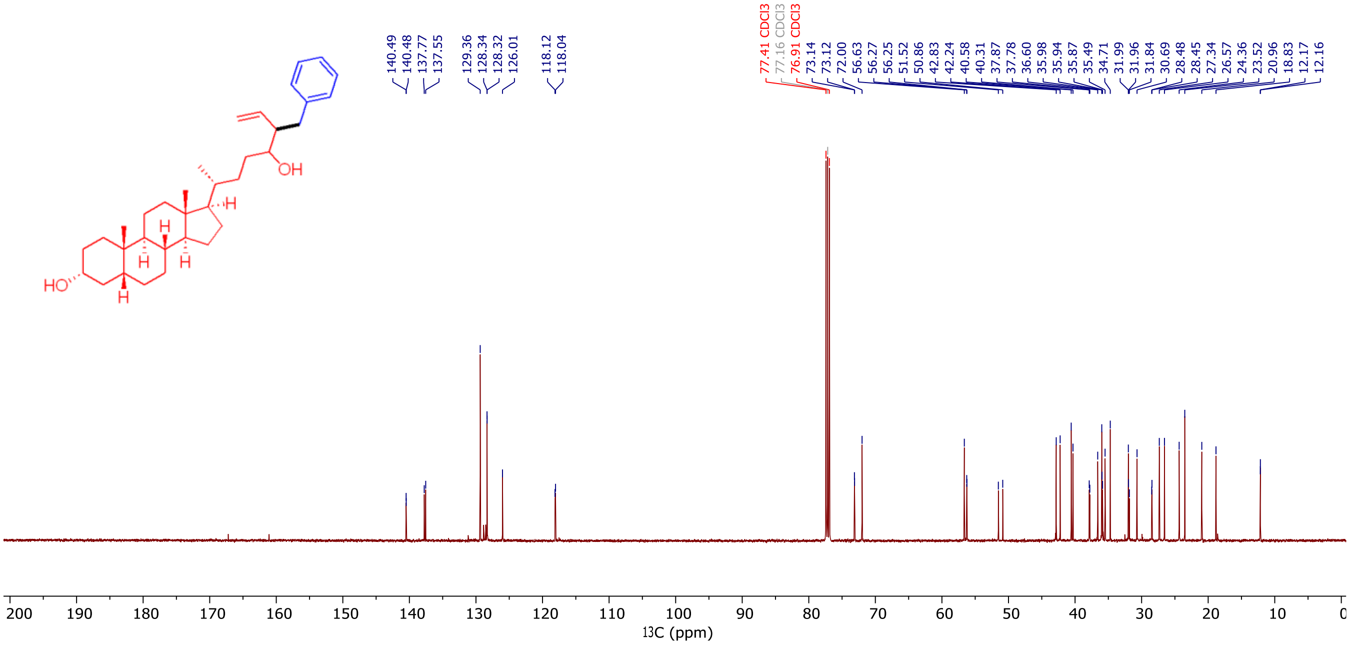

^{1}H NMR (500 MHz, $CDCl_3$) spectrum of **2-benzyl-1-((3aR,5R,5aS,8aS,8bR)-2,2,7,7-tetramethyltetrahydro-5H-bis([1,3]dioxolo)[4,5-b:4',5'-d]pyran-5-yl)but-3-en-1-ol (3bt)**

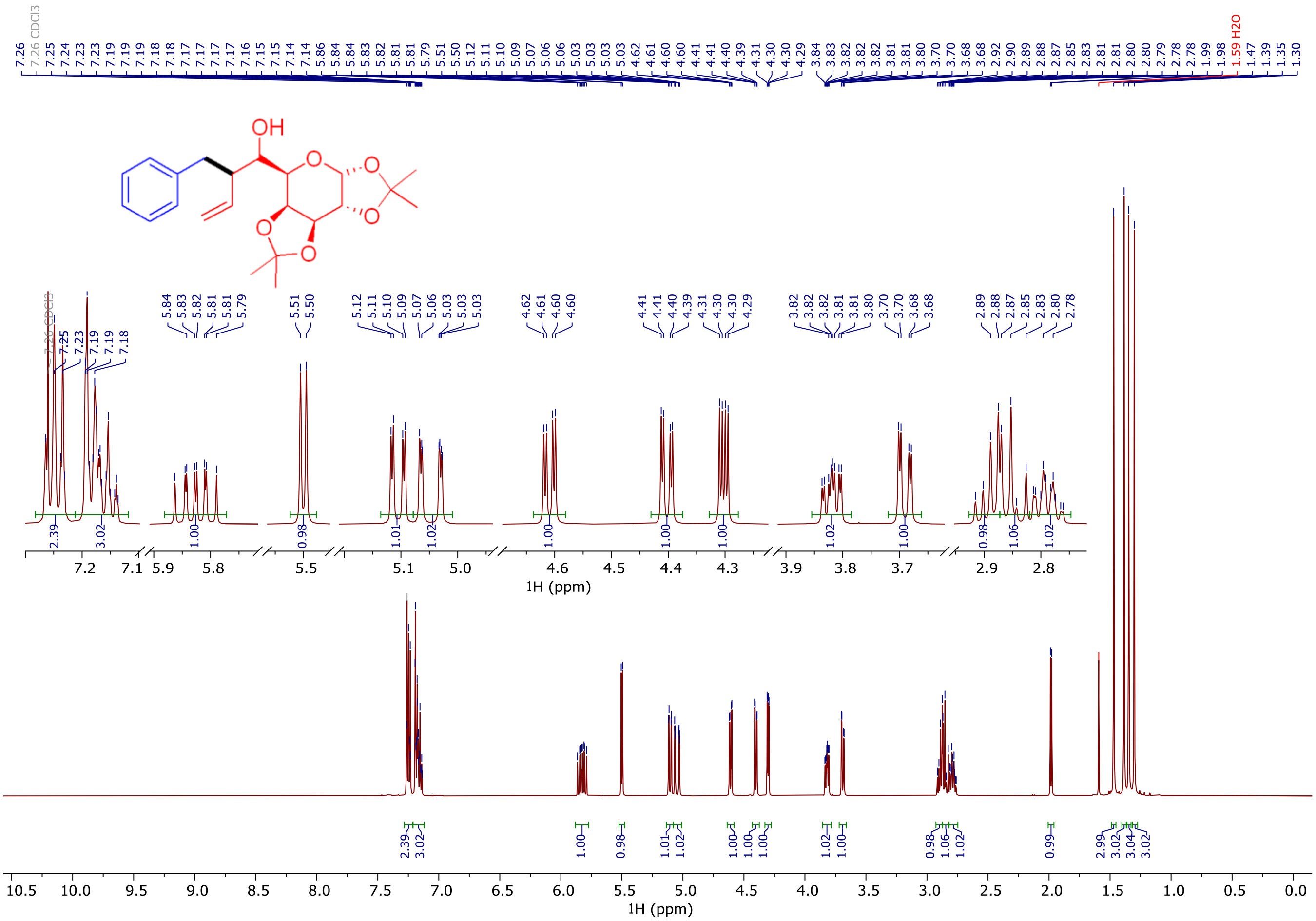

$^{13}C\{^{1}H\}$ NMR (126 MHz, $CDCl_3$) spectrum of **2-benzyl-1-((3aR,5R,5aS,8aS,8bR)-2,2,7,7-tetramethyltetrahydro-5H-bis([1,3]dioxolo)[4,5-b:4',5'-d]pyran-5-yl)but-3-en-1-ol (3bt)**

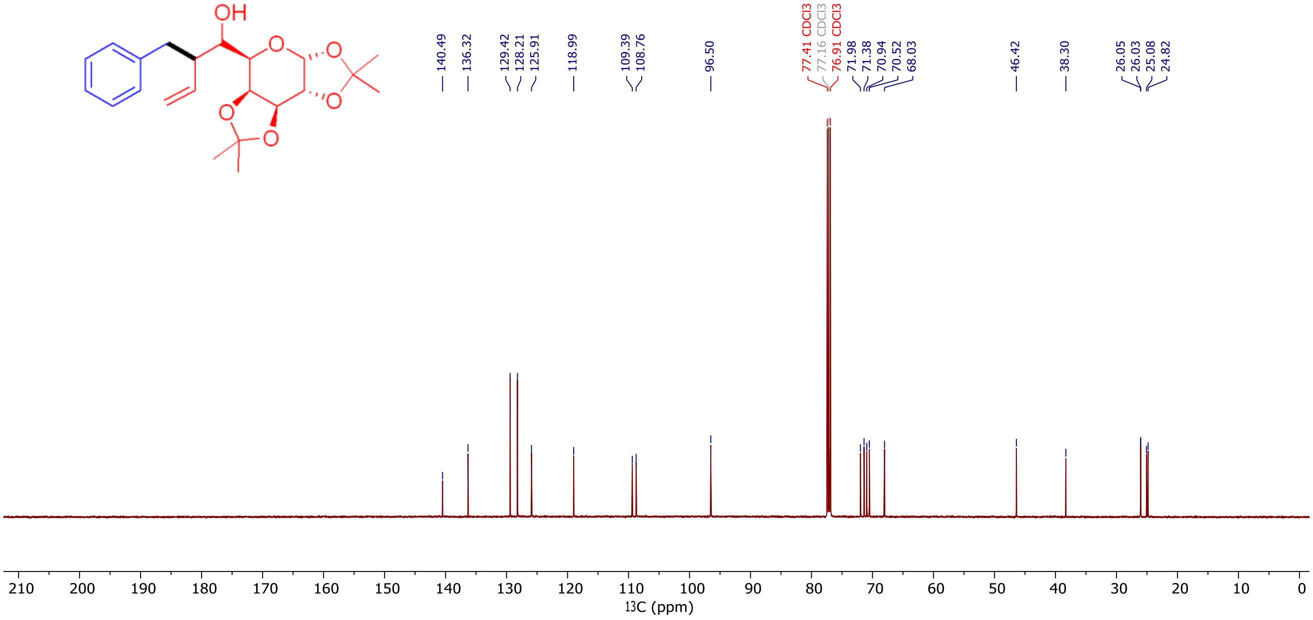

^{1}H NMR (500 MHz, $CDCl_3$) spectrum of **1-(4-((4-allyl-2-methoxyphenoxy)methyl)phenyl)-2-benzylbut-3-en-1-ol (3bu)**

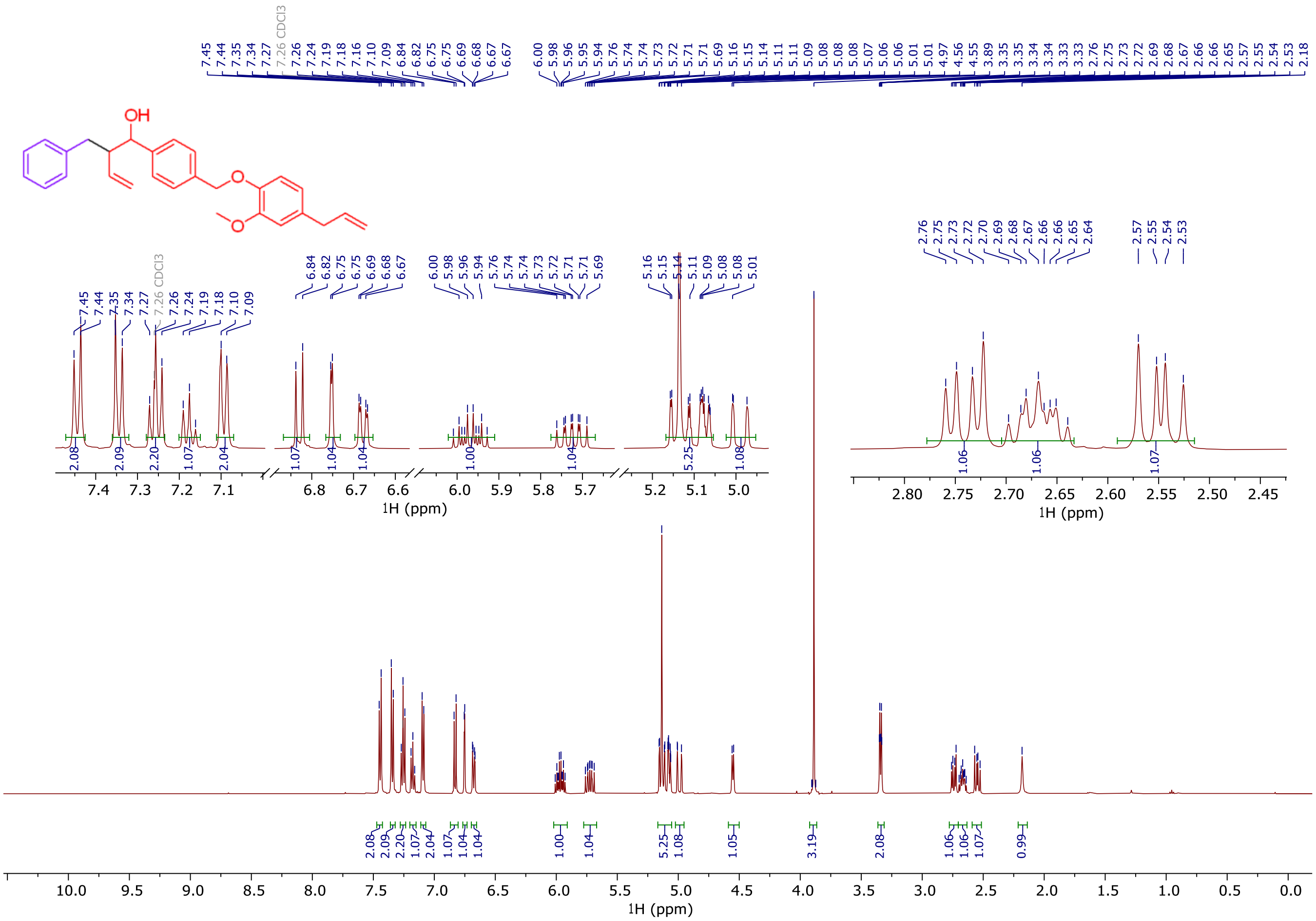

^{13}C{^{1}H} NMR (126 MHz, $CDCl_3$) spectrum of **1-(4-((4-allyl-2-methoxyphenoxy)methyl)phenyl)-2-benzylbut-3-en-1-ol (3bu)**

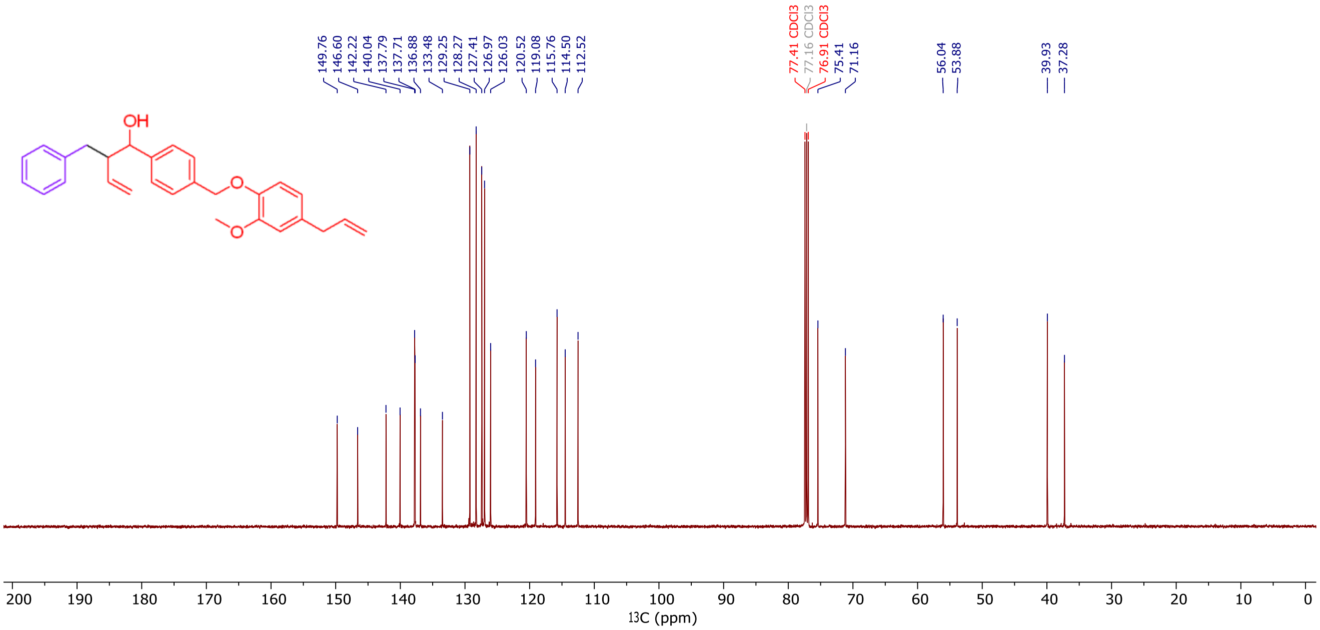

^{1}H NMR (500 MHz, $CDCl_3$) spectrum of **7-(4-(2-benzyl-1-hydroxybut-3-en-1-yl)benzyl)-1,3-dimethyl-3,7-dihydro-1H-purine-2,6-dione (3bv)**

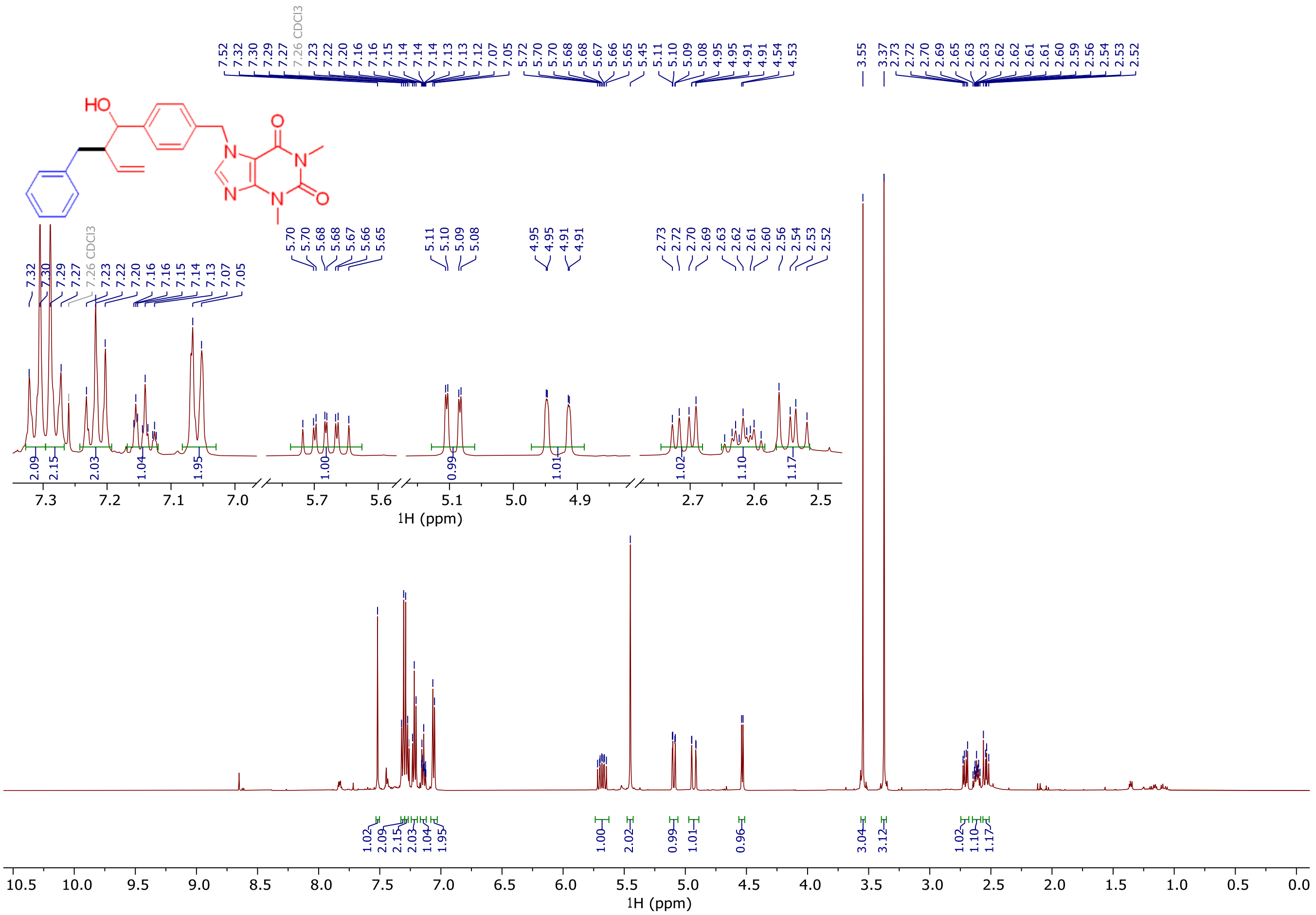

^{13}C{^{1}H} NMR (126 MHz, $CDCl_3$) spectrum **7-(4-(2-benzyl-1-hydroxybut-3-en-1-yl)benzyl)-1,3-dimethyl-3,7-dihydro-1H-purine-2,6-dione (3bv)**

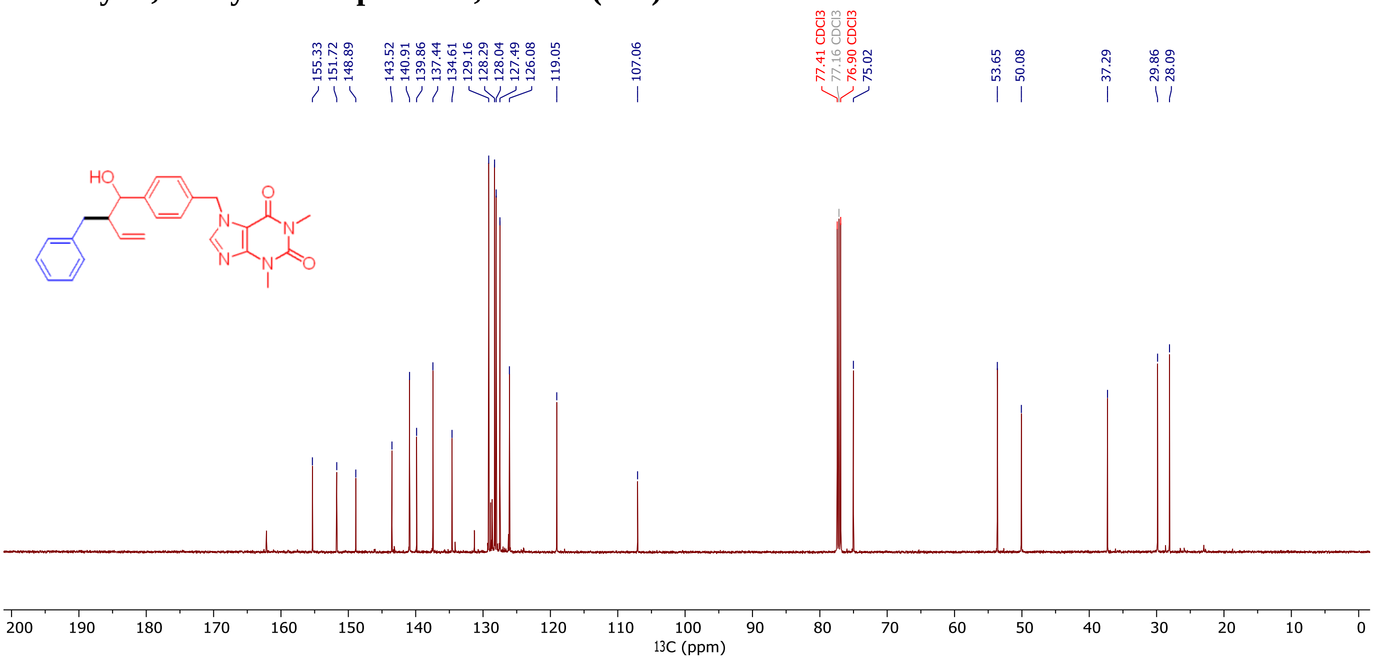

^{1}H NMR (500 MHz, $CDCl_3$) spectrum of **2-benzyl-1-(4-((((R)-2,5,7,8-tetramethyl-2-((4R,8R)-4,8,12-trimethyltridecyl)chroman-6-yl)oxy)methyl)phenyl)but-3-en-1-ol (3bw)**

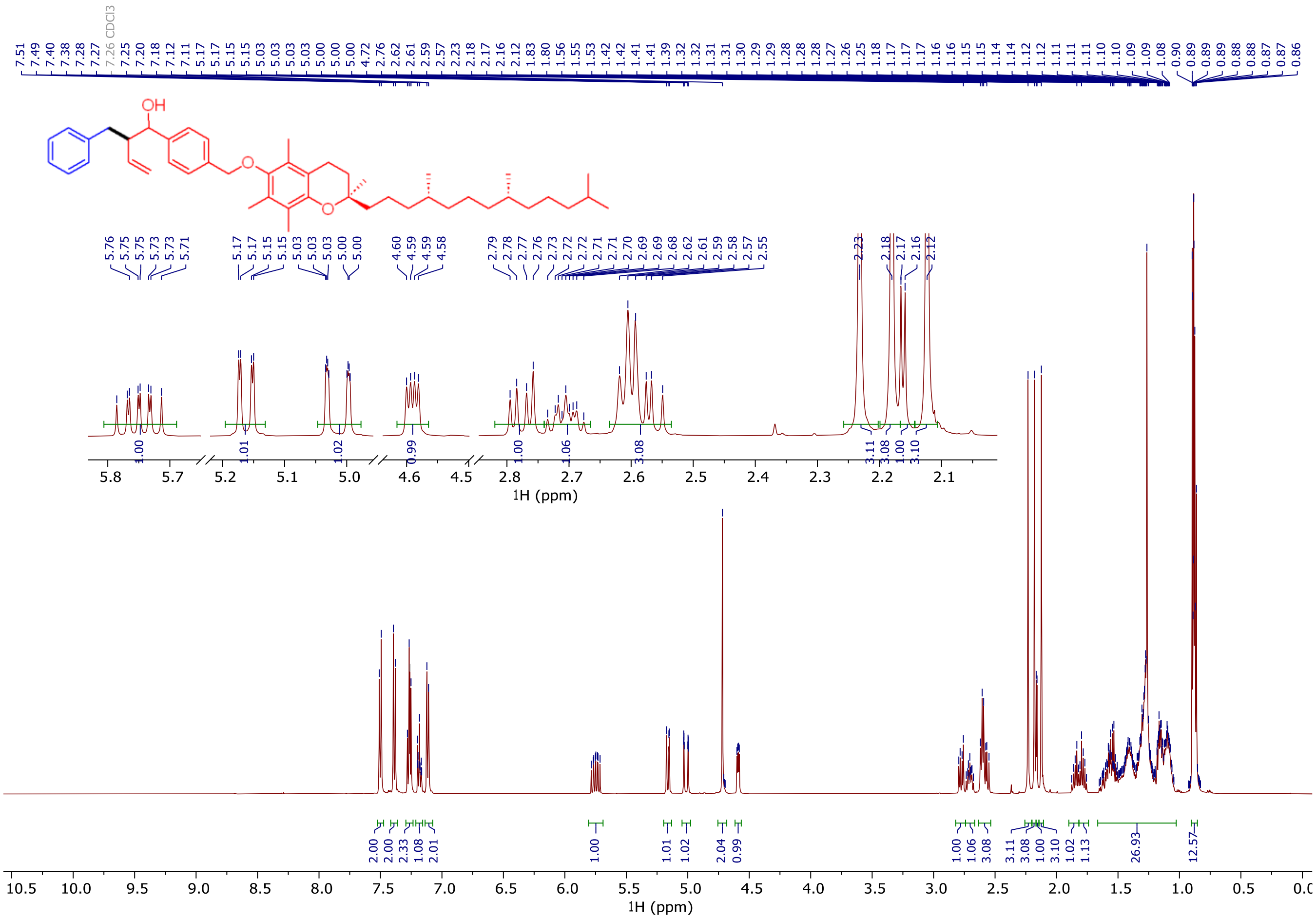

^{13}C{^{1}H} NMR (126 MHz, $CDCl_3$) spectrum of **2-benzyl-1-(4-((((R)-2,5,7,8-tetramethyl-2-((4R,8R)-4,8,12-trimethyltridecyl)chroman-6-yl)oxy)methyl)phenyl)but-3-en-1-ol (3bw)**

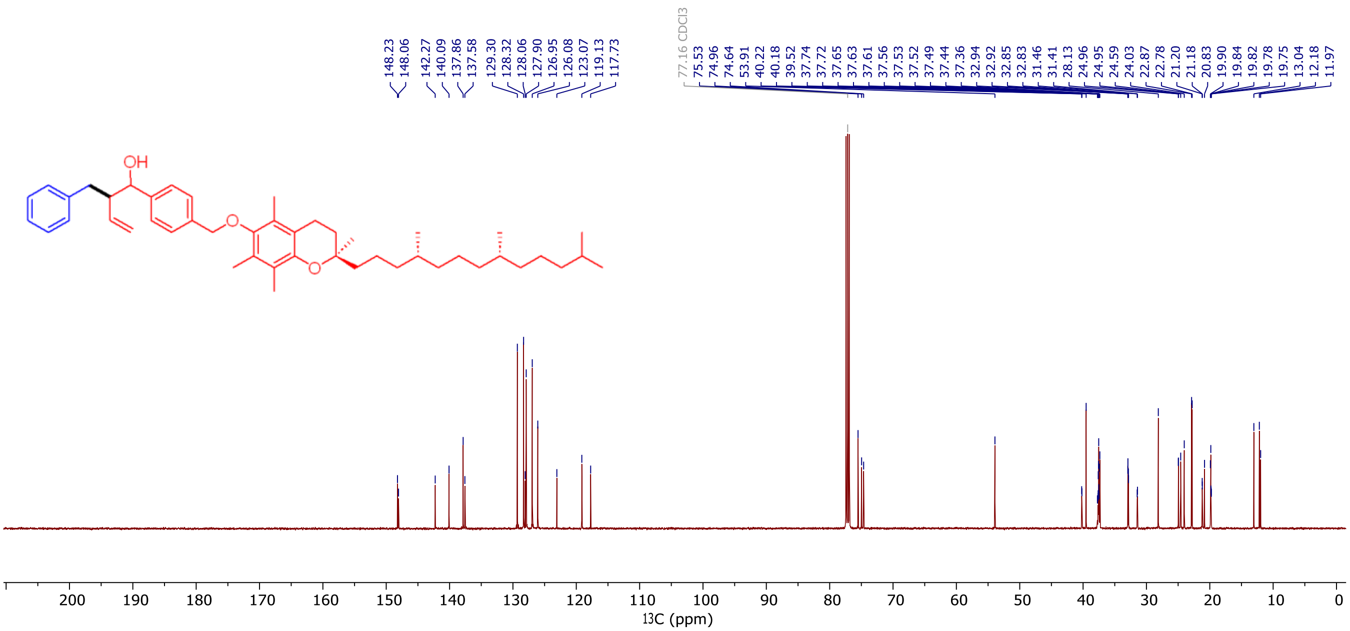

Supplement: SC-015-D4SC05411C-s001 [file SC-015-D4SC05411C-s001.pdf]
